# The Merger of Benzophenone HAT Photocatalysis and Silyl Radical-Induced XAT Enables Both Nickel-Catalyzed Cross-Electrophile Coupling and 1,2-Dicarbofunctionalization of Olefins

**DOI:** 10.1021/acscatal.2c03805

**Published:** 2022-09-01

**Authors:** Alberto Luridiana, Daniele Mazzarella, Luca Capaldo, Juan A. Rincón, Pablo García-Losada, Carlos Mateos, Michael O. Frederick, Manuel Nuño, Wybren Jan Buma, Timothy Noël

**Affiliations:** † Flow Chemistry Group, Van’t Hoff Institute for Molecular Sciences (HIMS), University of Amsterdam, Science Park 904, 1098 XH Amsterdam, The Netherlands; ‡ Centro de Investigación Lilly S.A., Avda. de la Industria 30, Alcobendas-Madrid 28108, Spain; § Small Molecule Design and Development, Eli Lilly and Company, Indianapolis, Indiana 46285, United States; ∥ Vapourtec Ltd. Park Farm Business Centre, Fornham St Genevieve, Bury St Edmunds, Suffolk IP28 6TS, U.K.; ⊥ Molecular Photonics, Van’t Hoff Institute for Molecular Sciences (HIMS), University of Amsterdam, Science Park 904, 1098 XH Amsterdam, The Netherlands

**Keywords:** hydrogen atom transfer, halogen atom transfer, photocatalysis, flow chemistry, cross-electrophile coupling

## Abstract

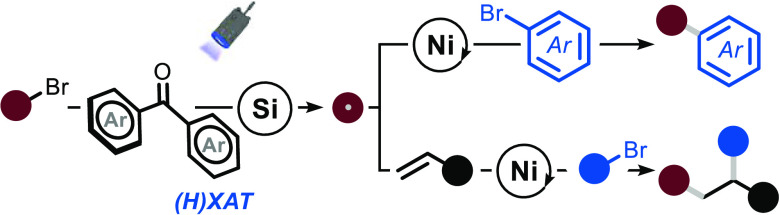

A strategy for both cross-electrophile coupling and 1,2-dicarbofunctionalization of olefins has been developed. Carbon-centered radicals are generated from alkyl bromides by merging benzophenone hydrogen atom transfer (HAT) photocatalysis and silyl radical-induced halogen atom transfer (XAT) and are subsequently intercepted by a nickel catalyst to forge the targeted C(sp^3^)–C(sp^2^) and C(sp^3^)–C(sp^3^) bonds. The mild protocol is fast and scalable using flow technology, displays broad functional group tolerance, and is amenable to a wide variety of medicinally relevant moieties. Mechanistic investigations reveal that the ketone catalyst, upon photoexcitation, is responsible for the direct activation of the silicon-based XAT reagent (HAT-mediated XAT) that furnishes the targeted alkyl radical and is ultimately involved in the turnover of the nickel catalytic cycle.

## Introduction

Over the past years, organic chemistry has been strongly influenced by the development of metallaphotocatalysis,^1^ which exploits the synthetic synergy between photocatalysis and transition-metal catalysis. Notably, by taking advantage of the radical-generating properties as well as the ability to modulate the oxidation state of the metal catalyst,^2^ photocatalysis has greatly expanded our ability to engage various electrophiles and nucleophiles in cross-coupling reactions.

**Figure 1:**
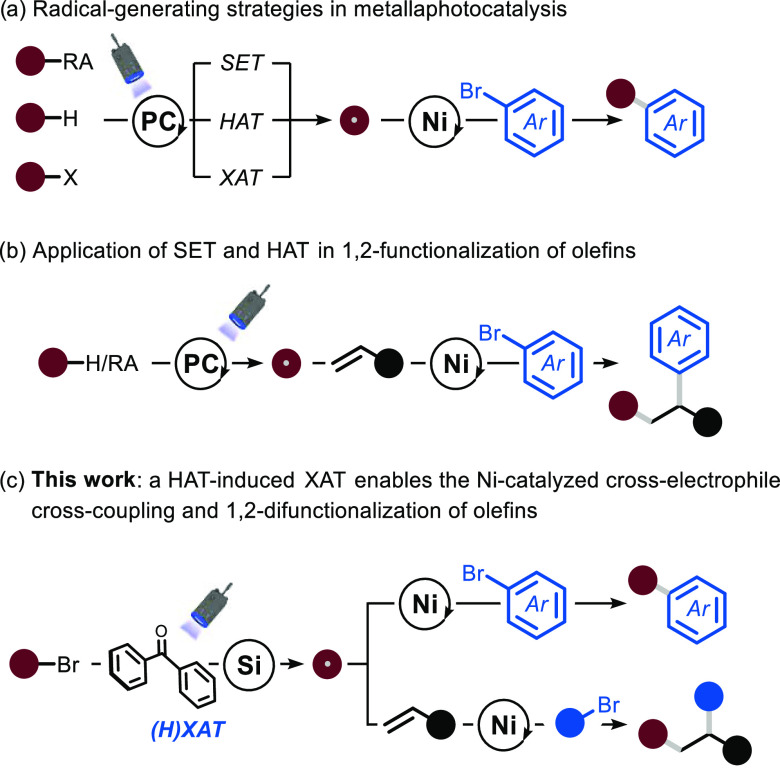
(a) Strategies for generating radicals through metallaphotocatalysis. (b) 1,2-Functionalization reactions enabled by SET and HAT methods. (c) Synergistic use of benzophenone photocatalysis, silane-mediated XAT, and nickel catalysis enables various useful coupling reactions. (H)XAT: HAT-induced XAT, HAT: hydrogen atom transfer, XAT: halogen atom transfer, SET: single electron transfer, RA: redox auxiliaries, PC: photocatalyst.

At the inception of this field,^3^ the photocatalyst was used to activate substrates bearing redox auxiliaries. The presence of such groups would lower the redox potential of the radical precursor, enabling the single electron transfer (SET) from the photocatalyst, and ensure the crucial mesolytic fragmentation to afford the targeted open-shell intermediate (Figure 1a).^4^ This radical can then be intercepted by a transition-metal catalyst and coupled with suitable aryl electrophiles in an overall C(sp^3^)–C(sp^2^) bond formation.^5^ Pioneering works by MacMillan^6a^ and Martin,^6b^ among others,^6^ expanded the range of radical precursors to simple alkanes by exploiting the hydrogen atom transfer (HAT)^7^ abilities of decatungstate^8^ or benzophenone (BP) photocatalysts.^9^ The selectivity of such HAT processes depends on the careful balance between electronic and steric properties of the substrates and the reaction conditions.^10^ In parallel to HAT, halogen atom transfer (XAT)^11^ has been used in metallaphotoredox manifolds as well,^12,13^ enabling the use of alkyl halides as radical precursors. This method relies on the photocatalytic generation of silicon-centered^12^ or α-amino^13^ radicals, which induce homolytic cleavage of the C–X (X: halogen) bond. The use of this radical tool allows the generation of the open-shell species in a programmable fashion, as the selectivity is now dictated by the position occupied by the halogen atom in the radical precursor.

More recently, several groups have focused on expanding metallaphotoredox into a platform that enables the 1,2-functionalization of olefins (Figure 1b).^14^ Indeed, the addition of the photochemically generated radicals and the aryl electrophiles onto an olefin represents a rapid and direct method to construct complex scaffolds from simple and readily available starting materials.

The groups of Molander,^14a^ Nevado,^14b^ Aggarwal,^14c^ Martin,^14d^ and Yuan^14e^ have disclosed elegant approaches based on the SET of a variety of radical precursors such as trifluoroborates, silicon-based reagents, carboxylates, and tertiary alkyl bromides. Kong^14f^ and Molander^14g^ have employed HAT methods to extend this type of protocol to include alkanes as radical precursors. Nonetheless, strategies relying on XAT mechanisms have not yet been applied to promote a difunctionalization of olefin derivatives.

Herein, we describe the development of a simple and straightforward protocol that exploits the synergistic use of triplet excited diaryl ketone^9^ and tris(trimethylsilyl)silane radicals to activate alkyl bromides (Figure 1c). This strategy has been shown to be uniquely effective in both nickel-catalyzed reductive cross-coupling^15^ and nickel-catalyzed 1,2-functionalization reactions^16^ of olefins. Our mechanistic work reveals that the ketone catalyst, upon photoexcitation, is responsible for the direct activation of the silicon-based XAT reagent (overall an (H)XAT) that furnishes the targeted alkyl radical and is ultimately involved in the turnover of the nickel catalytic cycle.

## Results and Discussion

### Direct Cross-Electrophile Coupling

At the outset of our investigations, and in view of the importance of the transformation,^17^ we selected a reductive cross-coupling procedure as a benchmark reaction to test the feasibility of the approach described in Figure 1c.^18^ We started our optimization endeavors by selecting alkyl bromide **1** and aryl bromide **2** as model substrates. Representative entries are shown in Table 1, but a comprehensive survey of reaction conditions is reported in the Supporting Information. We selected benzophenone derivative **I** as a HAT photocatalyst, tris(trimethylsilyl)silane as the XAT promoter, nickel-based catalyst **II** as the transition-metal catalyst, and 2,6-lutidine as a homogeneous base to ensure the removal of the hydrobromic acid developed during the reaction. The reaction mixture was irradiated with a near-UV light source (λ = 390 nm) for 16 h. Under these conditions, product **3** was obtained in 75% yield (72% after isolation, entry 1).

**Table 1:**
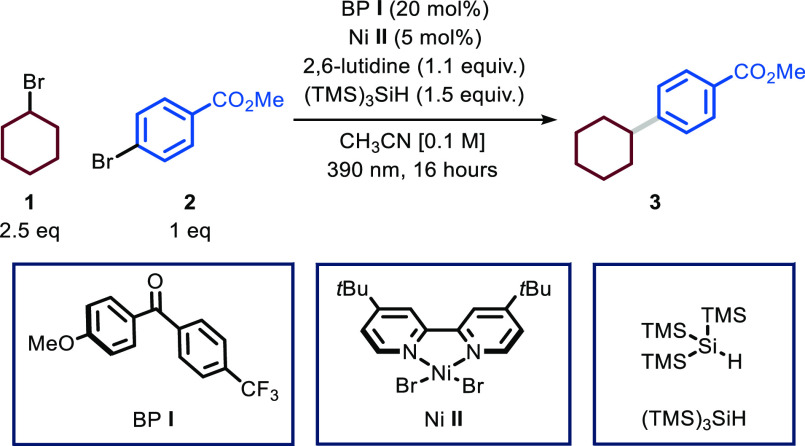
Optimization of the Reaction Conditions

entry	deviation	**3** yield (%)^a^^,^^b^
**1**	**none**	**75 (72)**
2	1.5 equiv of **1**	51
3	1.1 equiv of (TMS)_3_SiH	60
4	Ph_3_SiH instead of (TMS)_3_SiH	20
5	acetone in place of CH_3_CN	67
6	Vapourtec 365 nm 45 min	74
7	no **I**, **II**, 2,6-lutidine or (TMS)_3_SiH	
8	no light or no light at 60 °C	

a **1** (1.25 mmol), **2** (0.5 mmol), BP **I** (20 mol %), Ni **II** (5 mol %), 2,6-lutidine (0.55 mmol), (TMS)_3_SiH (0.75 mmol) at room temperature, 390 nm for 16 h.

b NMR yields using trichloroethylene as an external standard. Yield of the isolated compound is given in parentheses.

Reducing the amounts of alkyl bromide or silane resulted in lower yields (entries 2 and 3). Different silanes (e.g., Ph_3_SiH) were also evaluated but did not lead to efficient formation of the product (entry 4). Various HAT catalysts were tested and proved less effective than BP **I** (see the Supporting Information). The replacement of acetonitrile with acetone gave almost identical results (entry 5). Separate control experiments, each carried out omitting an individual component, revealed that light, **I**, **II**, base, and silane were all essential for the observed reactivity (entries 7 and 8). While these reactions can be smoothly carried out in batch, the homogeneity of the reaction mixture prompted us to investigate the possible development of a procedure in flow to reduce the reaction times and to increase the productivity of the overall process.^19^ For this purpose, we used a commercially available Vapourtec UV-150 reactor (PFA, *V* = 10 mL, ID = 0.80 mm), equipped with a UV lamp (λ = 365 nm, 16 W) and, after a short optimization of the residence times, found that the reaction could be readily performed with similar efficiency in only 45 min (entry 6, flow rate 0.22 mL min^–1^).

**Figure 2:**
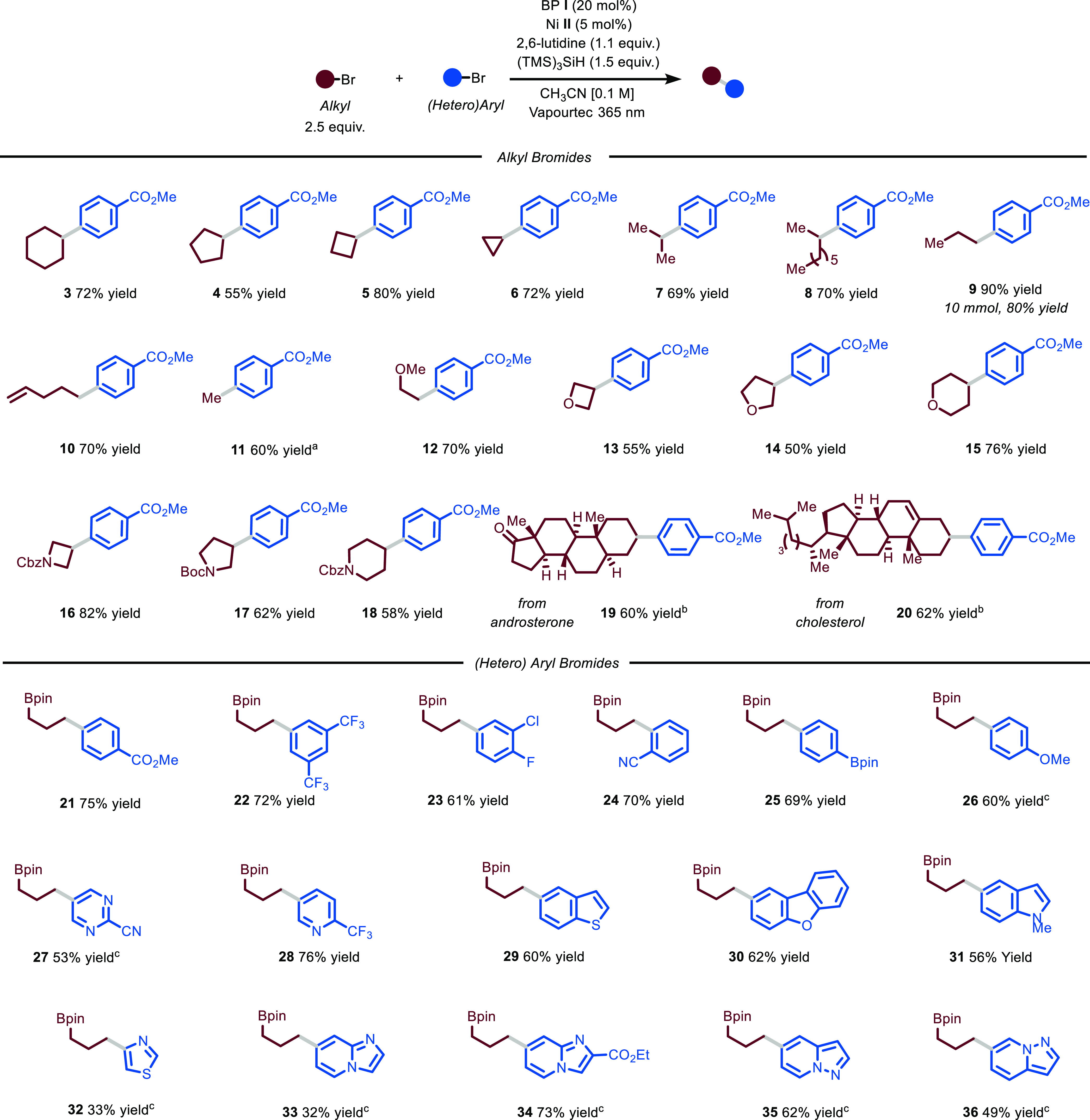
Survey of the aryl bromide and alkyl bromide reaction partners for the photocatalytic C(sp^2^)–C(sp^3^) cross-coupling protocol. Reaction conditions: aryl bromide (0.5 mmol), BP **I** (20 mol %), Ni **II** (5 mol %), 2,6-lutidine (1.1 equiv), (TMS)_3_SiH (1.5 equiv) at room temperature (rt) in Vapourtec UV-150 reactor (λ = 365 nm, 16 W, reactor volume: 10 mL, flow rate: 0.22 mL min^–1^, τ: 45 min). Yields refer to isolated products. ^a^MeOTs as coupling partner (2.5 equiv) and TBABr as bromide source (2.5 equiv). ^b^Reactions performed in batch (16 h) using PhCF_3_ as solvent (see the Supporting Information). ^c^Reactions performed in batch (16 h) using Na_2_CO_3_ (1.5 equiv) instead of 2,6-lutidine. Bpin: Pinacol boronic ester, TBABr: tetrabutylammonium bromide.

With suitable flow conditions in hand, we set off to interrogate the scope of the transformation (Figure 2). First, we combined methyl 4-bromobenzoate **2** with a diverse set of secondary cyclic alkyl bromides and found that the system is performing well regardless of the ring size (**3**–**6**, 55–80% yield).

Also, acyclic secondary alkyl substrates were effective coupling partners (**7**–**8**, 69–70% yield). Primary alkyl derivatives proved to be excellent reaction partners as well (**9**–**11**, 60–90% yield), including the methylation of aryl bromides with methyl tosylate in the presence of tetrabutylammonium bromide as bromide source (**11**, 60% yield). Notably, both primary and secondary oxygen-containing bromides were coupled with methyl 4-bromobenzoate **2** in good yields (**12**–**15**, 55–76% yield), showing no byproducts derived from competing HAT. Medicinally relevant moieties, such as azetidine, pyrrolidine, and piperidine, can be readily engaged in this protocol (**16**–**18**, 58–82% yield). To further highlight the synthetic utility of this process, we pursued the arylation of both androsterone and cholesterol bromides. Due to their low solubility in CH_3_CN, the reaction was conducted in batch using PhCF_3_ as solvent (**19** and **20**, 60–62%).

We then turned to assess the scope in terms of (hetero)aryl bromides and, to this end, we decided to use bromopropyl boronic pinacol ester to incorporate a functional handle for subsequent functionalization. Using identical microfluidic conditions, the reaction with model aryl bromide **2** gave 75% yield for compound **21**. Double substitution at the meta positions is well tolerated (**22**, 72% yield). Interestingly, polyhalogenated arenes were solely functionalized at the C–Br bond (**23**, 61% yield), providing opportunities for sequential decoration of the arene core using, for example, classical cross-coupling conditions. Bromoarenes bearing substituents at the *ortho* position were smoothly cross-coupled (**24**, 70% yield), indicating that the protocol is not particularly sensitive to steric hindrance. Aryl bromides bearing electron-donating groups were also amenable to the reaction protocol: while pinacol boronic ester was well tolerated (**25**, 69% yield), 4-bromo anisole reacted more slowly requiring 16 h to yield the target compound **26** in 60% yield. Notably, also heteroarenes with different electronic properties could be readily functionalized using this photocatalytic protocol. For instance, electron-poor six-membered heteroaromatics, such as pyrimidines and pyridines, were efficiently converted into the desired products **27** and **28** (53–76% yield). Moreover, electron-rich heteroaromatics, such as benzofuran, benzothiophene, and methylated indole, proved to be suitable substrates for the process, leading to compounds **29**, **30**, and **31** in good chemical yields (60, 62, and 56% yields, respectively). Also, thiazoles (**32**, 33% yield), imidazo[1,2-*a*]pyridines (**33–34**, 32–73% yield), and pyrazo[1,2-*a*]pyridines (**35**–**36**, 49–62% yield) could be readily cross-coupled in synthetically useful yields. Finally, we performed a 20-fold scale-up using our continuous-flow protocol; this allowed us to isolate larger quantities of compound **9** simply by extending the total collection time in flow (see the Supporting Information for further details).^20^

### 1,2-Dicarbofunctionalization of Olefins

After having established catalytic conditions for the reductive cross-coupling between alkyl and aryl bromides, we sought to further demonstrate the applicability of our approach to other reaction classes. Specifically, we wondered whether, using a similar reaction cocktail, we could promote the reductive 1,2-difunctionalization of olefins. The implementation of such a process could enable the construction of two new chemical bonds in a single operation, enabling the rapid buildup of molecular complexity starting from simple and widely available substrates. As a testament to the synthetic relevance of these reactions, several groups have focused on expanding photocatalytic cross-coupling procedures to olefin difunctionalization reactions.^14^ However, the majority of the reported methods are either relying on substrates suitable for single electron oxidation, such as trifluoroalkyl borates,^14a^ carboxylates,^14c^ or on substrates prone to HAT.^14f,14g^ In fact, less attention has been devoted to the use of substrates prone to reduction, such as alkyl bromides. One report by the group of Martin highlighted the possibility to engage these derivatives in a metallaphotoredox process.^14d^ However, as the method relies on SET to activate the substrate, only tertiary bromides, which possess an accessible redox potential, can be activated. As our method is based on a XAT activation step, we would not rely on the redox properties of the target substrate, but solely on the bond dissociation energy of the C–X bond.

With this idea in mind, we started our investigation by exploring the reaction between cyclohexyl bromide **1**, *tert*-butyl acrylate **37**, and aryl bromide **2** in PhCF_3_. Similar to the previous protocol, we selected BP **I** as a HAT photocatalyst, tris(trimethylsilyl)silane as the XAT promoter, nickel-based catalyst **II** as the transition-metal catalyst, and 2,6-lutidine as a homogeneous base (Table 2).

**Table 2:**
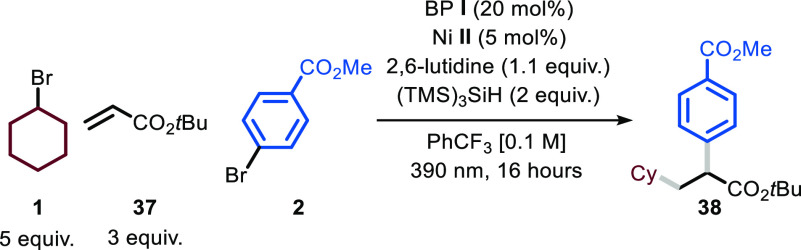
Optimization of the Reaction Conditions

entry	deviation	yield (%)^a^^,^^b^
1	none	58
2	acetone as solvent	36
3	CH_3_CN as solvent	25
4	[0.2 M]	60
**5**	**[0.4 M]**	**64 (63)**
6	[0.4 M] and K_3_PO_4_ or Na_2_CO_3_	
7	no **I**, **II**, 2,6-lutidine, or (TMS)_3_SiH	
8	no light or no light at 60 °C	

a **1** (2.50 mmol), **37** (1.50 mmol), **2** (0.5 mmol), BP **I** (20 mol %), Ni **II** (5 mol %), 2,6-lutidine (0.55 mmol), and (TMS)_3_SiH (1 mmol) at room temperature, 390 nm for 16 h.

b NMR yields using trichloroethylene as an external standard. Yield of the isolated compound is given in parentheses. BP: Benzophenone.

The reaction was run in a homemade three-dimensional (3D)-printed reactor equipped with a near-UV lamp (λ = 390 nm, see the Supporting Information for further details). Under these reaction conditions (entry 1), we observed the formation of the desired product **38** in 58% yield.

Notably, we did not find any traces of byproducts that result from the reduction of the olefin moiety by the intermediacy of a nickel hydride.^21^ Changing the solvent to acetone or acetonitrile was detrimental toward the formation of **38** (entries 2 and 3). However, by increasing the reaction concentration (entries 4 and 5), we obtained the desired product in increased yield. Notably, when using an inorganic base, the reaction did not proceed at all (entry 6). When removing the photocatalyst, Ni catalyst, 2,6-lutidine, or silane, no product was formed, highlighting their crucial role in the difunctionalization process (entry 7). Finally, the reaction is purely photocatalytic in nature as no product was detected when the protocol was run in the dark at 60 °C.

**Figure 3:**
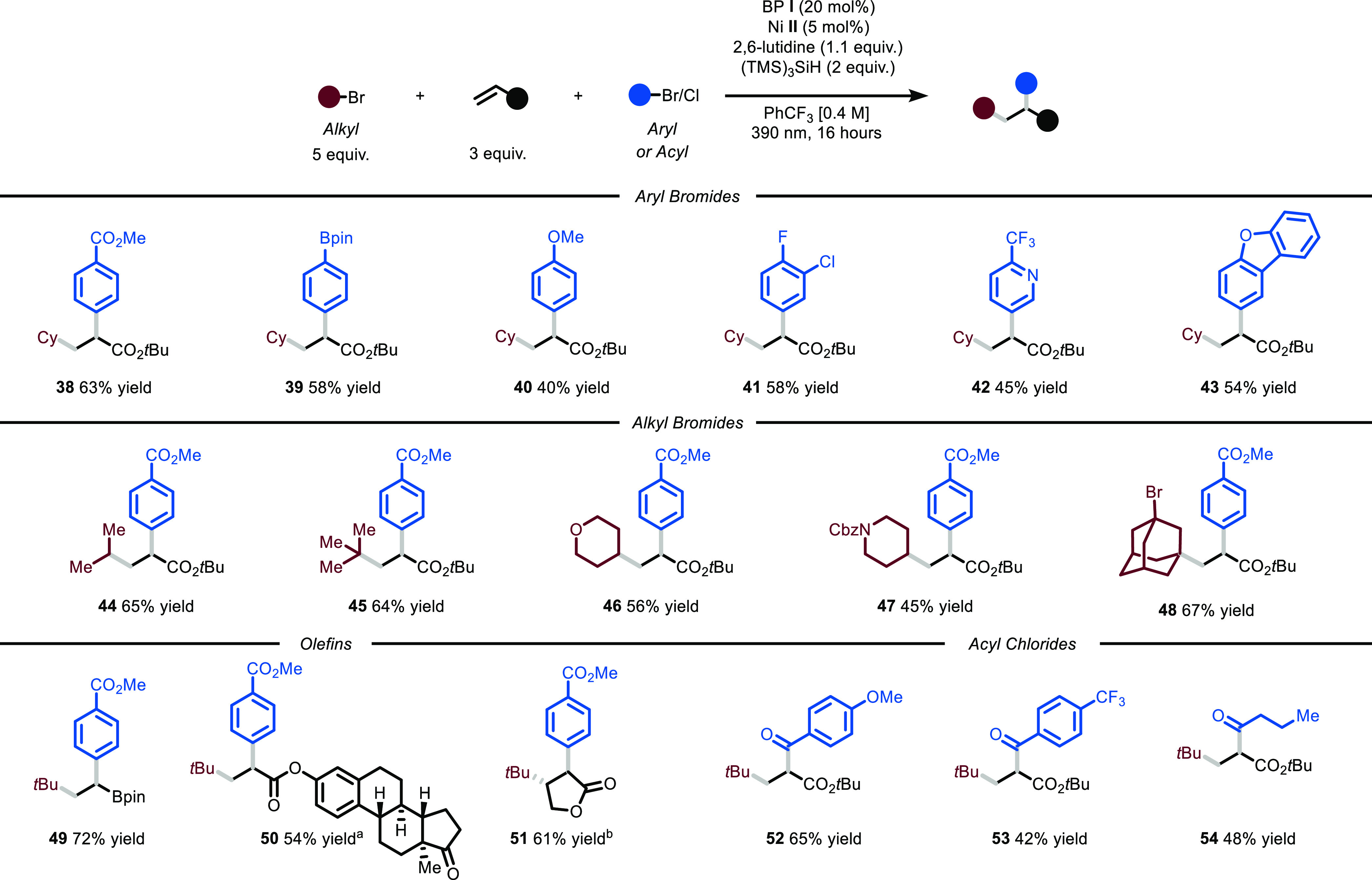
Survey of the aryl bromides, alkyl bromides, olefins, and acyl chlorides that can participate in the photochemical protocol. Reaction conditions: aryl bromide or acyl chloride (0.5 mmol), BP **I** (20 mol %), Ni **II** (5 mol %), 2,6-lutidine (1.1 equiv), (TMS)_3_SiH (2 equiv) at rt, irradiated at 390 nm for 16 h. Yields refer to isolated product. ^a^,1:1 d.r, ^b^.20:1 d.r.. BP: benzophenone, Cbz: benzyloxycarbonyl, Bpin: pinacol boronic ester.

With optimal conditions in hand, we decided to evaluate the generality of this photocatalytic difunctionalization process (Figure 3). First, we assessed the scope by varying the aryl bromide coupling partner (**38**–**43**). Not only electron-poor (**38**, 63% yield) but also electron-rich derivatives could be converted into the desired products **39** and **40** (58 and 40% yields, respectively). As shown for our cross-coupling protocol, other halides on the aromatic ring, such as fluoride and chloride, remained untouched by the nickel catalyst, leading to the selective formation of **41** (58% yield). Finally, both electron-poor and electron-rich heteroarenes could be easily installed (**42** and **43**, 45 and 54% yields).

Next, we turned our attention toward the variation of the alkyl bromides that can undergo this transformation. Acyclic derivatives such as the one leading to **44** and **45** could be directly used (65 and 64% yield). Moreover, heterocyclic substrates containing oxygen (**46**, 56% yield) and nitrogen (**47**, 45% yield) atoms could be selectively functionalized on a position distal from the heteroatom, which is challenging via HAT.^10^ Additionally, because of the mild conditions of the process, we were able to selectively functionalize 1,3-dibromoadamantane on only a single position (**48**, 67% yield). We also found that vinylboronic acid pinacol ester could be smoothly engaged in the reaction protocol (**49**, 72% yield). Importantly, the presence of the boronic group serves as the entry point for further decoration of the organic scaffold using, for example, Suzuki–Miyaura cross-coupling conditions. Moreover, an estrone-containing olefin swiftly reacted to yield the targeted difunctionalized product (**50**, 54% yield). A five-membered ring lactone was also efficiently converted to the desired product (**51**, 61% yield) with full control on diastereoselectivity. Finally, we observed that also acyl chlorides could be used as electrophiles in the nickel catalytic cycle to afford 2-substituted 1,3-dicarbonyl compounds. Importantly, not only electron-rich (**52**, 65% yield) and electron-poor (**53**, 42% yield) aroyl derivatives could be employed but also alkyl ones (**54**, 48% yield).

The results detailed in this section demonstrate the applicability of this (H)XAT platform to the difunctionalization of olefin derivatives, leading to rather complex structures starting from simple precursors.

### Mechanistic Studies

**Figure 4:**
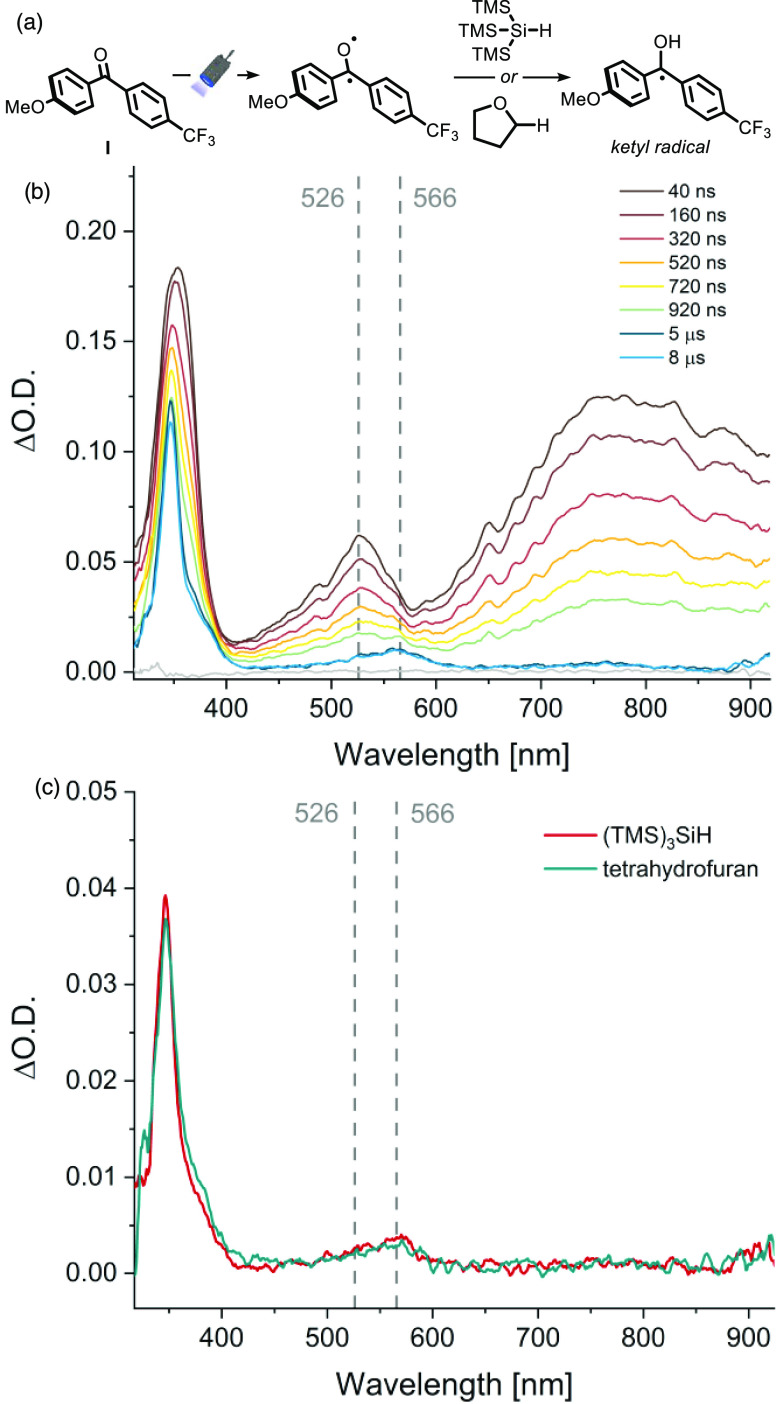
(a) Photoexcitation of benzophenone entails the hydrogen atom abstraction from the silane. (b) Microsecond triplet–triplet differential absorption spectra recorded at different times after laser excitation of the employed BP **I** in deoxygenated acetonitrile with a 5 ns laser pulse at 319 nm in the presence of an excess of (TMS)_3_SiH (8.6 equiv). (c) Comparison of microsecond triplet–triplet differential absorption spectra recorded at 50 μs after 319 nm excitation in the presence of (red dash) (TMS)_3_SiH (8.6 equiv) and (green dash) tetrahydrofuran (8.6 equiv).

As for the mechanistic scenario, we envisaged that photocatalyzed HAT would afford a nucleophilic tris(trimethylsilyl)silyl radical accountable for the subsequent XAT event from the alkyl bromide, delivering a C-centered radical. Parallelly, an in situ generated Ni^0^ species undergoes oxidative addition onto the aryl bromide to generate a relatively stable Ar–Ni^II^–Br species. In the case of the direct cross-coupling, upon interception of the C-centered radical generated via XAT, a Ni^III^ intermediate is generated, which readily undergoes reductive elimination to forge a C(sp^3^)–C(sp^2^) bond. Conversely, in the case of the three-component reaction, the alkyl radical is first intercepted by an electron-poor olefin and then the resulting radical adduct enters the Ni-catalytic cycle for the formation of the desired C(sp^3^)–C(sp^2^) bond. For both transformations, we identified three crucial steps to be investigated: (i) the HAT event (Figure 4a), (ii) the XAT step, and (iii) the radical trapping by the nickel catalytic cycle.

First, we started by observing the decay of the triplet excited state of the employed BP **I** (5 × 10^–4^ M in CH_3_CN, degassed solution) via transient absorption spectroscopy (TAS). In particular, upon excitation with a tunable Nd:YAG-laser (λ_ex_ = 319 nm, 1 mJ), a characteristic band is formed at 526 nm due to a triplet–triplet transition (see the Supporting Information), which very much resembles the one observed for the parent benzophenone.^22^ This band was monitored over a period of 50 μs and decayed completely with comparable rates in CH_3_CN and benzene, which suggests that the excited state of the diaryl ketone is not quenched by acetonitrile (see the Supporting Information).^23,24^ Next, we performed the same experiment in the presence of an excess of silane (8.6 equiv): the overall intensity of the spectrum decreased, together with the appearance of a new long-living feature with a shoulder at 375 nm and a weak, broad band at 566 nm (Figure 4b). The quenching constant for the triplet excited state of parent benzophenone by tris(trimethylsilyl)silane has been reported to be 10^8^ M^–1^ s^–1^ (Φ = 0.95).^25^ Based on comparison with the literature,^26^ we hypothesized that the species responsible for this new spectrum could be the ketyl radical generated upon HAT from the silane. It is important to stress that the latter compound does not absorb light significantly at the excitation wavelength.^25^

To ascertain the presence of the ketyl radical, we reasoned that a similar spectral feature would be observed if a different H-donor was used. Indeed, when tetrahydrofuran (8.6 equiv), a well-known quencher for diaryl ketones triplet excited states via HAT, was added as a quencher instead of tris(trimethylsilyl)silane, the very same persistent signal was observed (Figure 4c). These experiments prove that BP **I** can activate the silane via HAT to afford a highly nucleophilic tris(trimethylsilyl)silyl radical.

We also recognized the possibility that a photogenerated bromine radical might be responsible for the HAT event from the silane.^6g,27,28^ In this scenario, the employed photocatalyst would oxidize the bromide generated upon oxidative addition to afford the halogen atom.^29^ In fact, we found that the luminescence of the photocatalyst was quenched by TBABr and silane with comparable rates (*k*_SV_ = 325 and 121 M^–1^, respectively). However, since the concentration of the latter is much higher, a direct photocatalyzed HAT is more likely. To confirm our hypothesis, we compared the initial rates of the direct coupling under standard conditions and in the presence of TBABr (0.5 equiv, see the Supporting Information for further details). The reaction is slightly slower in the presence of the bromide, which led us to exclude that the latter is significantly contributing to the observed reactivity.

Additionally, we wondered whether the aforementioned HAT step was rate-determining for the transformation and set off to evaluate the kinetic isotope effect (KIE) via the parallel reactions method, accordingly (see the Supporting Information).^30^ In detail, we selected the direct coupling to study this aspect: the use of (TMS)_3_Si-D did not affect the rate of product formation, which resulted in a KIE ∼ 1. Hence, HAT is not the rate-determining step of the reaction.

We also investigated the feasibility of the XAT event triggered by the silyl radical by computational means ((U)M06-2X/6-311++G(d,p)):^31^ a Δ*G* = −16 kcal·mol^–1^ was found, suggesting an exergonic process (see the Supporting Information).

**Figure 5:**
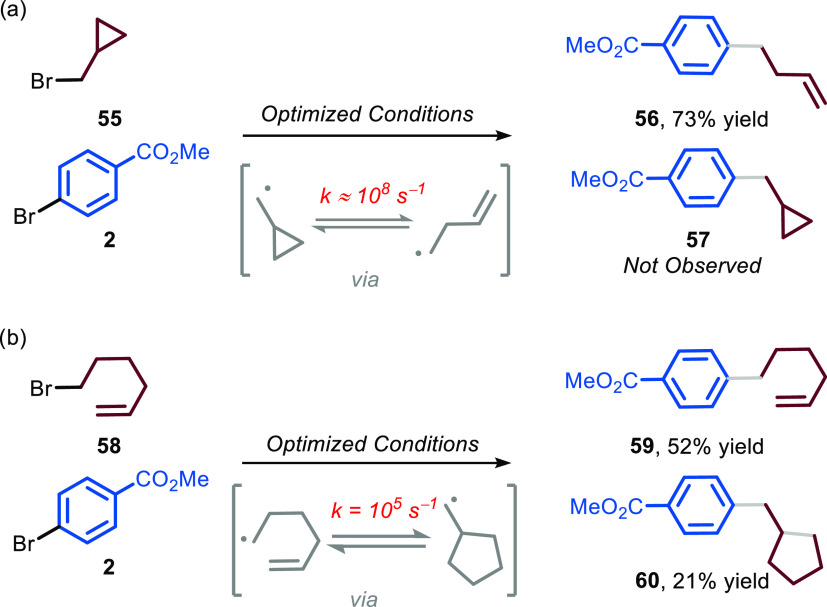
Radical clock experiments. (a) Reaction with (bromomethyl)cyclopropane **55** affords product **56**, yield refers to isolated product; (b) reaction with 6-bromo-1-hexene **58** affords a mixture of uncyclized product **59** and cyclized product **60**, yields were calculated by gas chromatography–mass spectrometry (GC–MS).

Subsequently, we decided to unequivocally assess the radical nature of the mechanism and get insights into the rate of radical addition on the nickel species by performing radical clock experiments (Figure 5). We selected (bromomethyl)cyclopropane **55** and 6-bromo-1-hexene **58** as probes and found out that, in the former case, only product **56** was formed, while in the latter case, a mixture of uncyclized (**59**) and cyclized (**60**) products was observed. Besides proving the radical nature of the mechanism, these experiments allow us to calculate a kinetic constant for the alkyl radical interception in the order of 10^7^ M^–1^ s^–1^ (see the Supporting Information).^32^

**Figure 6:**
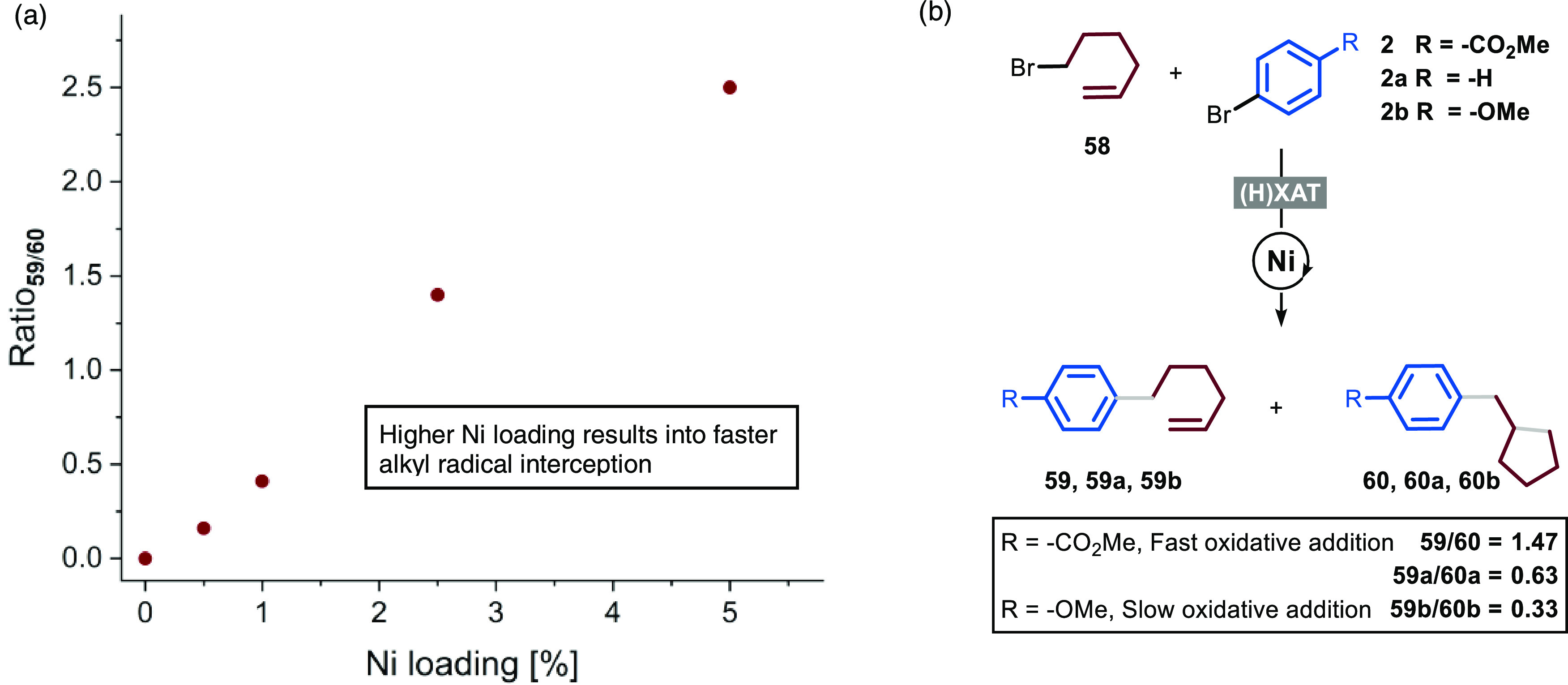
(a) Evaluation of the influence of the nickel loading on the ratio of uncyclized and cyclized products. Reactions performed in the optimized conditions (as in Table 1, entry 1), varying the loading of nickel. (b) Evaluation of the influence of the nature of the aryl bromide on the ratio of uncyclized and cyclized products. Reactions performed in the optimized conditions (as in Table 1, entry 1) with 2.5 mol % of Ni **II**.

The observation of a mixture of cyclized and uncyclized products when employing **58** led us to explore the influence of the conversion, as well as the nickel loading and the nature of the aryl bromide, on the ratio of the uncyclized versus cyclized products (Figure 6). In agreement with a previously reported protocol, we have observed no significant changes in the ratio with conversions higher than 50% (see the Supporting Information for further details).^33^ On the other hand, when performing the reaction with different nickel catalyst loadings (Figure 6a), we clearly observed a trend in the ratio: the higher the nickel loading, the higher the amount of uncyclized product. This observation is in good agreement with a situation where, in the presence of high amounts of nickel catalyst, the radical is trapped more rapidly by the latter, outpacing the cyclization step, which is instead predominant when the catalyst is present in low amounts.

Finally, we evaluated the influence of the nature of the aryl bromide (Figure 6b). We selected aryl bromides bearing different groups (−OMe, −H, −CO_2_Me), and we subjected them in parallel to the reaction conditions. We observed that the more electron-rich the aromatic ring is, the lower the ratio of the uncyclized versus cyclized product becomes. These intriguing findings suggest that, as the nature of the aryl bromide influences the product distribution, the radical is likely adding on a nickel species arising after oxidative addition, possibly a nickel(II) intermediate, as postulated by the group of Martin.^6b^

**Figure 7:**
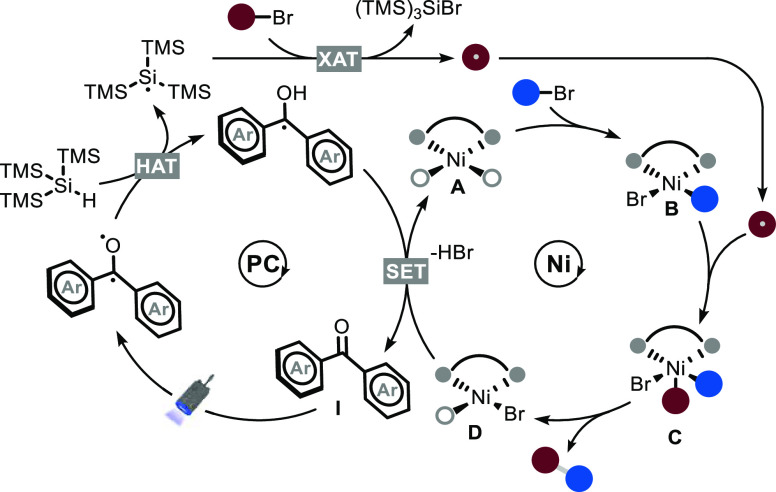
Proposed reaction mechanism for the (H)XAT cross-electrophile coupling.

Combining all of these experimental insights, we propose the following reaction mechanism for reductive cross-coupling (Figure 7). Photoexcitation of BP **I** leads to a diradical species that is responsible for the abstraction of a hydrogen atom from tris(trimethylsilyl)silane, generating a silicon-centered radical.

The latter species is capable of abstracting a bromine atom from an alkyl bromide, affording a carbon-centered radical. Simultaneously, nickel catalyst **A** is able to activate an aryl bromide through oxidative addition, affording intermediate **B** that is responsible to trap the alkyl radical, affording **C**. Reductive elimination generates the target product and Ni(I) species **D**, which engages with the reduced form of the BP **I** in a SET step to close both catalytic cycles. In agreement with such a mechanistic picture,^34^ we calculated the quantum yield for the direct coupling to be 3%, thus excluding a radical chain process. We envision that a similar mechanistic scenario is operational in the 1,2-difunctionalization. In particular, the first-formed C-centered radical is intercepted by the electron-poor olefin and the ensuing radical enters the nickel catalytic cycle.

## Conclusions

In conclusion, we have detailed the development of an operationally convenient HAT-induced XAT methodology that exploits the synergistic combination of benzophenone catalysis and a silane reagent with nickel catalysis to promote both the direct cross-electrophile coupling and the 1,2-dicarbofunctionalization of olefins. Our mechanistic studies demonstrate that the process involves a photoinduced HAT-XAT sequence leading to a carbon-centered radical that can be trapped by a nickel(II) species. Given the mild reaction conditions and the ability to engage medicinally relevant alkyl and aryl bromides, we anticipate that this methodology will be of great value to synthetic chemists in both academia and industry.

## Supporting Information Available

Experimental details, used materials, sample preparation and analytical data (NMR) (PDF)

## Notes

The primary NMR FID files for compounds **3**–**36** and **38**–**54** are available in the FigShare repository at DOI: 10.21942/uva.20237352.
