# Supplementary material for: The Merger of Benzophenone HAT Photocatalysis and Silyl Radical-Induced XAT Enables Both Nickel-Catalyzed Cross-Electrophile Coupling and 1,2-Dicarbofunctionalization of Olefins
Source: ACS Catal. 2022 Sep 1;12(18):11216–25. doi: 10.1021/acscatal.2c03805 (PMC9486949; doi:10.1021/acscatal.2c03805)

Supporting Information

The Merger of Benzophenone HAT Photocatalysis and Silyl Radical-induced XAT enables both Nickel-catalyzed Cross-Electrophile Coupling and 1,2-Dicarbofunctionalization of Olefins

Alberto Luridiana,[a]‡ Daniele Mazzarella,[a]‡ Luca Capaldo,[a] Juan A. Rincón,[b] Pablo García-Losada,[b] Carlos Mateos,[b] Michael O. Frederick,[c] Manuel Nuño,[d] Wybren Jan Buma,[e] Timothy Noël[a]*

[a] Flow Chemistry Group, Van 't Hoff Institute for Molecular Sciences (HIMS), University of Amsterdam Science Park 904, 1098 XH, Amsterdam, The Netherlands.

[b] Centro de Investigación Lilly S.A., Avda. de la Industria 30, Alcobendas-Madrid 28108, Spain.

[c] Small Molecule Design and Development, Eli Lilly and Company, Indianapolis, Indiana 46285, United States.

[d] Vapourtec Ltd. Park Farm Business Centre, Fornham St Genevieve, Bury St Edmunds, Suffolk IP28 6TS, United Kingdom.

[e] Molecular Photonics, Van 't Hoff Institute for Molecular Sciences (HIMS), University of Amsterdam, Science Park 904, 1098 XH, Amsterdam, The Netherlands.

‡ These authors contributed equally.

* Corresponding author: t.noel@uva.nl

Table of Contents

1. General Information 3
1.1 Materials 4
1.2 Reactor Design 5
2. Optimization 8
2.1 Optimization of the direct coupling reaction 8
2.2. Optimization of the 1,2-dicarbofunctionalization reaction 10
3. Experimental Procedures 12
3.1 Direct coupling reaction 12
3.2. 1,2-Dicarbofunctionalization reaction 23
4. Limitation of the Scope 29
5. Mechanistic Studies 30
5.1 Photophysical Studies 30
5.2 Computational Studies 37
5.3 Experimental Studies 42
6. References 49
7. NMR Spectra 51

1. General Information

^{1}H (300 and 400 MHz), ^{13}C (75 and 101 MHz) and ^{19}F (282 and 376 MHz) spectra were recorded at ambient temperature using Bruker AV 300-I, AV 400. ^{1}H NMR spectra are reported in parts per million (ppm) downfield relative to $CDCl_3$ (7.26 ppm) and all ^{13}C NMR spectra are reported in ppm relative to $CDCl_3$ (77.16 ppm) unless stated otherwise. The multiplicities of signals are designated by the following abbreviations: s (singlet), d (doublet), t (triplet), q (quartet), m (multiplet), dd (doublet of doublets), dt (doublet of triplets), td (triplet of doublets), ddd (doublet of doublet of doublets). Coupling constants (J) are reported in hertz (Hz). NMR data was processed using the MestReNova 14 software package. High resolution mass spectra (HRMS) were collected on an AccuTOF LC, JMS-T100LP Mass spectrometer (JEOL, Japan) or on an AccuTOF GC v 4g, JMS-T100GCV Mass spectrometer (JEOL, Japan). Disposable syringes were purchased from Laboratory Glass Specialist. Syringe pumps were purchased from Chemix Inc. model Fusion 200 Touch. Product isolation was performed manually, using silica (P60, SILICYCLE). TLC analysis was performed using Silica on aluminum foils TLC plates (F254, SILICYCLE) with visualization under ultraviolet light (254 nm and 365 nm) or appropriate TLC staining (Cerium Ammonium Molybdate). Organic solutions were concentrated under reduced pressure on a Büchi rotary evaporator (in vacuo at 40 ºC, ~5 mbar).

UV-Vis spectra were recorded with a double beam spectrophotometer Shimadzu UV2700 equipped with a deuterium lamp (190-350 nm), a halogen lamp (330-900 nm) and a photomultiplier (Hamamatsu R928). Measurements were performed in a quartz cuvette (optical path: 1 cm). All spectra were recorded in CH_3CN (solvent cutoff: 190 nm) in quartz cuvettes (optical path: 1 cm) with a bandwidth of 5 nm and a data pitch of 1 nm.

Luminescence spectra were recorded with a Horiba Fluorolog-3 equipped with a 450W Xenon lamp and a photomultiplier (Hamamatsu R636). Measurements were performed in a quartz cuvette (optical path: 1 cm) sealed with a rubber septum. The solution was bubbled with N_2 for 10 minutes before irradiation at 362 nm (A = 0.1). The slits of the excitation monochromator were kept at 10 nm, while those of the emission monochromator at 5 nm. Integration time was 0.1 s, each spectrum is an average of 2 acquisitions with a data pitch of 1 nm. A long-pass filter was used to minimize the intensity of the Raman band. The nature of the latter was verified by observing its shift by changing the excitation wavelength (data not shown).

Nanosecond transient absorptions were recorded with an in-house assembled setup. An excitation wavelength of 319 nm was used. The excitation wavelength of 319 nm was generated using a tunable Nd:YAG-laser system (NT342B, Ekspla) comprising the pump laser (NL300) with harmonics generators (SHG, THG) producing 355 nm to pump an optical parametric oscillator (OPO) with SHG connected in a single device. The laser system was operated at a repetition rate of 5 Hz with a pulse length of 5 ns. The probe light running at 10 Hz was generated by a high-stability short arc xenon flash lamp (FX-1160, Excelitas Technologies) using a modified PS302 controller (EG&G). Using a 50/50 beam splitter, the probe light was split equally into a signal beam and a reference beam and focused on the entrance slit of a spectrograph (SpectraPro-150, Princeton Instruments) with a grating of 150 ln/mm blaze at 500nm. The probe beam (A = 1 mm^2) was passed through the sample cell and orthogonally overlapped with the excitation beam on a 1 mm × 1 cm area. The excitation energy was recorded by measuring the excitation power at the back of an empty sample holder. In order to correct for fluctuations in the flash lamp spectral intensity, the reference was used to normalize the signal. Both beams were recorded simultaneously using a gated intensified CCD camera (PI-MAX3, Princeton Instruments) which has an adjustable gate of minimal 2.9 ns (20 ns were used). Two delay generators (DG535 and DG645, Stanford Research Systems, Inc.) were used to time the excitation pulse, and to change

the delay of the flash lamp and gate of the camera during the experiment. The setup was controlled by an in-house written Labview program.

1.1 Materials

All reagents and solvents were used as received without further purification. Reagents and solvents were purchased from Sigma Aldrich, TCI, abcr and Fluorochem. Technical solvents were purchased from VWR International and used as received. The benzophenone[1] and nickel[2] catalysts were prepared according to reported procedures. The majority of the substrates are commercially available. The synthesis of (3S,5S,8R,9S,10S,13S,14S)-3-bromo-10,13-dimethylhexadecahydro-17H-cyclopenta[*a*]phenanthren-17-one[3] and (8R,9S,13S,14S)-13-methyl-17-oxo-7,8,9,11,12,13,14,15,16,17-decahydro-6H-cyclopenta[*a*]phenanthren-3-yl acrylate[4] was performed according to literature procedures.

1.2 Reactor Design

UFO reactor

For the reactions performed in batch, we have used a homemade, 3D-printed reactor. The reactor was designed to fit reaction vials and to be equipped with a Kessil lamp. The reactor was designed in Adobe Inventor 2021 with 4 different parts. The lid (100mm x 12mm) is designed to host up to 8 reactions vials and holds the Kessil lamp in the center (Figure S1A). The box is designed with holes to allow the air flow to escape the reactor and keep the temperature stable (Figure S1B), as measured by an external thermometer. A reflector is situated underneath the lamp and reflects the photons inside the box to have a light distribution as homogeneous as possible (Figure S1C). A fan is glued on the bottom to provide an air flow while keeping light from escaping the system. Finally, the stirring plate adapter (Figure S1D) was added to fix the system on a stirring plate and provide homogeneous stirring (Figure S1E). It also spaces the reflector from the plate to ensure a continuous air flow from the top to the bottom of the system. All the inside surfaces were covered with reflective tape. An overview of the assembled reactor is shown in Figure S2.

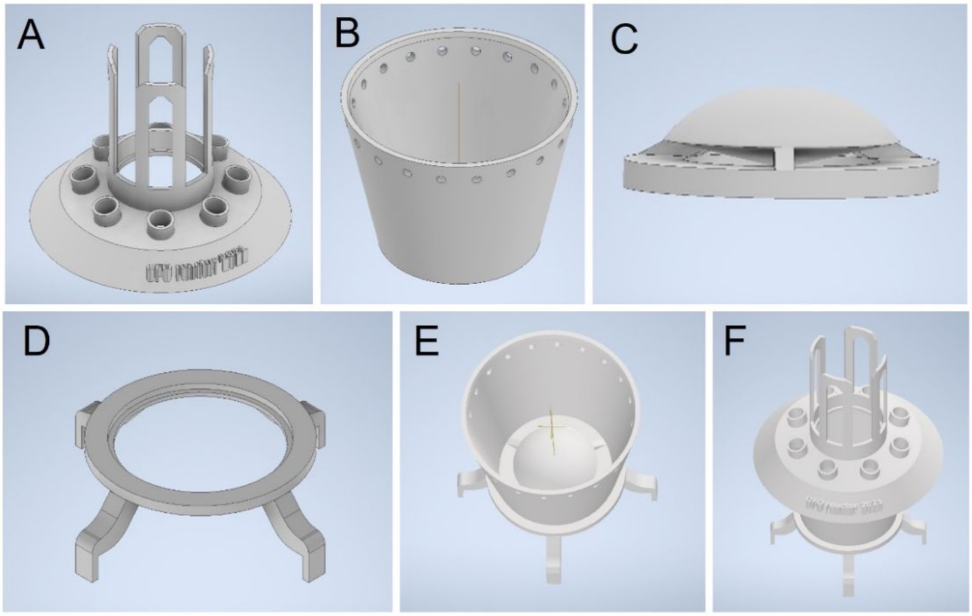

Figure S1 Overview of the 3D-printed reactor.

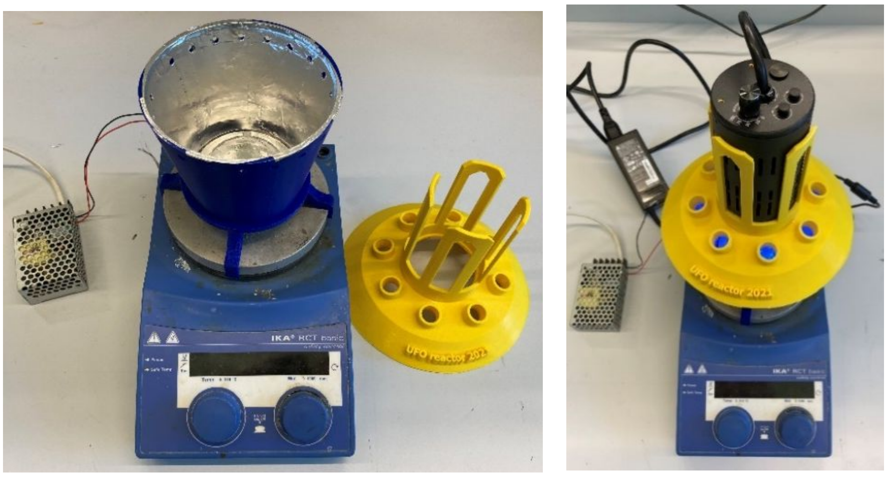

Figure S2: Pictures of the assembled reactor equipped with a Kessil lamp (365 nm).

Continuous-Flow System

For reaction performed in flow, a Vapourtec device with a UV-150 photochemical reactor was used, equipped with 16 W 365 nm LED. The temperature of the photochemical chamber was kept constant at 25 °C.

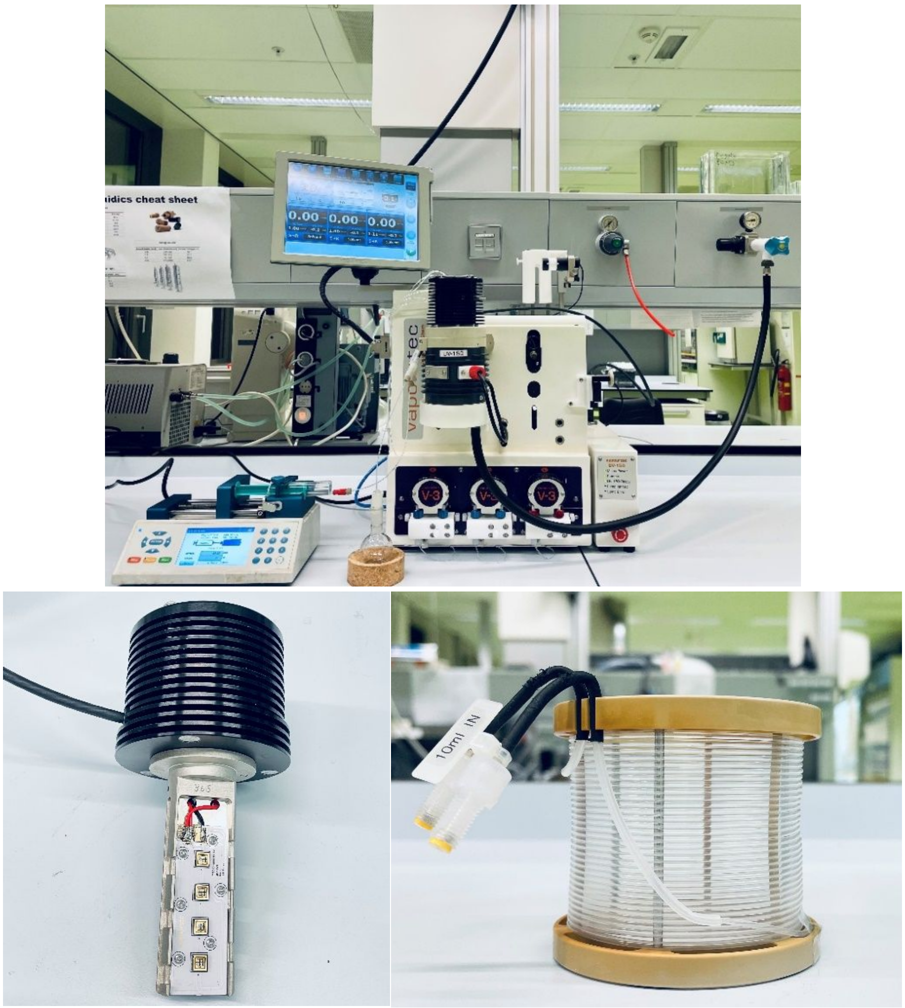

Figure S3: Overview and details of the Vapourtec system: in particular, LEDs and the PFA coil (internal diameter 1.3 mm, external diameter 1.6 mm) are shown.

For the scale-up a peristaltic pump was used with a flow rate of 0.41 mL min^{-1}. The coil of the Vapourtec device was replaced by a two layers coil (V = 25 mL).

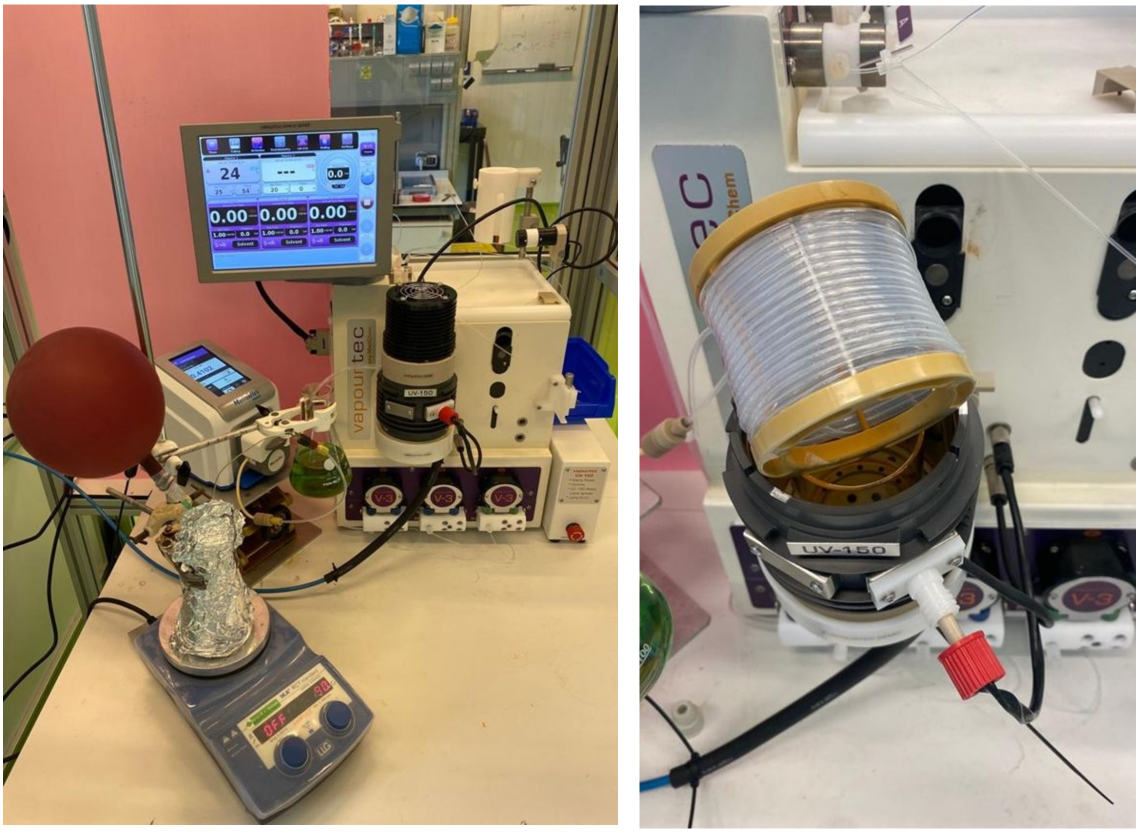

Figure S4 Overview and details of the Vapourtec system (flow setup for scale-up): in particular, PFA coil (internal diameter 2.0 mm, external diameter 3.0 mm) is shown.

2. Optimization

2.1 Optimization of the direct coupling reaction

Table S1. Screening of the HAT Photocatalyst

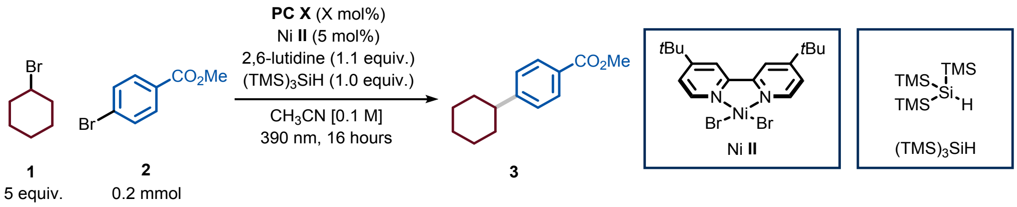

Entry	Photocatalyst(mol%)	Time	Yield
1	**PC 1** (5 %)	16	36%
2	**PC 2** (10 %)	16	36%
3	**PC 3** (10 %)	16	48%
4	**PC 4** (10 %)	16	58%
5	**PC 5** (10 %)	16	37%
6	**PC 7** (10 %)	16	25%
7	**PC 6** (10 %)	16	60%
8	**PC 6** (10 %)	48	61%

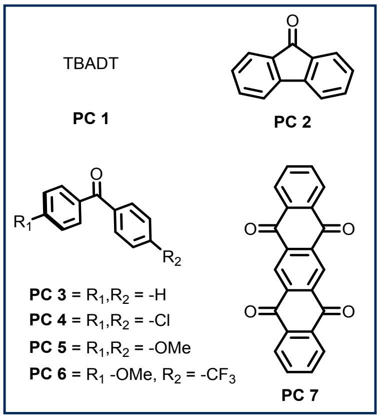

Table S2. Optimization of the Stoichiometry and Concentration

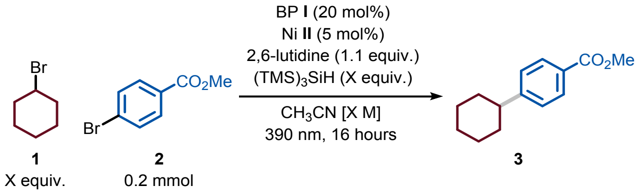

Entry	Equiv. of **1**	Equiv. of Silane	Concentration	Yield
1	5	1.0	0.1	60%
2	2.5	1.0	0.1	56%
3	2.5	1.5	0.1	75%
4	2.5	1.5	0.2	59%
5	2.5	1.5	0.05	40%

Table S3. Screening of the XAT Reagent

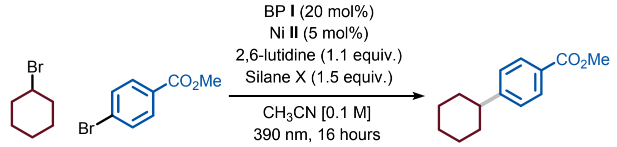

1 2.5 equiv. 2 0.2 mmol 3

Entry	Silane	Time	Yield
1	tris(trimethylsilyl)silane	16	75%
2	dimethyl(phenyl)silane	16	20%
3	triphenylsilane	16	20%
4	triisopropylsilane	16	16%
5	triethoxysilane	16	7%

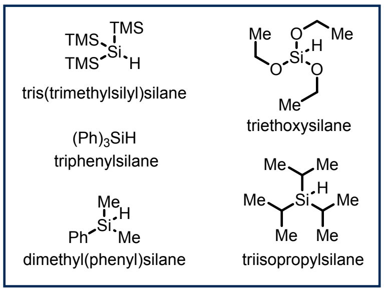

Table S4. Control experiments.

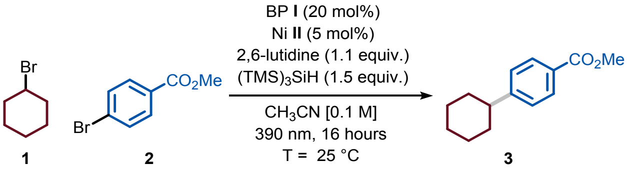

1 2.5 equiv. 2 0.2 mmol 3

Entry	Variation	Yield
1	390 nm	74
2	No light	0
3	No light 60 °C	0
4	No **I**, **II**, 2,6-lutidine or $(TMS)_3SiH$	0
5	No **I**, **II**, 2,6-lutidine or $(TMS)_3SiH$ Vapourtec 365 nm 45 min	0
6[a]	With naphthalene	16

[a] Reaction conducted with naphthalene (26 mg, 0.2 mmol 1.0 equiv.) as triplet quencher.

Table S5. Optimization of parameter for the continuous flow.

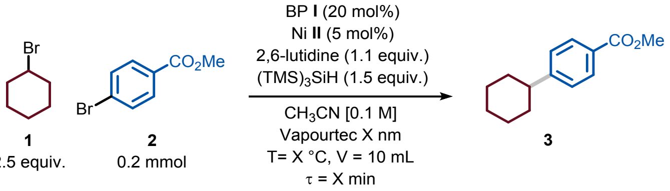

1 2.5 equiv. 2 0.2 mmol 3

Entry	Irradiation Source	Temperature	τ	Yield
1	365 nm 16 W	25	30	63
2	405 nm 16 W	25	30	35
3	365 nm 60 W	25	30	49
4	365 nm 16 W	25	45	74
5	365 nm 16 W	40	45	62

2.2. Optimization of the 1,2-dicarbofunctionalization reaction

Table S6. Optimization of the Solvent and Concentration.

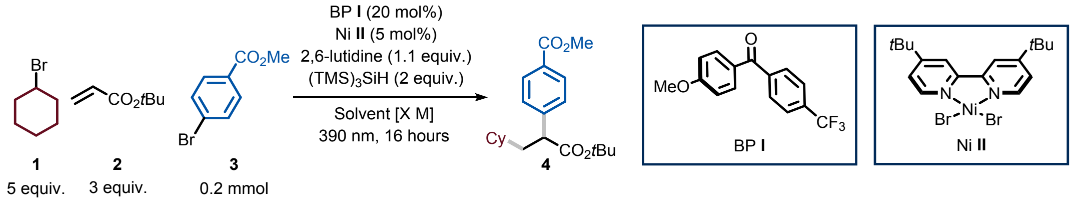

Entry	Solvent	Concentration	Yield
1	CH_3CN	0.1	25%
2	Acetone	0.1	36%
3	$PhCF_3$	0.1	58%
4	Acetone	0.2	37%
5	$PhCF_3$	0.2	60%
6	$PhCF_3$	0.4	64%

Table S7. Optimization of the Base.

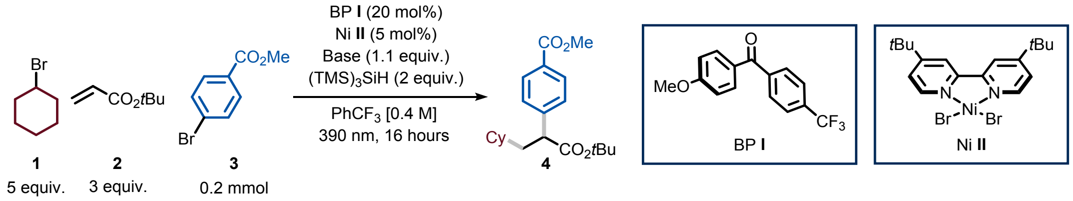

Entry	Base	Yield
1	2,6-lutidine	64%
2	Na_2CO_3	-
3	K_3PO_4	-
4	NaOAc	-

Table S8. Optimization of the Stoichiometry.

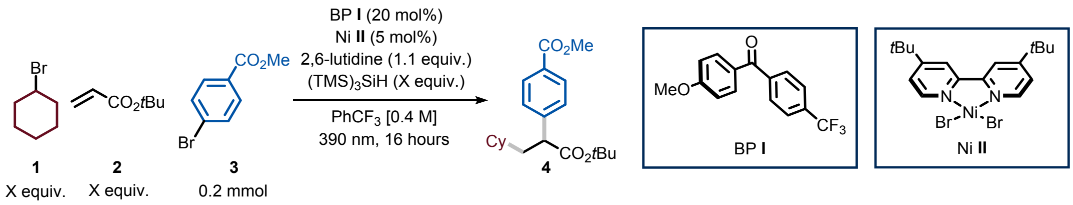

Entry	Equiv. of **1**	Equiv. of **2**	Equiv. of Silane	Yield
1	5	3	1.5	61%
2	5	2	1.5	45%
3	2.5	3	1.5	35%
4	2.5	2	1.5	30%
5	5	4	1.5	55%
6	5	3	1.1	45%
7	5	3	2	64%

Table S9. Optimization of Other Parameters.

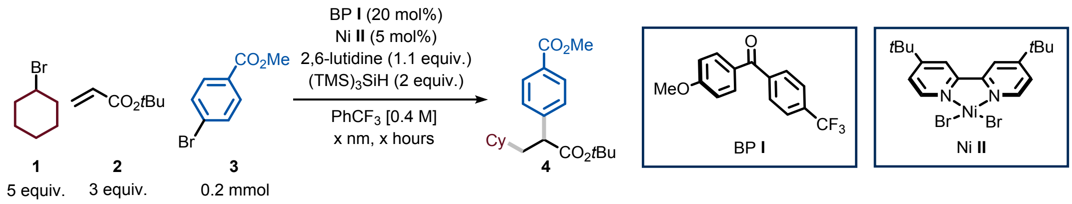

Entry	Light Wavelength	Time	Yield
1	370 nm	16 hours	49%
2	390 nm	16 hours	64%
3	390 nm	64 hours	50%

3. Experimental Procedures

3.1 Direct coupling reaction

General Procedure A

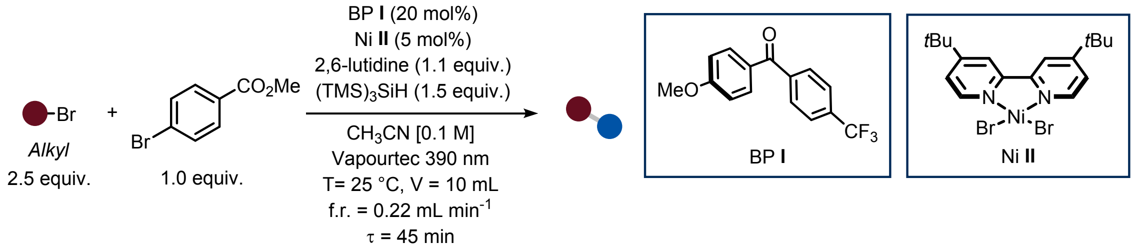

To a flame-dried argon-purged screw-capped vial, fitted with a rubber septum, charged with the nickel complex Ni **II** (12.2 mg, 5 mol%), BP **I** (28.0 mg, 20 mol%) and the aryl bromide (0.5 mmol, 1 equiv.) in CH_3CN (5 mL, 0.1 M), the alkyl bromide derivative (1.25 mmol, 2.5 equiv.), 2,6-lutidine (63 µL, 0.55 mmol, 1.1 equiv.), $(TMS)_3SiH$ (231 µL, 0.75 mmol, 1.5 equiv.) were added. Subsequently, the solution was sparged with nitrogen for 30 seconds and the reaction vessel was sealed with parafilm. This solution was taken with a 6 mL syringe (12.4 mm of diameter), positioned on a syringe pump and connected to Vapourtec UV-150. The solution was pumped, unless differently specified, with a total 0.22 mL min^{-1} flow rate and collected at the end of the reactor. The solvent of the resulting reaction mixture was removed under reduced pressure and purification by flash column chromatography on silica gel gave the corresponding products in the stated yield.

General Procedure B1

To a flame-dried argon-purged screw-capped vial, fitted with a rubber septum, charged with the nickel complex Ni **II** (12.2 mg, 5 mol%), BP **I** (28.0 mg, 20 mol%) and the aryl bromide (0.5 mmol, 1 equiv.) in $PhCF_3$ (5 mL, 0.1 M), the alkyl bromide derivative (1.25 mmol, 2.5 equiv.), 2,6-lutidine (63 µL, 0.55 mmol, 1.1 equiv.), $(TMS)_3SiH$ (231 µL, 0.75 mmol, 1.5 equiv.) were added. Subsequently, sparged with nitrogen for 30 seconds and the reaction vessel was sealed with parafilm. The vial was then placed in a 3D-printed photoreactor (see Figure S1) using a Kessil Lamp (390 nm, 100% intensity) as light source and irradiated overnight. The solvent of the resulting reaction mixture was removed under reduced pressure and purification by flash column chromatography on silica gel gave the corresponding products in the stated yield.

General Procedure B2

To a flame-dried argon-purged screw-capped vial, fitted with a rubber septum, charged with the nickel complex Ni **II** (12.2 mg, 5 mol%), BP **I** (28.0 mg, 20 mol%) and the aryl bromide (0.5 mmol, 1 equiv.) in CH_3CN (5 mL, 0.1 M), the alkyl bromide derivative (1.25 mmol, 2.5 equiv.), Na_2CO_3 (79.5 mg, 0.75 mmol, 1.5 equiv.), $(TMS)_3SiH$ (231 µL, 0.75 mmol, 1.5 equiv.) were added. Subsequently, sparged with nitrogen for 30 seconds and the reaction vessel was sealed with parafilm. The vial was then placed in a 3D-printed photoreactor (see Figure S1) using a Kessil Lamp (390 nm, 100% intensity) as light source and irradiated overnight. The solvent of the resulting reaction mixture was removed under reduced pressure and purification by flash column chromatography on silica gel gave the corresponding products in the stated yield

General procedure for scale-up

To a oven dried erlenmeyer flask with stirring bar, fitted with rubber septum, charged with the nickel complex Ni **II** (12.2 mg, 5 mol%), Benzophenone (547 mg, 30 mol%), methyl 4-bromobenzoate (10 mmol, 1 equiv.) in CH_3CN (100 mL, 0.1 M), 1-bromopropane (2.3 mL, 25.0 mmol, 2.5 equiv.), 2,6-lutidine (1.3 mL, 11.0 mmol, 1.1 equiv.), $(TMS)_3SiH$ (4.6 mL, 15.0 mmol, 1.5 equiv.) were added. Subsequently, sparged with nitrogen for 2 minutes, the solution was kept stirring in nitrogen atmosphere. By using a peristaltic pump the solution was delivered to Vapourtec UV-150 equipped with a 25 mL PFA coil prefilled with CH_3CN (internal diameter 2.0 mm, external diameter 3.0 mm) as shown in Figure S4. The solution was pumped with a total flow rate of 0.42 mL/min flow rate (60 minutes of residence time) and after 4.5 h (time to finish the collection) fresh CH_3CN was poured into the flask and used to push the last reactor volume. The solvent of the resulting reaction mixture was removed under reduced pressure and purification by flash column chromatography on silica gel (100% hexane to hexane 95:5 Et_2O, two consecutive runs) to afford product **9** (1.4 g, 80% yield) as a colourless oil.

Characterization Data of Products Direct Coupling

Methyl 4-cyclohexylbenzoate (3)

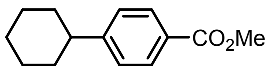

3

Prepared according to general procedure A, using cyclohexyl bromide (154 μL, 1.25 mmol, 2.5 equiv.), methyl 4-bromobenzoate (108 mg, 0.5 mmol, 1 equiv.). The crude mixture was purified by flash column chromatography (100% hexane to hexane 95:5 Et_2O, two consecutive runs) to afford product **3** (79 mg, 72% yield) as a colorless oil. The spectroscopic data are consistent with those reported previously.[5] ^1H NMR (400 MHz, $CDCl_3$) δ 7.98 (d, *J* = 8.4 Hz, 2H), 7.29 (d, *J* = 7.4 Hz, 2H), 3.92 (s, 3H), 2.58 (ddd, *J* = 11.5, 7.8, 3.1 Hz, 1H), 1.88 (td, *J* = 7.0, 4.0 Hz, 4H), 1.54 – 1.36 (m, 1H), 1.54 – 1.36 (m, 4H), 1.30 – 1.26 (m, 1H). ^{13}C NMR (101 MHz, $CDCl_3$) δ 167.3, 153.6, 129.8, 127.9, 127.0, 52.1, 44.8, 34.3, 26.9, 26.2.

Methyl 4-cyclopentylbenzoate (4)

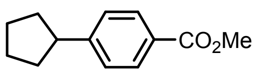

4

Prepared according to general procedure A, using cyclopentyl bromide (134 μL, 1.25 mmol, 2.5 equiv.), methyl 4-bromobenzoate (108 mg, 0.5 mmol, 1 equiv. The crude mixture was purified by flash column chromatography (100% hexane to hexane 15:1 Et_2O, two consecutive runs) to afford product **4** (56 mg, 55% yield) as a colorless oil. The spectroscopic data are consistent with those reported previously.[6]

^1H NMR (400 MHz, $CDCl_3$) δ 7.95 (d, *J* = 8.2 Hz, 2H), 7.30 (d, *J* = 8.1 Hz, 2H), 3.90 (s, 3H), 3.04 (tt, *J* = 9.5, 7.5 Hz, 1H), 2.19 – 1.99 (m, 2H), 1.89 – 1.50 (m, 6H). ^{13}C NMR (101 MHz, $CDCl_3$) δ 167.3, 152.3, 129.7, 127.8, 127.3, 52.0, 46.1, 34.6, 25.7.

Methyl 4-cyclobutylbenzoate (5)

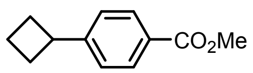

5

Prepared according to general procedure A, using cyclobutyl bromide (121 μL, 1.25 mmol, 2.5 equiv.), methyl 4-bromobenzoate (108 mg, 0.5 mmol, 1 equiv). The crude mixture was purified by flash column chromatography (100% hexane to hexane 15:1 Et_2O, two consecutive runs) to afford product **5** (76 mg, 80% yield) as a colorless oil. The spectroscopic data are consistent with those reported previously.[6]

^1H NMR (400 MHz, $CDCl_3$) δ 7.99 (d, *J* = 8.3 Hz, 2H), 7.29 (d, *J* = 8.0 Hz, 2H), 3.92 (s, 3H), 3.62 (t, *J* = 8.8 Hz, 1H), 2.39 (qd, *J* = 8.0, 3.8 Hz, 2H), 2.26 – 2.01 (m, 3H), 1.94 – 1.85 (m, 1H).

^{13}C NMR (75 MHz, $CDCl_3$) δ 167.3, 151.8, 129.7, 127.7, 126.4, 52.1, 40.4, 29.7, 18.4.

Methyl 4-cyclopropylbenzoate (6)

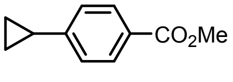

6

Prepared according to general procedure A, using bromocyclopropane (100 µL, 1.25 mmol, 2.5 equiv.), methyl 4-bromobenzoate (108 mg, 0.5 mmol, 1 equiv.). The crude mixture was purified by flash column chromatography (100% hexane to hexane 15:1 Et_2O, two consecutive runs) to afford product **6** (63 mg, 72% yield) as a colorless oil. The spectroscopic data are consistent with those reported previously.[6]

^{1}H NMR (400 MHz, $CDCl_3$) δ 7.92 (d, J = 7.8 Hz, 2H), 7.10 (d, J = 7.9 Hz, 2H), 3.89 (s, 3H), 1.94 (tt, J = 9.0, 5.1 Hz, 1H), 1.11 – 0.98 (m, 2H), 0.83 – 0.71 (m, 2H).

^{13}C NMR (75 MHz, $CDCl_3$) δ 167.3, 150.1, 129.8, 127.4, 125.5, 52.1, 15.8, 10.4.

Methyl 4-isopropylbenzoate (7)

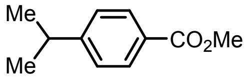

7

Prepared according to general procedure A, using 2-bromopropane (117 µL, 1.25 mmol, 2.5 equiv.), methyl 4-bromobenzoate (108 mg, 0.5 mmol, 1 equiv. The crude mixture was purified by flash column chromatography (100% hexane to hexane 15:1 Et_2O, two consecutive runs) to afford product **7** (61 mg, 69% yield) as a colorless oil. The spectroscopic data are consistent with those reported previously.[6]

^{1}H NMR (400 MHz, $CDCl_3$) δ 7.96 (d, J = 8.3 Hz, 2H), 7.29 (d, J = 8.3 Hz, 2H), 3.90 (s, 3H), 2.96 (sept, J = 6.9 Hz, 1H), 1.26 (d, J = 6.9 Hz, 6H).

^{13}C NMR (75 MHz, $CDCl_3$) δ 167.3, 154.5, 129.9, 127.9, 126.6, 52.1, 34.4, 23.9.

Methyl 4-(octan-2-yl)benzoate (8)

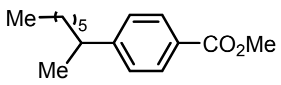

8

Prepared according to general procedure A, using 2-bromooctane (219 µL, 1.25 mmol, 2.5 equiv.), methyl 4-bromobenzoate (108 mg, 0.5 mmol, 1 equiv. The crude mixture was purified by flash column chromatography (100% hexane to hexane 15:1 Et_2O, two consecutive runs) to afford product **8** (87 mg, 70% yield) as a colorless oil. The spectroscopic data are consistent with those reported previously.[7]

^{1}H NMR (300 MHz, $CDCl_3$) δ 7.96 (d, J = 8.3 Hz, 2H), 7.24 (d, J = 8.3 Hz, 2H), 3.90 (s, 3H), 2.73 (h, J = 7.0 Hz, 1H), 1.57 (dd, J = 13.7, 6.7 Hz, 2H), 1.32 – 1.09 (m, 11H), 0.90 – 0.80 (m, 3H).

^{13}C NMR (75 MHz, $CDCl_3$) δ 167.4, 153.7, 129.8, 127.9, 127.2, 52.1, 40.2, 38.3, 31.9, 29.5, 27.7, 22.8, 22.2, 14.2.

Methyl 4-propylbenzoate (9)

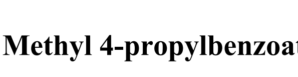

9

Prepared according to general procedure A, using 1-bromopropane (114 µL, 1.25 mmol, 2.5 equiv.), methyl 4-bromobenzoate (108 mg, 0.5 mmol, 1 equiv). The crude mixture was purified by flash column chromatography (100% hexane to hexane 15:1 Et_2O, two consecutive runs) to afford product **9** (80 mg, 90% yield) as a colorless oil. The spectroscopic data are consistent with those reported previously.[8]

^{1}H NMR (400 MHz, $CDCl_3$) δ 7.95 (d, J = 8.3 Hz, 2H), 7.24 (d, J = 8.3 Hz, 2H), 3.90 (s, 3H), 2.64 (dd, J = 8.5, 6.7 Hz, 2H), 1.75 – 1.57 (m, 2H), 0.94 (t, J = 7.3 Hz, 3H).

^{13}C NMR (75 MHz, $CDCl_3$) δ 167.4, 148.4, 129.7, 128.6, 127.8, 52.1, 38.2, 24.4, 13.9.

Methyl 4-(pent-4-en-1-yl)benzoate (10)

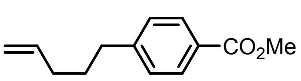

10

Prepared according to general procedure A, using 5-Bromo-1-pentene (150 µL, 1.25 mmol, 2.5 equiv.), methyl 4-bromobenzoate

(108 mg, 0.5 mmol, 1 equiv.). The crude mixture was purified by flash column chromatography (100% hexane to hexane 85:15 AcOEt) to afford product **10** (79 mg, 77% yield) as a colorless oil. This compound was previously unreported.
^{1}H NMR (400 MHz, $CDCl_3$) δ 7.99 – 7.91 (m, 2H), 7.28 – 7.21 (m, 2H), 5.82 (ddt, *J* = 16.9, 10.2, 6.6 Hz, 1H), 5.07 – 4.93 (m, 2H), 3.90 (s, 3H), 2.72 – 2.63 (m, 2H), 2.14 – 2.00 (m, 2H), 1.80 – 1.66 (m, 2H).
^{13}C NMR (101 MHz, $CDCl_3$) δ 167.3, 148.2, 138.4, 129.8, 128.6, 127.9, 115.1, 52.1, 35.4, 33.3, 30.4.
HRMS (FD) *m/z* calcd for $C_{13}H_{16}O_2$: 204.1150; found: 204.1113.

Methyl 4-methylbenzoate (11)

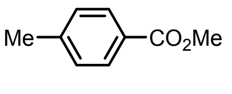

11

Prepared according to general procedure A, using methyl 4-methylbenzenesulfonate (233 mg, 1.25 mmol, 2.5 equiv.), tetrabutylammonium bromide (403 mg, 1.25 mmol, 2.5 equiv.), methyl 4-bromobenzoate (108 mg, 0.5 mmol, 1 equiv.). The crude mixture was purified by flash column chromatography (100% hexane to hexane 15:1 Et_2O, two consecutive runs) to afford product **11** (45 mg, 60% yield) as a colorless oil. The spectroscopic data are consistent with those reported previously.[9]
^{1}H NMR (300 MHz, $CDCl_3$) δ 7.93 (d, *J* = 8.4 Hz, 2H), 7.23 (d, *J* = 8.0 Hz, 2H), 3.90 (s, 3H), 2.40 (s, 3H).
^{13}C NMR (101 MHz, $CDCl_3$) δ 167.3, 143.7, 129.7, 129.2, 127.6, 52.1, 21.8.

Methyl 4-(2-methoxyethyl)benzoate (12)

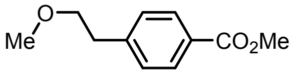

12

Prepared according to general procedure A, using 2-bromoethylmethyl ether (119 µL, 1.25 mmol, 2.5 equiv.), methyl 4-bromobenzoate (108 mg, 0.5 mmol, 1 equiv.). The crude mixture was purified by flash column chromatography (100% hexane to hexane 85:15 AcOEt) to afford product **12** (68 mg, 70% yield) as a colorless oil. The spectroscopic data are consistent with those reported previously.[10]
^{1}H NMR (300 MHz, $CDCl_3$) δ 8.01 – 7.90 (m, 2H), 7.29 (d, *J* = 8.4 Hz, 2H), 3.90 (s, 3H), 3.62 (t, *J* = 6.8 Hz, 2H), 3.34 (s, 3H), 2.93 (t, *J* = 6.8 Hz, 2H).
^{13}C NMR (75 MHz, $CDCl_3$) δ 167.2, 144.7, 129.8, 129.0, 128.3, 73.1, 58.9, 52.1, 36.4.

Methyl 4-(oxetan-3-yl)benzoate (13)

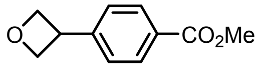

13

Prepared according to general procedure A, using 3-Bromo-oxetane (100 µL, 1.25 mmol, 2.5 equiv.), methyl 4-bromobenzoate (108 mg, 0.5 mmol, 1 equiv). The crude mixture was purified by flash column chromatography (100% hexane to hexane 80:20 AcOEt) to afford product **13** (53 mg, 55% yield) as a colorless oil. The spectroscopic data are consistent with those reported previously.[9]
^{1}H NMR (400 MHz, $CDCl_3$) δ 8.04 (d, J = 8.4 Hz, 2H), 7.47 (d, J = 8.2 Hz, 2H), 5.10 (dd, J = 8.3, 6.1 Hz, 2H), 4.77 (t, J = 6.3 Hz, 2H), 4.27 (tt, J = 8.4, 6.6 Hz, 1H), 3.92 (s, 3H).
^{13}C NMR (101 MHz, $CDCl_3$) δ 167.0, 146.9, 130.3, 129.1, 127.0, 76.8, 52.3, 40.4.

Methyl 4-(tetrahydrofuran-3-yl)benzoate (14)

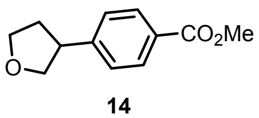

14

Prepared according to general procedure A, using 3-bromo-oxetane (100 µL, 1.25 mmol, 2.5 equiv.), methyl 4-bromobenzoate (108 mg, 0.5 mmol, 1 equiv). The crude mixture was purified by flash column chromatography (100% hexane to hexane 80:20 AcOEt) to afford product **14** (52 mg, 50% yield) as a colorless oil. The spectroscopic data are consistent with those reported previously.[11]

^{1}H NMR (400 MHz, $CDCl_3$) δ 8.10 – 7.69 (m, 2H), 7.31 (d, *J* = 8.2 Hz, 2H), 4.13 (dd, *J* = 8.6, 7.4 Hz, 1H), 4.07 (td, *J* = 8.4, 4.7 Hz, 1H), 3.97 – 3.86 (m, 4H), 3.75 (dd, *J* = 8.6, 7.0 Hz, 1H), 3.45 (p, *J* = 7.6 Hz, 1H), 2.45 – 2.26 (m, 1H), 2.00 (dq, *J* = 12.4, 7.9 Hz, 1H).
^{13}C NMR (101 MHz, $CDCl_3$) δ 167.1, 148.5, 130.1, 128.6, 127.4, 74.6, 68.6, 52.2, 45.1, 34.7.

Methyl 4-(tetrahydro-2H-pyran-4-yl)benzoate (15)

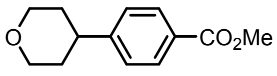

15

Prepared according to general procedure A, using 4-bromotetrahydro-2H-pyran (141 µL, 1.25 mmol, 2.5 equiv.), methyl 4-bromobenzoate (108 mg, 0.5 mmol, 1 equiv). The crude mixture was purified by flash column chromatography (100% hexane to hexane 80:20 AcOEt) to afford product **15** (84 mg, 76% yield) as a colorless oil. The spectroscopic data are consistent with those reported previously.[10]
^{1}H NMR (300 MHz, $CDCl_3$) δ 8.09 – 7.90 (m, 2H), 7.37 – 7.19 (m, 2H), 4.14 – 3.98 (m, 2H), 3.89 (s, 3H), 3.52 (td, *J* = 11.3, 3.2 Hz, 2H), 2.90 – 2.72 (m, 1H), 1.96 – 1.63 (m, 4H).
^{13}C NMR (75 MHz, $CDCl_3$) δ 167.0, 151.1, 130.0, 128.4, 126.9, 68.3, 52.1, 41.7, 33.7.

Benzyl 3-(4-(methoxycarbonyl)phenyl)azetidine-1-carboxylate (16)

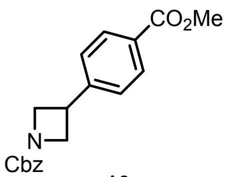

16

Prepared according to general procedure A, using 1-Cbz-3-bromoazetidine (338 mg, 1.25 mmol, 2.5 equiv.), methyl 4-bromobenzoate (108 mg, 0.5 mmol, 1 equiv). The crude mixture was purified by flash column chromatography (100% hexane to hexane 80:20 AcOEt) to afford product **16** (133 mg, 82% yield) as a colorless oil. This compound was previously unreported.
^{1}H NMR (300 MHz, $CDCl_3$): δ 8.07 – 7.97 (m, 2H), 7.42 – 7.31 (m, 7H), 5.14 (s, 2H), 4.44 (t, *J* = 8.7 Hz, 2H), 4.07 (dd, *J* = 8.7, 6.0 Hz, 2H), 3.92 (s, 3H), 3.84 (ddd, *J* = 8.7, 5.9, 2.8 Hz, 1H).
^{13}C NMR (101 MHz, $CDCl_3$) δ 166.9, 156.6, 147.2, 136.7, 130.3, 129.2, 128.7, 128.3, 128.2, 126.9, 66.9, 56.6, 52.3, 34.0.
HRMS (FD) *m/z* calcd for $C_{19}H_{19}N_1O_4$: 325.1314; found: 325.1315.

***Tert*-butyl 3-(4-(methoxycarbonyl)phenyl)pyrrolidine-1-carboxylate (17)**

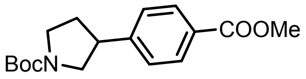

17

Prepared according to general procedure A, using 2-methyl-2-propanyl 3-bromo-1-pyrrolidinecarboxylate (313 mg, 1.25 mmol, 2.5 equiv.), methyl 4-bromobenzoate (108 mg, 0.5 mmol, 1 equiv). The crude mixture was purified by flash column chromatography (100% hexane to hexane 80:20 AcOEt) to afford product **17** (95 mg, 62% yield) as a colorless oil. The spectroscopic data are consistent with those reported previously.[11]
^{1}H NMR (300 MHz, $CDCl_3$) δ 7.98 (d, *J* = 8.0 Hz, 2H), 7.30 (d, *J* = 8.1 Hz, 2H), 3.90 (s, 3H), 3.87 – 3.72 (m, 1H), 3.68 – 3.52 (m, 1H), 3.49 – 3.26 (m, 3H), 2.39 – 2.18 (m, 1H), 2.09 – 1.88 (m, 1H), 1.47 (s, 9H).
^{13}C NMR (101 MHz, $CDCl_3$) δ 167.0, 154.6, 147.0, 130.1, 128.9, 127.2, 79.5, 52.4, 52.2, 51.7, 46.0, 45.7, 44.4, 43.5, 33.3, 32.5, 28.7.

Benzyl 4-(4-(methoxycarbonyl)phenyl)piperidine-1-carboxylate (18)

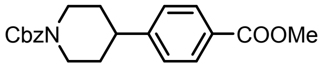

18

Prepared according to general procedure A, 4-bromo-*N*-Cbz-piperidine (373 mg, 1.25 mmol, 2.5 equiv.), methyl 4-bromobenzoate (108 mg, 0.5 mmol, 1 equiv). The crude mixture was purified by flash column chromatography (100% hexane to hexane 80:20 AcOEt) to afford product **18** (102 mg, 62% yield) as a white solid. This compound was previously unreported.
^{1}H NMR (300 MHz, $CDCl_3$) δ 7.98 (d, *J* = 8.3 Hz, 2H), 7.56 – 6.79 (m, 7H), 5.16 (s, 2H), 4.34 (d, *J* = 10.9 Hz, 2H), 3.90 (s, 3H), 2.89 (t, *J* = 12.8 Hz, 2H), 2.73 (tt, *J* = 12.1, 3.6 Hz, 1H), 1.98 – 1.50 (m, 4H).

^{13}C NMR (101 MHz, $CDCl_3$) δ 167.0, 155.4, 150.9, 136.9, 130.0, 128.60, 128.5, 128.1, 128.0, 126.9, 67.2, 52.1, 44.6, 42.8, 33.0.
HRMS (FD) *m/z* calcd for $C_{21}H_{23}N_1O_4$: 353.1627; found: 325.1622.

4-((5S,8R,9S,10S,13S,14S)-10,13-dimethyl-17-oxohexadecahydro-1H-cyclopenta[*a*]phenanthren-3-yl)phenyl acetate (19)

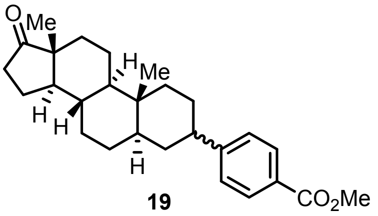

19

Prepared according to general procedure B1, using (3S,5S,8R,9S,10S,13S,14S)-3-bromo-10,13-dimethylhexadecahydro-17H-cyclopenta[*a*]phenanthren-17-one (442 mg, 1.25 mmol, 2.5 equiv.), methyl 4-bromobenzoate (108 mg, 0.5 mmol, 1 equiv). The crude mixture was purified by flash column chromatography (100% hexane to hexane 80:20 AcOEt, to afford product **19** (122 mg, 60% yield) as a white solid. This compound was previously unreported.
^{1}H NMR (400 MHz, C_6D_6) *isolated as a 1:1 mixture of diastereomers cis : trans*): δ 8.22 (d, *J* = 8.1 Hz, 4H), 7.28 (d, *J* = 8.1 Hz, 2H), 7.10 (d, *J* = 8.0 Hz, 2H), 3.55 (s, 3H), 3.52 (s, 3H), 2.85 (d, *J* = 6.4 Hz, 1H), 2.37 (ddd, *J* = 16.2, 9.6, 4.7 Hz, 1H), 2.20 – 2.06 (m, 2H), 1.93 – 1.84 (m, 3H), 1.82 – 1.70 (m, 2H), 1.67 – 1.50 (m, 6H), 1.49 – 1.35 (m, 6H), 1.33 – 1.24 (m, 4H), 1.22 – 0.89 (m, 15H), 0.85 – 0.72 (m, 3H), 0.70 (s, 3H), 0.68 (s, 3H), 0.65 (s, 3H), 0.61 (s, 3H), 0.59 – 0.45 (m, 2H), 0.30 (ddd, *J* = 11.5, 9.6, 4.1 Hz, 1H).
^{13}C NMR (75 MHz, C_6D_6) δ 218.17, 218.14, 166.84, 166.77, 152.91, 151.00, 130.23, 129.95, 128.85, 127.96, 127.28, 54.83, 54.65, 51.58, 51.50, 51.30, 47.64, 47.55, 47.50, 47.10, 45.13, 41.06, 38.91, 37.06, 36.47, 35.99, 35.70, 35.66, 35.19, 34.99, 34.55, 33.39, 32.24, 32.09, 31.13, 30.70, 29.81, 28.87, 28.82, 25.01, 21.86, 21.73, 20.60, 20.31, 13.84, 13.78, 12.49, 12.13.

Methyl 4-((8S,9S,10R,13R,14S,17R)-10,13-dimethyl-17-((R)-6-methylheptan-2-yl)-2,3,4,7,8,9,10,11,12,13,14,15,16,17-tetradecahydro-1H-cyclopenta[a]phenanthren-3-yl)benzoate (20)

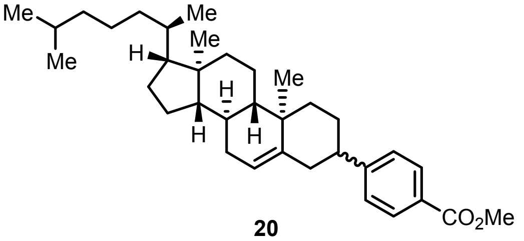

20

Prepared according to general procedure B1, using (3S,8S,9S,10R,13R,14S,17R)-3-bromo-10,13-dimethyl-17-((R)-6-methylheptan-2-yl)-2,3,4,7,8,9,10,11,12,13,14,15,16,17-tetradecahydro-1H-cyclopenta[*a*]phenanthrene (562 mg, 1.25mmol 1.25 equiv.), methyl 4-bromobenzoate (108 mg, 0.5 mmol, 1 equiv). The crude mixture was purified by flash column chromatography (100% hexane to hexane 20:1 *i*-PrOH) to afford product **20** (157 mg, 62% yield) as a white wax. This compound was previously unreported.

^{1}H NMR (400 MHz, C_6D_6) *isolated as a 1:1 mixture of diastereomers cis : trans*) δ 8.19 (d, *J* = 8.1 Hz, 4H), 7.40 (d, *J* = 8.3 Hz, 2H), 7.09 (d, *J* = 8.1 Hz, 2H), 5.45 – 5.38 (m, 2H), 3.55 (s, 3H), 3.51 (s, 3H), 2.92 – 2.84 (m, 1H), 2.72 – 2.60 (m, 1H), 2.55 – 2.33 (m, 3H), 2.22 – 1.76 (m, 15H), 1.71 – 0.84 (m, 62H), 0.74 (s, 3H), 0.69 (s, 3H).

^{13}C NMR (101 MHz, C_6D_6) *isolated as a 1:1 mixture of diastereomers cis : trans*) δ 166.9, 166.8, 152.5, 152.1, 142.5, 141.1, 130.3, 129.7, 128.9, 128.7, 128.2, 127.9, 127.2, 122.3, 120.8, 57.1, 56.9, 56.6, 51.6, 51.5, 50.9, 50.2, 46.2, 42., 42.6, 40.7, 40.29, 40.11, 40.07, 39.98, 39.95, 38.97, 37.5, 37.2, 36.7, 36.7, 36.3, 35.7, 33.4, 32.4, 32.3, 32.2, 32.1, 30.2, 28.7, 28.5, 28.46, 28.4, 24.7, 24.6, 24.4, 23.1, 23.1, 22.8, 21.4, 21.1, 19.9, 19.8, 19.1, 19.1, 12.2, 12.2.

The product **20**, isolated as a 1:1 mixture of diastereomers cis : trans, was separated by preparative HPLC (100% hexane to hexane 20:1 AcOEt,) to afford the corresponding Diastereomer **20a** and Diastereomer **20b**.

Diastereomer 20a

^{1}H NMR (400 MHz, C_6D_6) δ 8.20 (d, *J* = 8.3 Hz, 2H), 7.09 (d, *J* = 8.3 Hz, 2H), 5.61 – 5.20 (m, 1H), 3.66 – 3.38 (m, 3H), 2.56 – 2.33 (m, 2H), 2.25 – 1.95 (m, 3H), 1.94 – 1.75 (m, 2H), 1.69 – 1.41 (m, 12H), 1.36 – 1.09 (m, 10H), 1.08 – 1.02 (m, 6H), 0.96 (s, 3H), 0.94 (s, 3H), 0.74 (s, 3H).
^{13}C NMR (75 MHz, C_6D_6) δ 166.8, 152.5, 142.5, 130.3, 128.9, 127.2, 120.8, 57.1, 56.6, 51.6, 50.9, 46.2, 42.7, 40.7, 40.3, 40.1, 40.9, 37.2, 36.7, 36.3, 32.4, 32.3, 30.2, 28.7, 28.5, 24.7, 24.4, 23.1, 22.8, 21.4, 19.8, 19.1, 12.2.
HRMS (FD) *m/z* calcd for $C_{35}H_{52}O_2$: 504.3967; found: 504.3980.

Diastereomer 20b

^{1}H NMR (300 MHz, C_6D_6) δ 8.20 (d, *J* = 8.3 Hz, 2H), 7.40 (d, *J* = 8.3 Hz, 2H), 5.40 (s, 1H), 3.50 (s, 3H), 2.87 (s, 1H), 2.64 (d, *J* = 15.0 Hz, 1H), 2.42 – 1.78 (m, 8H), 1.71 – 0.87 (m, 31H), 0.69 (s, 3H).
^{13}C NMR (101 MHz, C_6D_6) δ 166.8, 152.1, 141.09, 129.7, 128.7, 122.3, 56.9, 56.6, 50.2, 46.2, 42.6, 40.7, 40.29, 40.1, 39.98, 39.95, 39.0, 37.5, 37.2, 36.7, 36.3, 35.7, 33.4, 32.2, 32.1, 30.2, 24.6, 23.1, 22.8, 21.1, 19.9, 19.1, 12.2.
HRMS (FD) *m/z* calcd for $C_{35}H_{52}O_2$: 504.3967; found: 504.3989.

Methyl 4-(3-(4,4,5,5-tetramethyl-1,3,2-dioxaborolan-2-yl)propyl)benzoate (21)

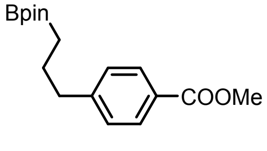

21

Prepared according to general procedure A, 3-bromopropylboronic acid pinacol ester (264 μL,1.25 mmol, 2.5 equiv.) and methyl 4-bromobenzoate (108 mg, 0.5 mmol, 1 equiv). The crude mixture was purified by flash column chromatography (100% hexane to hexane 90:10 AcOEt) to afford product **21** (114 mg, 75% yield) as a colorless oil. The spectroscopic data are consistent with those reported previously.[12]
^{1}H NMR (300 MHz, $CDCl_3$) δ 7.93 (d, *J* = 8.3 Hz, 2H), 7.23 (d, *J* = 8.2 Hz, 2H), 3.89 (s, 3H), 2.65 (dd, *J* = 8.7, 6.7 Hz, 2H), 1.74 (ddd, *J* = 15.6, 8.4, 7.0 Hz, 2H), 1.24 (s, 12H), 0.81 (t, *J* = 7.9 Hz, 2H).
^{13}C NMR (101 MHz, $CDCl_3$) δ 167.4, 148.4, 129.7, 128.7, 127.7, 83.2, 52.1, 38.7, 25.9, 25.1. The signal of the α-B-carbon was not observed.

2-(3-(3,5-bis(trifluoromethyl)phenyl)propyl)-4,4,5,5-tetramethyl-1,3,2-dioxaborolane (22)

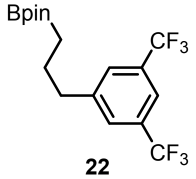

22

Prepared according to general procedure A, 3-bromopropylboronic acid pinacol ester (264 μL,1.25 mmol, 2.5 equiv.) and 3,5-Bis-trifluoromethylbromobenzene (147 mg, 0.5 mmol, 1 equiv.) The crude mixture was purified by flash column chromatography (100% hexane to hexane 90:10 AcOEt) to afford product **22** (115 mg, 72% yield) as a colorless oil. This compound was previously unreported.
^{1}H NMR (400 MHz, $CDCl_3$) δ 7.69 (s, 1H), 7.62 (s, 2H), 2.78 – 2.70 (m, 2H), 1.77 (p, *J* = 7.8 Hz, 2H), 1.25 (s, 12H), 0.83 (t, *J* = 7.9 Hz, 2H).
^{13}C NMR (101 MHz, $CDCl_3$) δ 145.14, δ 131.5 (q, *J* = 32.9 Hz), 125.0, 122.3, 119.9 (dt, *J* = 7.7, 3.8 Hz), 83.3, 38.2, 25.8, 25.0. The signal of the α-B-carbon was not observed.
^{19}F NMR (282 MHz, $CDCl_3$) δ -62.82.
HRMS (FD) *m/z* calcd for $C_{19}H_{19}N_1O_4$: 383.1620; found: 383.2056.

2-(3-(3-chloro-4-fluorophenyl)propyl)-4,4,5,5-tetramethyl-1,3,2-dioxaborolane (23)

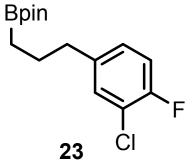

23

Prepared according to general procedure A, 3-bromopropylboronic acid pinacol ester (264 µL,1.25 mmol, 2.5 equiv.) and 4-bromo-2-chloro-fluorobenzene (105 mg, 0.5 mmol, 1 equiv.). The crude mixture was purified by flash column chromatography (100% hexane to hexane 90:10 AcOEt) to afford product **23** (100 mg, 61% yield) as a colorless oil. This compound was previously unreported.

^{1}H NMR (400 MHz, $CDCl_3$) δ 7.22 – 7.16 (m, 1H), 7.00 (dt, J = 6.2, 1.4 Hz, 2H), 2.55 (t, J = 7.7 Hz, 2H), 1.75 – 1.63 (m, 2H), 1.24 (s, 12H), 0.79 (t, J = 7.9 Hz, 2H). The signal of the α-B-carbon was not observed.

^{13}C NMR (101 MHz, $CDCl_3$) δ 156.5 (d, *J* = 245.8 Hz), 139.7 (d, *J* = 3.8 Hz), 130.6, 128.2 (d, *J* = 6.8 Hz), 120.4 (d, *J* = 17.5 Hz), 116.2 (d, *J* = 20.7 Hz), 83.2, 37.6, 26.0, 25.0.

^{19}F NMR (376 MHz, $CDCl_3$) δ -118.51 – -124.98 (m).

HRMS (FD) *m/z* calcd for $C_{19}H_{19}N_1O_4$: 298.1310; found: 298.1302.

2-(3-(4,4,5,5-tetramethyl-1,3,2-dioxaborolan-2-yl)propyl)benzonitrile (24)

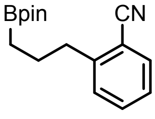

24

Prepared according to general procedure A , 3-bromopropylboronic acid pinacol ester (264 µL,1.25 mmol, 2.5 equiv.) and methyl 2-bromobenzonitrile (91 mg, 0.5 mmol, 1 equiv.). The crude mixture was purified by flash column chromatography (100% hexane to hexane 90:10 AcOEt) to afford product **24** (115 mg, 70% yield) as a colorless oil. This compound was previously unreported.

^{1}H NMR (400 MHz, $CDCl_3$) δ 7.58 (dd, *J* = 7.8, 1.4 Hz, 1H), 7.48 (td, *J* = 7.7, 1.4 Hz, 1H), 7.31 (d, *J* = 7.8 Hz, 1H), 7.25 (td, *J* = 7.6, 1.2 Hz, 1H), 2.88 – 2.80 (m, 2H), 1.79 (p, *J* = 7.9 Hz, 2H), 1.24 (s, 12H), 0.85 (t, *J* = 7.9 Hz, 2H).

^{13}C NMR (101 MHz, $CDCl_3$) δ 146.7, 132.9, 132.7, 129.8, 126.4, 118.3, 112.5, 83.2, 37.0, 25.6, 25.0. The signal of the α-B-carbon was not observed.

HRMS (FD) *m/z* calcd for $C_{16}H_{22}B_1N_1O_2$: 272.1825 found: 272.1819.

4,4,5,5-tetramethyl-2-(3-(4-(4,4,5,5-tetramethyl-1,3,2-dioxaborolan-2-yl)phenyl)propyl)-1,3,2-dioxaborolane (25)

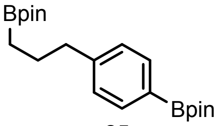

25

Prepared according to general procedure A, 3-bromopropylboronic acid pinacol ester (264 µL, 1.25 mmol, 2.5 equiv.) and 4-bromobenzeneboronic acid pinacol ester (141 mg, 0.5 mmol, 1 equiv.). The crude mixture was purified by flash column chromatography (100% hexane to hexane 90:10 AcOEt) to afford product **25** (128 mg, 69% yield) as a colorless oil. The spectroscopic data are consistent with those reported previously.[13]

^{1}H NMR (300 MHz, $CDCl_3$) δ 7.78 – 7.69 (m, 2H), 7.25 – 7.17 (m, 2H), 2.70 – 2.58 (m, 2H), 1.75 (tt, *J* = 9.4, 6.9 Hz, 2H), 1.36 (s, 12H), 1.26 (s, 12H), 0.87 – 0.80 (m, 2H).

^{13}C NMR (101 MHz, $CDCl_3$) δ 146.3, 134.9, 128.2, 83.7, 83.1, 82.9, 77.4, 38.9, 32.4, 26.1, 25.0.

2-(3-(4-methoxyphenyl)propyl)-4,4,5,5-tetramethyl-1,3,2-dioxaborolane (26)

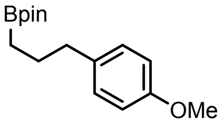

26

Prepared according to general procedure B2, 3-bromopropylboronic acid pinacol ester (264 µL, 1.25 mmol, 2.5 equiv.) and methyl 4-methoxybenzoate (83 mg, 0.5 mmol, 1 equiv.) The crude mixture was purified by flash column chromatography (100% hexane to hexane 90:10 AcOEt) to afford product **26** (83 mg, 60% yield) as a colorless oil. The spectroscopic data are consistent with those reported previously.[14]

^{1}H NMR (400 MHz, $CDCl_3$) δ 7.09 (d, *J* = 8.2 Hz, 2H), 6.81 (dd, *J* = 8.4, 1.3 Hz, 2H), 3.78 (s, 3H), 2.55 (t, *J* = 7.7 Hz, 2H), 1.75 – 1.63 (m, 2H), 1.24 (d, *J* = 1.2 Hz, 12H), 0.81 (t, *J* = 7.9 Hz, 2H).

^{13}C NMR (101 MHz, $CDCl_3$) δ 157.7, 135.0, 129.5, 113.73 83.1, 55.4, 37.8, 26.5, 25.0.

5-(3-(4,4,5,5-tetramethyl-1,3,2-dioxaborolan-2-yl)propyl)pyrimidine-2-carbonitrile (27)

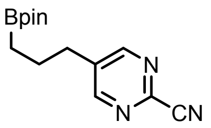

27

Prepared according to general procedure B2, 3-bromopropylboronic acid pinacol ester (264 μL, 1.25 mmol, 2.5 equiv.) and methyl 5-bromopyrimidine-2-carbonitrile (92 mg, 0.5 mmol, 1 equiv.). The crude mixture was purified by flash column chromatography (100% hexane to hexane 90:10 AcOEt) to afford product **27** (72.4 mg, 53% yield) as a colorless oil. This compound was previously unreported.

^{1}H NMR (400 MHz, $CDCl_3$) δ 8.65 (s, 2H), 2.69 (t, *J* = 7.7 Hz, 2H), 1.77 (p, *J* = 7.8 Hz, 2H), 1.24 (s, 12H), 0.84 (t, *J* = 7.7 Hz, 2H).

^{13}C NMR (101 MHz, $CDCl_3$) δ 158.0, 143.1, 138.7, 165.0, 83.5, 32.9, 25.2, 25.0. The signal of the α-B-carbon was not observed.

HRMS (FD) *m/z* calcd for $C_{14}H_{20}B_1N_3O_2$: 274.1729; found: 274.1729.

5-(3-(4,4,5,5-tetramethyl-1,3,2-dioxaborolan-2-yl)propyl)-2-(trifluoromethyl)pyridine (28)

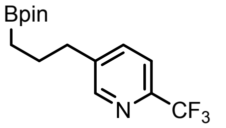

28

Prepared according to general procedure A, 3-bromopropylboronic acid pinacol ester (264 μL, 1.25 mmol, 2.5 equiv.) and 5-bromo-2-(trifluoromethyl)pyridine (113 mg, 0.5 mmol, 1 equiv.). The crude mixture was purified by flash column chromatography (100% hexane to hexane 90:10 AcOEt) to afford product **28** (120 mg, 76% yield) as a colorless oil. This compound was previously unreported.

^{1}H NMR (400 MHz, $CDCl_3$) δ 8.56 – 8.51 (m, 1H), 7.65 (dd, *J* = 8.0, 2.1 Hz, 1H), 7.57 (d, *J* = 8.0 Hz, 1H), 2.68 (t, *J* = 7.7 Hz, 2H), 1.75 (p, *J* = 7.6 Hz, 2H), 1.23 (s, 12H), 0.82 (t, *J* = 7.8 Hz, 2H).

^{13}C NMR (101 MHz, $CDCl_3$) δ 150.44, 145.91 (d, *J* = 34.6 Hz), 141.38, 137.18, 121.89 (q, *J* = 273.6 Hz), 120.16 (q, *J* = 2.8 Hz), 83.3, 35.4, 25.7, 25.0. The signal of the α-B-carbon was not observed.

^{19}F NMR (282 MHz, $CDCl_3$) δ -67.68 – -67.70 (m).

HRMS (FD) *m/z* calcd for $C_{15}H_{21}B_1F_3N_1O_2$: 316.1698; found: 316.1695.

2-(3-(benzo[b]thiophen-5-yl)propyl)-4,4,5,5-tetramethyl-1,3,2-dioxaborolane (29)

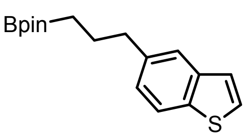

29

Prepared according to general procedure A, 3-bromopropylboronic acid pinacol ester (264 μL, 1.25 mmol, 2.5 equiv.) and 5-bromobenzo[*b*]thiophene (107 mg, 0.5 mmol, 1 equiv.). The crude mixture was purified by flash column chromatography (100% hexane to hexane 90:10 AcOEt) to afford product **29** (98 mg, 60% yield) as a colorless oil. This compound was previously unreported.

^{1}H NMR (400 MHz, $CDCl_3$) δ 7.76 (d, *J* = 8.2 Hz, 1H), 7.62 (s, 1H), 7.39 (d, *J* = 5.4 Hz, 1H), 7.28 – 7.25 (m, 1H), 7.18 (d, *J* = 8.2 Hz, 1H), 2.73 (t, *J* = 7.7 Hz, 2H), 1.78 (p, *J* = 7.7 Hz, 2H), 1.24 (d, *J* = 1.3 Hz, 12H), 0.85 (s, 2H).

^{13}C NMR (101 MHz, $CDCl_3$) δ 139.9, 138.8, 137.1, 126.3, 125.6, 123.7, 123.2, 122.1, 83.0, 38.5, 26.4, 24.9.

HRMS (FD) *m/z* calcd for $C_{17}H_{23}B_1O_2S_1$: 302.1515; found: 302.1516.

2-(3-(dibenzo[b,d]furan-2-yl)propyl)-4,4,5,5-tetramethyl-1,3,2-dioxaborolane (30)

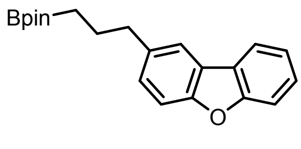

30

Prepared according to general procedure A, 3-bromopropylboronic acid pinacol ester (264 μL, 1.25 mmol, 2.5 equiv.) and using 2-bromodibenzofuran (124 mg, 0.5 mmol, 1 equiv.) The crude mixture was purified by flash column chromatography (100% hexane to hexane 90:10 AcOEt) to afford product **30** (104 mg, 62% yield) as a white solid. This compound was previously unreported.

^{1}H NMR (400 MHz, $CDCl_3$) δ 7.97 – 7.89 (m, 2H), 7.23 (d, *J* = 8.3 Hz, 2H), 3.89 (s, 3H), 2.70 – 2.61 (m, 2H), 1.80 – 1.68 (m, 2H), 1.24 (s, 12H), 0.86 – 0.77 (m, 2H).

^{13}C NMR (101 MHz, $CDCl_3$) δ 156.6, 154.8, 137.4, 128.1, 127.0, 124.5, 124.2, 122.6, 120.7, 120.3, 111.7, 111.2, 83.1, 38.6, 26.8, 25.0. The signal of the α-B-carbon was not observed.

HRMS (FD) *m/z* calcd for $C_{21}H_{25}B_1O_3$: 336.1901; found: 336.1912.

1-methyl-5-(3-(4,4,5,5-tetramethyl-1,3,2-dioxaborolan-2-yl)propyl)-1H-indole (31)

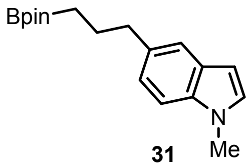

31

Prepared according to general procedure A , 3-bromopropylboronic acid pinacol ester (264 µL, 1.25 mmol, 2.5 equiv.) and 5-bromo-1-methyl-1H-indole (105 mg, 0.5 mmol, 1 equiv.). The crude mixture was purified by flash column chromatography (100% hexane to hexane 90:10 AcOEt, two consecutive runs) to afford product **31** (84 mg, 56% yield) as a pale yellow oil. This compound was previously unreported.

^{1}H NMR (400 MHz, $CDCl_3$) δ 7.41 (s, 1H), 7.22 (d, *J* = 8.3 Hz, 1H), 7.06 (dd, *J* = 8.4, 1.6 Hz, 1H), 7.00 (d, *J* = 3.0 Hz, 1H), 6.40 (d, *J* = 3.1 Hz, 1H), 3.76 (s, 3H), 2.74 – 2.67 (m, 2H), 1.77 (p, *J* = 7.8 Hz, 2H), 1.24 (s, 12H), 0.85 (t, *J* = 8.0 Hz, 2H).

^{13}C NMR (101 MHz, $CDCl_3$) δ 135.5, 133.7, 128.9, 128.7, 122.9, 120.3, 108.9, 100.6, 83.0, 38.85, 33.0, 27.1, 25.0. The signal of the α-B-carbon was not observed.

HRMS (FD) *m/z* calcd for $C_{18}H_{26}B_1N_1O_2$: 300.2138; found: 300.2149.

4-(3-(4,4,5,5-tetramethyl-1,3,2-dioxaborolan-2-yl)propyl)thiazole (32)

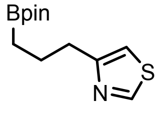

32

Prepared according to general procedure A, 3-bromopropylboronic acid pinacol ester (264 µL, 1.25 mmol, 2.5 equiv.) and 4-bromothiazole (82 mg, 0.5 mmol, 1 equiv.) The crude mixture was purified by flash column chromatography (100% hexane to hexane 85:15 AcOEt) to afford product **32** (42 mg, 33% yield) as a colourless oil. This compound was previously unreported.

^{1}H NMR (400 MHz, $CDCl_3$) δ 8.72 (d, *J* = 2.0 Hz, 1H), 6.93 (dt, *J* = 1.9, 0.9 Hz, 1H), 2.88 – 2.80 (m, 2H), 1.84 (p, *J* = 7.7 Hz, 2H), 1.24 (s, 12H), 0.84 (t, *J* = 7.9 Hz, 2H).

^{13}C NMR (101 MHz, $CDCl_3$) δ 158.5, 152.2, 112.8, 83.1, 33.9, 24.8, 23.9. The signal of the α-B-carbon was not observed.

7-(3-(4,4,5,5-tetramethyl-1,3,2-dioxaborolan-2-yl)propyl)imidazo[1,2-*a*]pyridine (33)

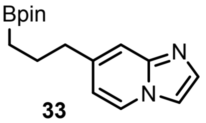

33

Prepared according to general procedure B2, 3-bromopropylboronic acid pinacol ester (264 µL, 1.25 mmol, 2.5 equiv.) and using 7-bromoimidazo[1,2-*a*]pyridine (98.5 mg, 0.5 mmol, 1 equiv.) The crude mixture was purified by flash column chromatography (100% hexane to hexane 85:15 AcOEt) to afford product **33** (46 mg, 32% yield) as pale brown oil. This compound was previously unreported.

^{1}H NMR (400 MHz, $CDCl_3$) δ 8.26 (s, 1H), 7.87 (d, *J* = 2.3 Hz, 1H), 7.43 (d, *J* = 9.0 Hz, 1H), 6.97 (dt, *J* = 9.1, 1.0 Hz, 1H), 6.43 (d, *J* = 2.2 Hz, 1H), 2.60 (t, *J* = 7.6 Hz, 2H), 1.76 (t, *J* = 7.7 Hz, 2H), 1.23 (d, *J* = 0.8 Hz, 12H), 0.84 (t, *J* = 7.8 Hz, 2H). ^{13}C NMR (101 MHz, $CDCl_3$) δ 141.4, 139.0, 126.6, 126.3, 125.9, 117.5, 96.3, 83.2, 35.1, 25.4, 25.0. The signal of the α-B-carbon was not observed.

HRMS (FD) *m/z* calcd for $C_{16}H_{23}B_1N_2O_2$: 287.1934; found: 287.1929.

Ethyl 7-(3-(4,4,5,5-tetramethyl-1,3,2-dioxaborolan-2-yl)propyl)imidazo[1,2-*a*]pyridine-2-carboxylate (34)

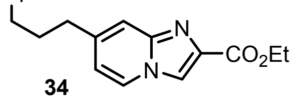

34

Prepared according to general procedure B2, 3-bromopropylboronic acid pinacol ester (264 µL, 1.25 mmol, 2.5 equiv.) and methyl 7-bromoimidazo[1,2-*a*]pyridine-2-carboxylate (128 mg, 0.5 mmol, 1 equiv.) The crude mixture was purified by flash column chromatography (100% hexane to hexane 85:15 AcOEt) to afford product **34** (126 mg, 73% yield) as a white solid. This compound was previously unreported.

^{1}H NMR (300 MHz, $CDCl_3$) δ 8.11 – 8.03 (m, 1H), 7.98 (dd, *J* = 7.0, 1.0 Hz, 1H), 7.38 (s, 1H), 6.69 (dd, *J* = 7.1, 1.6 Hz, 1H), 4.42 (q, *J* = 7.1 Hz, 2H), 2.62 (t, *J* = 7.6 Hz, 2H), 1.73 (p, *J* = 7.7 Hz, 2H), 1.40 (t, *J* = 7.1 Hz, 3H), 1.21 (s, 12H), 0.80 (t, *J* = 7.8 Hz, 2H).

^{13}C NMR (75 MHz, $CDCl_3$) δ 163.6, 145. 9, 141.8, 136.8, 125.4, 116.7, 116.5, 116.1, 83.2, 61.1, 38.0, 24.9, 25.9, 14.5. The signal of the α-B-carbon was not observed.

HRMS (FD) *m/z* calcd for $C_{19}H_{27}N_2O_4$: 359.2146; found: 359.2143.

5-(3-(4,4,5,5-tetramethyl-1,3,2-dioxaborolan-2-yl)propyl)pyrazolo[1,5-*a*]pyridine (35)

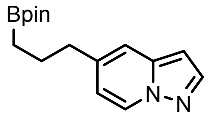

35

Prepared according to general procedure B2, 3-bromopropylboronic acid pinacol ester (264 µL, 1.25 mmol, 2.5 equiv.) and using 5-bromopyrazolo[1,5-*a*]pyridine (98.5 mg, 0.5 mmol, 1 equiv.). The crude mixture was purified by flash column chromatography (100% hexane to hexane 85:15 AcOEt) to afford product **35** (89 mg, 62% yield) as pale brown oil. This compound was previously unreported.

^{1}H NMR (300 MHz, $CDCl_3$) δ 8.34 (dt, *J* = 7.2, 1.0 Hz, 1H), 7.87 (d, *J* = 2.3 Hz, 1H), 7.26 (s, 1H), 6.57 (dd, *J* = 7.1, 1.9 Hz, 1H), 6.36 (dd, *J* = 2.3, 0.9 Hz, 1H), 2.66 – 2.55 (m, 2H), 1.84 – 1.67 (m, 2H), 1.23 (s, 12H), 0.83 (t, *J* = 7.8 Hz, 2H).

^{13}C NMR (75 MHz, $CDCl_3$) δ 142.0, 140.4, 138.5, 128.1, 116.1, 113.8, 95.7, 83.2, 37.9, 25.1, 24.9. The signal of the α-B-carbon was not observed.

HRMS (FD) *m/z* calcd for $C_{16}H_{23}B_1N_2O_2$: 287.1934; found: 287.1929.

6-(3-(4,4,5,5-tetramethyl-1,3,2-dioxaborolan-2-yl)propyl)pyrazolo[1,5-*a*]pyridine (36)

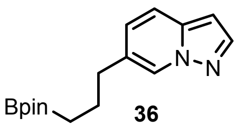

36

Prepared according to general procedure B2, 3-bromopropylboronic acid pinacol ester (264 µL, 1.25 mmol, 2.5 equiv.) and using 6-bromopyrazolo[1,5-*a*]pyridine (98.5 mg, 0.5 mmol, 1 equiv.) The crude mixture was purified by flash column chromatography (100% hexane to hexane 85:15 AcOEt) to afford product **36** (70 mg, 49% yield) as a pale brown oil. This compound was previously unreported.

^{1}H NMR (300 MHz, $CDCl_3$) δ 8.00 (dd, *J* = 6.9, 1.0 Hz, 1H), 7.56 (d, *J* = 1.3 Hz, 1H), 7.49 (t, *J* = 1.0 Hz, 1H), 7.40 – 7.34 (m, 1H), 6.63 (dd, *J* = 6.9, 1.7 Hz, 1H), 2.70 – 2.59 (m, 2H), 1.77 (dt, *J* = 15.4, 7.8 Hz, 2H), 1.24 (s, 12H), 0.82 (d, *J* = 7.8 Hz, 2H).

^{13}C NMR (101 MHz, $CDCl_3$) δ 141.4, 139.0, 126.6, 126.5, 125.9, 117.5, 96.3, 83.2, 35.1, 25.4, 25.0.

HRMS (FD) *m/z* calcd for $C_{16}H_{23}B_1N_2O_2$: 287.1934; found: 287.1919.

3.2. 1,2-Dicarbofunctionalization reaction

General Procedure C

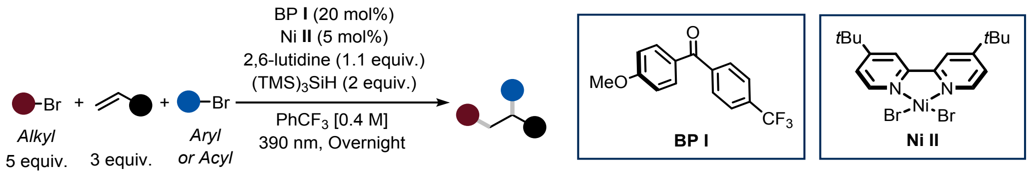

To a flame-dried argon-purged screw-capped vial, fitted with a rubber septum, charged with the nickel complex **II** (12.2 mg, 5 mol%), BP **I** (28.0 mg, 20 mol%) and the aryl bromide or acyl chloride (0.5 mmol, 1 equiv.) in $PhCF_3$ (1.25 mL, 0.40 M), the alkyl bromide derivative (2.5 mmol, 5 equiv.), 2,6-lutidine (64 µL, 0.55 mmol, 1.1 equiv.), $(TMS)_3SiH$ (309 µL, 1 mmol, 2 equiv.) and the olefin (1.5 mmol, 3 equiv.) were added. Subsequently, the reaction vessel was placed in an ice bath, sparged with nitrogen for 30 seconds and the reaction vessel was sealed with parafilm. The vial was then placed in a 3D-printed photoreactor (see Figure S1) using a Kessil Lamp (390 nm, 100% intensity) as light source and irradiated overnight. The solvent of the resulting reaction mixture was removed under reduced pressure and purification by flash column chromatography on silica gel, thin layer chromatography or high-performance liquid chromatography gave the corresponding products in the stated yield.

Characterization Data of Products

Methyl 4-(1-(*tert*-butoxy)-3-cyclohexyl-1-oxopropan-2-yl)benzoate (38)

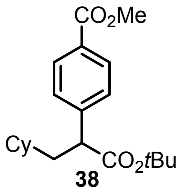

38

Prepared according to general procedure C, using cyclohexyl bromide (308 µL, 2.5 mmol, 5 equiv.), methyl 4-bromobenzoate (108 mg, 0.5 mmol, 1 equiv.) and *tert*-butyl acrylate (220 µL, 1.5 mmol, 3 equiv.). The crude mixture was purified by flash column chromatography (100% hexane to hexane 95:5 Et_2O, two consecutive runs) to afford product **38** (109 mg, 63% yield) as an off-white solid. This compound was previously unreported.

^{1}H NMR (400 MHz, $CDCl_3$) δ 8.01 – 7.93 (m, 2H), 7.40 – 7.32 (m, 2H), 3.89 (s, 3H), 3.62 (t, J = 7.8 Hz, 1H), 1.93 (ddd, J = 13.8, 8.4, 7.1 Hz, 1H), 1.77 – 1.53 (m, 6H), 1.37 (s, 9H), 1.18 – 1.04 (m, 4H), 0.97 – 0.79 (m, 2H).
^{13}C NMR (101 MHz, $CDCl_3$) δ 173.0, 167.1, 145.5, 129.9, 128.9, 128.1, 80.9, 52.1, 50.1, 41.1, 35.5, 33.4, 33.2, 28.0, 26.6, 26.3, 26.2.
HRMS (FD) *m/z* calcd for $C_{21}H_{30}O_4$: 346.2144; found: 346.2132.

***Tert*-butyl 3-cyclohexyl-2-(4-(4,4,5,5-tetramethyl-1,3,2-dioxaborolan-2 yl)phenyl) propanoate (39)**

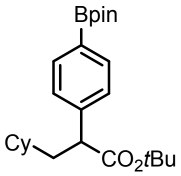

39

Prepared according to general procedure C, using cyclohexyl bromide (308 µL, 2.5 mmol, 5 equiv.), 2-(4-bromophenyl)-4,4,5,5-tetramethyl-1,3,2-dioxaborolane (141 mg, 0.5 mmol, 1 equiv.) and *tert*-butyl acrylate (220 µL, 1.5 mmol, 3 equiv.). The crude mixture was purified first by flash column chromatography (100% hexane to hexane 95:5 Et_2O) and subsequently by preparative HPLC (100% hexane to hexane 95:5 AcOEt) to afford product **39** (121 mg, 45% yield) as a white solid. The spectroscopic data are consistent with those reported previously.[15]

^{1}H NMR (400 MHz, $CDCl_3$) δ 7.74 (d, J = 8.0 Hz, 2H), 7.30 (d, J = 8.0 Hz, 2H), 3.58 (t, J = 7.8 Hz, 1H), 1.91 (dt, J = 13.6, 7.8 Hz, 1H), 1.83 – 1.50 (m, 6H), 1.37 (s, 9H), 1.34 (s, 12H), 1.18 – 1.09 (m, 4H), 0.96 – 0.70 (m, 2H).

^{13}C NMR (101 MHz, $CDCl_3$) δ 173.5, 143.5, 135.0, 127.5, 83.9, 80.6, 50.2, 41.1, 35.5, 33.4, 33.2, 28.1, 26.7, 26.3, 26.3, 25.0, 25.0. The signal of the α-B-carbon was not observed.

***Tert*-butyl 3-cyclohexyl-2-(4-methoxyphenyl)propanoate (40)**

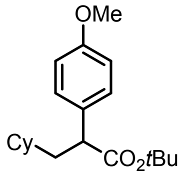

40

Prepared according to general procedure C, using cyclohexyl bromide (308 μL, 2.5 mmol, 5 equiv.), 1-bromo-4-methoxybenzene (63 μL, 0.5 mmol, 1 equiv.) and *tert*-butyl acrylate (220 μL, 1.5 mmol, 3 equiv.). The crude mixture was purified first by flash column chromatography (100% hexane to hexane 90:10 Et_2O) and subsequently by preparative TLC (hexane 70:30 toluene) to afford product **40** (64 mg, 40% yield) as a yellow wax. This compound was previously unreported.

^1H NMR (400 MHz, $CDCl_3$) δ 7.25 – 7.17 (m, 2H), 6.88 – 6.79 (m, 2H), 3.79 (s, 3H), 3.51 (dd, J = 8.4, 7.3 Hz, 1H), 1.89 (ddd, J = 13.6, 8.4, 6.9 Hz, 1H), 1.77 – 1.51 (m, 6H), 1.38 (s, 9H), 1.23 – 1.08 (m, 4H), 0.96 – 0.78 (m, 2H).

^{13}C NMR (101 MHz, $CDCl_3$) δ 174.1, 158.6, 132.4, 129.0, 113.9, 80.4, 55.3, 49.1, 41.4, 35.5, 33.4, 33.2, 28.1, 26.7, 26.4, 26.3.

HRMS (FD) *m/z* calcd for $C_{20}H_{30}O_3$: 318.2195; found: 318.2200.

***Tert*-butyl 2-(3-chloro-4-fluorophenyl)-3-cyclohexylpropanoate (41)**

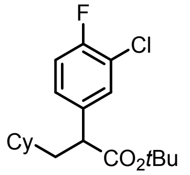

41

Prepared according to general procedure C, using cyclohexyl bromide (308 μL, 2.5 mmol, 5 equiv.), 4-bromo-2-chloro-1-fluorobenzene (61 μL, 0.5 mmol, 1 equiv.) and *tert*-butyl acrylate (220 μL, 1.5 mmol, 3 equiv.). The crude mixture was purified by flash column chromatography (100% hexane to hexane 95:5 Et_2O, two consecutive runs) to afford product **41** (99 mg, 58% yield) as a white solid. This compound was previously unreported.

^1H NMR (400 MHz, $CDCl_3$) δ 7.35 (dd, J = 7.0, 2.3 Hz, 1H), 7.16 (ddd, J = 8.5, 4.6, 2.3 Hz, 1H), 7.06 (t, J = 8.7 Hz, 1H), 3.52 (dd, J = 8.5, 7.3 Hz, 1H), 1.89 (ddd, J = 13.6, 8.5, 6.9 Hz, 1H), 1.76 – 1.49 (m, 6H), 1.39 (s, 9H), 1.24 – 1.06 (m, 4H), 0.98 – 0.80 (m, 2H), .

^{13}C NMR (101 MHz, $CDCl_3$) δ 173.0, 157.2 (d, J = 247.8 Hz), 137.2 (d, J = 3.6 Hz), 130.1, 127.7 (d, J = 6.9 Hz), 120.9 (d, J = 17.8 Hz), 116.5 (d, J = 21.1 Hz), 81.1, 49.1, 41.3, 35.5, 33.3, 33.2, 28.1, 26.6, 26.3, 26.2.

^{19}F NMR (376 MHz, $CDCl_3$) δ -118.29 (td, J = 8.0, 4.5 Hz).

HRMS (FD) *m/z* calcd for $C_{19}H_{26}ClFO_2$: 340.1605; found: 340.1595.

***Tert*-butyl 3-cyclohexyl-2-(6-(trifluoromethyl)pyridin-3-yl)propanoate (42)**

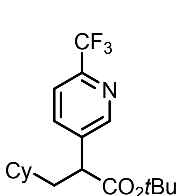

42

Prepared according to general procedure C, using cyclohexyl bromide (308 μL, 2.5 mmol, 5 equiv.), methyl 4-bromobenzoate (108 mg, 0.5 mmol, 1 equiv.) and *tert*-butyl acrylate (220 μL, 1.5 mmol, 3 equiv.). The crude mixture was purified first by flash column chromatography (100% hexane to hexane 95:5 Et_2O) and subsequently by preparative HPLC (100% hexane to hexane 95:5 AcOEt) to afford product **42** (81 mg, 45% yield) as a white solid. This compound was previously unreported.

^1H NMR (400 MHz, $CDCl_3$) δ 8.62 (d, J = 2.4 Hz, 1H), 7.86 (dd, J = 8.1, 2.4 Hz, 1H), 7.64 (dd, J = 8.2, 0.7 Hz, 1H), 3.68 (t, J = 7.8 Hz, 1H), 1.97 (ddd, J = 13.8, 8.3, 7.1 Hz, 1H), 1.82 – 1.55 (m, 6H), 1.40 (s, 9H), 1.19 – 1.07 (m, 4H), 0.99 – 0.85 (m, 2H).

^{13}C NMR (101 MHz, $CDCl_3$) δ 172.2, 150.0, 147.0 (q, J = 34.7 Hz), 139.1, 136.6, 121.7 (q, J = 273.8 Hz), 120.4 (q, J = 2.9 Hz), 81.8, 47.4, 41.3, 35.5, 33.3, 33.0, 28.1, 26.5, 26.2, 26.2.

^{19}F NMR (376 MHz, $CDCl_3$) δ -67.79.

HRMS (ESI) *m/z* calcd for $C_{19}H_{27}F_3NO_2$ $[M+H^+]$: 358.1994; found: 358.1990.

***Tert*-butyl 3-cyclohexyl-2-(dibenzo[*b,d*]furan-2-yl)propanoate (43)**

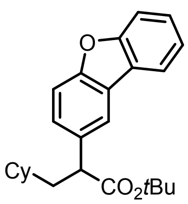

43

Prepared according to general procedure C, using cyclohexyl bromide (308 μL, 2.5 mmol, 5 equiv.), 2-bromodibenzo[*b,d*]furan (123 mg, 0.5 mmol, 1 equiv.) and *tert*-butyl acrylate (220 μL, 1.5 mmol, 3 equiv.). The crude mixture was purified by flash column chromatography (100% hexane to hexane 95:5 Et_2O, two consecutive runs) to afford product **43** (102 mg, 54% yield) as a white solid. This compound was previously unreported.

^1H NMR (400 MHz, $CDCl_3$) δ 7.96 (dt, J = 7.6, 1.0 Hz, 1H), 7.91 (d, J = 2.0 Hz, 1H), 7.56 (d, J = 8.1 Hz, 1H), 7.51 (d, J = 8.5 Hz, 1H), 7.47 – 7.38 (m, 2H), 7.34 (td, J = 7.4, 1.1 Hz, 1H), 3.75 (t, J = 7.8 Hz, 1H), 2.03 (ddd, J = 13.6, 8.4, 6.9 Hz, 1H), 1.85 – 1.58 (m, 6H), 1.41 (s, 9H), 1.29 – 1.10 (m, 4H), 1.02 – 0.89 (m, 2H).
^{13}C NMR (101 MHz, $CDCl_3$) δ 174.0, 156.6, 155.4, 134.8, 127.3, 127.2, 124.5, 124.4, 122.8, 120.8, 119.9, 111.8, 111.5, 80.7, 49.9, 41.7, 35.6, 33.4, 33.2, 28.1, 26.7, 26.3, 26.3.
HRMS (FD) *m/z* calcd for $C_{25}H_{30}O_3$: 378.2195; found: 378.2209.

Methyl 4-(1-(*tert*-butoxy)-4-methyl-1-oxopentan-2-yl)benzoate (44)

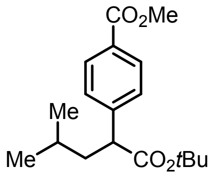

44

Prepared according to general procedure C, using 2-bromopropane (235 μL, 2.5 mmol, 5 equiv.), methyl 4-bromobenzoate (108 mg, 0.5 mmol, 1 equiv.) and *tert*-butyl acrylate (220 μL, 1.5 mmol, 3 equiv.). The crude mixture was purified by flash column chromatography (100% hexane to hexane 95:5 Et_2O, two consecutive runs) to afford product **44** (100 mg, 65% yield) as a white solid. This compound was previously unreported.

^1H NMR (400 MHz, $CDCl_3$) δ 7.99 – 7.92 (m, 2H), 7.40 – 7.33 (m, 2H), 3.89 (s, 3H), 3.59 (t, J = 7.8 Hz, 1H), 1.93 (ddd, J = 13.6, 8.3, 7.2 Hz, 1H), 1.60 (dt, J = 13.6, 7.1 Hz, 1H), 1.53 – 1.40 (m, 1H), 1.37 (s, 9H), 0.89 (d, J = 6.5 Hz, 6H).
^{13}C NMR (101 MHz, $CDCl_3$) δ 172.8, 167.1, 145.4, 129.9, 128.9, 128.1, 80.9, 52.1, 50.9, 42.4, 28.0, 26.1, 22.7, 22.4.
HRMS (FD) *m/z* calcd for $C_{18}H_{26}O_4$: 306.1831; found: 306.1839.

Methyl 4-(1-(*tert*-butoxy)-4,4-dimethyl-1-oxopentan-2-yl)benzoate (45)

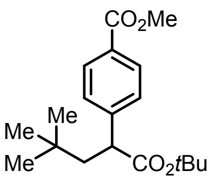

45

Prepared according to general procedure C, using 2-bromo-2-methylpropane (281 μL, 2.5 mmol, 5 equiv.), methyl 4-bromobenzoate (108 mg, 0.5 mmol, 1 equiv.) and *tert*-butyl acrylate (220 μL, 1.5 mmol, 3 equiv.). The crude mixture was purified by flash column chromatography (100% hexane to hexane 95:5 Et_2O, two consecutive runs) to afford product **45** (102 mg, 64% yield) as an off-white solid. The spectroscopic data are consistent with those reported previously.[16]

^1H NMR (400 MHz, $CDCl_3$) δ 7.99 – 7.91 (m, 2H), 7.40 – 7.32 (m, 2H), 3.88 (s, 3H), 3.58 (dd, J = 9.0, 3.8 Hz, 1H), 2.26 (dd, J = 14.0, 9.0 Hz, 1H), 1.49 (dd, J = 14.0, 3.8 Hz, 1H), 1.35 (s, 9H), 0.87 (s, 9H).
^{13}C NMR (101 MHz, $CDCl_3$) δ 173.3, 167.1, 146.9, 129.9, 128.8, 127.9, 80.8, 52.1, 49.5, 47.0, 31.2, 29.6, 27.9.

Methyl 4-(1-(*tert*-butoxy)-1-oxo-3-(tetrahydro-*2H*-pyran-4-yl)propan-2-yl)benzoate (46)

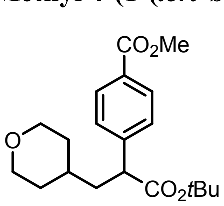

46

Prepared according to general procedure C, using 4-bromotetrahydro-*2H*-pyran (281 μL, 2.5 mmol, 5 equiv.), methyl 4-bromobenzoate (108 mg, 0.5 mmol, 1 equiv.) and *tert*-butyl acrylate (220 μL, 1.5 mmol, 3 equiv.). The crude mixture was purified first by flash column chromatography (100% hexane to hexane 85:15 Et_2O) and subsequently by preparative HPLC

(100% hexane to hexane 90:10 AcOEt) to afford product **46** (97 mg, 56% yield) as an off-white solid. This compound was previously unreported.

^{1}H NMR (400 MHz, $CDCl_3$) δ 8.02 – 7.94 (m, 2H), 7.40 – 7.32 (m, 2H), 3.96 – 3.84 (m, 5H), 3.62 (t, J = 7.8 Hz, 1H), 3.34 – 3.23 (m, 2H), 2.00 (ddd, J = 13.8, 8.3, 6.8 Hz, 1H), 1.76 – 1.50 (m, 3H), 1.47 – 1.22 (m, 12H).

^{13}C NMR (101 MHz, $CDCl_3$) δ 172.6, 167.0, 144.9, 130.0, 129.1, 128.0, 81.2, 68.0, 67.9, 52.2, 49.6, 40.3, 33.1, 33.0, 32.9, 28.0.

HRMS (FD) *m/z* calcd for $C_{20}H_{28}O_5$: 348.1937; found: 348.1950.

Benzyl 4-(3-(*tert*-butoxy)-2-(4-(methoxycarbonyl)phenyl)-3-oxopropyl)piperidine-1-carboxylate (47)

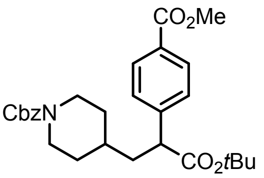

47

Prepared according to general procedure C, using benzyl 4-bromopiperidine-1-carboxylate (542 µL, 2.5 mmol, 5 equiv.), methyl 4-bromobenzoate (108 mg, 0.5 mmol, 1 equiv.) and *tert*-butyl acrylate (220 µL, 1.5 mmol, 3 equiv.). The crude mixture was purified first by flash column chromatography (100% hexane to hexane 85:15 Et_2O) and subsequently by preparative HPLC (100% hexane to hexane 90:10 AcOEt) to afford product **47** (108 mg, 45% yield) as a colorless wax. This compound was previously unreported.

^{1}H NMR (400 MHz, $CDCl_3$) δ 8.02 – 7.95 (m, 2H), 7.40 – 7.26 (m, 7H), 5.11 (s, 2H), 4.25 – 4.05 (bs, 2H), 3.91 (s, 3H), 3.61 (t, J = 7.8 Hz, 1H), 2.80 – 2.57 (bs, 2H) 1.99 (dt, J = 14.6, 7.6 Hz, 1H), 1.67 (p, J = 6.6 Hz, 2H), 1.47 – 1.40 (m, 1H), 1.38 – 1.25 (m, 10H), 1.21 – 1.05 (m, 2H).

^{13}C NMR (101 MHz, $CDCl_3$) δ 172.5, 167.0, 155.3, 144.8, 137.0, 130.0, 129.2, 128.6, 128.1, 128.0, 128.0, 81.3, 67.1, 52.3, 49.9, 44.1, 44.1, 39.9, 33.9, 28.0.

HRMS (FD) *m/z* calcd for $C_{28}H_{35}NO_6$: 481.2464; found: 481.2503.

Methyl 4-(3-(3-bromoadamantan-1-yl)-1-(*tert*-butoxy)-1-oxopropan-2-yl)benzoate (48)

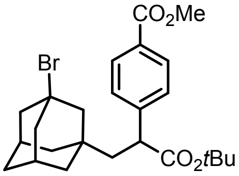

48

Prepared according to general procedure C, using 1,3-dibromoadamantane (735 mg, 2.5 mmol, 5 equiv.), methyl 4-bromobenzoate (108 mg, 0.5 mmol, 1 equiv.) and *tert*-butyl acrylate (220 µL, 1.5 mmol, 3 equiv.). The crude mixture was purified by flash column chromatography (100% hexane to hexane 95:5 Et_2O, two consecutive runs) to afford product **48** (160 mg, 67% yield) as a colorless wax. This compound was previously unreported.

^{1}H NMR (400 MHz, $CDCl_3$) δ 8.00 – 7.92 (m, 2H), 7.38 – 7.30 (m, 2H), 3.89 (s, 3H), 3.61 (dd, J = 9.4, 3.6 Hz, 1H), 2.35 – 2.00 (m, 10H), 1.75 – 1.48 (m, 4H), 1.43 – 1.39 (m, 2H), 1.36 (s, 9H).

^{13}C NMR (101 MHz, $CDCl_3$) δ 172.9, 167.0, 146.4, 130.0, 129.0, 127.8, 81.2, 65.9, 53.7, 52.2, 48.6, 48.6, 47.5, 46.6, 40.6, 40.4, 38.3, 34.9, 32.5, 32.5, 27.9.

Methyl 4-(2-cyclohexyl-1-(4,4,5,5-tetramethyl-1,3,2-dioxaborolan-2-yl)ethyl)benzoate (49)

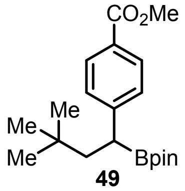

49

Prepared according to general procedure C, using 2-bromo-2-methylpropane (281 µL, 2.5 mmol, 5 equiv.), methyl 4-bromobenzoate (108 mg, 0.5 mmol, 1 equiv.) and 4,4,5,5-tetramethyl-2-vinyl-1,3,2-dioxaborolane (254 µL, 1.5 mmol, 3 equiv.). The crude mixture was purified by flash column chromatography (100% hexane to hexane 95:5 Et_2O) to afford product **49** (125 mg, 72% yield) as a white solid. The spectroscopic data are consistent with those reported previously.[16]

^{1}H NMR (400 MHz, $CDCl_3$) δ 7.94 – 7.86 (m, 2H), 7.32 – 7.24 (m, 2H), 3.87 (s, 3H), 2.47 (dd, *J* = 9.5, 4.3 Hz, 1H), 2.02 (dd, *J* = 13.4, 9.5 Hz, 1H), 1.51 (dd, *J* = 13.4, 4.3 Hz, 1H), 1.12 (d, *J* = 1.1 Hz, 12H), 0.88 (s, 9H).
^{13}C NMR (101 MHz, $CDCl_3$) δ 167.4, 150.9, 129.7, 128.2, 127.1, 83.5, 52.0, 46.1, 31.6, 29.8, 24.6, 24.5. The signal of the α-B-carbon was not observed.

Methyl 4-(4,4-dimethyl-1-(((8R,9S,13S,14S)-13-methyl-17-oxo-7,8,9,11,12,13,14,15,16,17-decahydro-6H-cyclopenta[*a*]phenanthren-3-yl)oxy)-1-oxopentan-2-yl)benzoate (50)

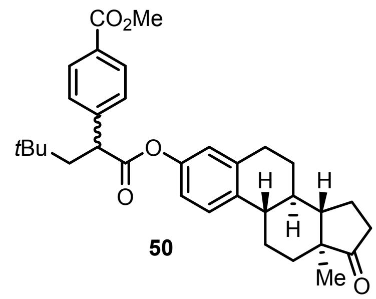

50

Prepared according to general procedure C, using 2-bromo-2-methylpropane (281 µL, 2.5 mmol, 5 equiv.), methyl 4-bromobenzoate (108 mg, 0.5 mmol, 1 equiv.) and (8R,9S,13S,14S)-13-methyl-17-oxo-7,8,9,11,12,13,14,15,16,17-decahydro-6H-cyclopenta[*a*]phenanthren-3-yl acrylate (486 mg, 1.5 mmol, 3 equiv.). The crude mixture was purified by flash column chromatography (100% hexane to hexane 90:10 Et_2O) to afford product **50** (140 mg, 54% yield, 1:1 d.r.) as an off-white solid. This compound was previously unreported.
^{1}H NMR (400 MHz, $CDCl_3$) δ 8.02 (d, *J* = 8.4 Hz, 2H), 7.49 (d, *J* = 8.4 Hz, 2H), 7.23 (d, *J* = 8.6 Hz, 1H), 6.72 (dt, *J* = 8.5, 2.9 Hz, 1H), 6.66 (t, *J* = 2.8 Hz, 1H), 3.97 – 3.89 (m, 4H), 2.89 – 2.81 (m, 2H), 2.71 – 1.85 (m, 8H), 1.77 – 1.37 (m, 7H), 0.99 (s, 9H), 0.88 (s, 3H).
^{13}C NMR (75 MHz, $CDCl_3$) δ 220.7, 172.8, 166.8, 148.6, 145.6, 138.0, 137.5, 130.1, 129.2, 128.0, 126.4, 121.2, 118.4, 52.1, 50.4, 48.4, 47.9, 47.2, 44.1, 38.0, 35.9, 31.5, 31.2, 29.5, 29.4, 26.3, 25.8, 21.6, 13.8.
HRMS (FD) *m/z* calcd for $C_{33}H_{40}O_5$: 516.2876; found: 516.2899.

Methyl 4-(4-(*tert*-butyl)-2-oxotetrahydrofuran-3-yl)benzoate (51)

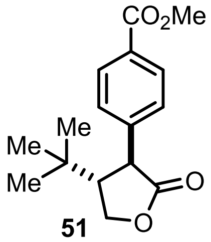

51

Prepared according to general procedure C, using 2-bromo-2-methylpropane (281 µL, 2.5 mmol, 5 equiv.), methyl 4-bromobenzoate (108 mg, 0.5 mmol, 1 equiv.) and furan-2(5H)-one (106 µL, 1.5 mmol, 3 equiv.). The crude mixture was purified by flash column chromatography (100% hexane to hexane 95:5 Et_2O, two consecutive runs) to afford product **51** (84 mg, 61% yield, >20:1 d.r.) as a pale yellow solid. This compound was previously unreported.
^{1}H NMR (400 MHz, $CDCl_3$) δ 8.06 – 7.98 (m, 2H), 7.34 – 7.26 (m, 2H), 4.44 (dd, *J* = 9.4, 8.1 Hz, 1H), 4.17 (dd, *J* = 9.4, 8.4 Hz, 1H), 3.89 (s, 3H), 3.67 (d, *J* = 9.5 Hz, 1H), 2.63 (dt, *J* = 9.6, 8.3 Hz, 1H), 0.86 (s, 9H).
^{13}C NMR (101 MHz, $CDCl_3$) δ 177.3, 166.7, 143.6, 130.4, 129.6, 128.5, 68.3, 54.3, 52.2, 48.9, 32.3, 27.2.
HRMS (FD) *m/z* calcd for $C_{16}H_{20}O_4$: 276.1362; found: 276.1354.

***Tert*-butyl 2-(4-methoxybenzoyl)-4,4-dimethylpentanoate (52)**

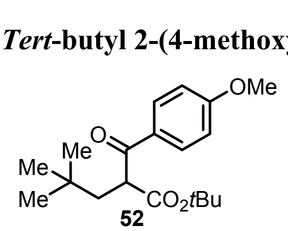

52

Prepared according to general procedure C, 2-bromo-2-methylpropane (281 µL, 2.5 mmol, 5 equiv.), 4-methoxybenzoyl chloride (68 µL, 0.5 mmol, 1 equiv.) and *tert*-butyl acrylate (220 µL, 1.5 mmol, 3 equiv.). The crude mixture was purified first by flash column chromatography (100% hexane to hexane 90:10 Et_2O) and subsequently by preparative HPLC (100% hexane to hexane 90:10 AcOEt) to afford product **52** (104 mg, 65% yield) as a white solid. This compound was previously unreported.

^{1}H NMR (400 MHz, $CDCl_3$) δ 8.04 – 7.95 (m, 2H), 7.01 – 6.91 (m, 2H), 4.21 (t, *J* = 5.9 Hz, 1H), 3.87 (s, 3H), 1.97 (d, *J* = 5.9 Hz, 2H), 1.35 (s, 9H), 0.91 (s, 9H).
^{13}C NMR (101 MHz, $CDCl_3$) δ 194.6, 170.0, 163.7, 131.2, 129.4, 113.9, 81.6, 55.6, 52.0, 41.7, 31.0, 29.6, 27.9.
HRMS (FD) *m/z* calcd for $C_{19}H_{28}O_4$: 320.1988; found: 320.2044.

***Tert*-butyl 4,4-dimethyl-2-(4-(trifluoromethyl)benzoyl)pentanoate (53)**

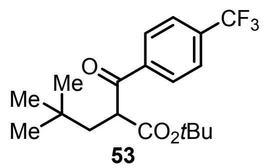

53

Prepared according to general procedure C, 2-bromo-2-methylpropane (281 μL, 2.5 mmol, 5 equiv.), 4-(trifluoromethyl)benzoyl chloride (75 μL, 0.5 mmol, 1 equiv.) and *tert*-butyl acrylate (220 μL, 1.5 mmol, 3 equiv.). The crude mixture was purified first by flash column chromatography (100% hexane to hexane 95:5 Et_2O) and subsequently by reverse phase chromatography (100% CH_3CN to CH_3CN 90:10 H_2O) to afford product **53** (76 mg, 42% yield) as a colorless liquid. This compound was previously unreported.
^{1}H NMR (300 MHz, $CDCl_3$) δ 8.13 – 8.06 (m, 2H), 7.74 (d, *J* = 8.8 Hz, 2H), 4.23 (t, *J* = 5.9 Hz, 1H), 2.07 – 1.93 (m, 2H), 1.34 (s, 9H), 0.90 (s, 9H).
^{13}C NMR (101 MHz, $CDCl_3$) δ 195.2, 169.3, 139.3, 134.6 (q, *J* = 32.7 Hz), 129.1, 125.8 (q, *J* = 3.8 Hz), 123.7 (q, *J* = 272.8 Hz), 82.3, 52.6, 41.5, 30.9, 29.6, 27.9.
^{19}F NMR (376 MHz, $CDCl_3$) δ -63.16.
HRMS (FD) *m/z* calcd for $C_{19}H_{25}F_3O_4$: 358.1756; found: 358.1770.

***Tert*-butyl 2-neopentyl-3-oxohexanoate (54)**

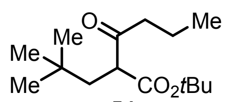

54

Prepared according to general procedure C, 2-bromo-2-methylpropane (281 μL, 2.5 mmol, 5 equiv.), butyryl chloride (52 μL, 0.5 mmol, 1 equiv.) and *tert*-butyl acrylate (220 μL, 1.5 mmol, 3 equiv.). The crude mixture was purified by flash column chromatography (100% hexane to hexane 95:5 Et_2O, two consecutive runs) to afford product **54** (62 mg, 48% yield) as a pale yellow liquid. This compound was previously unreported.
^{1}H NMR (300 MHz, $CDCl_3$) δ 3.37 (dd, *J* = 7.4, 4.5 Hz, 1H), 2.63 – 2.37 (m, 2H), 1.85 (dd, *J* = 14.3, 7.4 Hz, 1H), 1.71 (dd, *J* = 14.3, 4.5 Hz, 1H), 1.60 (q, *J* = 7.3 Hz, 2H), 1.44 (s, 9H) 0.94 – 0.84 (m, 12H).
^{13}C NMR (101 MHz, $CDCl_3$) δ 205.8, 169.9, 81.6, 57.3, 43.4, 40.9, 30.7, 29.4, 28.0, 17.2, 13.8.
HRMS (FD) *m/z* calcd for $C_{18}H_{28}O_3$: 256.2038; found: 256.2045.

4. Limitation of the Scope

For what concerns the direct coupling reaction, the protocol seems not to allow the use redox active ester (RAE), tertiary derivatives, allyl radical precursor and some electron rich heteroarenes.

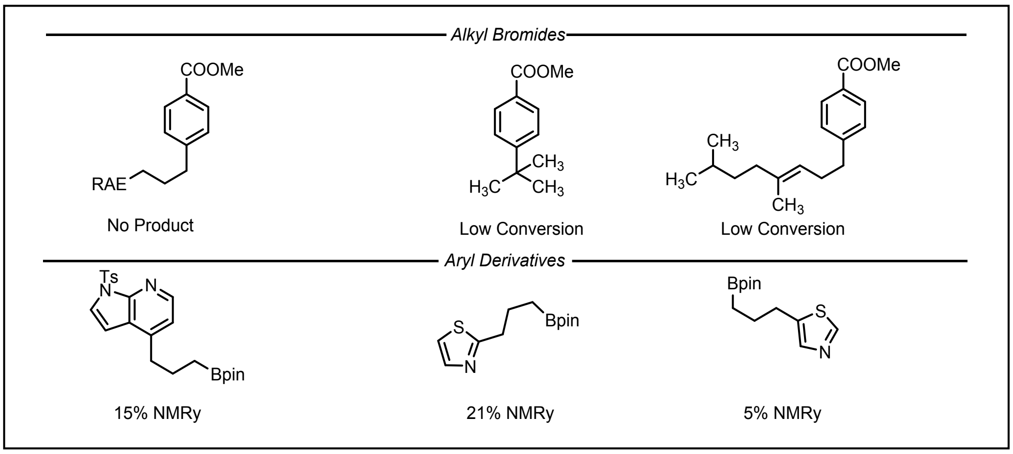

Figure S5 : Overview of some unsuccessful substrates due to low yield or no reaction in the direct coupling reaction.

For what concerns the difunctionalization reaction, the protocol seems not to allow the use of primary derivatives. Indeed, when testing this type of compounds, we observed mostly the direct coupling with the aryl substrate.

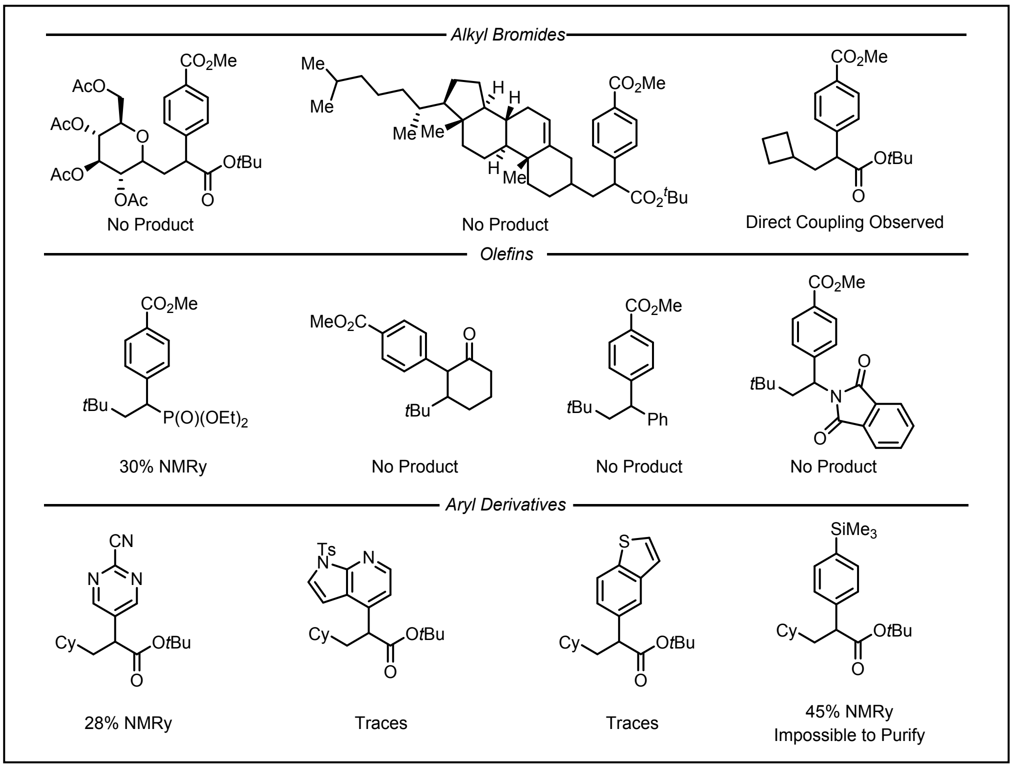

Figure S6 Overview of some unsuccessful substrates due to low yield or no reaction for the difunctionalization protocol.

5. Mechanistic Studies

5.1 Photophysical Studies

UV-Vis spectroscopy

First, the absorption spectra of the photocatalyst ((4-methoxyphenyl)(4-(trifluoromethyl)phenyl)methanone) BP **I** and Ni(dtbbpy)Br_2 (dtbbpy: 4,4'-di-tert-butyl-2,2'-bipyridine) Ni **II**, both $1.0 \cdot 10^{-4}$ M in CH_3CN, were taken (Figure S7).

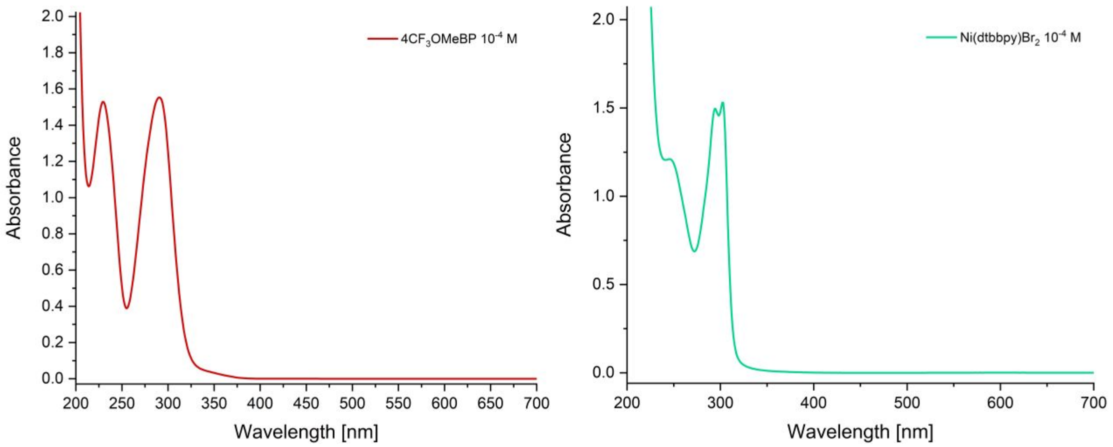

Figure S7: Absorption spectra of the employed 4,4'-disubstituted benzophenone BP **I** ((4-methoxyphenyl)(4-(trifluoromethyl)phenyl)methanone) (left) and Ni(dtbbpy)Br_2 (dtbbpy: 4,4'-di-tert-butyl-2,2'-bipyridine) Ni **II** (right), both $1.0 \cdot 10^{-4}$ M in CH_3CN.

Next, the absorption spectra of the photocatalyst, the nickel complex and the mixture of the two, were recorded in the same concentrations as the optimized conditions ([BP **I**]: $2.0 \cdot 10^{-2}$ M; [Ni **II**]: $5.0 \cdot 10^{-3}$ M in CH_3CN).

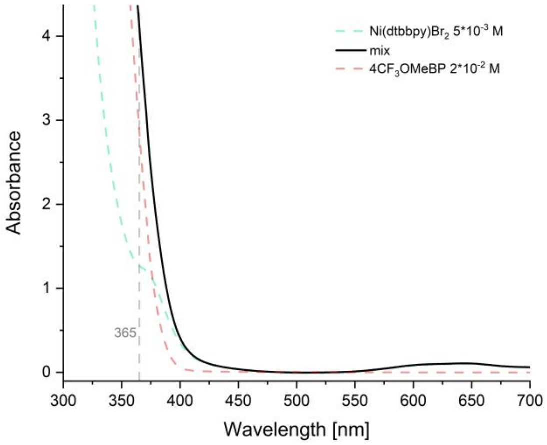

Figure S8: Absorption spectrum of the employed 4,4'-disubstituted benzophenone ((4-methoxyphenyl)(4-(trifluoromethyl)phenyl)methanone) BP **I**, Ni(dtbbpy)Br_2 (dtbbpy: 4,4'-di-tert-butyl-2,2'-bipyridine) Ni **II** and the mixture of the two. Concentrations are indicated in the legend and correspond to those adopted in the optimized reaction conditions. Irradiation wavelength for the flow setup (λ = 365 nm) is highlighted.

Accordingly, we set out to measure the molar extinction coefficient (ε) at 365 nm Figure S9 and values of 145 and 84 $M^{-1} \cdot cm^{-1}$ were found for photocatalyst BP **I** and nickel complex Ni **II**, respectively.

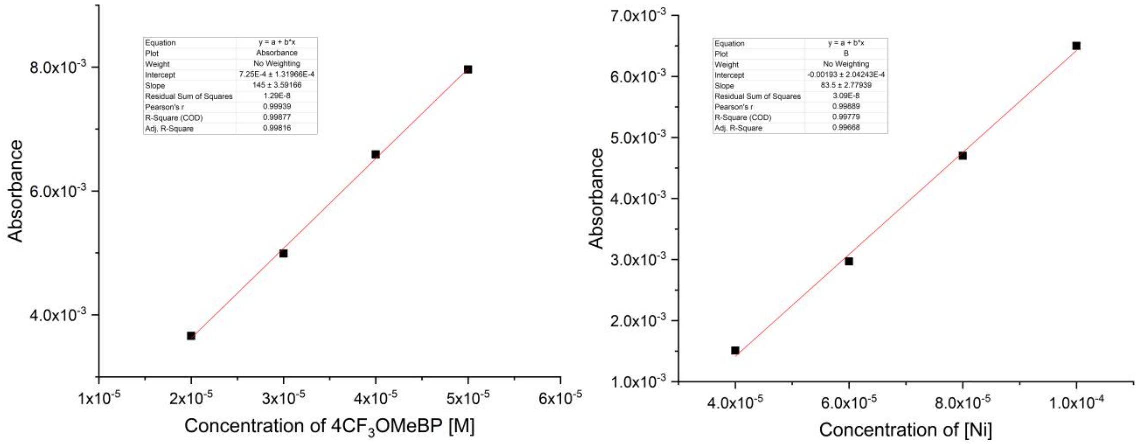

Figure S9 Plots of absorbance vs concentration for the photocatalyst BP **I** ((4-methoxyphenyl)(4-(trifluoromethyl)phenyl)methanone) (left) and Ni(dtbbpy)Br_2 (dtbbpy: 4,4'-di-tert-butyl-2,2'-bipyridine) Ni **II** (right) to determine the molar extinction coefficients.

Emission spectroscopy

When a N_2-bubbled solution of BP **I** ($5 \cdot 10^{-4}$ M in CH_3CN) was excited at 362 nm (A = 0.1), a luminescence was detected (Figure S10). Given the wide literature documenting the highly efficient inter-system crossing in diaryl ketones,[1,17] we attributed this luminescence to the triplet excited state.

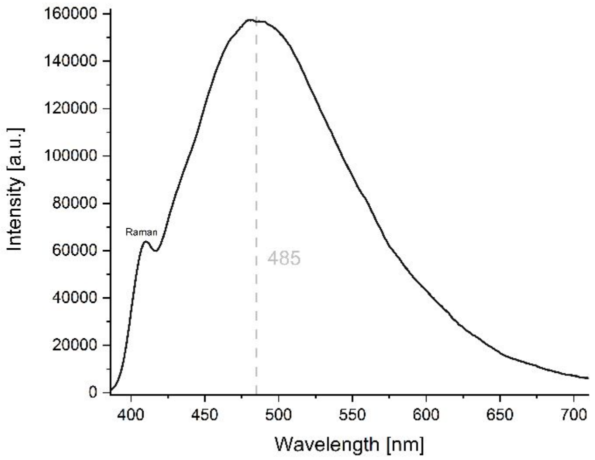

Figure S10 Luminescence spectrum of the photocatalyst BP **I** ($5 \cdot 10^{-4}$ M in CH_3CN); $_{exc}$ = 362 nm, A = 0.1.

Next, we monitored the luminescence decay in the presence of increasing amounts of $(TMS)_3SiH$ and a quenching of the signal was observed (Figure S11 left). The corresponding Stern-Volmer plot, built by monitoring the decay at 485 nm using the following equation S1

$$\frac{I_0}{I} = 1 + k_{SV}[Q] \qquad \text{[Eq. S1]}$$

is reported in Figure S11 right. A Stern-Volmer constant (k_{SV}) of 121 M^{-1} is calculated.

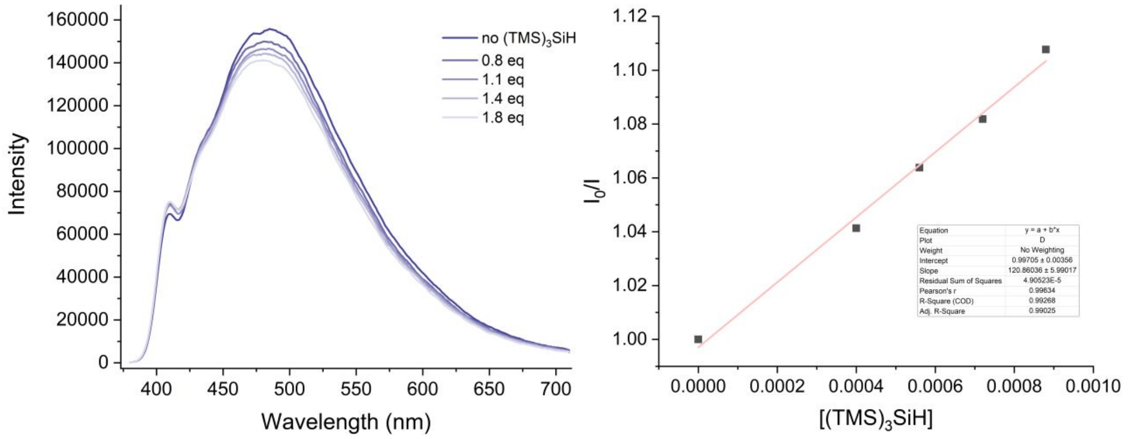

Figure S11 Luminescence decay in the presence of increasing amounts of $(TMS)_3SiH$ as a quencher (left) and the corresponding Stern-Volmer plot (right). Solvent: CH_3CN. [BP **I**] = $5\cdot10^{-4}$ M. $(TMS)_3SiH$ added as a 0.05 M solution in CH_3CN.

The very same experiment was performed by adopting tetrabutylammonium bromide (TBABr) as the quencher (Figure S12 left). TBABr served as a source of bromide ions.

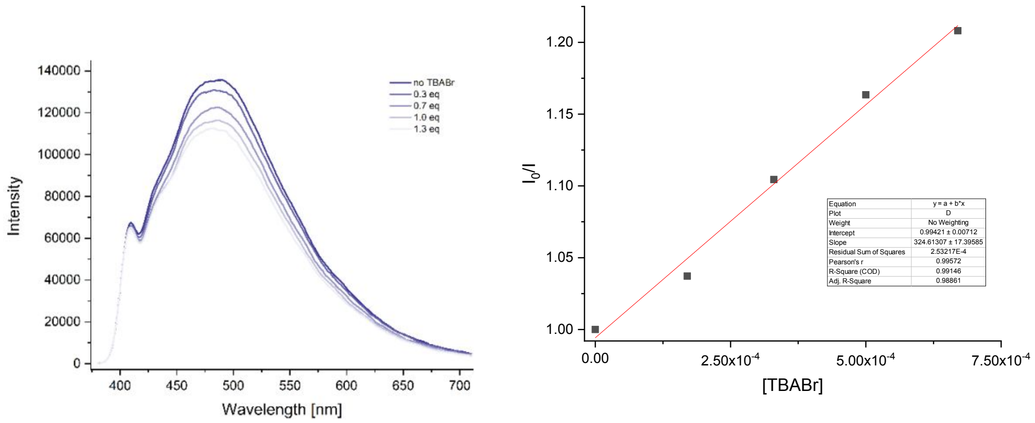

Figure S12: Luminescence decay in the presence of increasing amounts of TBABr as a quencher (left) and the corresponding Stern-Volmer plot (right). Solvent: CH_3CN. [BP **I**] = $5\cdot10^{-4}$ M. TBABr added as a 0.1 M solution in CH_3CN.

The corresponding Stern-Volmer plot, built by monitoring the decay at 485 nm using the following equation Eq. S2

$$\frac{I_0}{I} = 1 + k_{SV}[Q] \qquad \text{[Eq. S1]}$$

is reported in Figure S12 right. A Stern-Volmer constant (k_{SV}) of 325 M^{-1} is calculated. As the two Stern-Volmer constants are similar and, in fact, bromide seems to be a faster quencher than $(TMS)_3SiH$, we envisioned two mechanisms.[9,18]

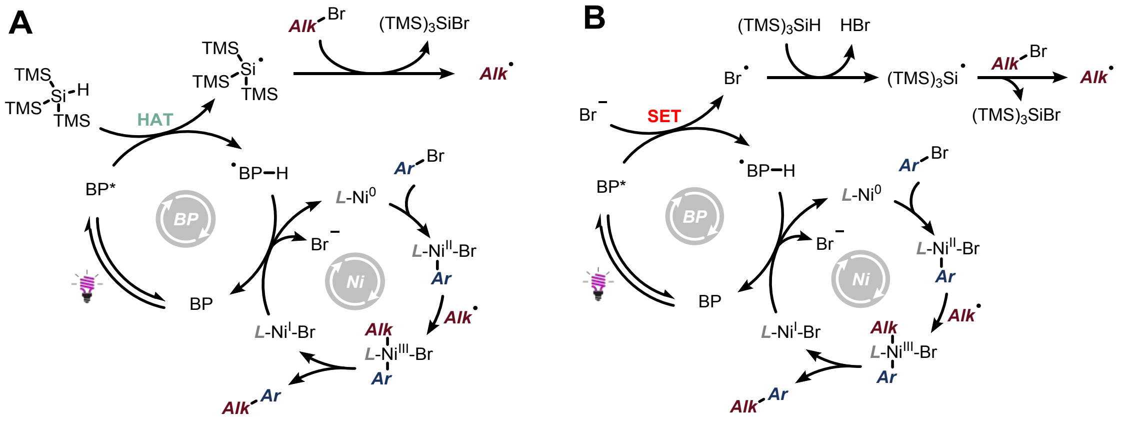

Figure S13 Two mechanistic scenario accounting for the observed reactivity: A) BP **I** acts via HAT activates $(TMS)_3SiH$ to trigger the XAT step or B) BP **I** acts as a photoredox catalyst to oxidize bromide to afford a bromine atom, which is responsible for the activation of the silane.

Transient absorption spectroscopy

Starting solutions of BP **I** in CH_3CN (bubbled with N_2 for 10 minutes) were employed to have an optical density of 1.0 at 319 nm ($5 \cdot 10^{-4}$ M). The sample solution was placed in a 1×1 cm quartz cell and excited with single pulses (~1 mJ, 5 Hz). The transient absorption spectrum was recorded over an interval of 0–50 μs and three bands were observed (λ_{max} = 350, 526 and 750 nm, Figure S14left). A global analysis of the spectra using the Glotaran software (v 1.5.1)[19] led to the conclusion that three decay components with decay times of τ_1= 0.9 μs, τ_2= 3.3 μs and τ_3= 208 μs are present. The longer component of the three was found to be concentration dependent and disappeared when adopting more diluted solutions ($8 \cdot 10^{-5}$ M). As for τ_1 and τ_2, we found that the associated EADS (Evolution Associated Difference Spectra) are highly similar. We therefore propose that the triplet excited state of BP **I** partially undergoes triplet-triplet annihilation under our conditions. It is rather unlike that hydrogen abstraction from CH_3CN by BP **I** plays a role in our experiments, as the kinetic constant for this step has been reported to be several orders of magnitude slower (~10^2 M^{-1} s^{-1}).[20,21] Indeed, a benzene solution of BP **I** ($5 \cdot 10^{-4}$ M) measured in the same experimental conditions, showed a similar profile (with a slight red-shift of the band at 526 nm to 545 nm Figure S14), thus ruling out the possibility of interference by CH_3CN on the measurement.

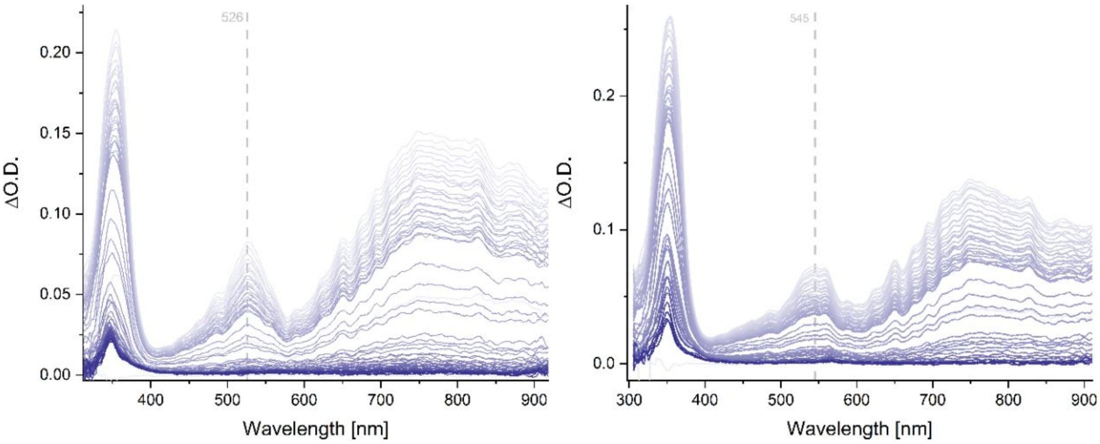

Figure S14 Decay of BP **I** ($5 \cdot 10^{-4}$ M) in CH_3CN (left) and benzene (right). Acquisition window: 0 – 50 ms. Acquisition intervals: (from 0 to 1 ms: 40 ns; from 1 to 16 ms: 500 ns; from 16 to 25 ms: 1 ms; from 25 to 50 ms: 5 ms).

The band at 526 nm is due to a triplet-triplet transition and resembles that observed for parent benzophenone.[22] Upon addition of an excess of $(TMS)_3SiH$ (8.6 equiv.), the overall intensity of the spectrum decreased and a new long-living feature appeared with a shoulder at 375 nm and a weak, broad band at 566 nm (Figure S15). We hypothesized that the species responsible for this new spectrum could be the ketyl radical generated upon HAT from $(TMS)_3SiH$ by BP **I**.[23] It is important to stress that $(TMS)_3SiH$ does not absorb light significantly at the excitation wavelength.[24] To prove this point, we performed the same experiment replacing $(TMS)_3SiH$ with THF (8.6 equiv.), a well-known quencher for BP **I** via HAT,[1] and obtained exactly the same profile (Figure S15).

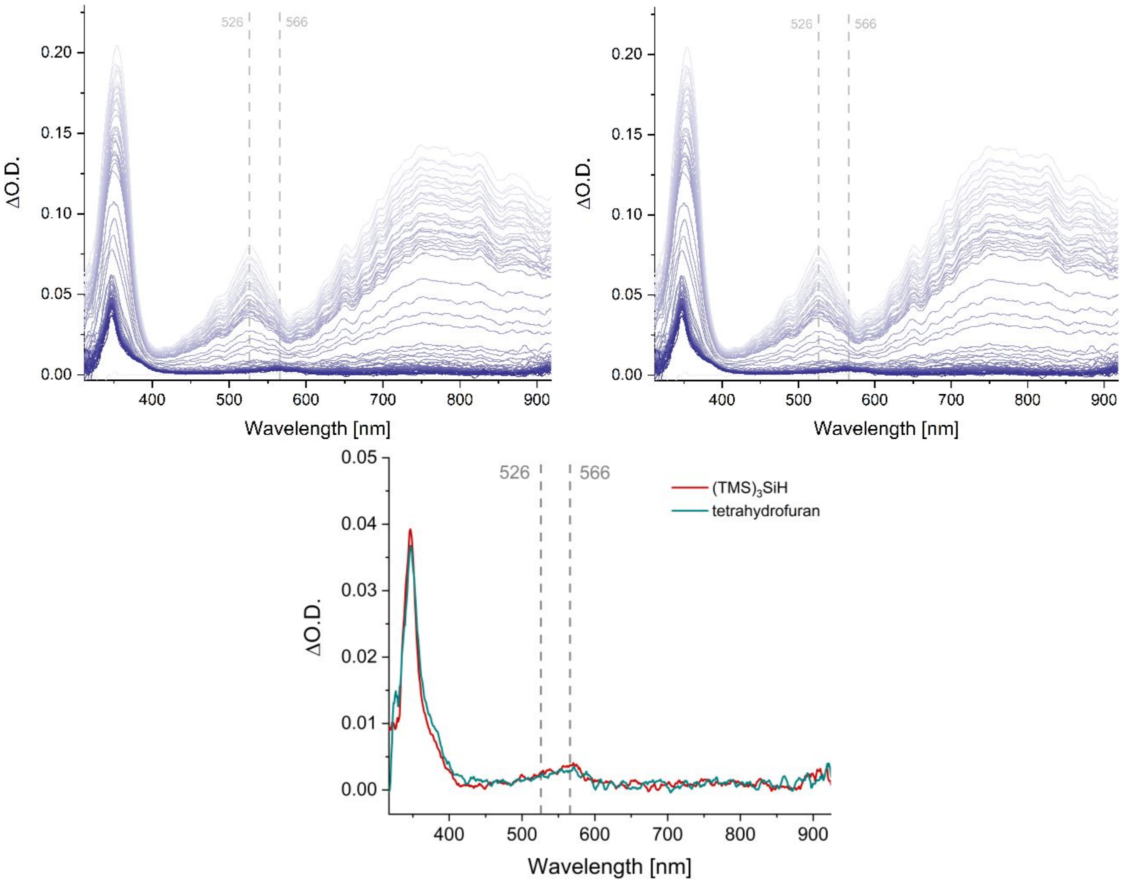

Figure S15: Decay of BP **I** ($5{\cdot}10^{-4}$ M) in CH_3CN in the presence of 8.6 equiv. of $(TMS)_3SiH$ (top left) and 8.6 equiv. of THF (top right). Acquisition window: 0–50 ms. Acquisition intervals: (from 0 to 1 ms: 40 ns; from 1 to 16 ms: 500 ns; from 16 to 25 ms: 1 ms; from 25 to 50 ms: 5 ms). A comparison of the results obtained upon addition of $(TMS)_3SiH$ and THF (8.6 equiv.) to a solution of BP **I** ($5{\cdot}10^{-4}$ M) in CH_3CN (t: 50 ms) is shown (bottom).

Given the above, we propose that $(TMS)_3SiH$ can quench the triplet excited state of BP **I** via HAT to deliver a ketyl radical.

Quantum Yield Measurements

Quantum yield (QY) was determined via ferrioxalate actinometry according to a procedure reported in the literature.[25]

Synthesis of the actinometer. Potassium ferrioxalate was prepared according to a procedure reported in the literature.[26] In particular, 3.2 g of $FeCl_3$ were dissolved in 8 mL of distilled H_2O and were added to a hot solution of 12 g of $K_2C_2O_4$ in 20 mL of distilled H_2O. After a couple of minutes at 100 °C, the mixture was let cooling down at room temperature and crystallization was triggered with a glass stick. After crystallization was complete, the mother liquor was removed via a Pasteur pipette and the green crystals ($K_3[Fe(C_2O_4)_3]$) were dissolved again in 20 mL of distilled H_2O. Potassium ferrioxalate was recrystallized two more times, washed with MeOH and dried at 45°C for 1 hour, light green crystals (5 g, 55%) were obtained. The salt was stored in the dark at −22 °C.

Actinometry. In a dark room equipped with a red light, a 0.15 M solution of ferrioxalate was prepared by dissolving 1.84 g of $K_3[Fe(C_2O_4)_3]*3H_2O$ in 25 mL of 0.05 M H_2SO_4. A buffered solution of phenanthroline was prepared by dissolving 50 mg of phenanthroline and 11.25 g of NaOAc in 50 mL of 0.5 M H_2SO_4.

Four 7-mL vials were charged with 3 mL of the ferrioxalate solution and irradiated with the same setup adopted for the batch experiments for the indicated time (see Table S10). 20 µL of the irradiated solutions were added to 2 mL of the phenanthroline solution (1:101 dilution); the resulting mixture was left equilibrating in the dark for 1 h. Next, the obtained solutions were further diluted by taking 50 µL and adding 3 mL of distilled water (1:61 dilution). The final solution was analyzed via UV-Vis spectroscopy.

Entry	t_{irr} (s)	$A_{510\ nm}$	mol Fe^{II}
Blank	0	0	0
1	3	3	$6.76\cdot10^{-6}$
2	6	6	$1.18\cdot10^{-5}$
3	9	9	$1.65\cdot10^{-5}$

Table S10: Results obtained for the ferrioxalate actinometry.

Given that

$$photon\ flux\ (F) = \frac{mol_{Fe^{2+}}}{t \times \Phi \times f} \quad \text{[Eq. S2]}$$

from which

$$mol_{Fe^{2+}} = (F \times \Phi \times f) \times t \quad \text{[Eq. S3]}$$

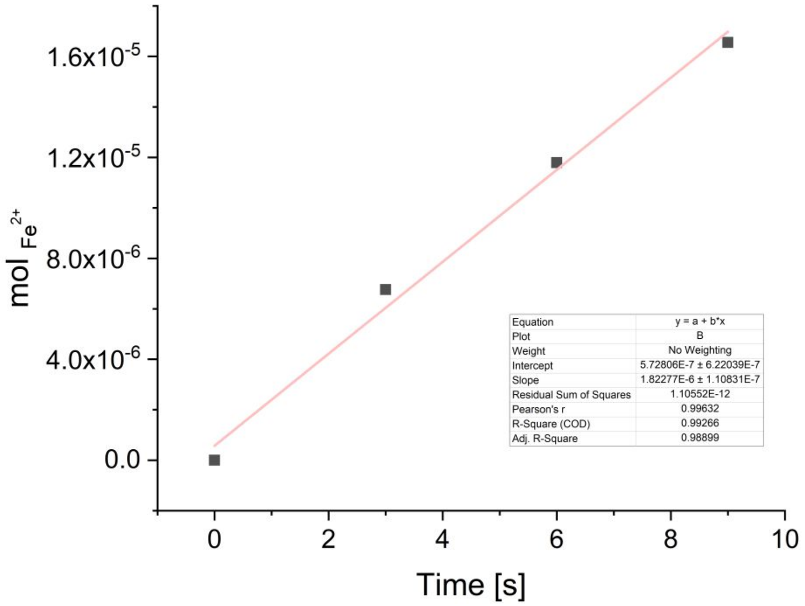

Figure S16 Plot of the moles of Fe^{2+} vs time (s) shows a linear correlation.

By dividing the slope ($1.82 \cdot 10^{-6}$) of the graph obtained by plotting data in Table S10, see Figure S16, by the quantum yield ($\Phi = 1.2$) and fraction of light absorbed by ferrioxalate at the considered wavelength ($f \sim 1$), a photon flux of $1.52 \cdot 10^{-6}$ E s^{-1} can be estimated.
Considering that the yield for compound **3** after 1 h in the same setup is 25%, the quantum yield for the reaction is calculated to be ~3%.

5.2 Computational Studies

General information

All the calculations were carried out using the Gaussian16 (Rev. C.01) program package[27] at the CINECA Supercomputer center (Italy). In our investigation, all the structures have been optimized in the gas phase by having recourse to density functional theory (DFT), *viz.* adopting the M06-2X functional with an unrestricted (U) formalism in the case of open-shell species, along with the 6-311++g(d,p) basis set. To confirm the nature of the optimized structures, vibrational frequencies have been calculated at the same level of theory as geometry optimizations, and it was verified that they had only real frequencies. For each of the reported structures, a systematic investigation of all of the possible conformations has been carried out. However, only the most stable conformation has been reported and considered for further work.
The solvent effect was included by single-point calculations on the optimized geometries obtained in the gas phase at the (U)M06-2X/6-311++g(d,p) level of theory in acetonitrile bulk, by maintaining the default solvent options of the PCM model.
A rigorous determination of the bond dissociation energies (BDEs) values for the relevant C–Br bonds (Figure S17a) calls for the use of high-level calculations with huge computational cost,[28] which is well beyond the aim of this work. As a matter of fact, DFT methods with unrestricted formalism may suffer from spin contamination, with the immediate consequence that the wavefunction is no longer an eigenfunction of the total spin. Accordingly, DFT approaches are unsuitable to address named objective, since some error may be introduced into the calculation.
A good trade-off consists in calculating the ΔG of the XAT step as a whole via equation shown in Figure S17b:[29] if negative, this would indicate a thermodynamically favored XAT process; if positive, on the contrary, an up-hill step.

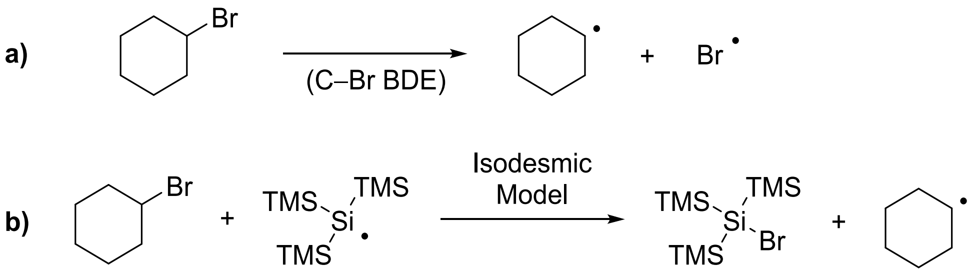

Figure S17 Computational models for the direct determination of the C–Br BDE value (a) and the corresponding isodesmic model (b).

DFT optimized geometries in the gas phase

In all cases, the structures reported below have been optimized at the (U)M06-2X/6-311++g(d,p) level of theory in the gas phase and it was verified that these had no imaginary frequencies.[18]

<table>
<tr><td rowspan="18">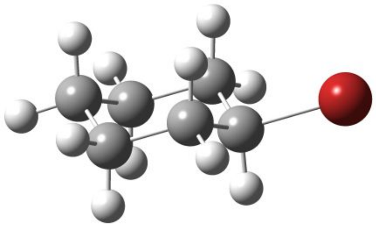
Bromocyclohexane
equatorial</td><td>C</td><td>2.13623700</td><td>-1.25948700</td><td>0.19684100</td></tr>
<tr><td>C</td><td>0.63952100</td><td>-1.26247200</td><td>-0.14103400</td></tr>
<tr><td>C</td><td>-0.01391700</td><td>0.00000000</td><td>0.40072100</td></tr>
<tr><td>C</td><td>0.63952100</td><td>1.26247200</td><td>-0.14103400</td></tr>
<tr><td>C</td><td>2.13623700</td><td>1.25948700</td><td>0.19684100</td></tr>
<tr><td>C</td><td>2.82101400</td><td>0.00000000</td><td>-0.33721300</td></tr>
<tr><td>H</td><td>0.50513700</td><td>-1.28946500</td><td>-1.22773700</td></tr>
<tr><td>H</td><td>0.14893200</td><td>-2.14613200</td><td>0.27142400</td></tr>
<tr><td>H</td><td>2.26250400</td><td>-1.30706100</td><td>1.28498200</td></tr>
<tr><td>H</td><td>2.60386200</td><td>-2.15701400</td><td>-0.21512700</td></tr>
<tr><td>H</td><td>0.50513700</td><td>1.28946400</td><td>-1.22773700</td></tr>
<tr><td>H</td><td>0.14893200</td><td>2.14613200</td><td>0.27142400</td></tr>
<tr><td>H</td><td>2.60386200</td><td>2.15701400</td><td>-0.21512700</td></tr>
<tr><td>H</td><td>2.26250400</td><td>1.30706100</td><td>1.28498200</td></tr>
<tr><td>H</td><td>2.77139300</td><td>0.00000000</td><td>-1.43249600</td></tr>
<tr><td>H</td><td>3.87966900</td><td>0.00000000</td><td>-0.06605200</td></tr>
<tr><td>H</td><td>0.00323200</td><td>0.00000000</td><td>1.49193200</td></tr>
<tr><td>Br</td><td>-1.93848100</td><td>0.00000000</td><td>-0.03632000</td></tr>
<tr><td rowspan="17">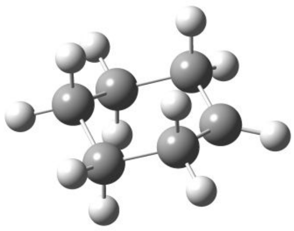
Cyclohexyl radical</td><td>C</td><td>-1.28127300</td><td>0.77258600</td><td>0.17020000</td></tr>
<tr><td>C</td><td>0.00000000</td><td>1.45360500</td><td>-0.18492400</td></tr>
<tr><td>C</td><td>1.28127300</td><td>0.77258600</td><td>0.17020000</td></tr>
<tr><td>C</td><td>1.26177000</td><td>-0.70677800</td><td>-0.24699100</td></tr>
<tr><td>C</td><td>0.00000000</td><td>-1.40346000</td><td>0.26712600</td></tr>
<tr><td>C</td><td>-1.26177000</td><td>-0.70677800</td><td>-0.24699100</td></tr>
<tr><td>H</td><td>2.13414600</td><td>1.28823500</td><td>-0.27724700</td></tr>
<tr><td>H</td><td>0.00000000</td><td>2.50578600</td><td>-0.44129300</td></tr>
<tr><td>H</td><td>-1.43110400</td><td>0.81498100</td><td>1.26303600</td></tr>
<tr><td>H</td><td>-2.13414600</td><td>1.28823500</td><td>-0.27724700</td></tr>
<tr><td>H</td><td>1.28403700</td><td>-0.76870400</td><td>-1.34072100</td></tr>
<tr><td>H</td><td>2.15721000</td><td>-1.21207900</td><td>0.12457800</td></tr>
<tr><td>H</td><td>0.00000000</td><td>-2.45439700</td><td>-0.03420100</td></tr>
<tr><td>H</td><td>0.00000000</td><td>-1.38681600</td><td>1.36447700</td></tr>
<tr><td>H</td><td>-1.28403700</td><td>-0.76870400</td><td>-1.34072100</td></tr>
<tr><td>H</td><td>-2.15721000</td><td>-1.21207900</td><td>0.12457800</td></tr>
<tr><td>H</td><td>1.43110400</td><td>0.81498100</td><td>1.26303600</td></tr>
</table>

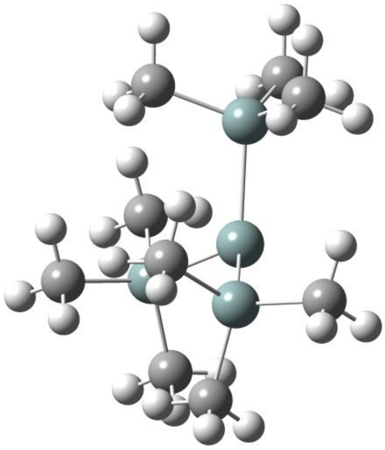

Tris(trimethylsilyl) radical

Si	-0.00014400	0.00042400	-0.68719700
Si	2.14220300	0.68802800	-0.05590600
Si	-1.66736300	1.51087100	-0.05618100
Si	-0.47512000	-2.19869200	-0.05608600
C	-1.03557700	3.27778800	-0.25062600
H	-0.16597000	3.45840100	0.38702700
H	-0.74386600	3.48192900	-1.28387900
H	-1.81144300	3.99632700	0.03010400
C	-2.10526700	1.21515600	1.75809800
H	-2.86149100	1.92981500	2.09765600
H	-2.50382300	0.20781900	1.90641700
H	-1.22356200	1.32286900	2.39557600
C	-3.21686800	1.28183700	-1.10417200
H	-4.00362700	1.97083400	-0.78261000
H	-3.00877500	1.47279900	-2.15955500
H	-3.60344400	0.26334700	-1.01759000
C	2.72107100	2.14084500	-1.10749400
H	2.03291600	2.98574400	-1.02459700
H	3.71103700	2.47785200	-0.78579800
H	2.78343400	1.86171800	-2.16192300
C	2.10493200	1.22001700	1.75707600
H	3.10255400	1.51553600	2.09668100
H	1.43432200	2.07130500	1.90288800
H	1.75453200	0.40526900	2.39645000
C	3.35533200	-0.74407200	-0.24642700
H	3.38687400	-1.10037500	-1.27912500
H	4.36560300	-0.43157200	0.03433200
H	3.07658800	-1.58660800	0.39226500
C	0.49814200	-3.42573500	-1.10440100
H	1.57342500	-3.25072100	-1.01829000
H	0.29550100	-4.45172400	-0.78286700
H	0.22828200	-3.34101800	-2.15967700
C	-2.32097900	-2.53584800	-0.25062000
H	-2.91313800	-1.87370900	0.38684400
H	-2.64342300	-2.38542600	-1.28397900
H	-2.55487600	-3.56722900	0.02981900
C	-0.00030900	-2.43079600	1.75829100
H	-0.24117500	-3.44317900	2.09739500
H	1.07129600	-2.27244800	1.90722400
H	-0.53469500	-1.72154100	2.39603300

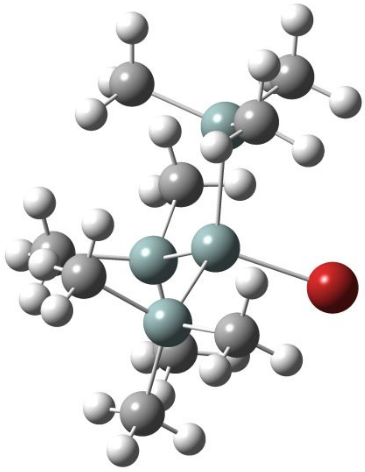

Tris(trimethylsilyl)bromide

Si	-0.00016700	-0.00017200	0.09822200
Si	1.71267600	-1.45414900	-0.56160800
Si	-2.11640400	-0.75599100	-0.56001100
Si	0.40268000	2.21049800	-0.56034200
C	-2.21744300	-2.62422800	-0.34262500
H	-1.50271200	-3.13824400	-0.99087900
H	-2.00085100	-2.90625900	0.69114200
H	-3.21838100	-2.98958300	-0.58999700
C	-2.33198300	-0.31054700	-2.38199100
H	-3.27844200	-0.70300800	-2.76520300
H	-2.33977100	0.77382800	-2.52310700
H	-1.52608200	-0.72421700	-2.99454200
C	-3.44246700	0.08375500	0.47468900
H	-4.43687500	-0.26601600	0.18251000
H	-3.29993700	-0.13677600	1.53520600
H	-3.41317900	1.16903800	0.35154700
C	1.64626000	-3.02446400	0.46997800
H	0.69146400	-3.54072700	0.34517500
H	2.44599600	-3.71105100	0.17747800
H	1.76520900	-2.79243800	1.53096700
C	1.43810700	-1.85896500	-2.38513200
H	2.25147500	-2.48223200	-2.76808000
H	0.50275500	-2.40661000	-2.53001200
H	1.39570700	-0.95254900	-2.99540600
C	3.38076500	-0.60800600	-0.33941200
H	3.51478600	-0.28136600	0.69522400
H	4.19822600	-1.29148000	-0.58672800
H	3.46936800	0.26926600	-0.98583200
C	1.79309800	2.93925700	0.47427400
H	2.71883700	2.37226900	0.35039000
H	1.98644400	3.97574500	0.18299100
H	1.53119700	2.92494600	1.53487500
C	-1.16490100	3.23156500	-0.34248900
H	-1.96764100	2.86876600	-0.99003500
H	-1.51674600	3.18547900	0.69152900
H	-0.98134500	4.28098400	-0.59066900
C	0.89567900	2.17417800	-2.38234400
H	1.02877100	3.19005000	-2.76569100
H	1.83869400	1.63878400	-2.52341100
H	0.13441100	1.68281100	-2.99453100
Br	0.00122700	-0.00078000	2.40079300

Calculated energies

The following table collects the calculated electronic energies (EE) in the gas phase (level of theory: (U)M06-/6-311++g(d,p)) corrected for the thermal free energy. Energy values in the condensed phase are given as the electronic energy (level of theory: PCM-(U)M06-/6-311++g(d,p), SCRF=acetonitrile) corrected for the thermal free energy obtained in the gas phase.

Structure	G, gas phase (Hartree)	G, liquid phase (Hartree)
Bromocyclohexane	-2809.267500	-2809.270882
Cyclohexyl radical	-235.024654	-235.025296
Tris(trimethylsilyl) radical	-1516.844321	-1516.846473
Tris(trimethylsilyl)bromide	-4091.112875	-4091.117360

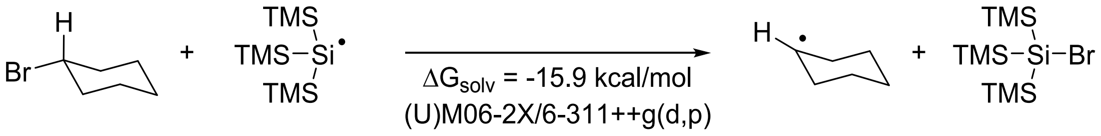

5.3 Experimental Studies

Evaluation of the intermediacy of bromine radical as HAT promoter

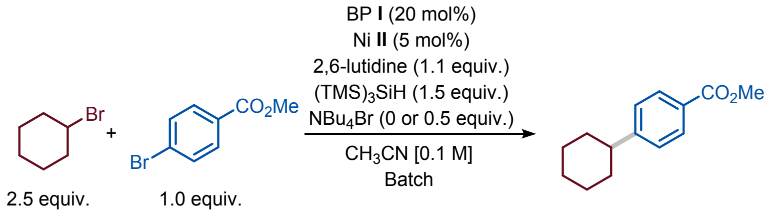

To a flame-dried argon-purged screw-capped vial, fitted with a rubber septum, charged with the nickel complex **II** (4.9 mg, 5 mol%), BP **I** (11.2 mg, 20 mol%), tetrabutylammonium bromide (0.5 or 0 equiv., 32 or 0 mg) and methyl 4-bromobenzoate (43 mg, 0.2 mmol, 1 equiv.) in CH_3CN (2 mL, 0.40 M), cyclohexyl bromide (62 µL, 0.5 mmol, 2.5 equiv.), 2,6-lutidine (25 µL, 0.22 mmol, 1.1 equiv.) and $(TMS)_3SiH$ (93 µL, 0.3 mmol, 1.5 equiv.) were added. Subsequently, the reaction vessel was placed in an ice bath, sparged with nitrogen for 30 seconds and the reaction vessel was sealed with parafilm. The vial was then placed in a 3D-printed photoreactor (see Figure S1) using a Kessil Lamp (390 nm, 100% intensity) as light source and irradiated for the stated amount of time. Subsequently, the acetonitrile was evaporated, the ensuing crude suspended in ethyl acetate, filtered on a short pad of silica and dried by rotary evaporation. An external standard was added (trichloroethylene, 18 µL, 1 equiv.) and the sample was analyzed by ^1H NMR spectroscopy.

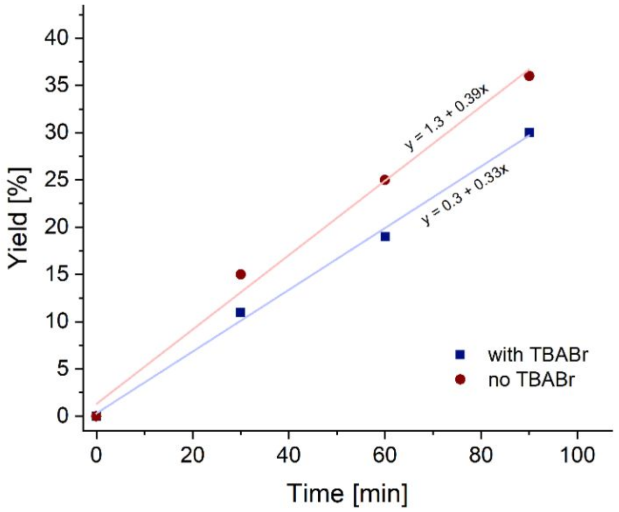

Figure S18. Yield in function of time in the presence/absence of TBABr.

As it is possible to see from the data plotted in Figure S18, no increase in the rate of the reaction is observed. In light of this, there seems not to be present any apparent contribution of bromine radicals to the observed reactivity.

Evaluation of the KIE

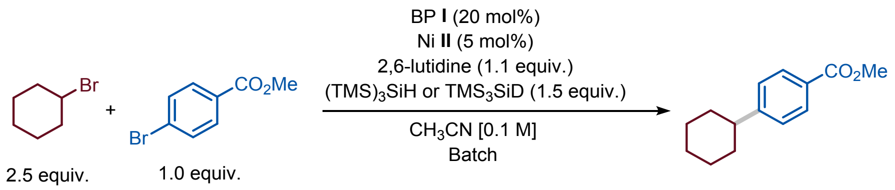

To a flame-dried argon-purged screw-capped vial, fitted with a rubber septum, charged with the nickel complex **II** (4.9 mg, 5 mol%), BP **I** (11.2 mg, 20 mol%), and methyl 4-bromobenzoate (43 mg, 0.2 mmol, 1 equiv.) in CH_3CN (2 mL, 0.40 M), cyclohexyl bromide (62 µL, 0.5 mmol, 2.5 equiv.), 2,6-lutidine (25 µL, 0.22 mmol, 1.1 equiv.) and $(TMS)_3SiH$ or $(TMS)_3SiD$ (93 µL, 0.3 mmol, 1.5 equiv.) were added. Subsequently, the reaction vessel was placed in an ice bath, sparged with nitrogen for 30 seconds and the reaction vessel was sealed with parafilm. The vial was then placed in a 3D-printed photoreactor (see Figure S1) using a Kessil Lamp (390 nm, 100% intensity) as light source and irradiated for the stated amount of time. Subsequently, the acetonitrile was evaporated, the ensuing crude suspended in ethyl acetate, filtered on a short pad of silica and dried by rotary evaporation. An external standard was added (trichloroethylene, 18 µL, 1 equiv.) and the sample was analyzed by ^{1}H NMR spectroscopy.

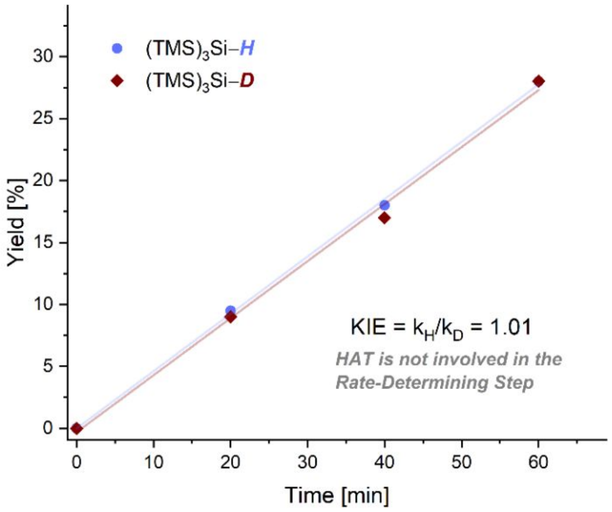

Figure S19. Kinetic Isotope Experiment to evaluate the rate of the reaction when using $(TMS)_3SiH$ or $(TMS)_3SiD$

As it is possible to see from the data plotted in Figure S19, no change in the rate of the reaction is observed when using $(TMS)_3SiH$ or $(TMS)_3SiD$. This suggests that the HAT is not involved in the rate limiting step.

Radical Clock Experiments

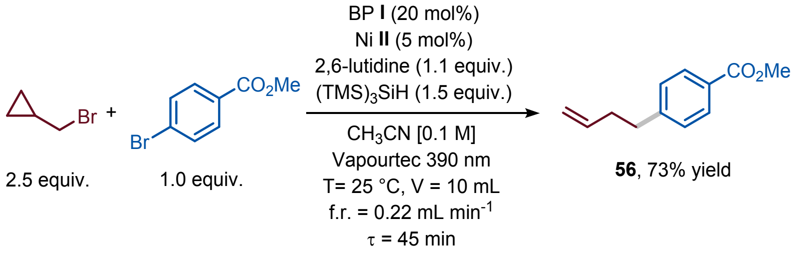

Prepared according to general procedure A, using (bromo-methyl)cyclopropane (121 µL, 1.25 mmol, 2.5 equiv.), methyl 4-bromobenzoate (108 mg, 0.5 mmol, 1 equiv). The crude mixture was purified by flash column chromatography (100% hexane to hexane 95:5 Et_2O, two consecutive runs) to afford product **56** (69 mg, 73% yield) as a colorless oil. The spectroscopic data are consistent with those reported previously.[30]

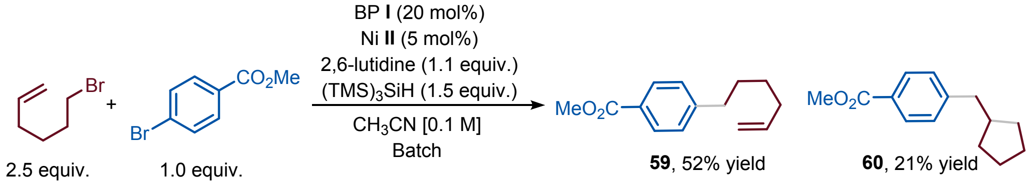

To a flame-dried argon-purged screw-capped vial, fitted with a rubber septum, charged with the nickel complex **II** (4.9 mg, 5 mol%), BP **I** (11.2 mg, 20 mol%) and methyl 4-bromobenzoate (43 mg, 0.2 mmol, 1 equiv.) in CH_3CN (2 mL, 0.40 M), 6-bromo-1-hexene (67 µL, 0.5 mmol, 2.5 equiv.), 2,6-lutidine (25 µL, 0.22 mmol, 1.1 equiv.) and $(TMS)_3SiH$ (93 µL, 0.3 mmol, 1.5 equiv.) were added. Subsequently, the reaction vessel was placed in an ice bath, sparged with nitrogen for 30 seconds and the reaction vessel was sealed with parafilm. The vial was then placed in a 3D-printed photoreactor (see Figure S1) using a Kessil Lamp (390 nm, 100% intensity) as light source and irradiated for 16 hours. Subsequently, the acetonitrile was evaporated, the ensuing crude suspended in ethyl acetate, filtered on a short pad of silica and dried by rotary evaporation. An external standard was added (trichloroethylene, 18 µL, 1 equiv.) and the sample was analyzed by ^{1}H NMR spectroscopy.

Evaluation of the kinetic constant for the alkyl radical interception

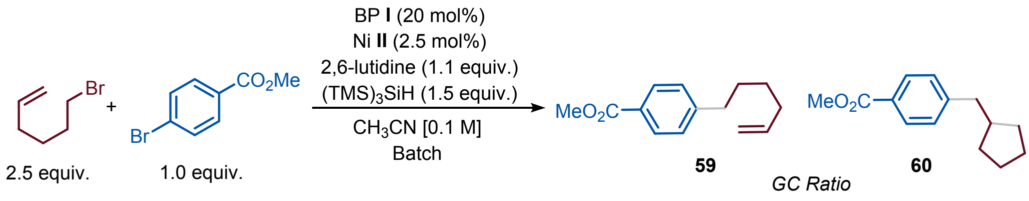

To a flame-dried argon-purged screw-capped vial, fitted with a rubber septum, charged with the nickel complex **II** (2.4 mg, 2.5 mol%), BP **I** (11.2 mg, 20 mol%) and methyl 4-bromobenzoate (43 mg, 0.2 mmol, 1 equiv.) in CH_3CN (2 mL, 0.40 M), 6-bromo-1-hexene (67 µL, 0.5 mmol, 2.5 equiv.), 2,6-lutidine (25 µL, 0.22 mmol, 1.1 equiv.) and $(TMS)_3SiH$ (93 µL, 0.3 mmol, 1.5 equiv.) were added. Subsequently, the reaction vessel was placed in an ice bath, sparged with nitrogen for 30 seconds and the reaction vessel was sealed with parafilm. The vial was then placed in a 3D-printed photoreactor (see Figure S1) using a Kessil Lamp (390 nm, 100% intensity) as light source and irradiated for 16 hours. Subsequently, the acetonitrile was evaporated, the ensuing crude suspended in ethyl acetate, filtered on a short pad of silica and dried by rotary evaporation. An external standard was added (trichloroethylene, 18 µL, 1 equiv.) and the sample was analyzed by ^{1}H NMR spectroscopy. Additionally, an aliquot of the crude was dissolved in dichloromethane and injected in GC-MS to further confirm the ratio between **59** and **60**, including the olefin isomers.

With these results in hand, we calculated the kinetic constant for the alkyl radical interception ($k_{capture}$). The kinetic constant for the cyclization (k_{Cycl}) of 5-hexenyl radical (Figure S20) is estimated from previous experiments in radical initiation and capture by Bu_3SnH.[31] At steady state, [**Ni**] is assumed to be 0.0025 M (catalyst loading). The distribution of **59** and **60** is based on GC measurements. The equation S4 below allows to estimate the kinetic constant for the alkyl radical interception (k_{Capt}).

$$k_{Capt} = \frac{k_{Cycl} \cdot [\mathbf{59}]}{[Ni]\quad[\mathbf{60}]} = \frac{1 \cdot 10^{5}\ s^{-1} \cdot 1.4}{0.0025\ M} = 5.6 \cdot 10^{7}\ M^{-1}s^{-1}$$

[Eq. S4]

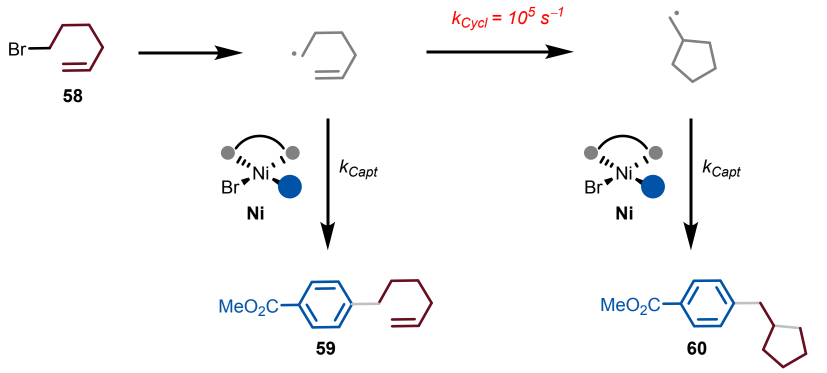

Figure S20. Mechanistic evaluation using 5-hexenyl radical as radical clock.

Effect of the conversion on the ratio between 59 and 60

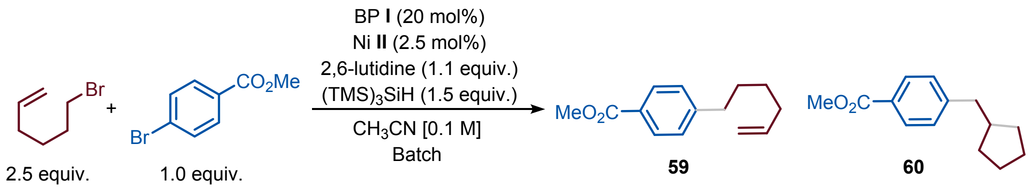

To a flame-dried argon-purged screw-capped vial, fitted with a rubber septum, charged with the nickel complex **II** (2.4 mg, 2.5 mol%), BP **I** (11.2 mg, 20 mol%) and methyl 4-bromobenzoate (43 mg, 0.2 mmol, 1 equiv.) in CH_3CN (2 mL, 0.40 M), 6-bromo-1-hexene (67 µL, 0.5 mmol, 2.5 equiv.), 2,6-lutidine (25 µL, 0.22 mmol, 1.1 equiv.) and $(TMS)_3SiH$ (93 µL, 0.3 mmol, 1.5 equiv.) were added. Subsequently, the reaction vessel was placed in an ice bath, sparged with nitrogen for 30 seconds and the reaction vessel was sealed with parafilm. The vial was then placed in a 3D-printed photoreactor (see Figure S1) using a Kessil Lamp (390 nm, 100% intensity) as light source and irradiated for the stated amount of time. Subsequently, the acetonitrile was evaporated, the ensuing crude suspended in ethyl acetate, filtered on a short pad of silica and injected in GC-MS to observe the ratio between **59** and **60**, including the olefin isomers (Figure S21).

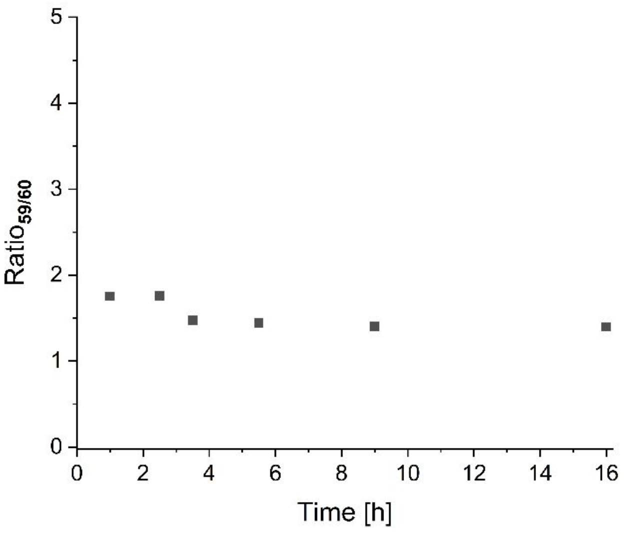

Figure S21. Ratio between compounds 59 and 60 followed over time.

As it is possible to see from the data plotted in Figure S21, and in agreement with previous results,[32] no change in the ratio of **59** vs **60** is observed when the conversion is higher than 50%.

Effect of the nickel loading on the ratio between 59 and 60

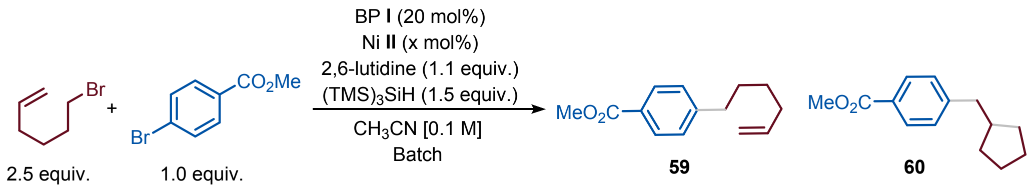

To a flame-dried argon-purged screw-capped vial, fitted with a rubber septum, charged with the nickel complex **II** (variable amounts: 0.5, 1, 2.5 and 5 mol%), BP **I** (11.2 mg, 20 mol%) and methyl 4-bromobenzoate (43 mg, 0.2 mmol, 1 equiv.) in CH_3CN (2 mL, 0.40 M), 6-bromo-1-hexene (67 µL, 0.5 mmol, 2.5 equiv.), 2,6-lutidine (25 µL, 0.22 mmol, 1.1 equiv.) and $(TMS)_3SiH$ (93 µL, 0.3 mmol, 1.5 equiv.) were added. Subsequently, the reaction vessel was placed in an ice bath, sparged with nitrogen for 30 seconds and the reaction vessel was sealed with parafilm. The vial was then placed in a 3D-printed photoreactor (see Figure S1) using a Kessil Lamp (390 nm, 100% intensity) as light source and irradiated for 16 hours. Subsequently, the acetonitrile was evaporated, the ensuing crude suspended in ethyl acetate, filtered on a short pad of silica and injected in GC-MS to observe the ratio between **59** and **60**, including the olefin isomers (Figure S22).

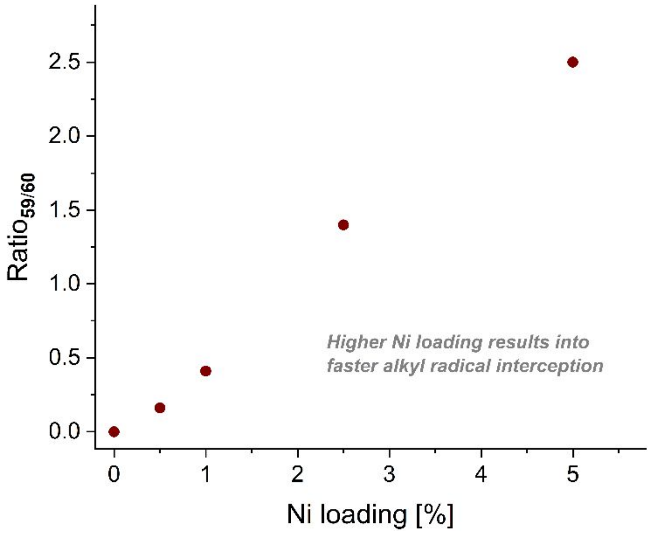

Figure S22. Evaluation of the influence of the nickel loading on the ratio of uncyclized and cyclized products.

As it is possible to see from the data plotted in Figure S22, the lower the amount of nickel catalyst, the higher the yield of **60**. Conversely, the higher the amount of nickel catalyst, the higher the amount of **59**. This is in agreement with a scenario where higher nickel loading results into a faster alkyl radical interception.

Effect of the nature of the aryl bromide on the ratio between 59 and 60

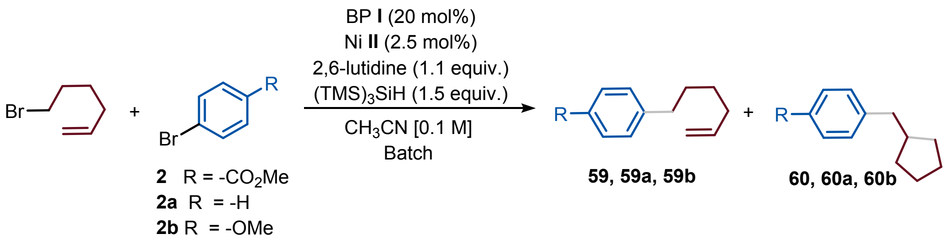

To a flame-dried argon-purged screw-capped vial, fitted with a rubber septum, charged with the nickel complex **II** (2.4 mg, 2.5 mol%), BP **I** (11.2 mg, 20 mol%), and either methyl 4-bromobenzoate (43 mg, 0.2 mmol, 1 equiv.), bromobenzene (21 µL, 1 equiv.) or 4-bromoanisole (25 µL, 1 equiv.) in CH_3CN (2 mL, 0.40 M), 6-bromo-1-hexene (67 µL, 0.5 mmol, 2.5 equiv.), 2,6-lutidine (25 µL, 0.22 mmol, 1.1 equiv.) and $(TMS)_3SiH$ (93 µL, 0.3 mmol, 1.5 equiv.) were added. Subsequently, the reaction vessel was placed in an ice bath, sparged with nitrogen for 30 seconds and the reaction vessel was sealed with parafilm. The vial was then placed in a 3D-printed photoreactor (see Figure S1) using a Kessil Lamp (390 nm, 100% intensity) as light source and irradiated for 16 hours. Subsequently, the acetonitrile was evaporated, the ensuing crude suspended in ethyl acetate, filtered on a short pad of silica and injected in GC-MS to observe the ratio between **59** and **60**, including the olefin isomers (Figure S23).

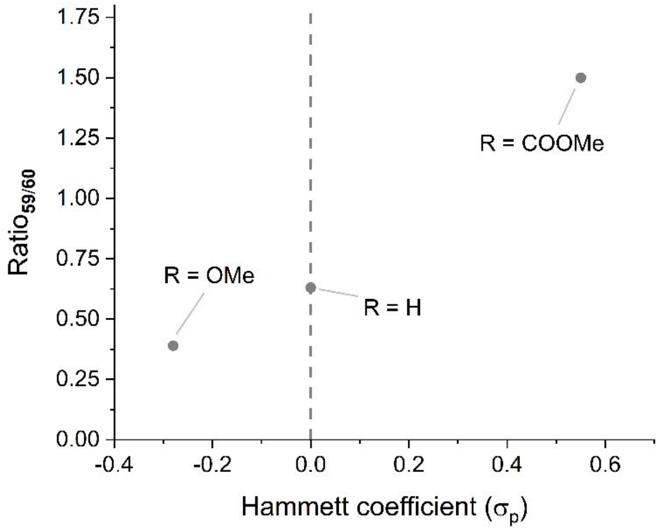

Figure S23. Hammett coefficient evaluation.

As it is possible to see from the data plotted in Figure S23, the ratio between **59** and **60** is heavily affected by the electronic nature of the parent aryl bromide. In detail, the more electron-rich is the aromatic ring, the higher the yield of **60**. Conversely, the more electron-poor is the aromatic ring, the higher the yield of **59**. This is in agreement with a scenario where the radical interception happens after the oxidative addition, thus the electrophilicity of the nickel complex is affected by the nature or the aromatic ring.

6. References

1) Y. Shen, Y. Gu, and R. Martin *J. Am. Chem. Soc.*, **2018**, *140*, 12200–12209.
2) I. B. Perry, T. F. Brewer, P. J. Sarver, D. M. Schultz, D. A. DiRocco, D. W. C. MacMillan *Nature* **2018**, *560*, 70–75.
3) A. Xia, X. Xie, X. Hu, W. Xu, and Y. Liu *J. Org. Chem.* **2019**, 84, *21*, 13841–13857.
4) S. M. Hell, C. F. Meyer, G. Laudadio, A. Misale, M. C. Willis, T. Noël, A. A. Trabanco, V. Gouverneur *J. Am. Chem. Soc.* **2020**, *142*, 720–725.
5) D. Liu; Y. Li; Chao; X. Qi, C. Liu, Y. Lan, A. Lei *Org. Lett.*, **2015**, *17*, 998–1001.
6) D. N. Primer, I. Karakaya, J. C. Tellis, G. A. Molander *J. Am. Chem. Soc.* **2015**, *137*, 2195−2198.
7) S. Ito, Y. Fujiwara, E. Nakamura, M. Nakamura *Org. Lett.* **2009**, *11*, 4306–4309.
8) Q. Jiang, A. Zhao, B. Xu, J. Jia, X. Liu, C. Guo *J. Org. Chem.* **2014**, *79*, 2709–2715.
9) P. Zhang, C. Le, D. W. C. MacMillan *J. Am. Chem. Soc.* **2016**, *138*, 8084−8087.
10) G. Li, D. Leow, L. Wan, J. Q. Yu *Angew. Chem. Int. Ed.* **2012**, *52*, 1245–1247.
11) X. Zhang, D. W. C. MacMillan *J. Am. Chem. Soc.* **2016**, 138, 13862–13865.
12) B. Sun, S. Zheng, F. Mo *Chem. Commun.*, **2021**, *57*, 5674–5677.
13) X. Wang, P. Cui, C. Xia, L. Wu *Angew. Chem. Int. Ed.* **2021**, *60*, 12298–12303.
14) V. K. Pandey, C. S. Tiwari, A. Rit *Org. Lett.* **2021**, *23*, 1681–1686.
15) A. García-Domínguez, R. Mondal, C. Nevado *Angew. Chem. Int. Ed.* **2019**, *58*, 12286–12290.
16) S.-Z. Sun, Y. Duan, R. S. Mega, R. J. Somerville, R. Martin *Angew. Chem. Int. Ed.* **2020**, *59*, 4370–4374.
17) S. Aloïse, C. Ruckebusch, L. Blanchet, J. Réhault, G. Buntinx, J.-P. Huvenne *J. Phys. Chem. A,* **2008**, *112*, 224–231.
18) G. H. Lovett, S. Chen, X. S. Xue, K. N. Houk, D. W. C. MacMillan *J. Am. Chem. Soc.,* **2019**, *141*, 20031–20036.
19) a) J. Snellenburg, S. Laptenok, R. Seger, K. M. Mullen, I. H. M. Van Stokkum, *J. Stat. Softw.* **2012**, *49*, 1–22; b) K. M. Mullen, I. H. M. Van Stokkum, *J. Stat. Softw.* **2007**, *18*, 1–46.
20) Y. M. A. Naguib, C. Steel, S. G. Cohen, M. A. Young *J. Phys. Chem.* **1987**, *91*, 3033–3036.
21) J. Chilton, L. Giering, C. Steel *J. Am. Chem. Soc.* **1976**, 98, 1865–1870.
22) T.S. Godfrey, J.W. Hilpern, G. Porter, *Chem. Phys. Lett.* **1967**, *1*, 490–492.
23) M. Sakamoto, X. Cai, M. Hara, S. Tojo, M. Fujitsuka, T. Majima *J. Phys. Chem. A* **2004**, *108,* 8147–8150.
24) J. Lalevée, X. Allonas, J. P. Fouassier *J. Org. Chem.* **2007**, *72*, 6434–6439.
25) M. A. Cismesia, T. P. Yoon *Chem. Sci.* **2015**, *6*, 5426–5434.
26) R. C. Jonhson *J. Chem. Educ.* **1970**, *47*, 702.
27) Gaussian 16, Revision C.01, M. J. Frisch, G. W. Trucks, H. B. Schlegel, G. E. Scuseria, M. A. Robb, J. R. Cheeseman, G. Scalmani, V. Barone, G. A. Petersson, H. Nakatsuji, X. Li, M. Caricato, A. V. Marenich, J. Bloino, B. G. Janesko, R. Gomperts, B. Mennucci, H. P. Hratchian, J. V. Ortiz, A. F. Izmaylov, J. L. Sonnenberg, D. Williams-Young, F. Ding, F. Lipparini, F. Egidi, J. Goings, B. Peng, A. Petrone, T. Henderson, D. Ranasinghe, V. G. Zakrzewski, J. Gao, N. Rega, G. Zheng, W. Liang, M. Hada, M. Ehara, K. Toyota, R. Fukuda, J. Hasegawa, M. Ishida, T. Nakajima, Y. Honda, O. Kitao, H. Nakai, T. Vreven, K. Throssell, J. A. Montgomery, Jr., J. E. Peralta, F. Ogliaro, M. J. Bearpark, J. J. Heyd, E. N. Brothers, K. N. Kudin, V. N. Staroverov, T. A. Keith, R. Kobayashi, J. Normand, K. Raghavachari, A. P. Rendell, J. C. Burant, S. S. Iyengar, J. Tomasi, M. Cossi, J. M. Millam, M. Klene, C. Adamo, R. Cammi, J. W. Ochterski, R. L. Martin, K. Morokuma, O. Farkas, J. B. Foresman, and D. J. Fox, Gaussian, Inc., Wallingford CT, **2016**.
28) B. Chan, E. Collins, K. Raghavachari, *WIREs Comput. Mol. Sci.* **2021**, *11*, e1501.
29) B. Chan, L. Radom, *J. Phys. Chem. A* **2012**, *116*, 4975–4986.

30) Q.-Y. Meng, T. E. Schirmer, K. Katou, B. König, *Angew. Chem. Int. Ed.* **2019**, 58, 5723–5728.
31) D.J. Carlsson, K. U. Ingold *J. Am. Chem. Soc.* **1968**, *90*, 7047–7055.
32) S. Biswas D. J. Weix *J. Am. Chem. Soc.* **2013**, 135, 43, 16192–16197.

7. NMR Spectra

3, ^{1}H NMR (400 MHz, $CDCl_3$)

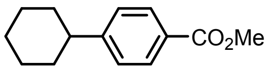

3

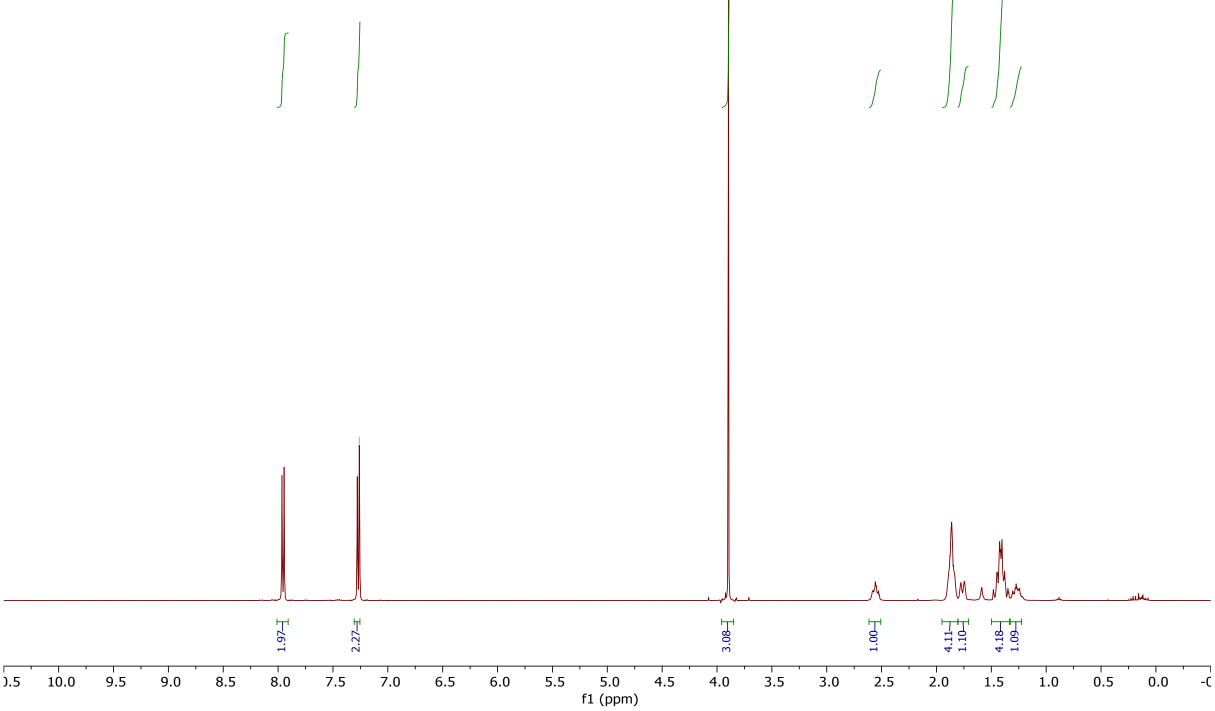

3, ^{13}C NMR (101 MHz, $CDCl_3$)

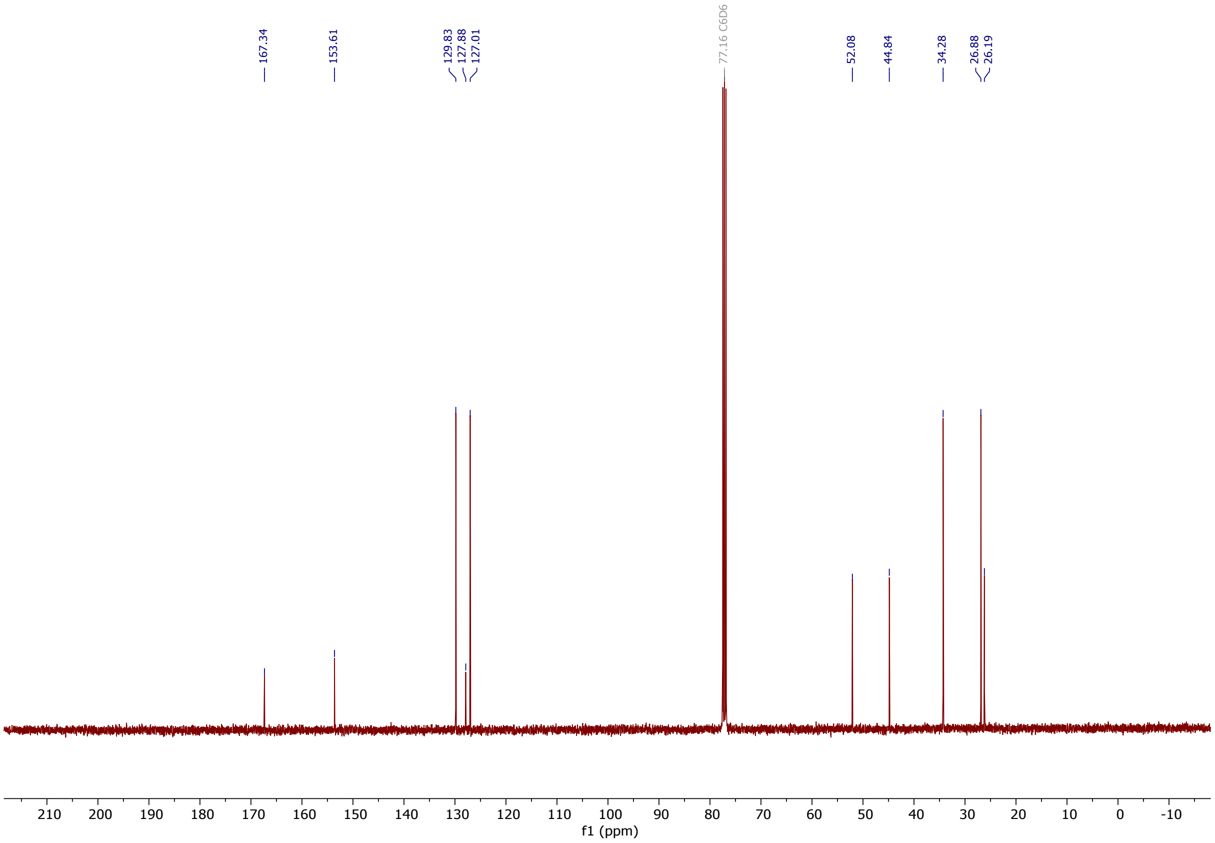

4, ^{1}H NMR (400 MHz, $CDCl_3$)

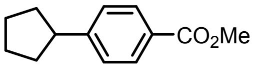

4

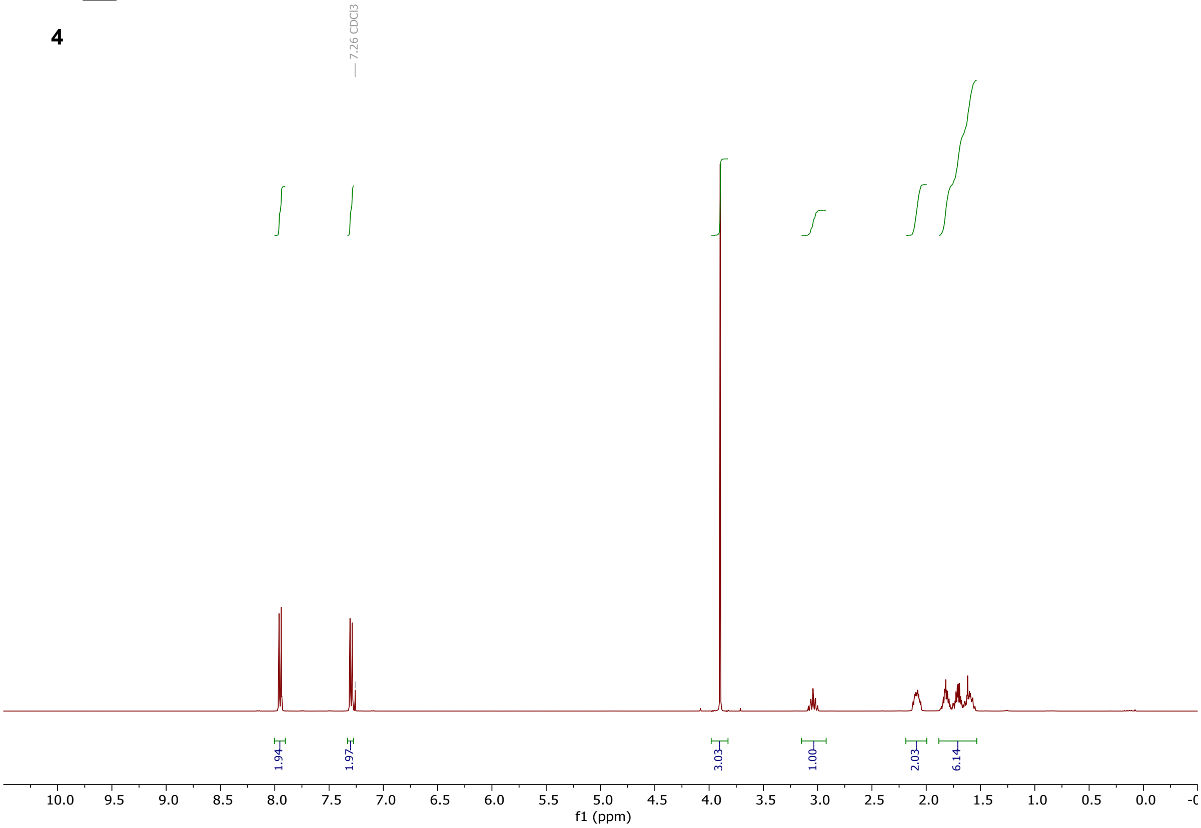

4, ^{13}C NMR (101 MHz, $CDCl_3$)

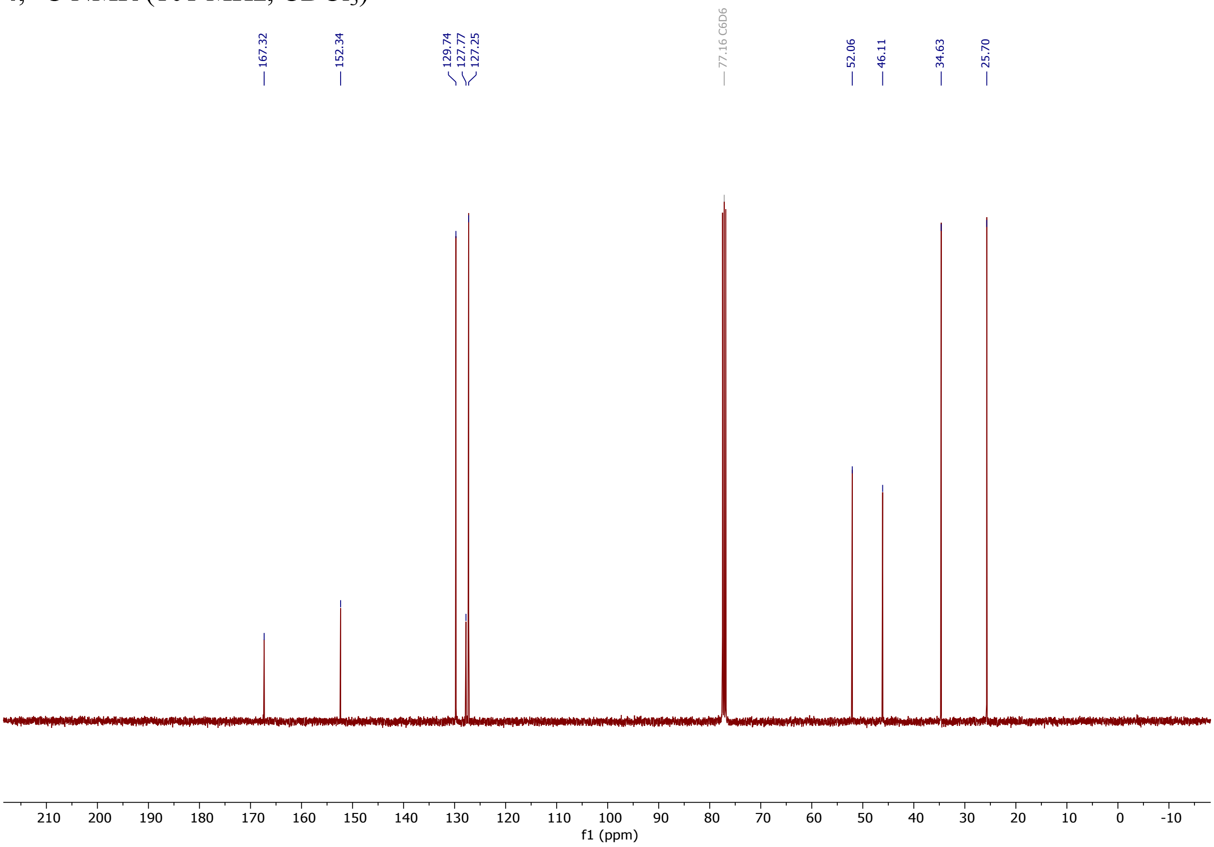

5, ^{1}H NMR (300 MHz, $CDCl_3$)

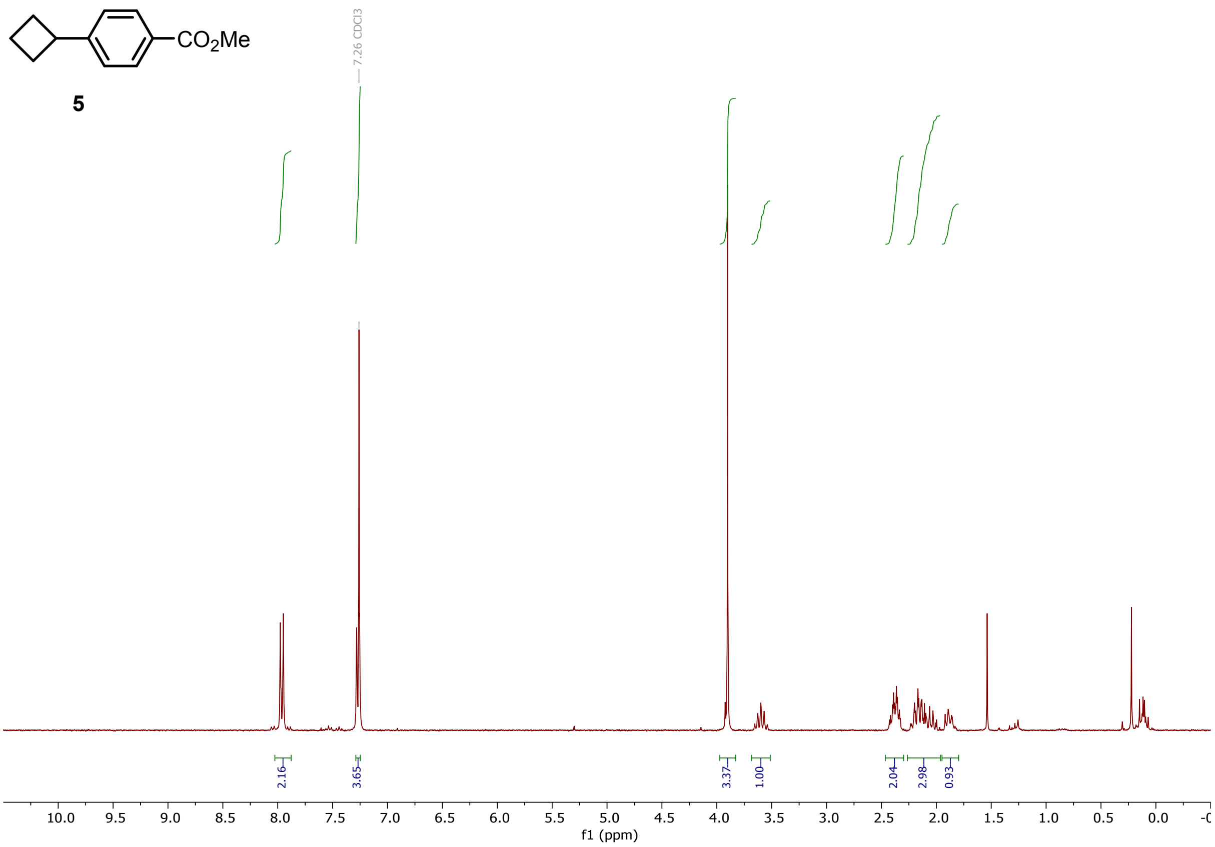

5, ^{13}C NMR (101 MHz, $CDCl_3$)

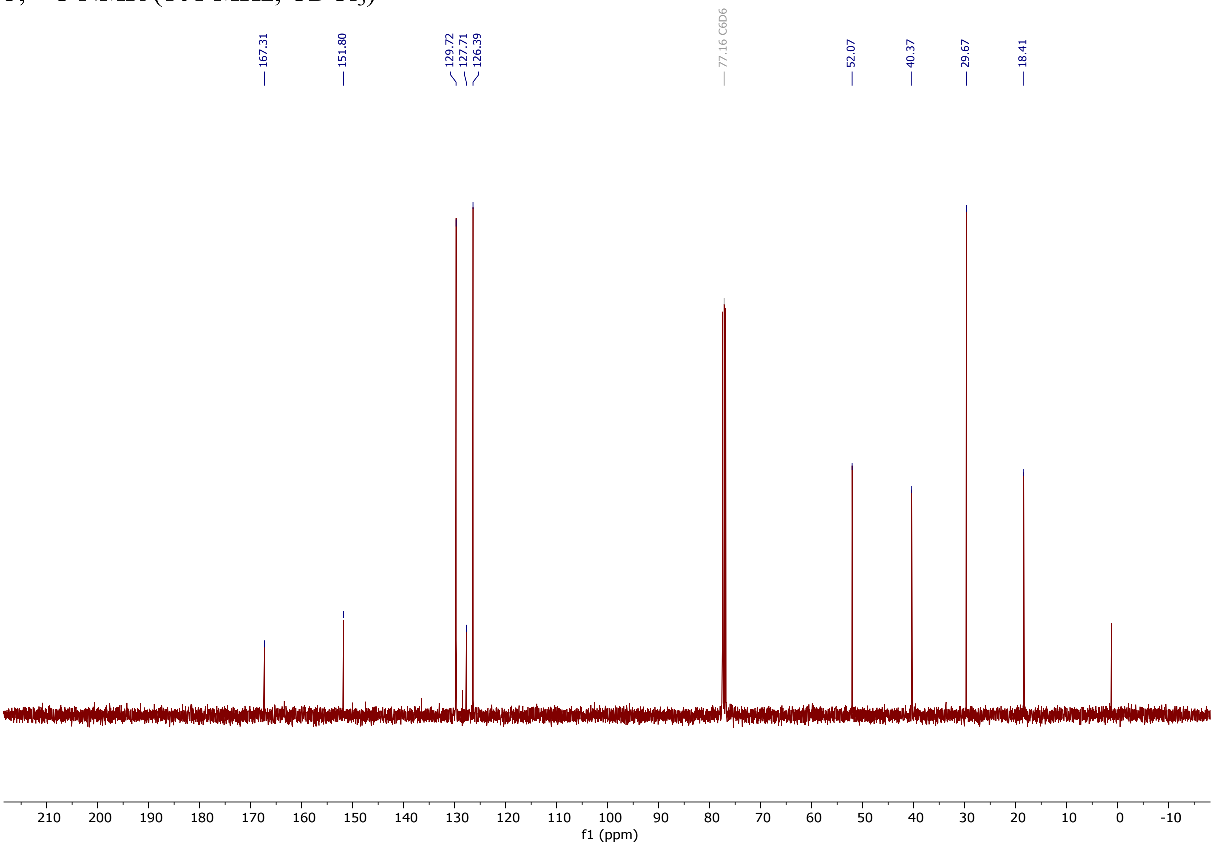

6, ^{1}H NMR (300 MHz, $CDCl_3$)

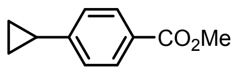

6

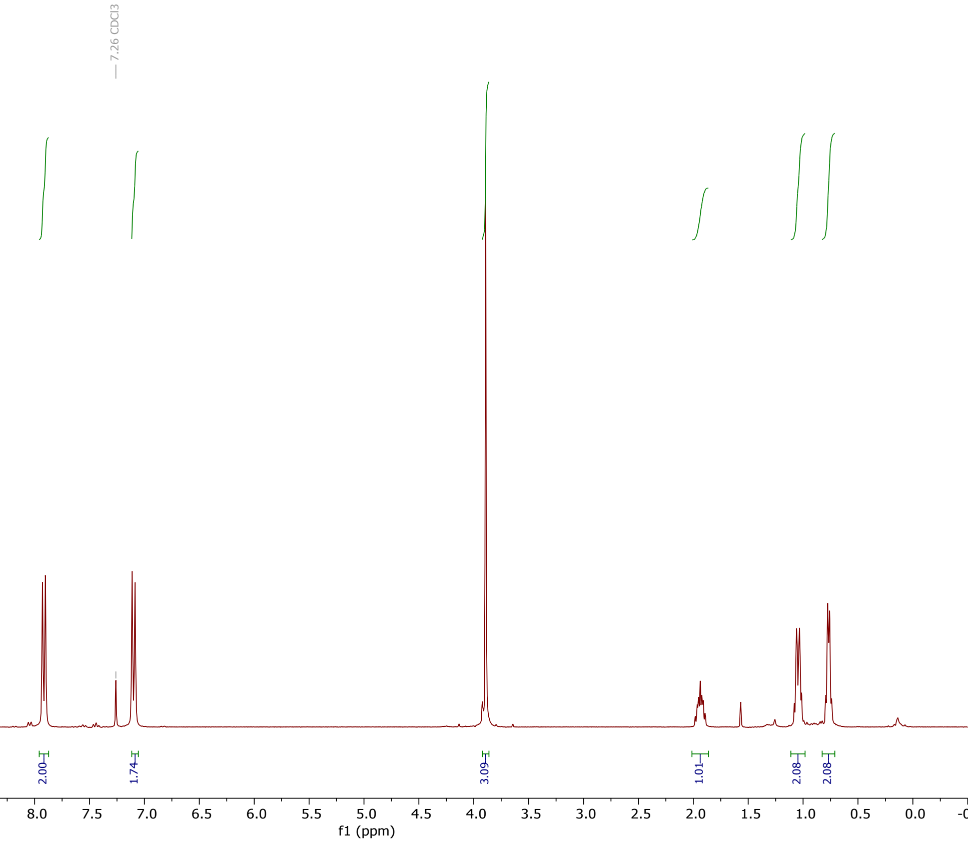

6, ^{13}C NMR (75 MHz, $CDCl_3$)

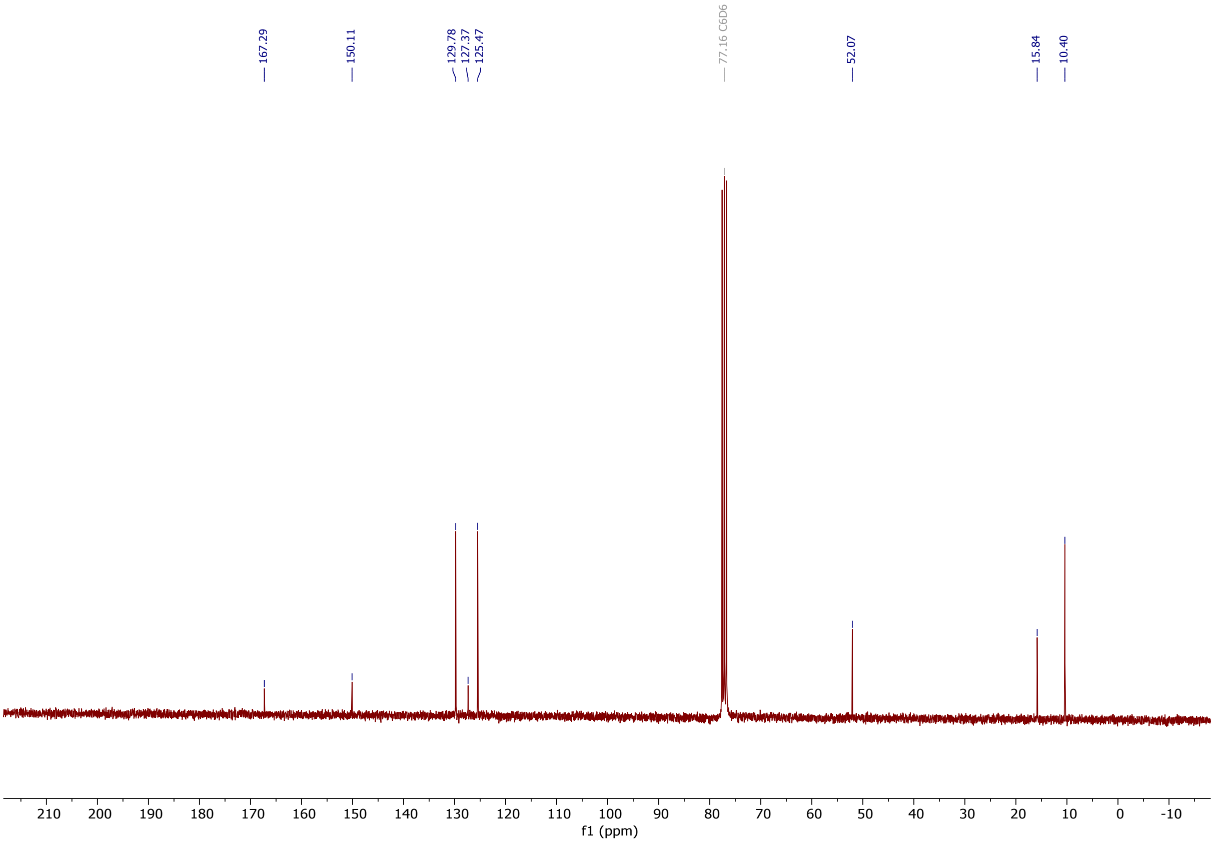

7, ^{1}H NMR (300 MHz, $CDCl_3$)

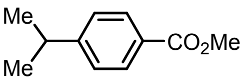

7

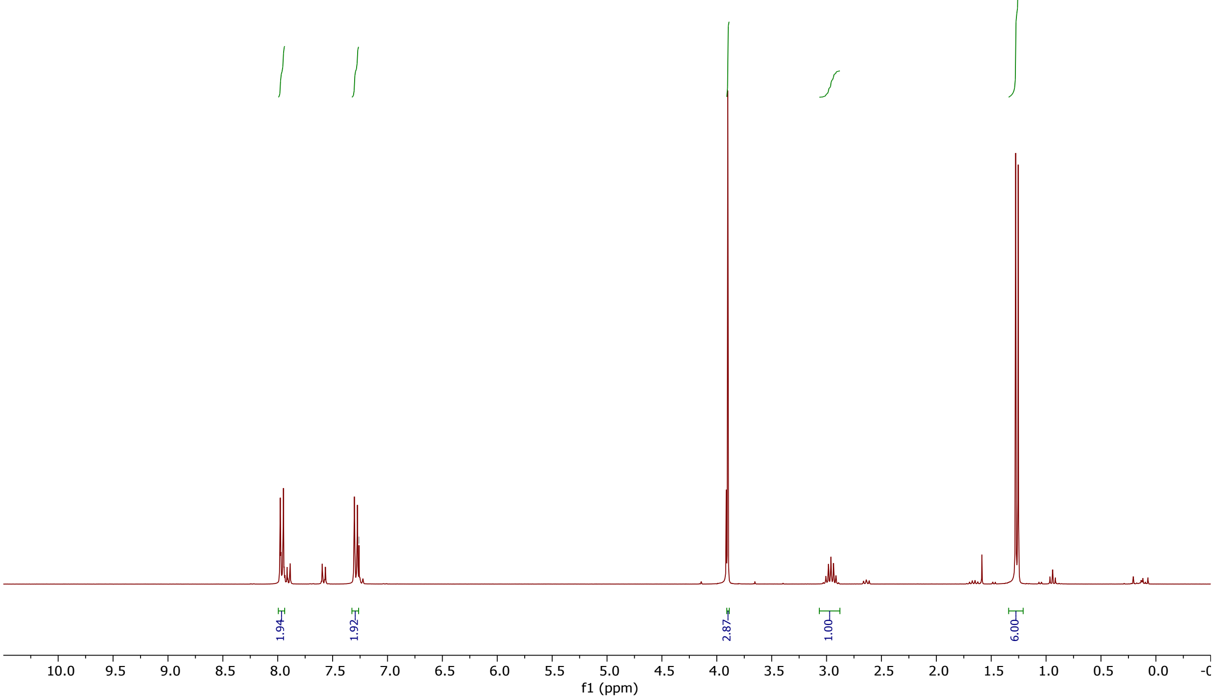

7, ^{13}C NMR (75 MHz, $CDCl_3$)

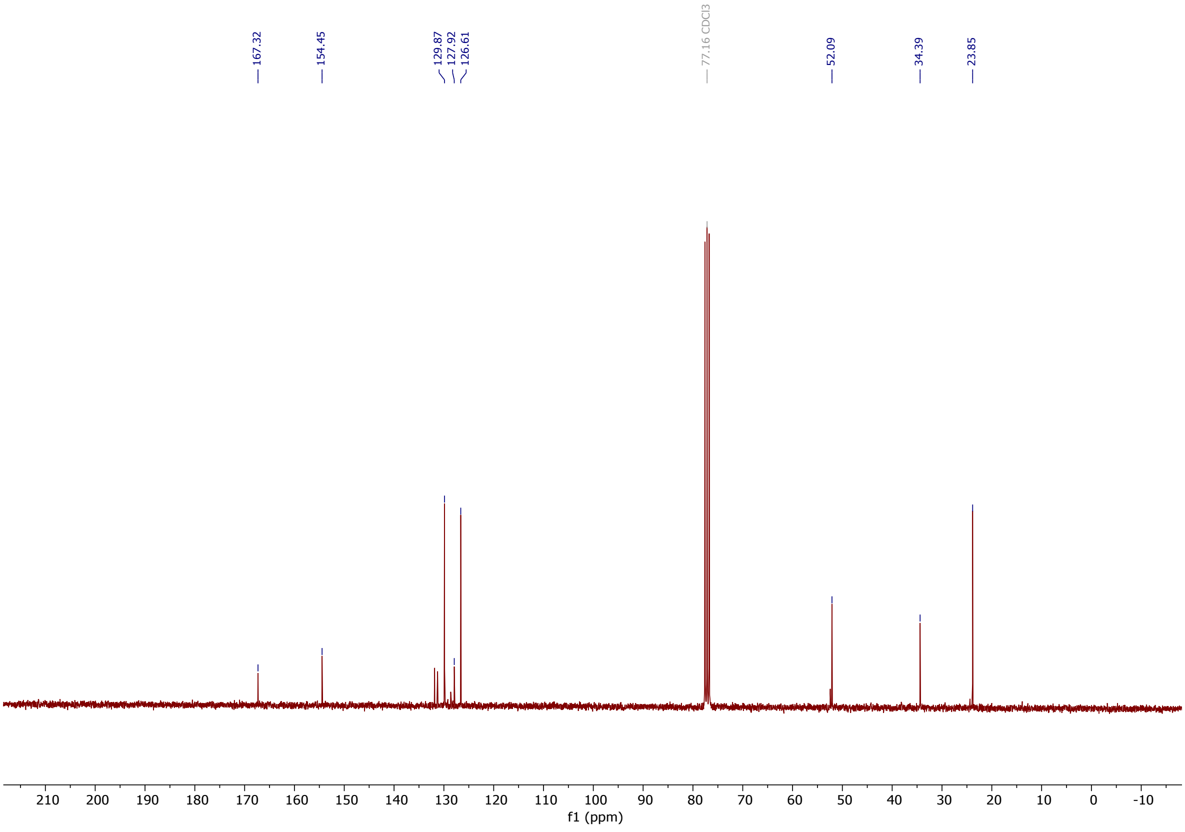

8, ^{1}H NMR (300 MHz, $CDCl_3$)

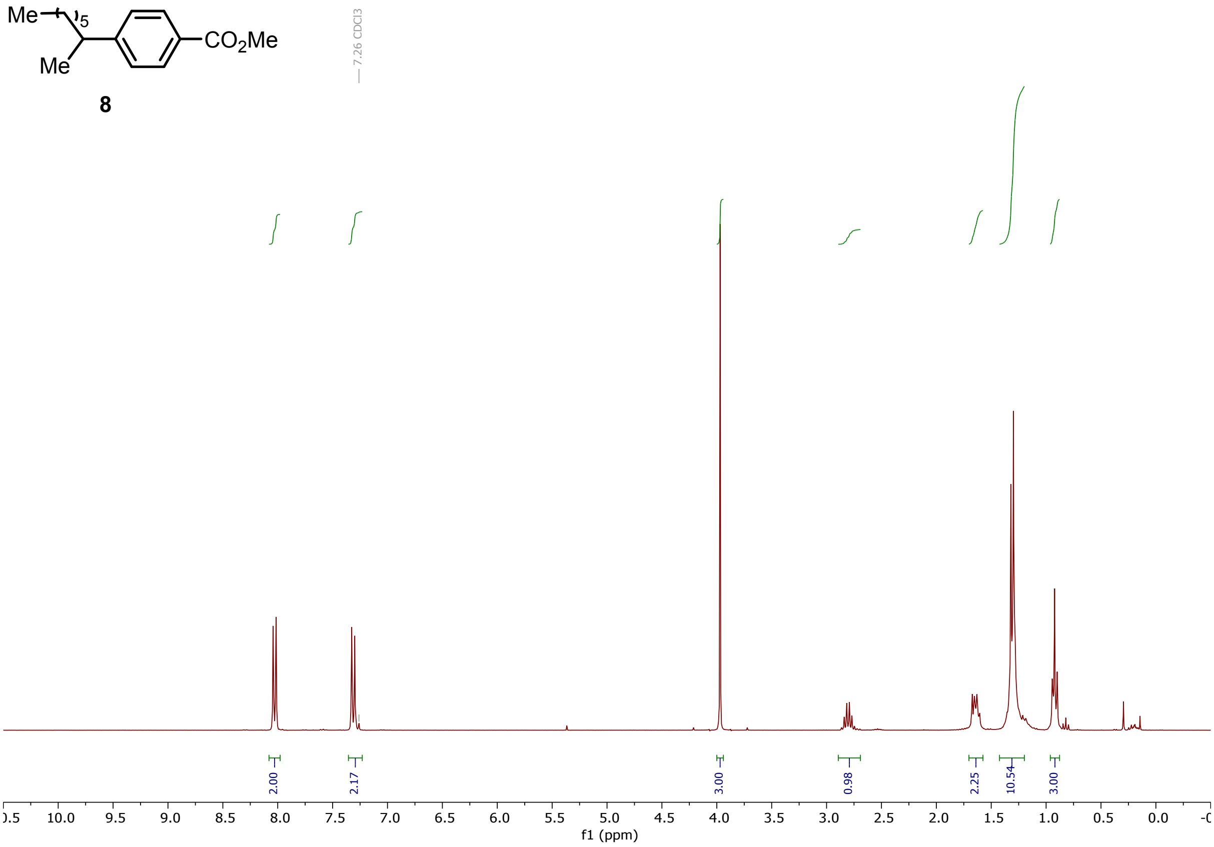

8, ^{13}C NMR (75 MHz, $CDCl_3$)

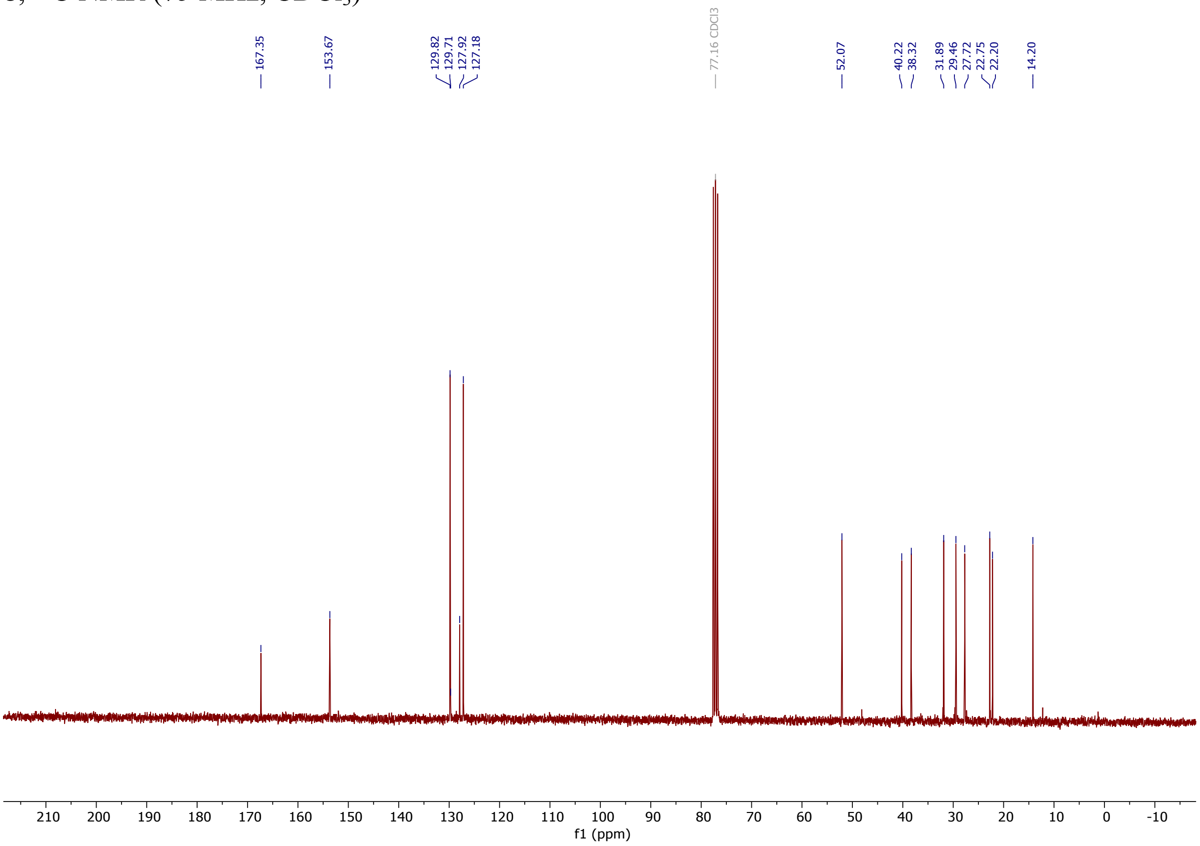

9, ^{1}H NMR (300 MHz, $CDCl_3$)

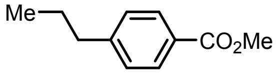

9

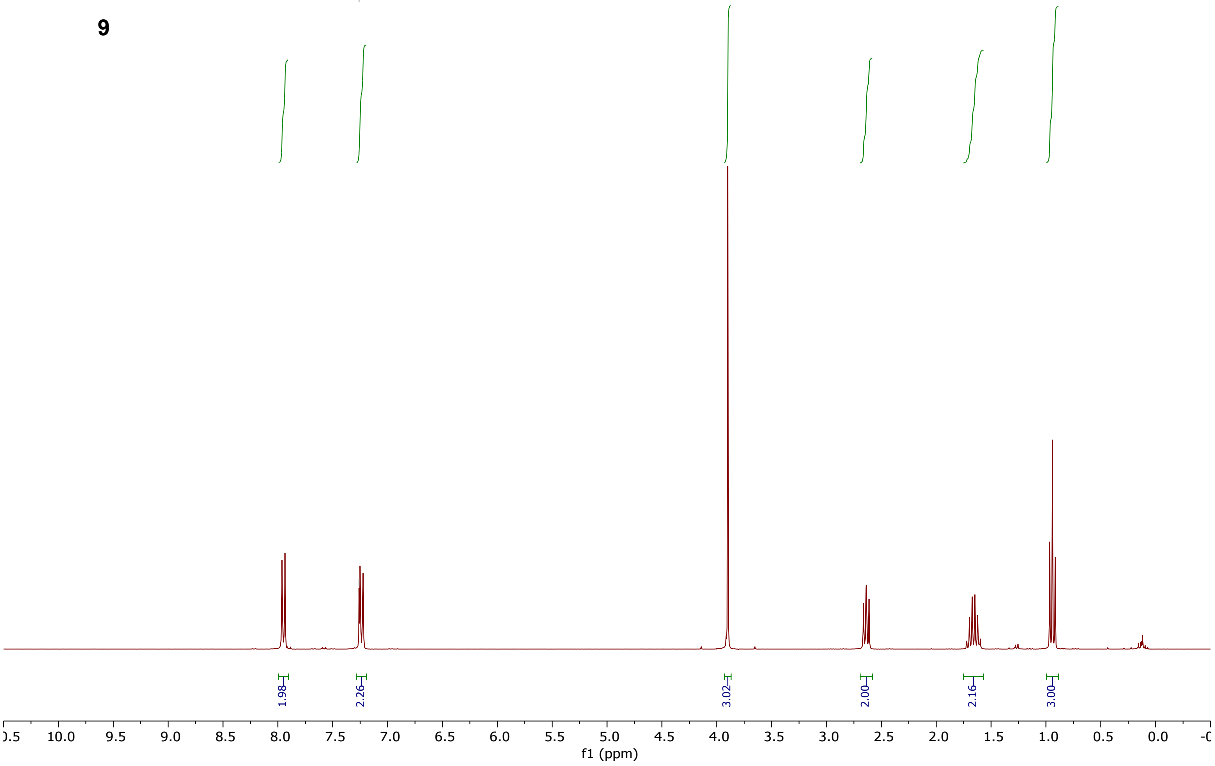

9, ^{13}C NMR (75 MHz, $CDCl_3$)

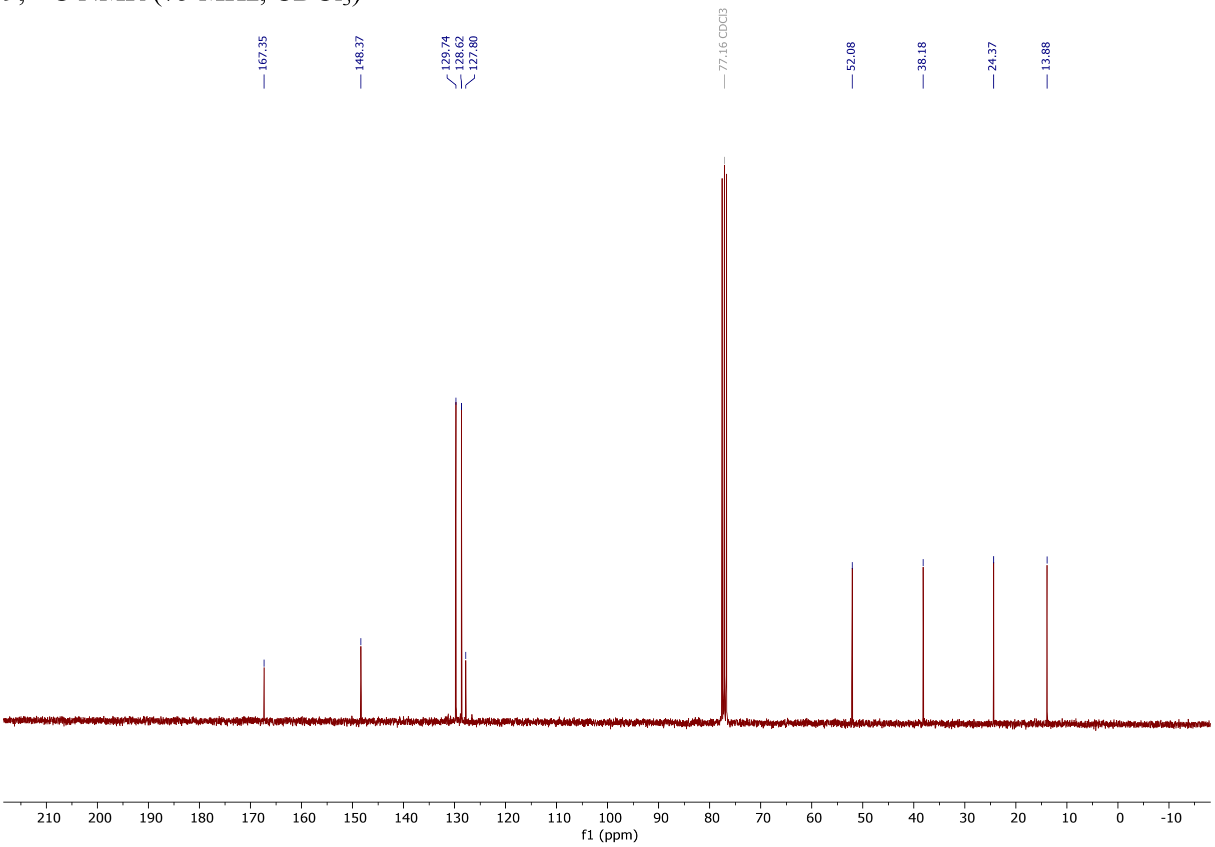

10, ^{1}H NMR (400 MHz, $CDCl_3$)

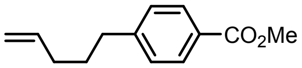

10

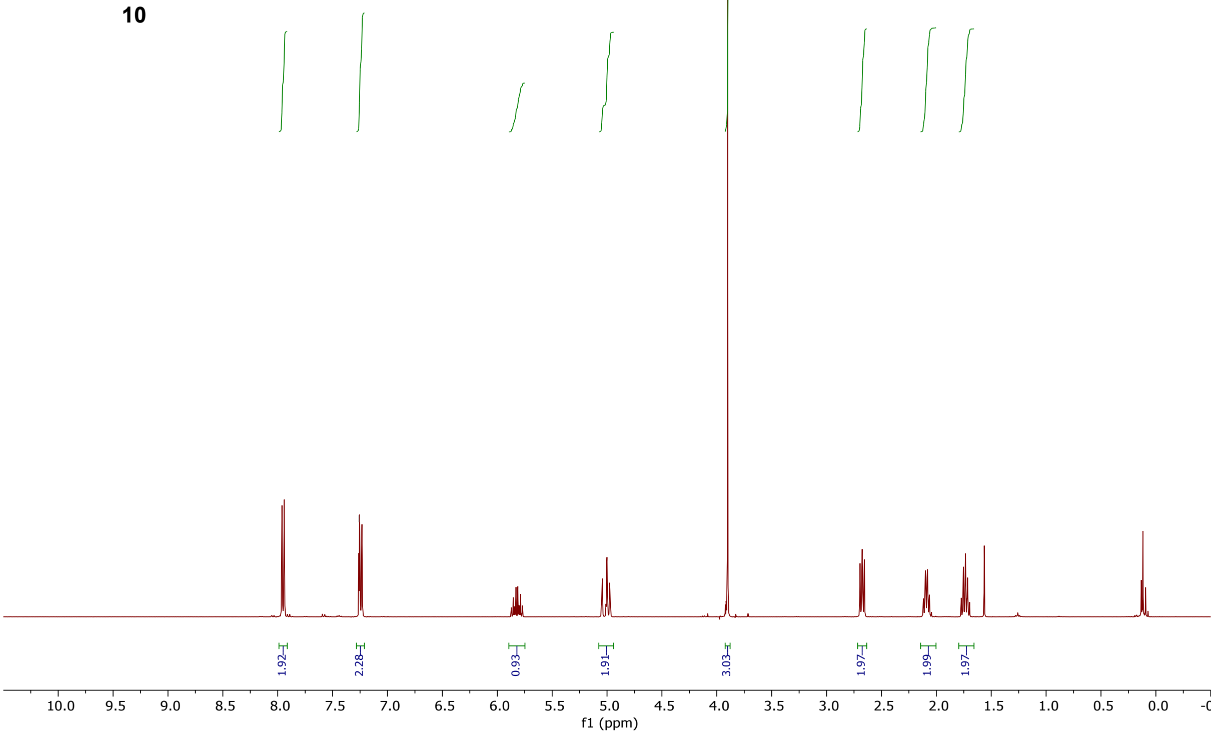

10, ^{13}C NMR (101 MHz, $CDCl_3$)

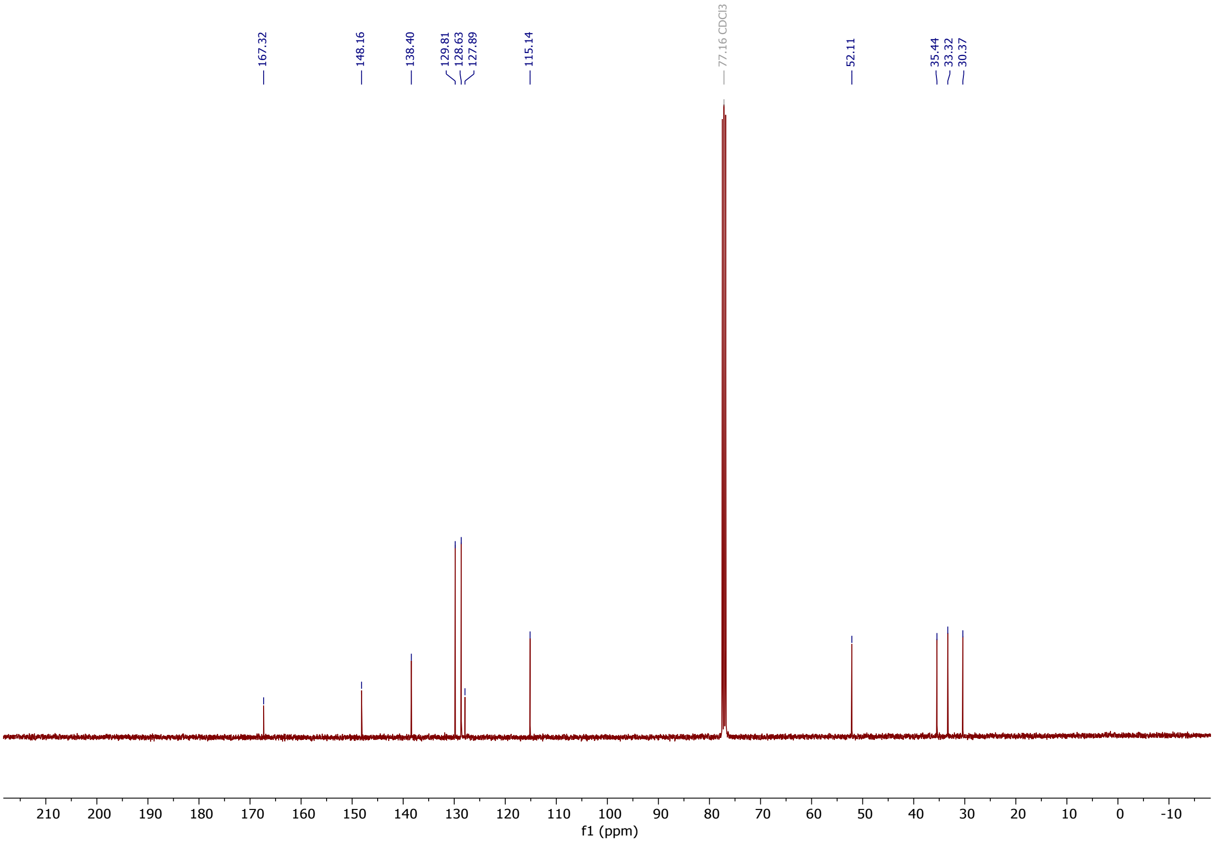

11, ^{1}H NMR (400 MHz, $CDCl_3$)

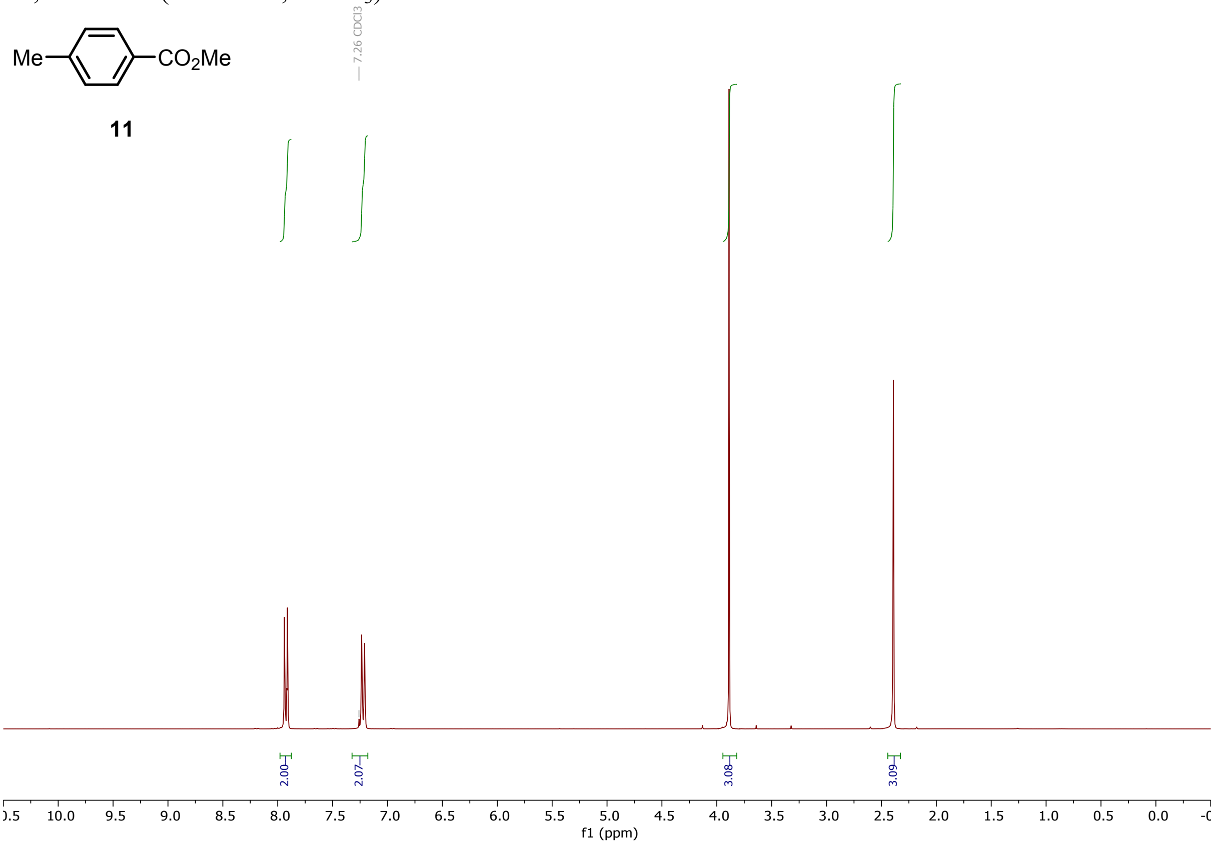

11, ^{13}C NMR (101 MHz, $CDCl_3$)

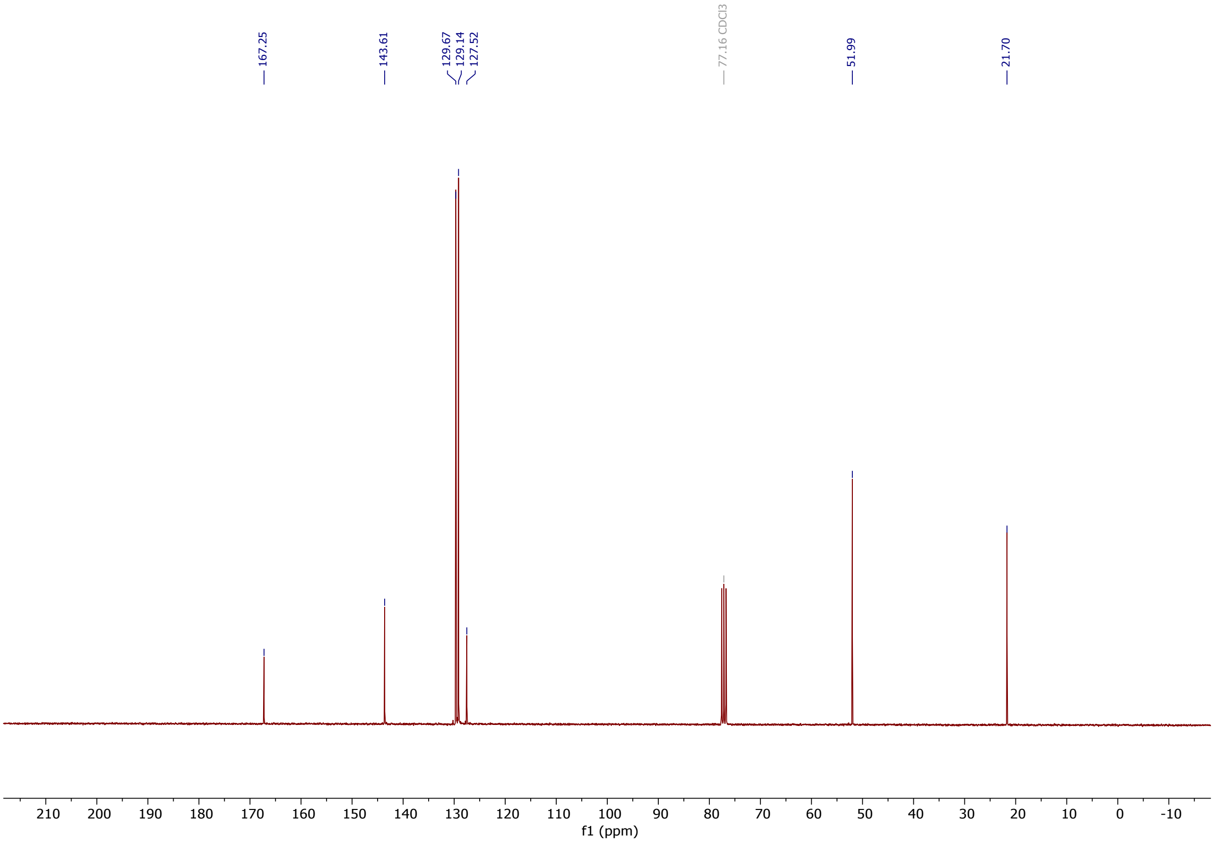

12, ^{1}H NMR (300 MHz, $CDCl_3$)

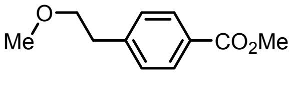

12

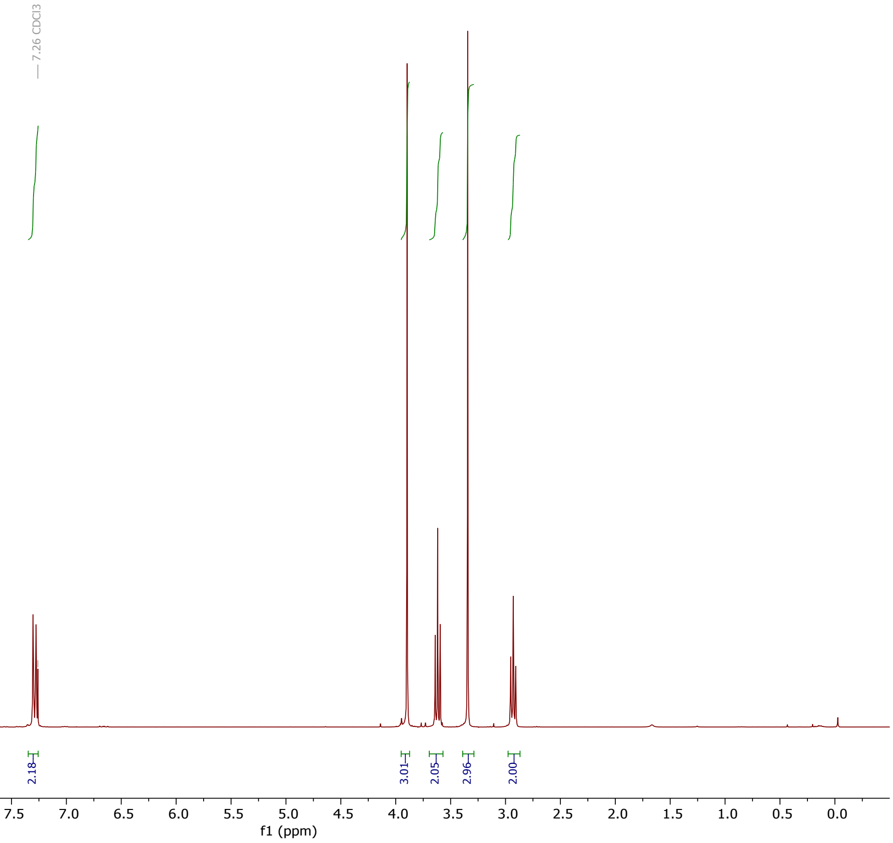

12, ^{13}C NMR (75 MHz, $CDCl_3$)

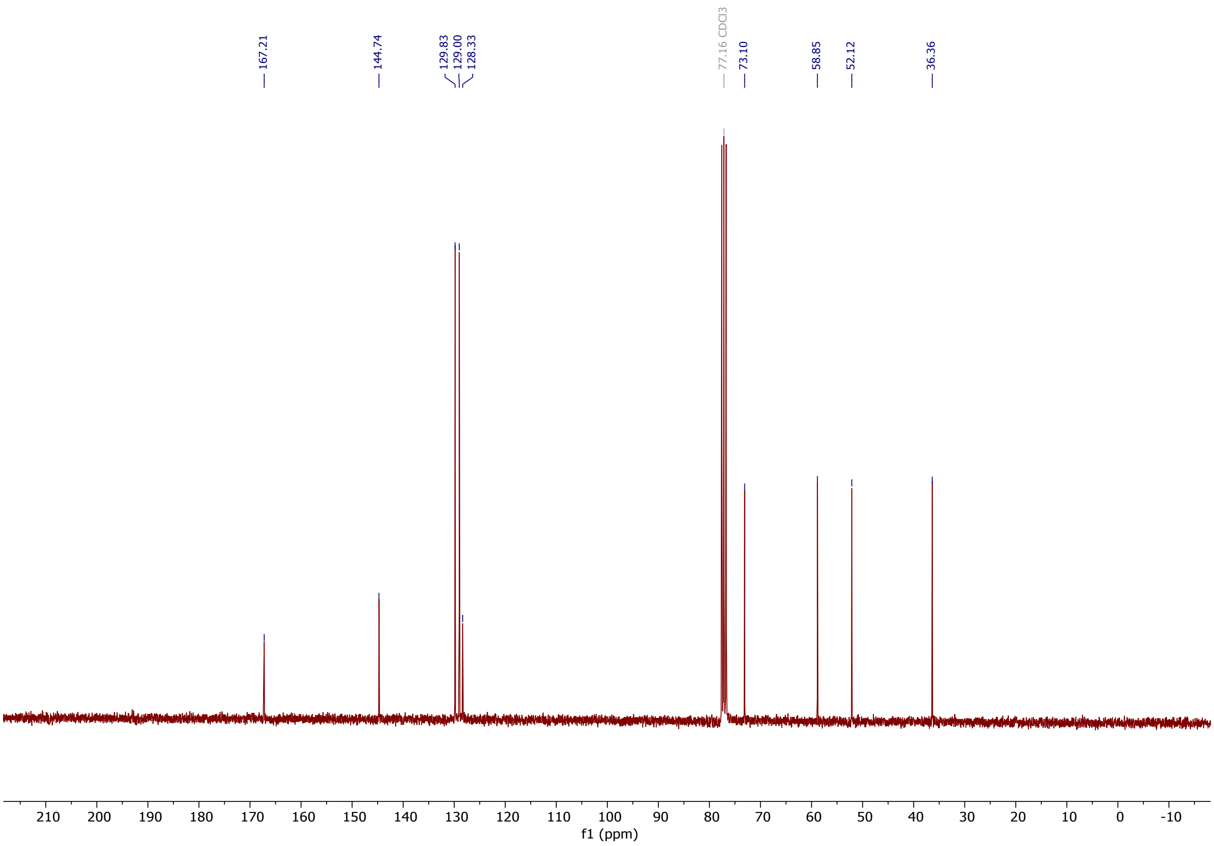

13, ^{1}H NMR (400 MHz, $CDCl_3$)

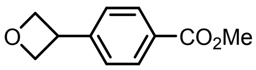

13

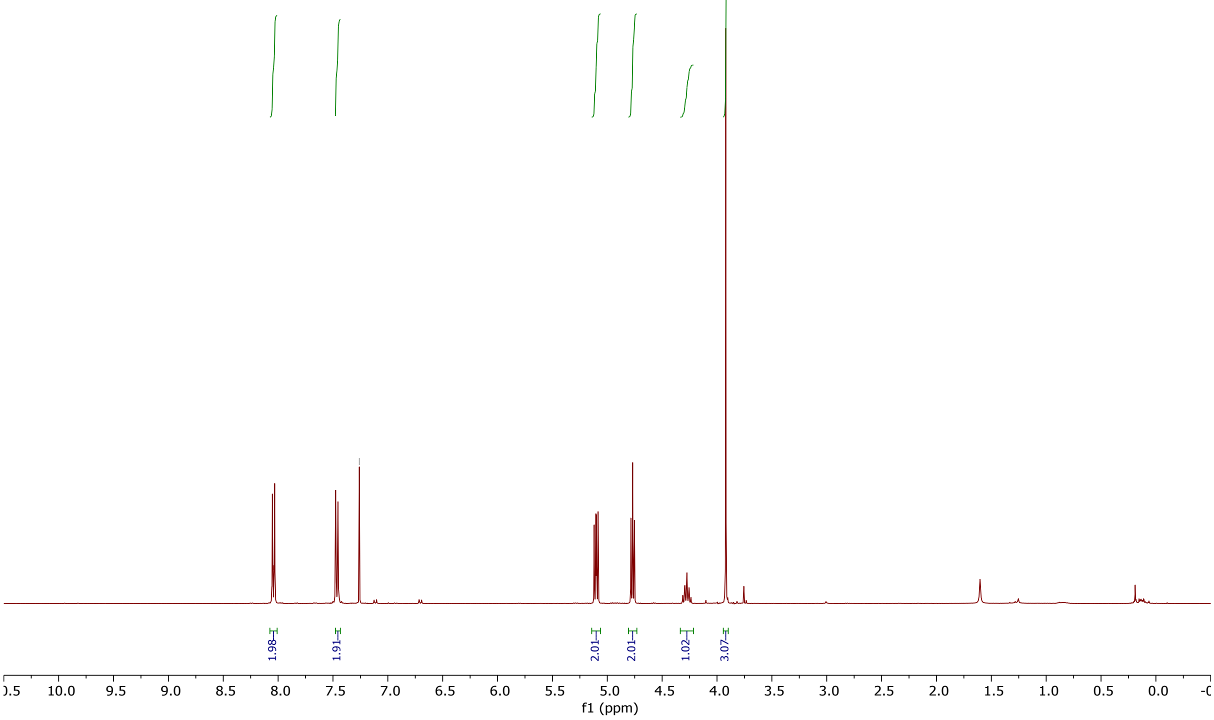

13, ^{13}C NMR (101 MHz, $CDCl_3$)

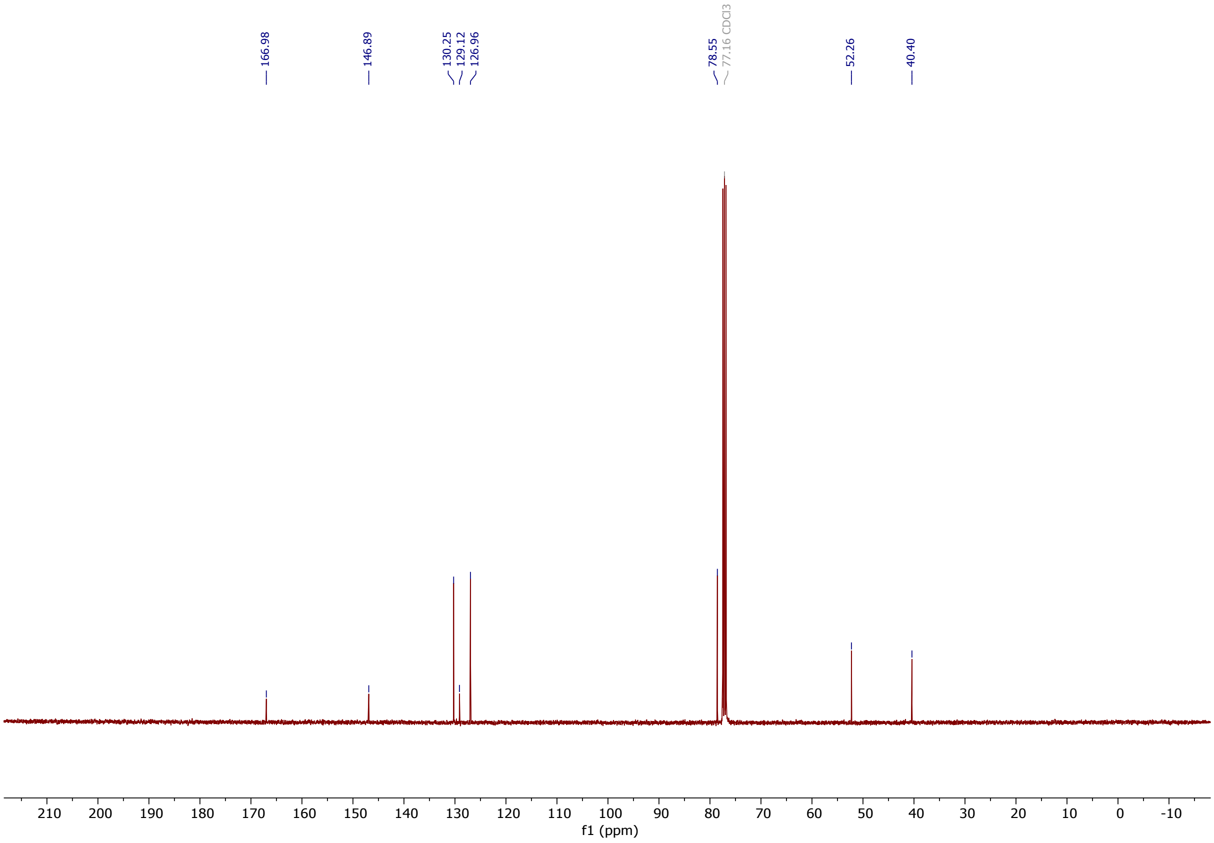

14, ^{1}H NMR (400 MHz, $CDCl_3$)

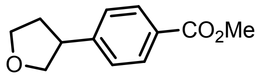

14

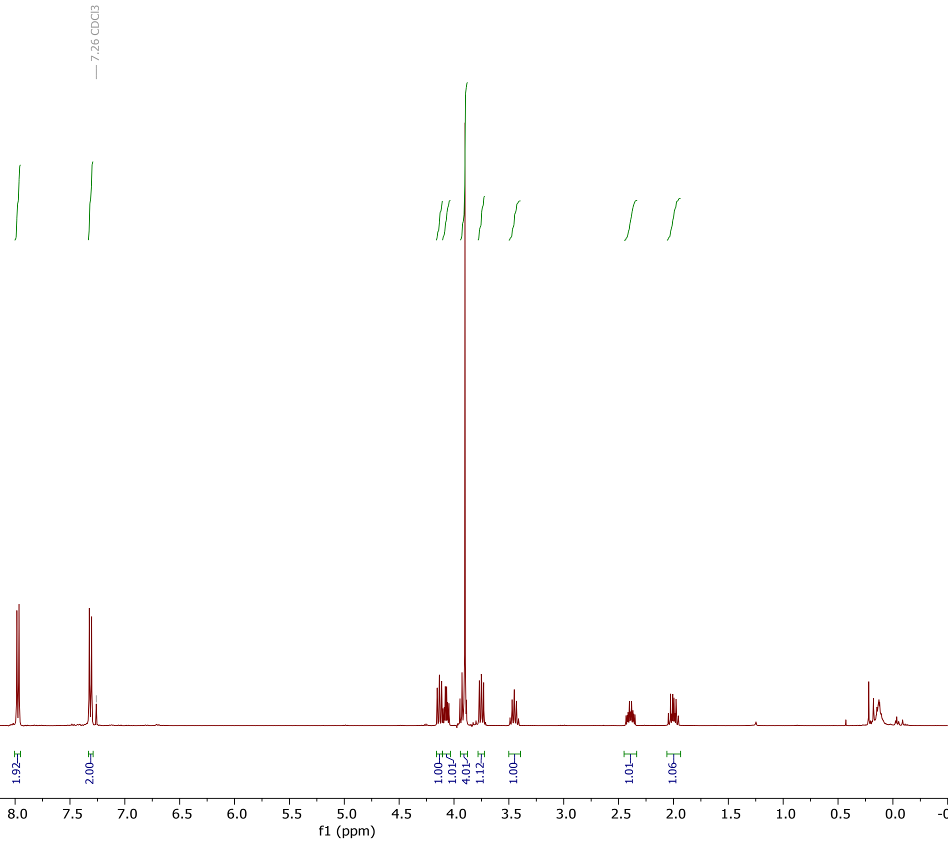

14, ^{13}C NMR (101 MHz, $CDCl_3$)

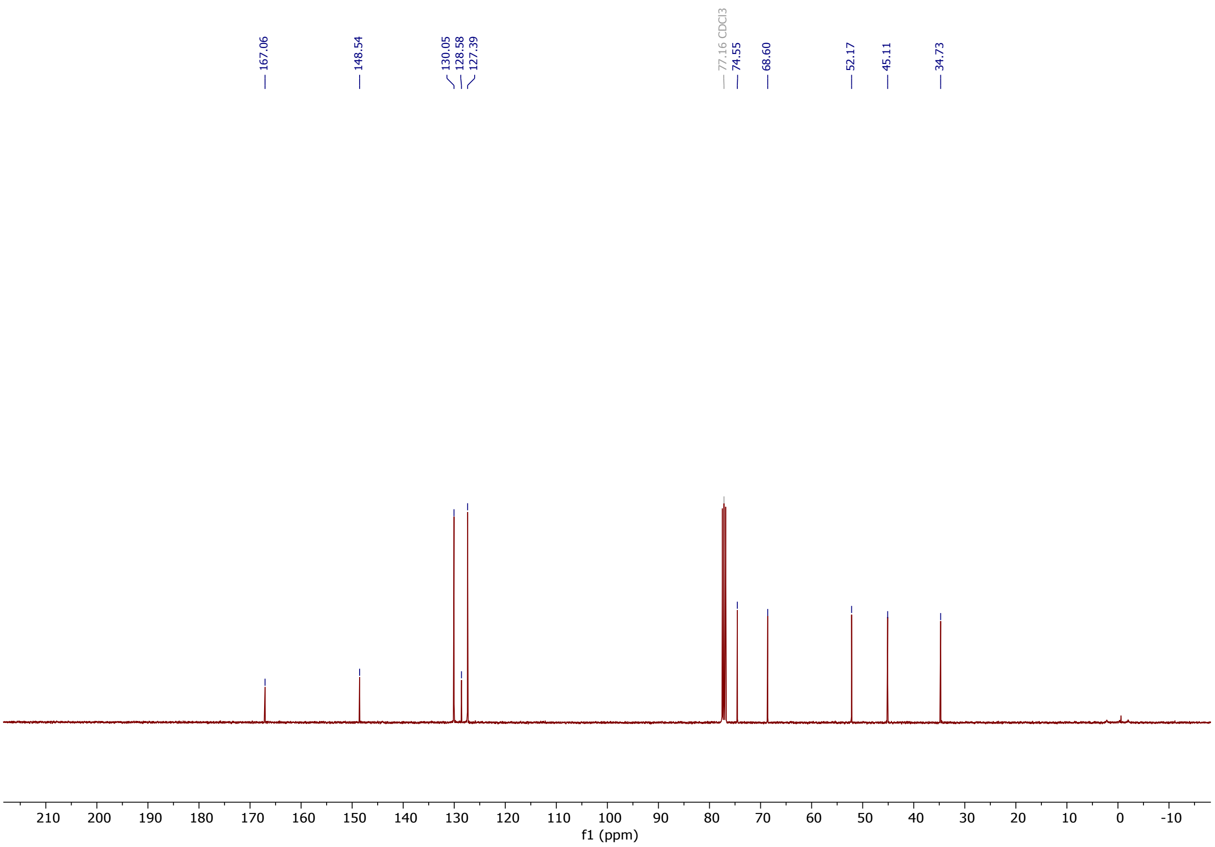

15, ^{1}H NMR (400 MHz, $CDCl_3$)

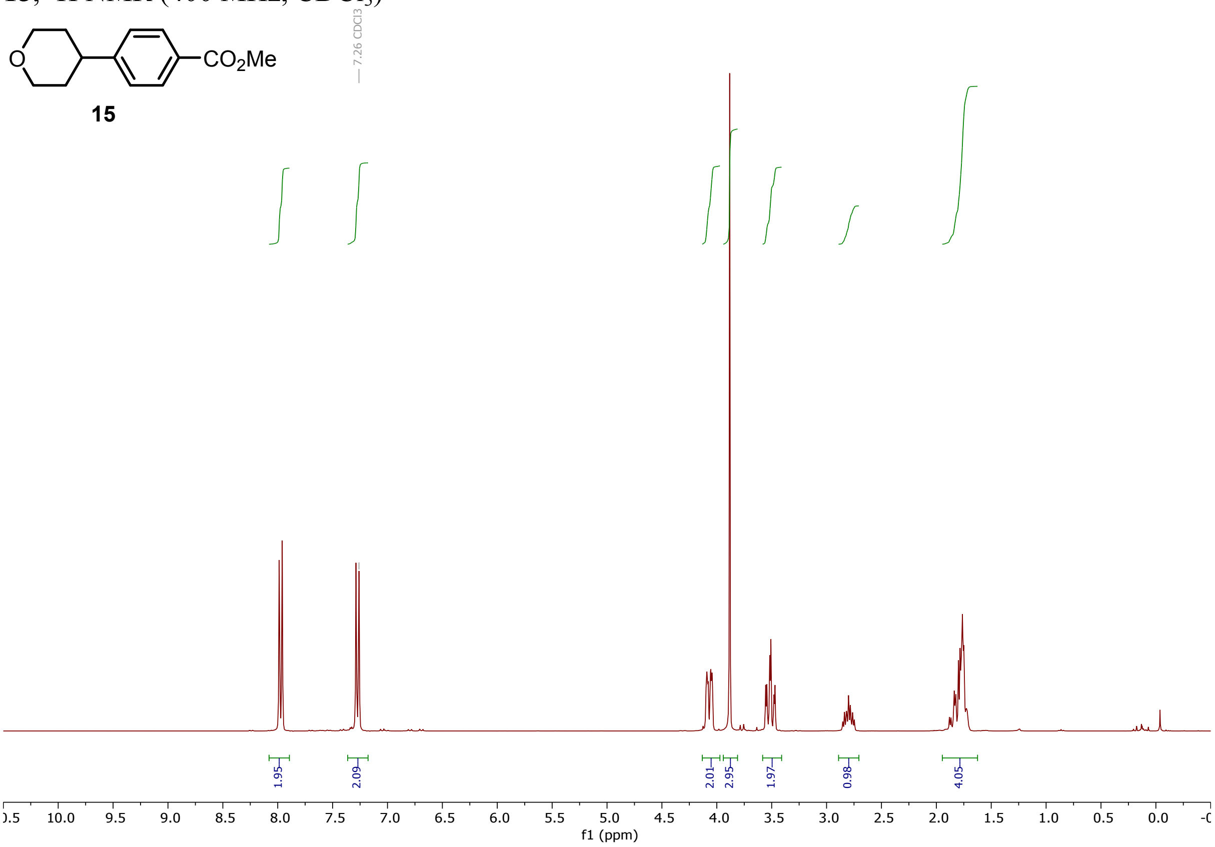

15, ^{13}C NMR (101 MHz, $CDCl_3$)

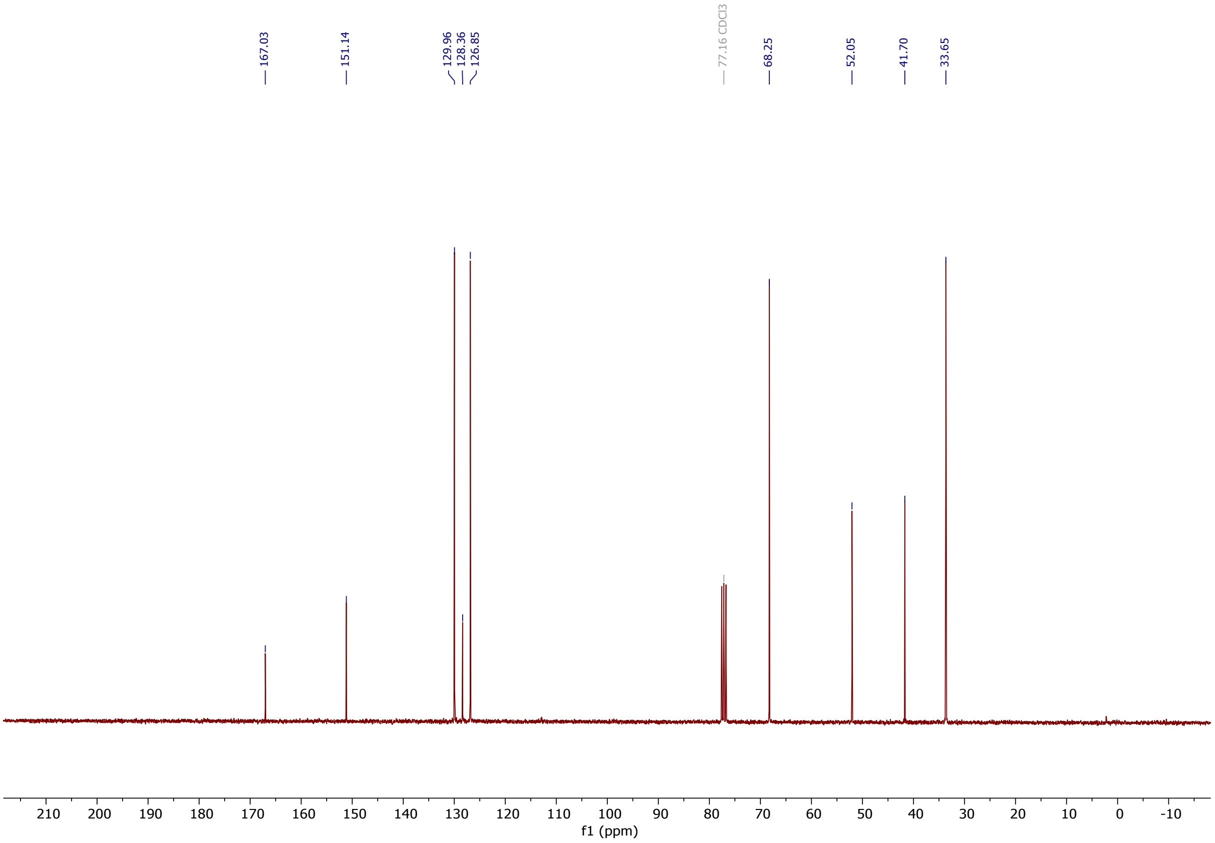

16, ^{1}H NMR (300 MHz, $CDCl_3$)

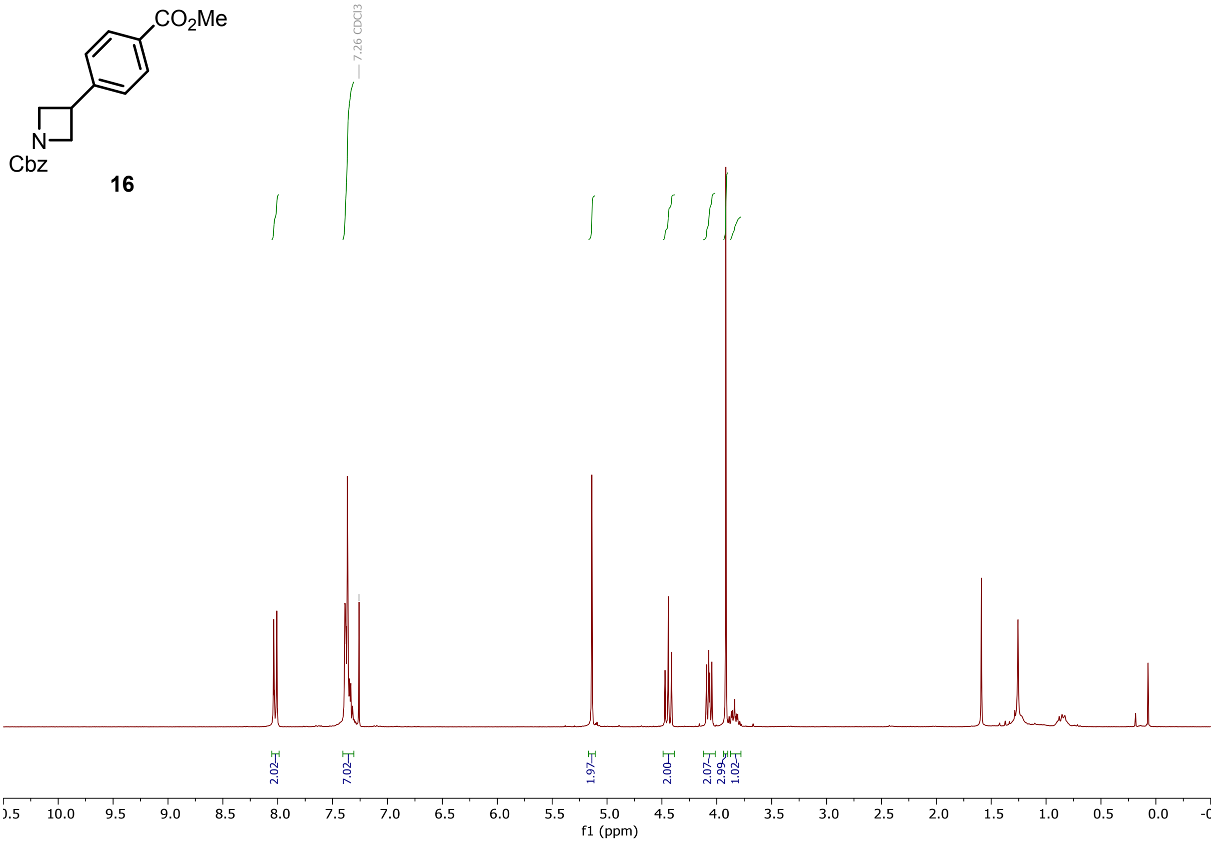

16, ^{13}C NMR (101 MHz, $CDCl_3$)

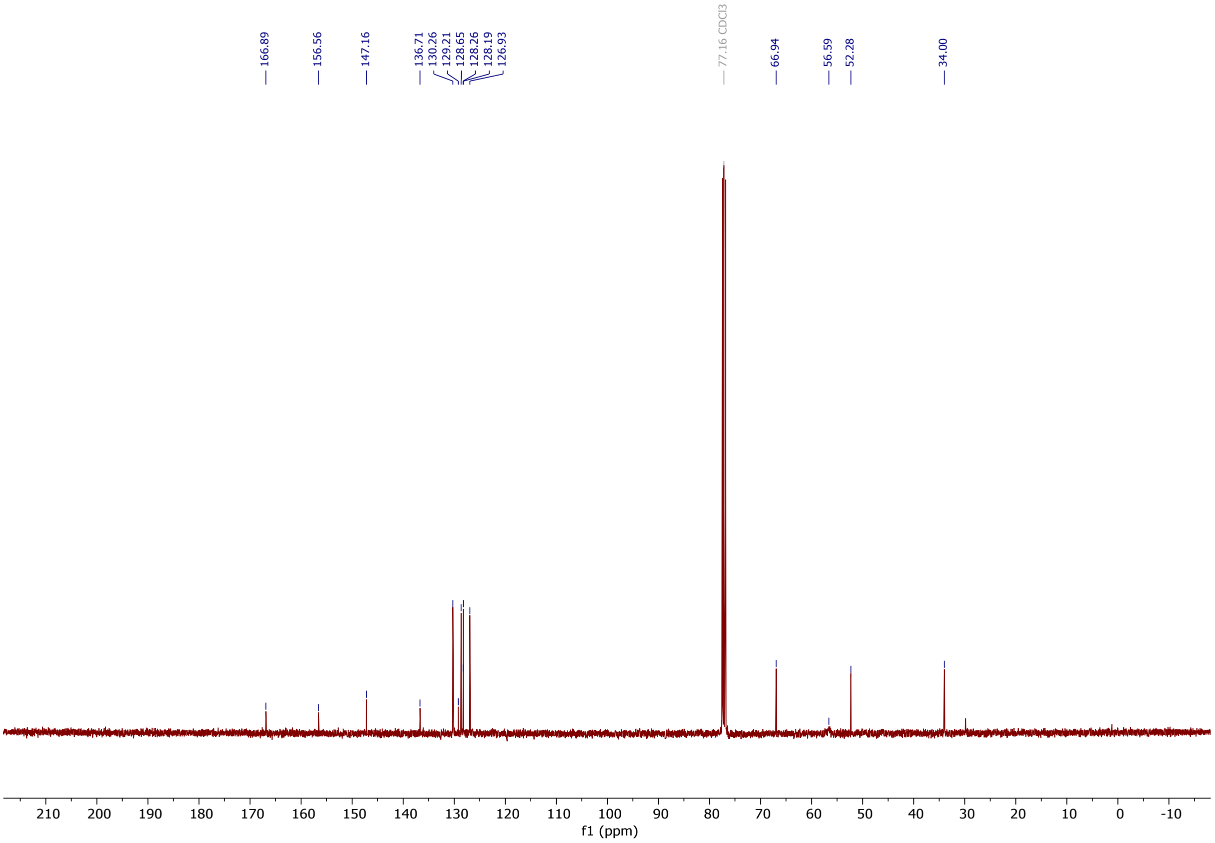

17, ^{1}H NMR (300 MHz, $CDCl_3$)

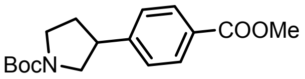

17

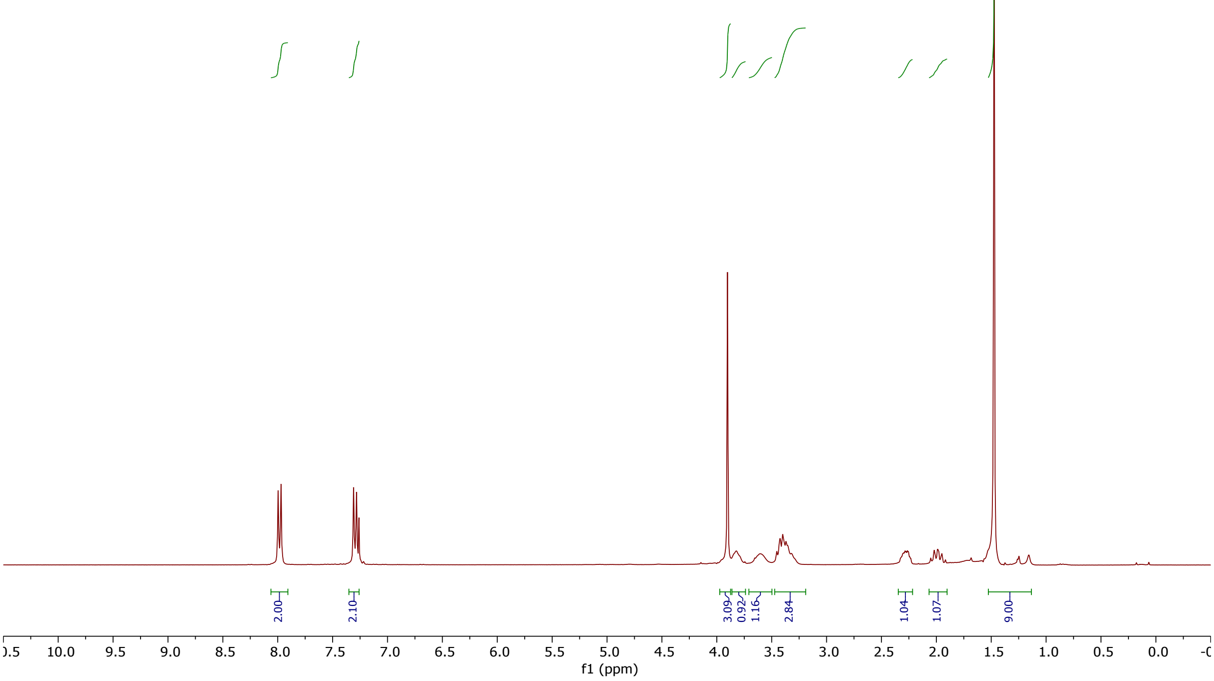

17, ^{13}C NMR (101 MHz, $CDCl_3$)

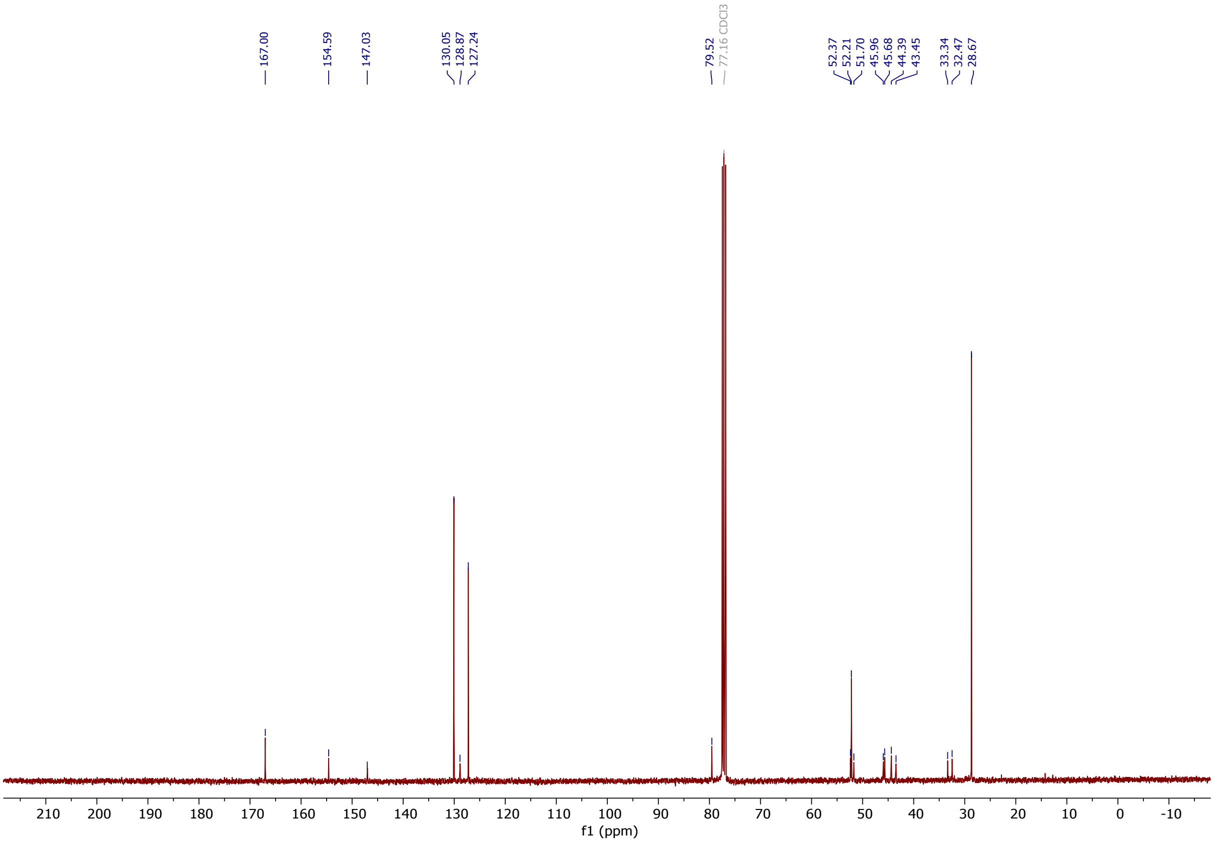

18, ^{1}H NMR (300 MHz, $CDCl_3$)

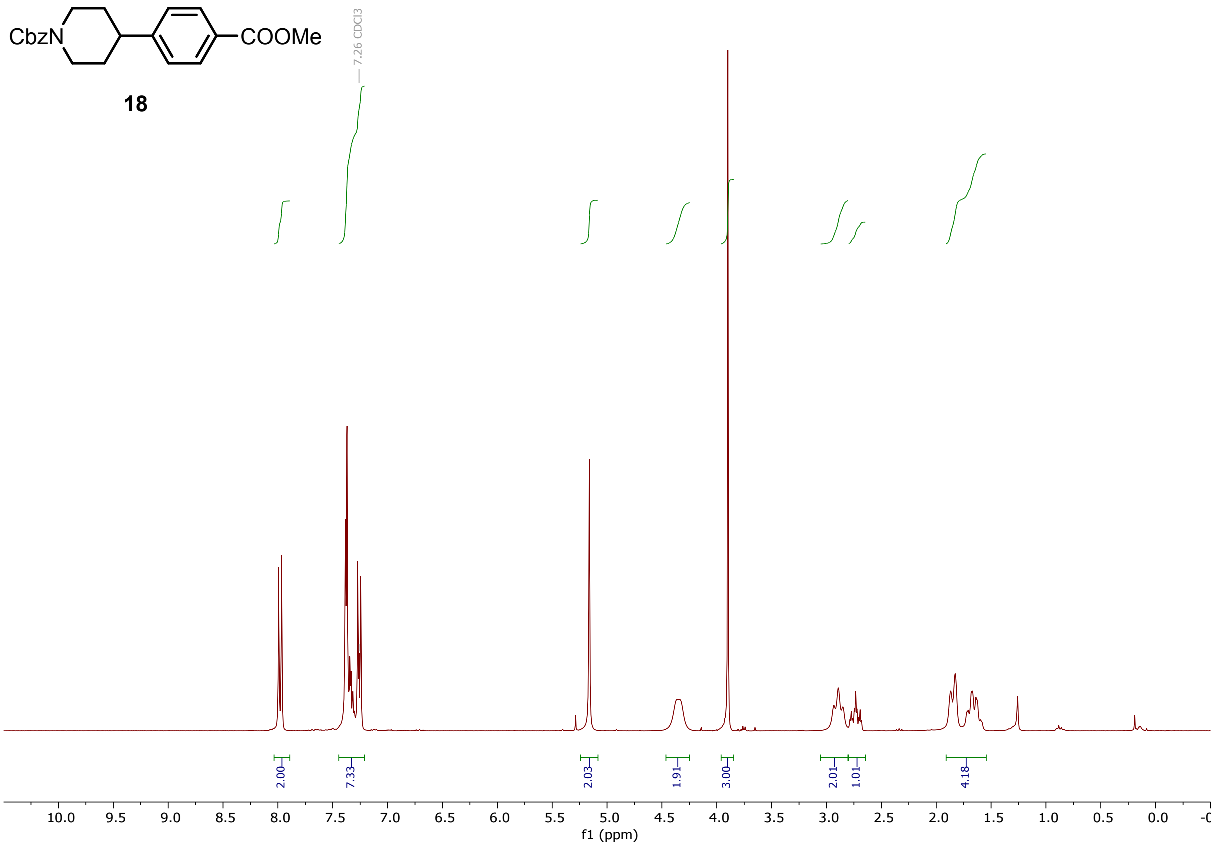

18, ^{13}C NMR (101 MHz, $CDCl_3$)

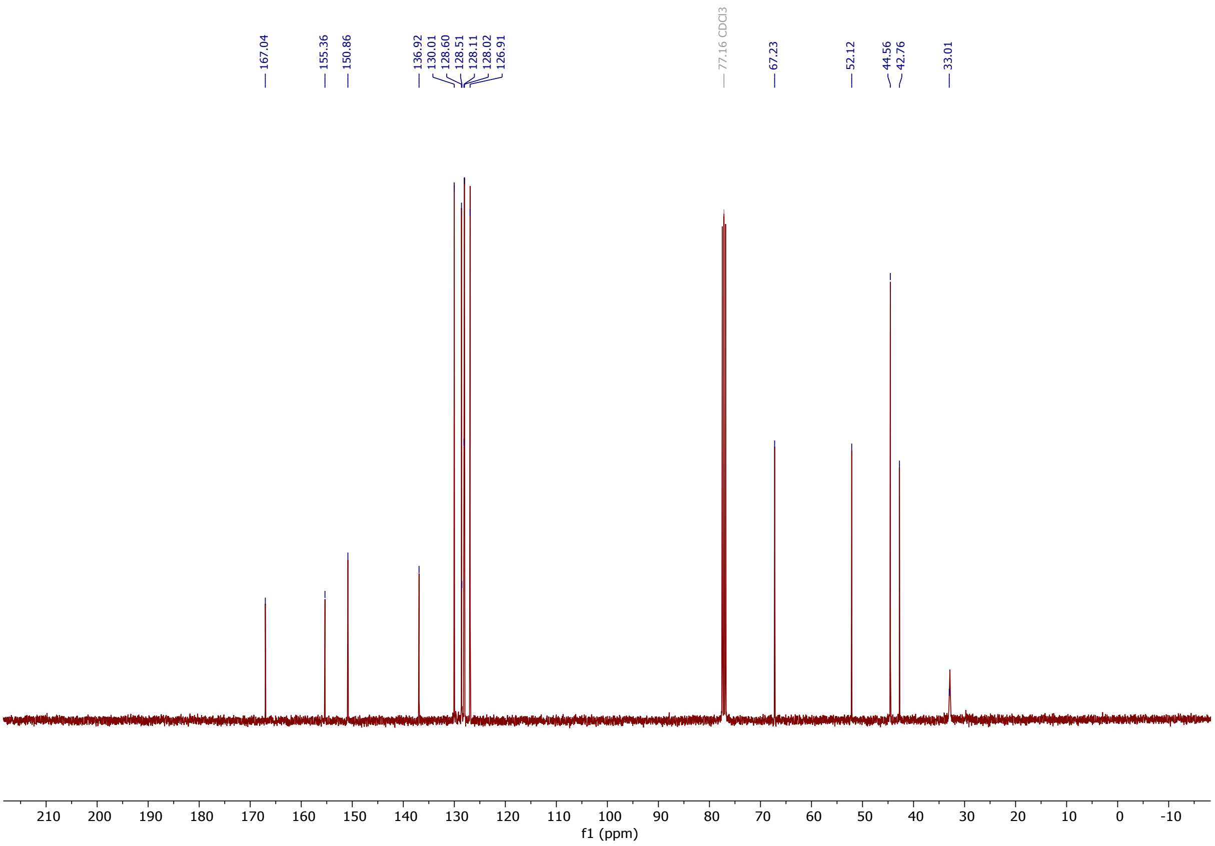

19, ^{1}H NMR (400 MHz, C_6D_6)

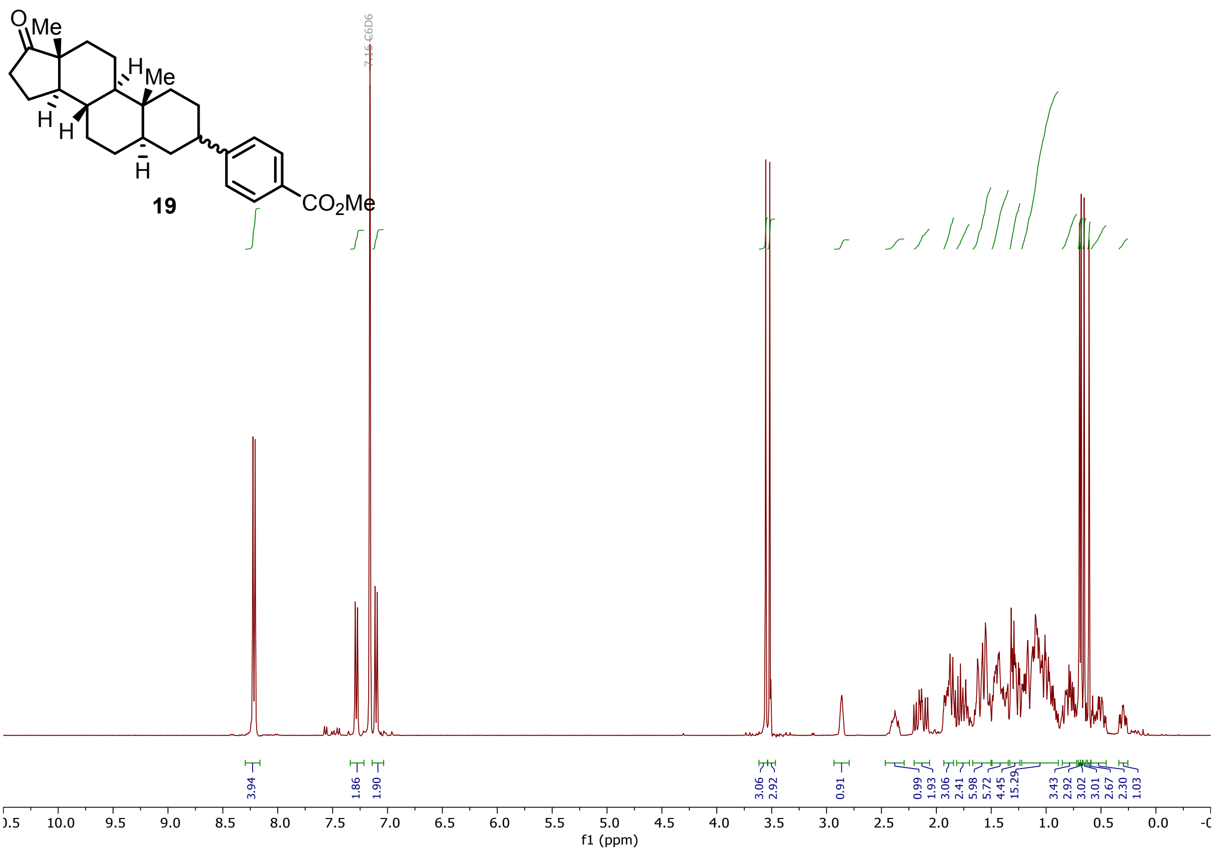

19, ^{13}C NMR (75 MHz, C_6D_6)

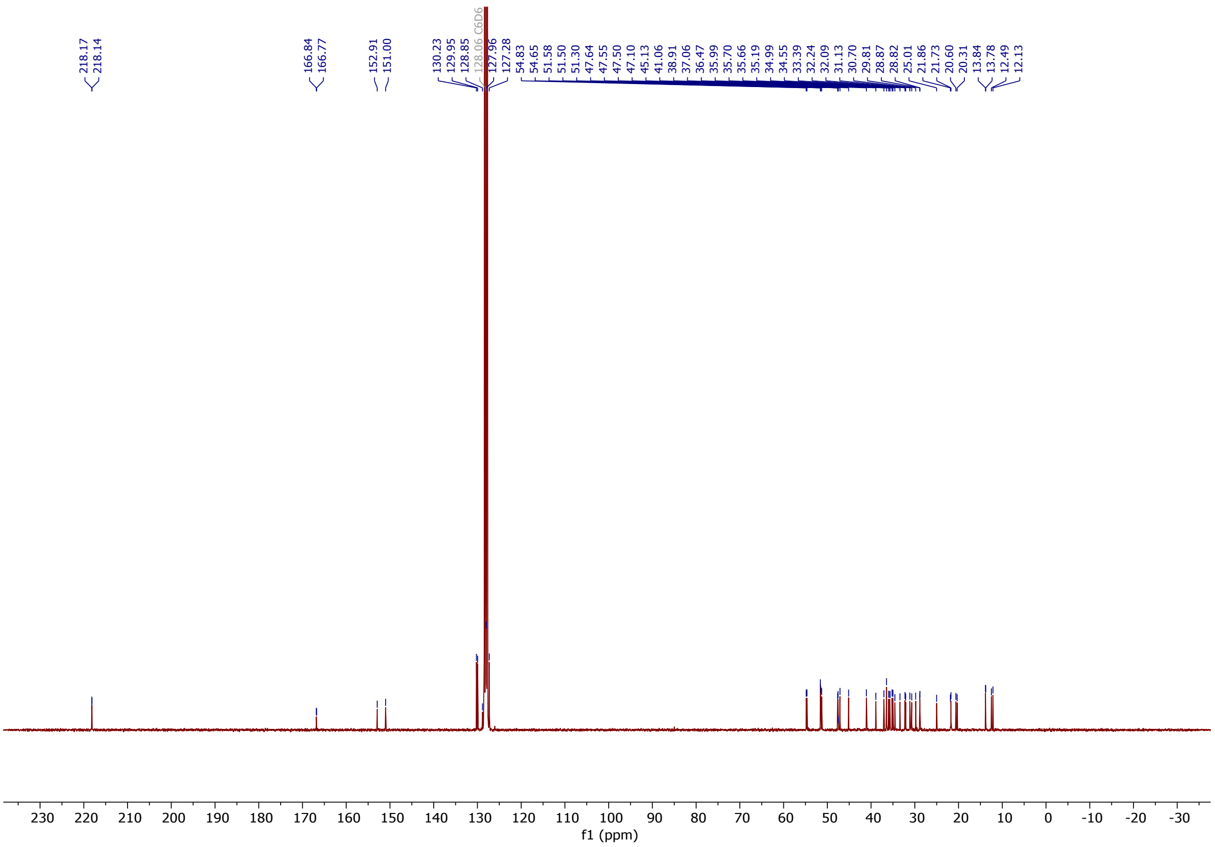

20, ^{1}H NMR (400 MHz, C_6D_6)

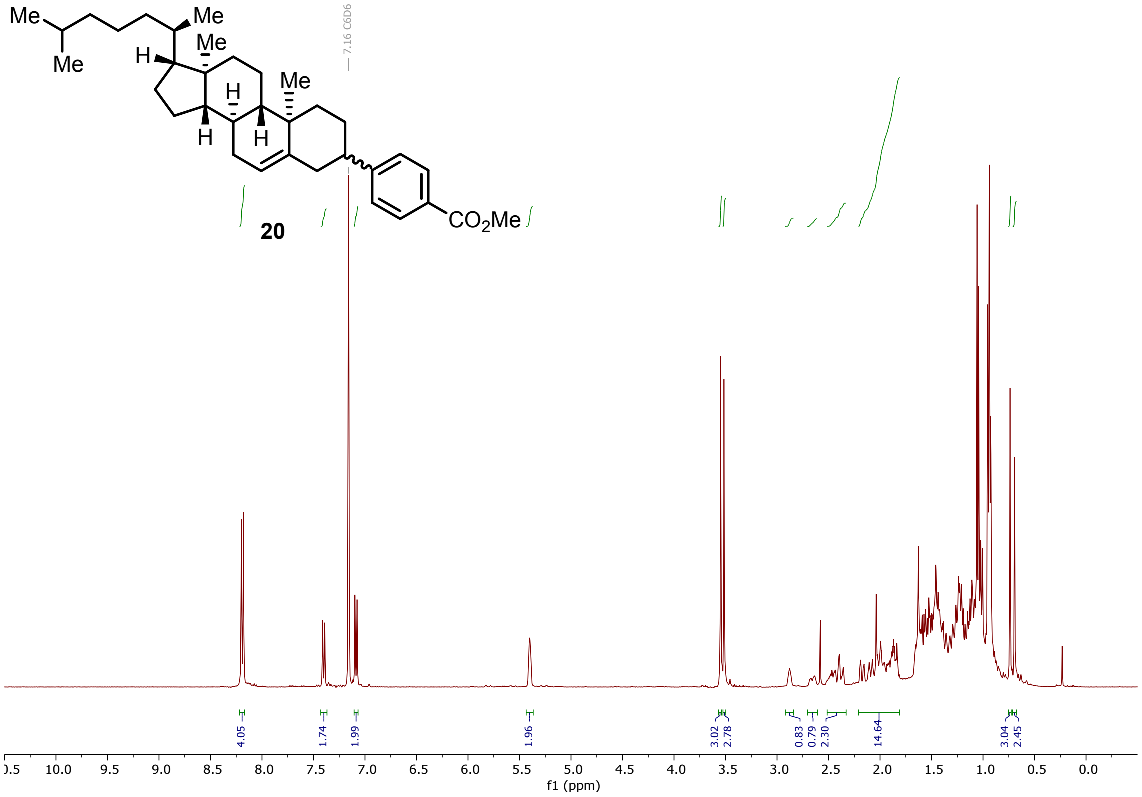

20, ^{13}C NMR (101 MHz, C_6D_6)

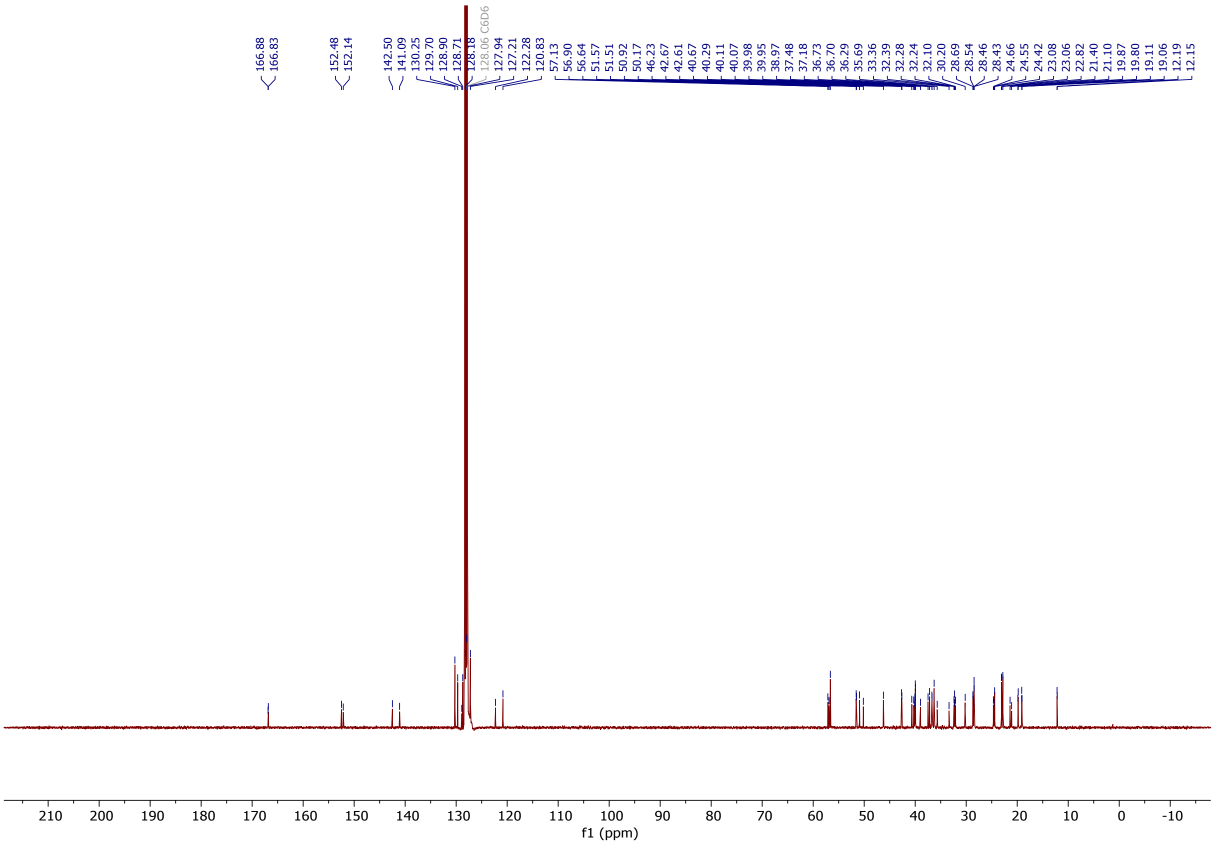

21, ^{1}H NMR (300 MHz, $CDCl_3$)

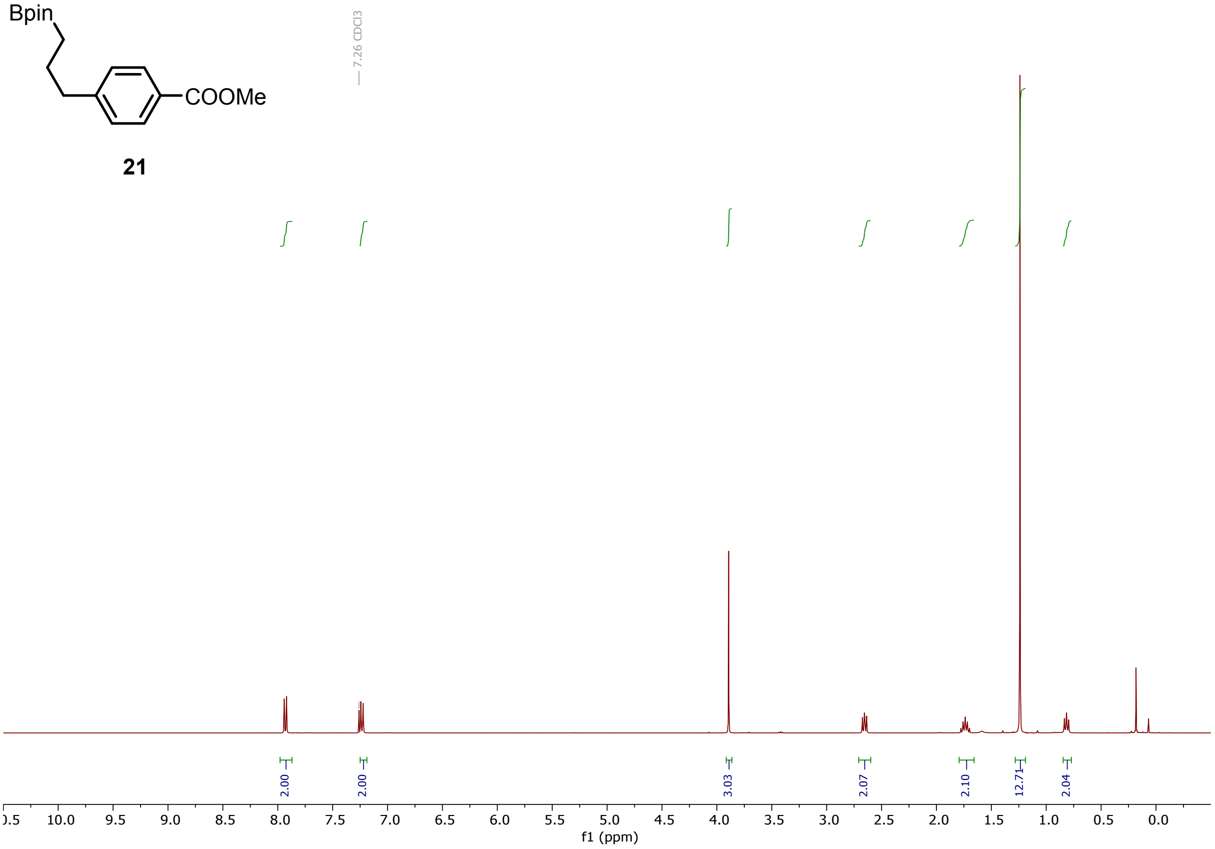

21, ^{13}C NMR (101 MHz, $CDCl_3$)

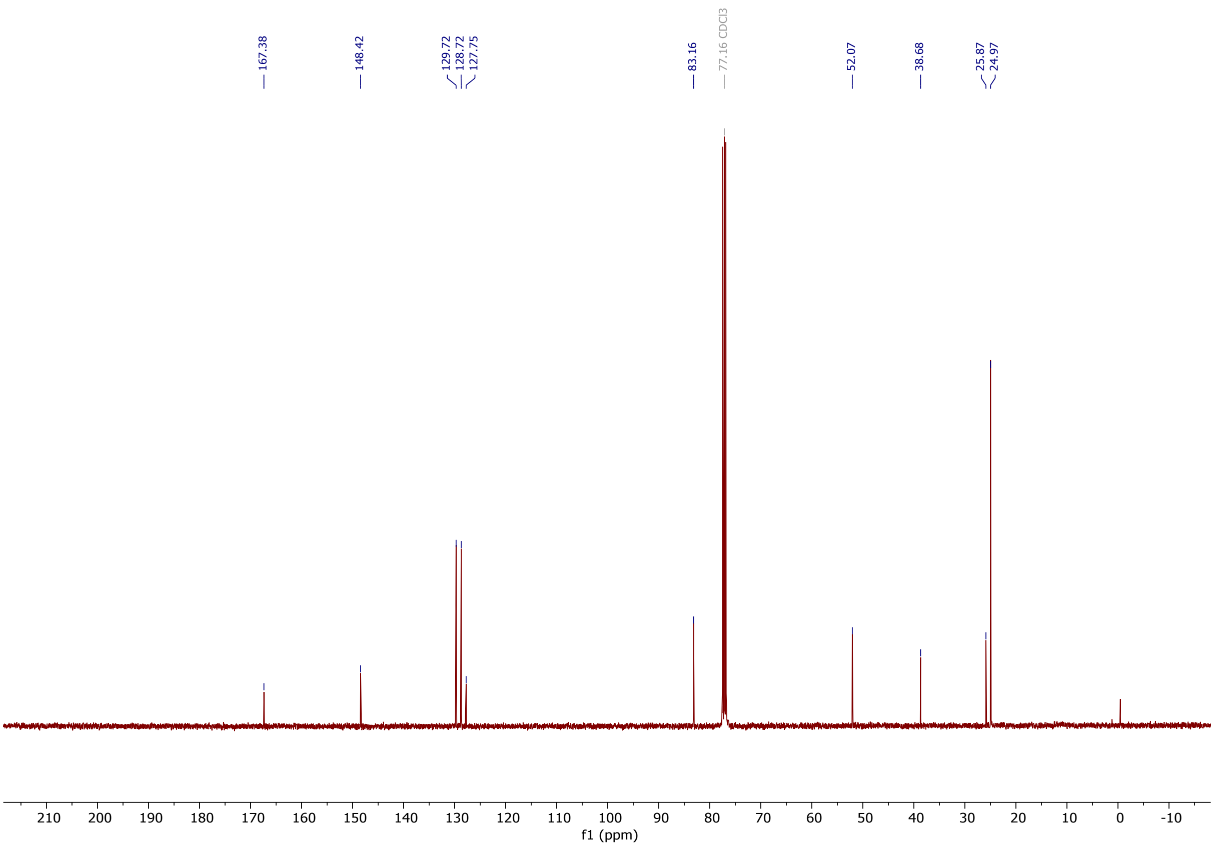

22, ^{1}H NMR (400 MHz, $CDCl_3$)

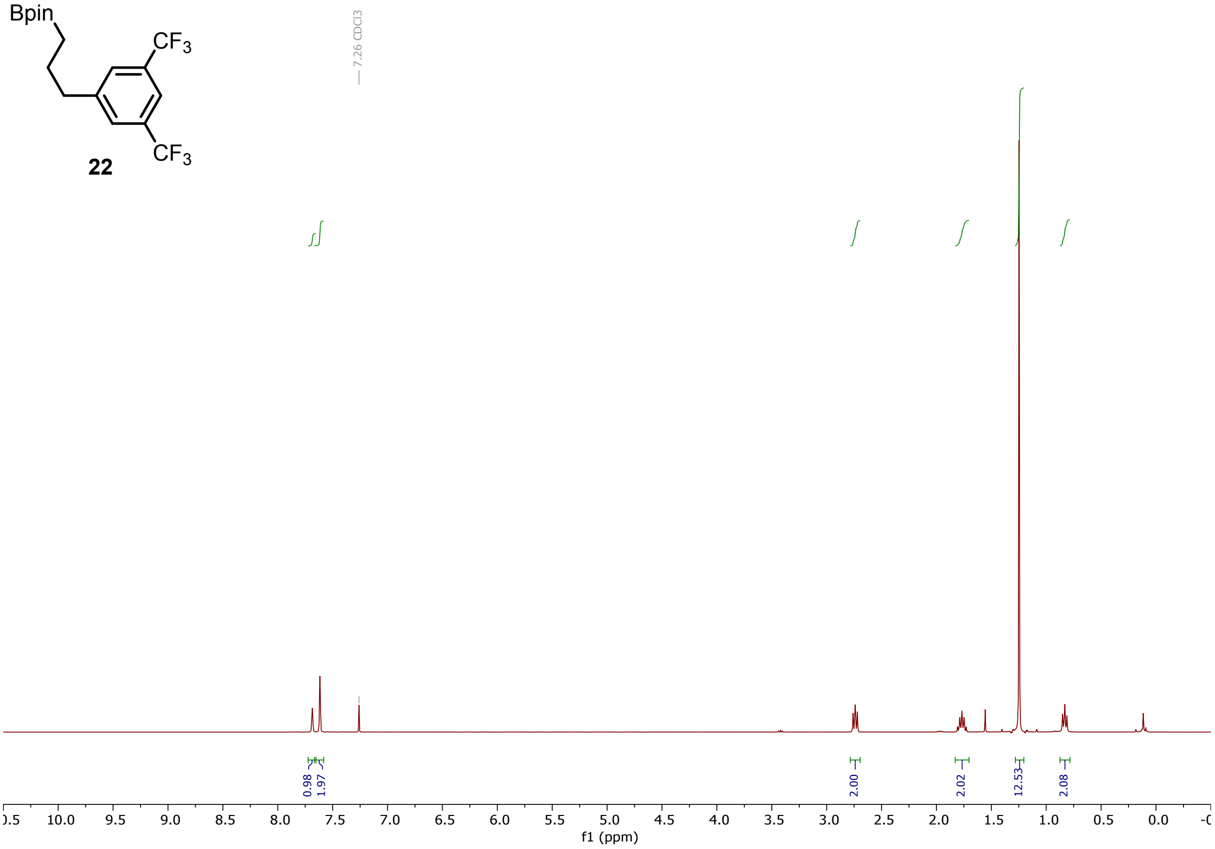

22, ^{13}C NMR (101 MHz, $CDCl_3$)

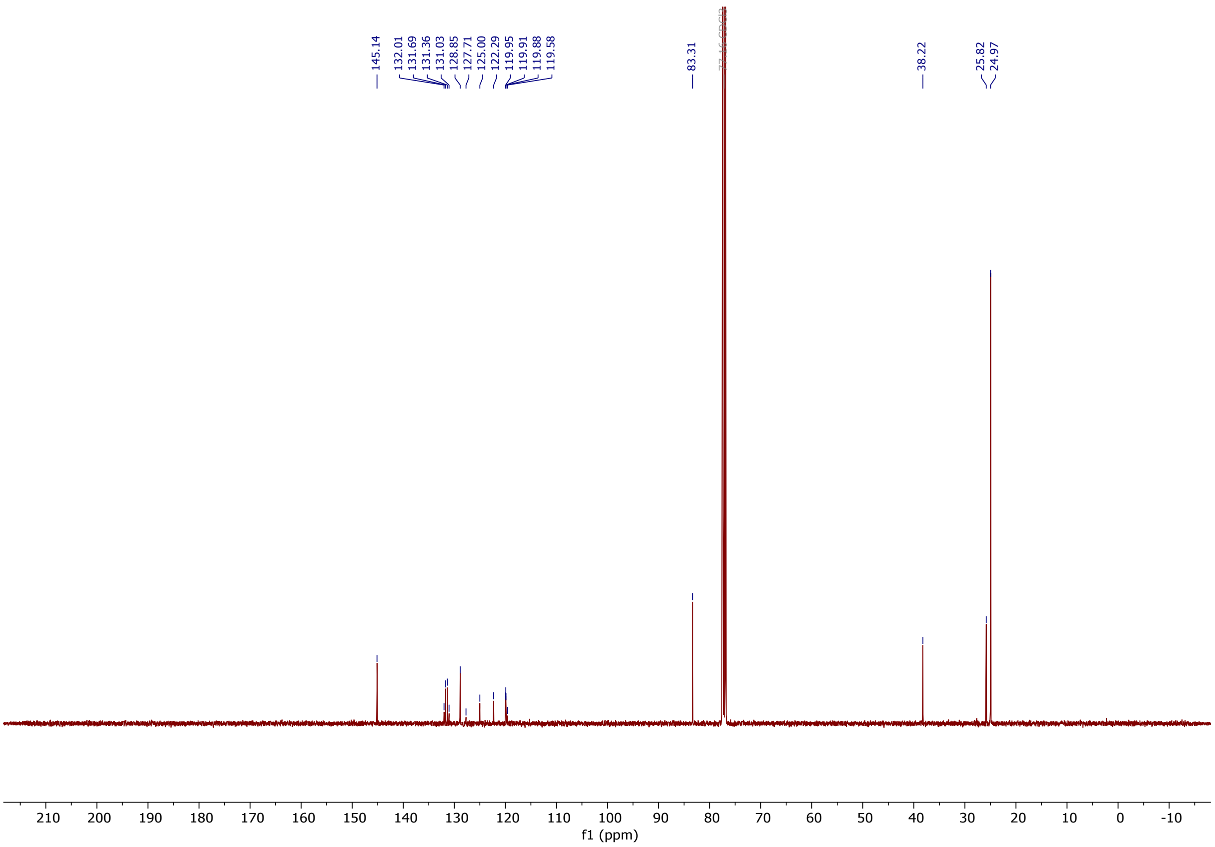

22, ^{19}F NMR (282 MHz, $CDCl_3$)

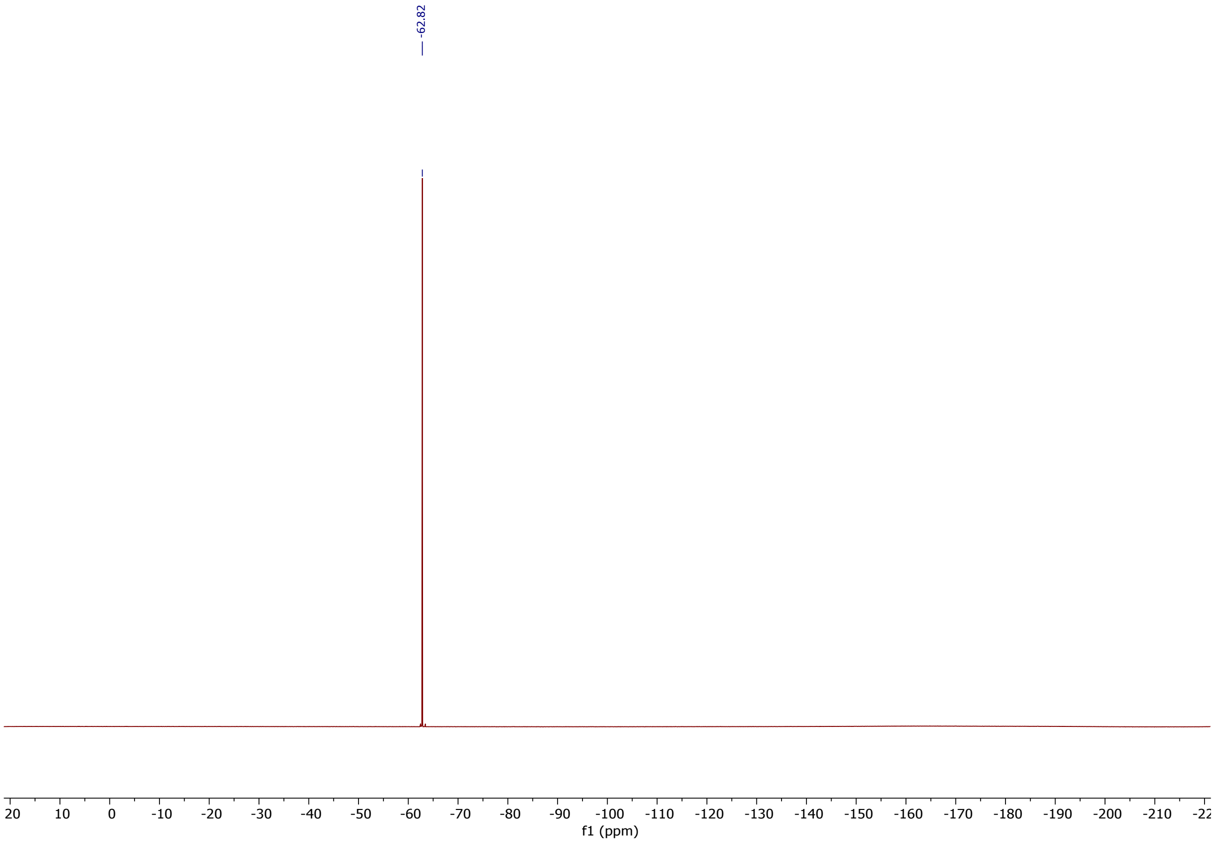

23, ^{1}H NMR (400 MHz, $CDCl_3$)

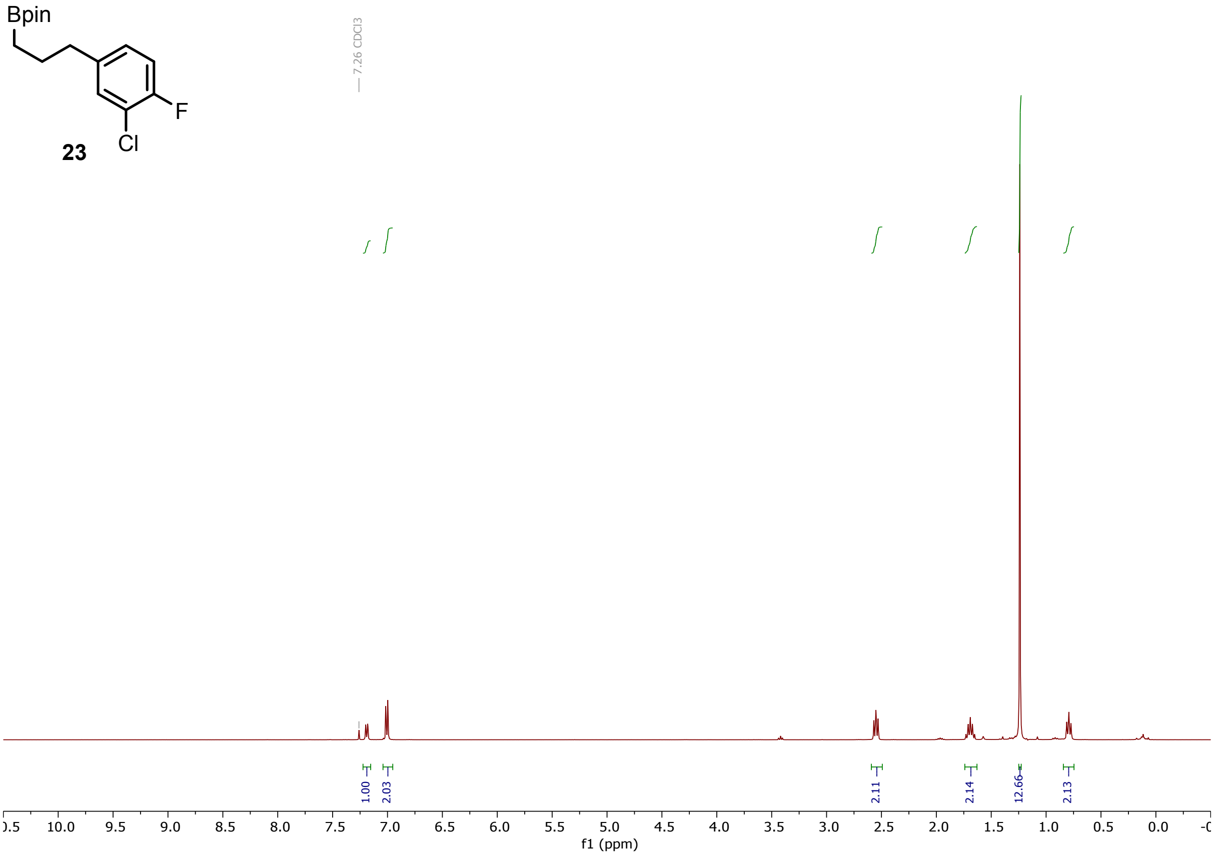

23, ^{13}C NMR (101 MHz, $CDCl_3$)

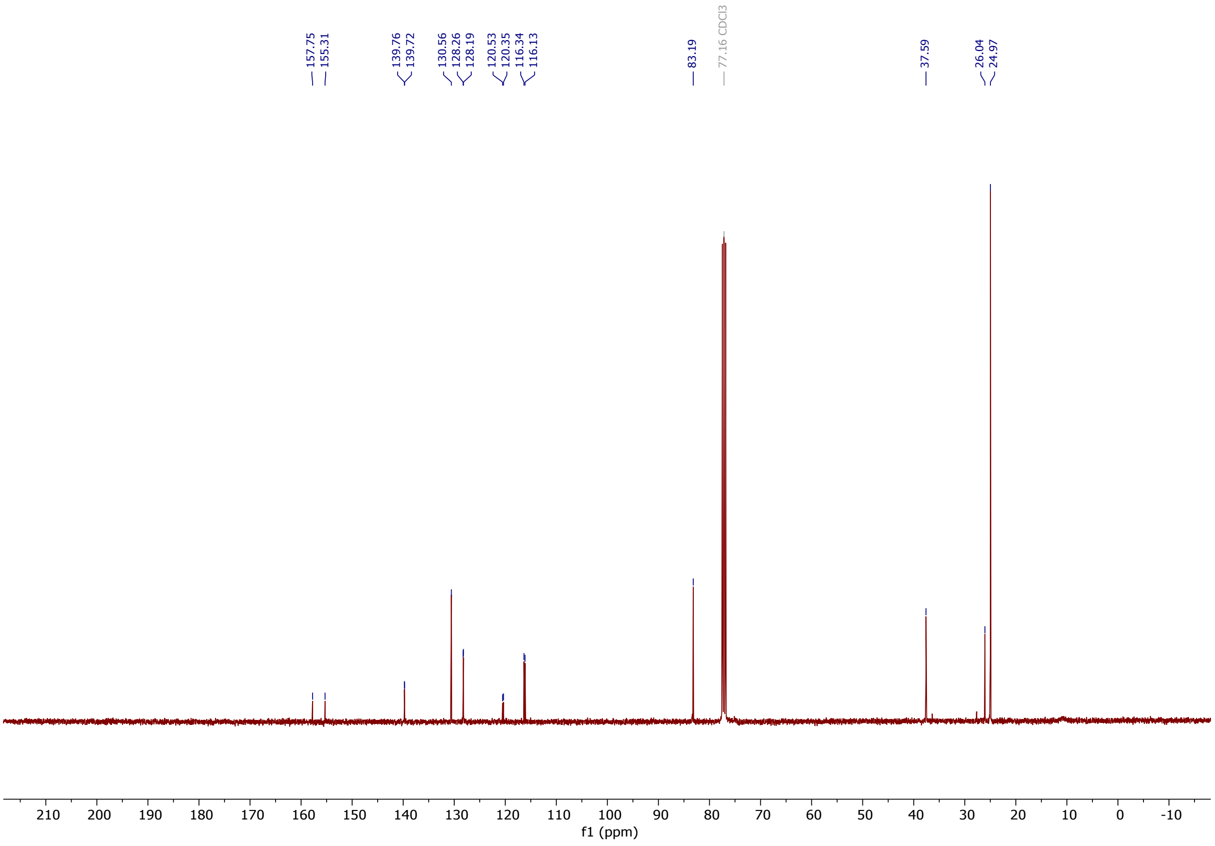

23, ^{19}F NMR (376 MHz, $CDCl_3$)

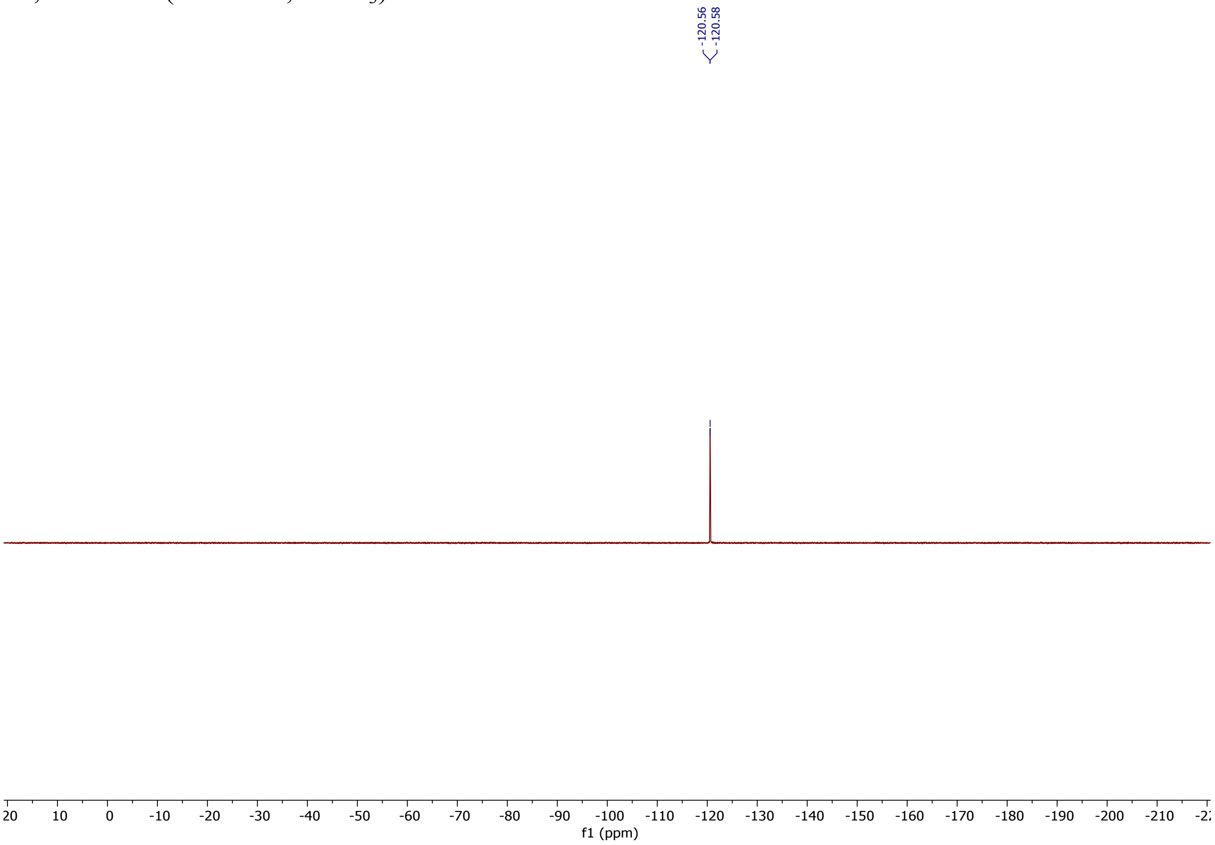

24, ^{1}H NMR (400 MHz, $CDCl_3$)

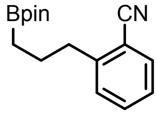

24

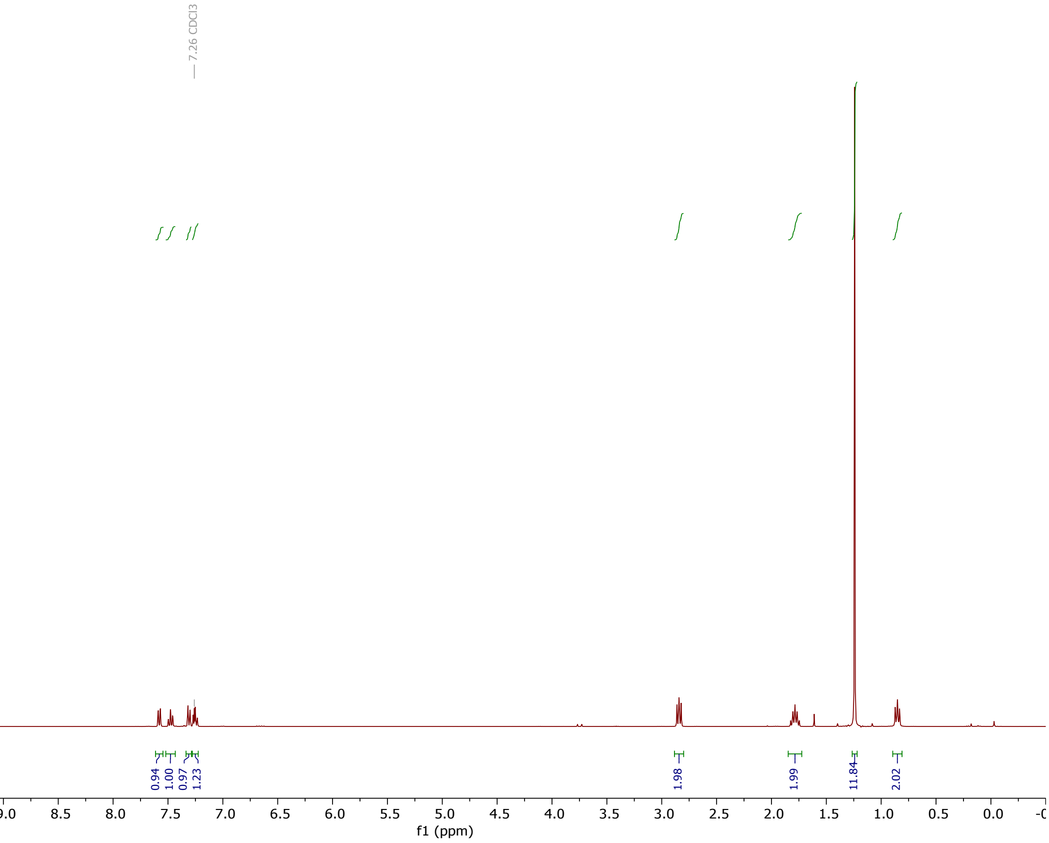

24, ^{13}C NMR (101 MHz, $CDCl_3$)

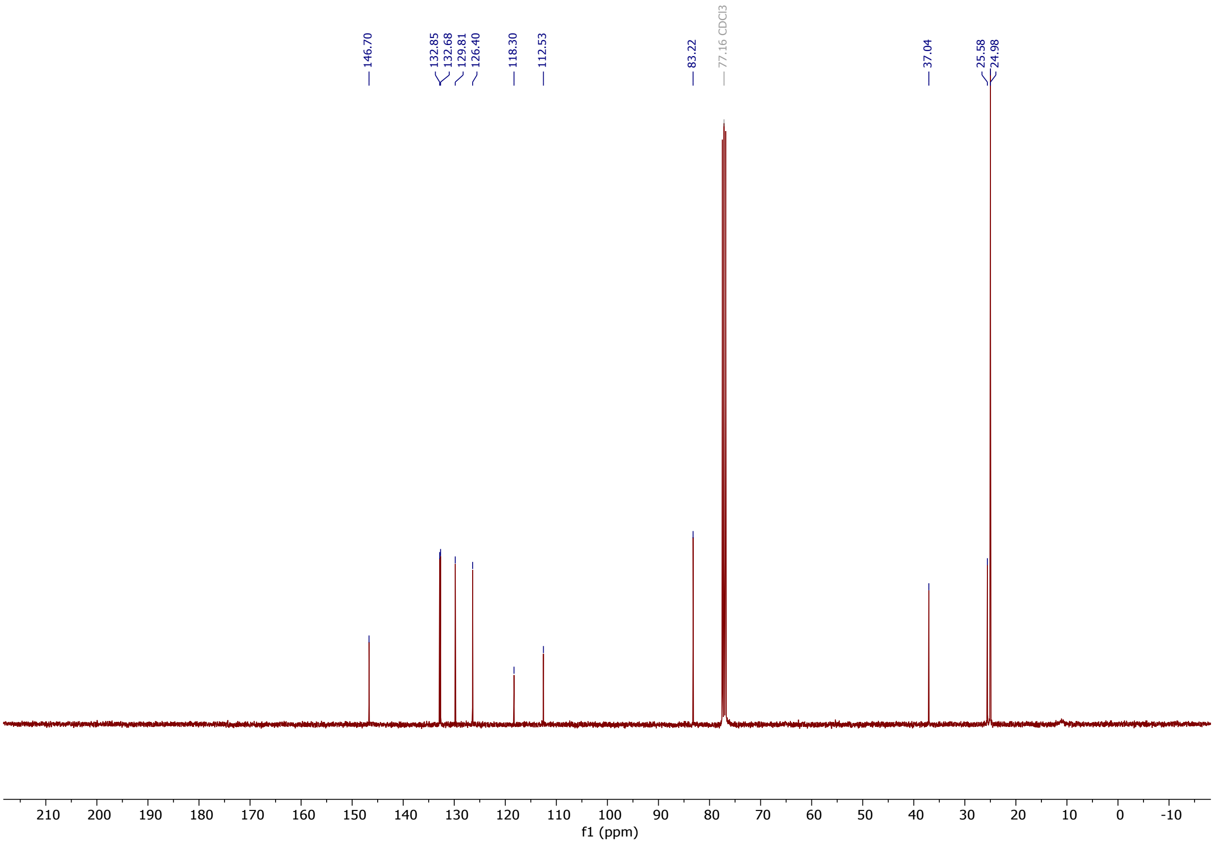

25, ^{1}H NMR (400 MHz, $CDCl_3$)

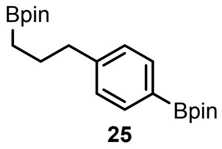

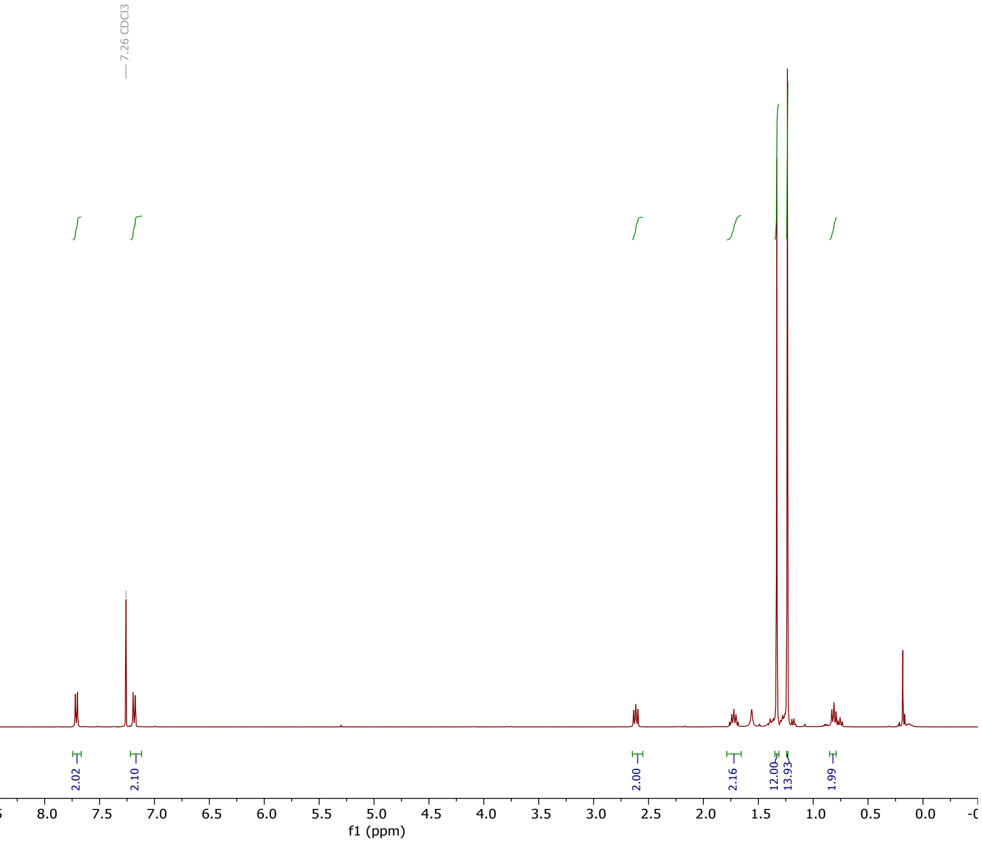

25, ^{13}C NMR (101 MHz, $CDCl_3$)

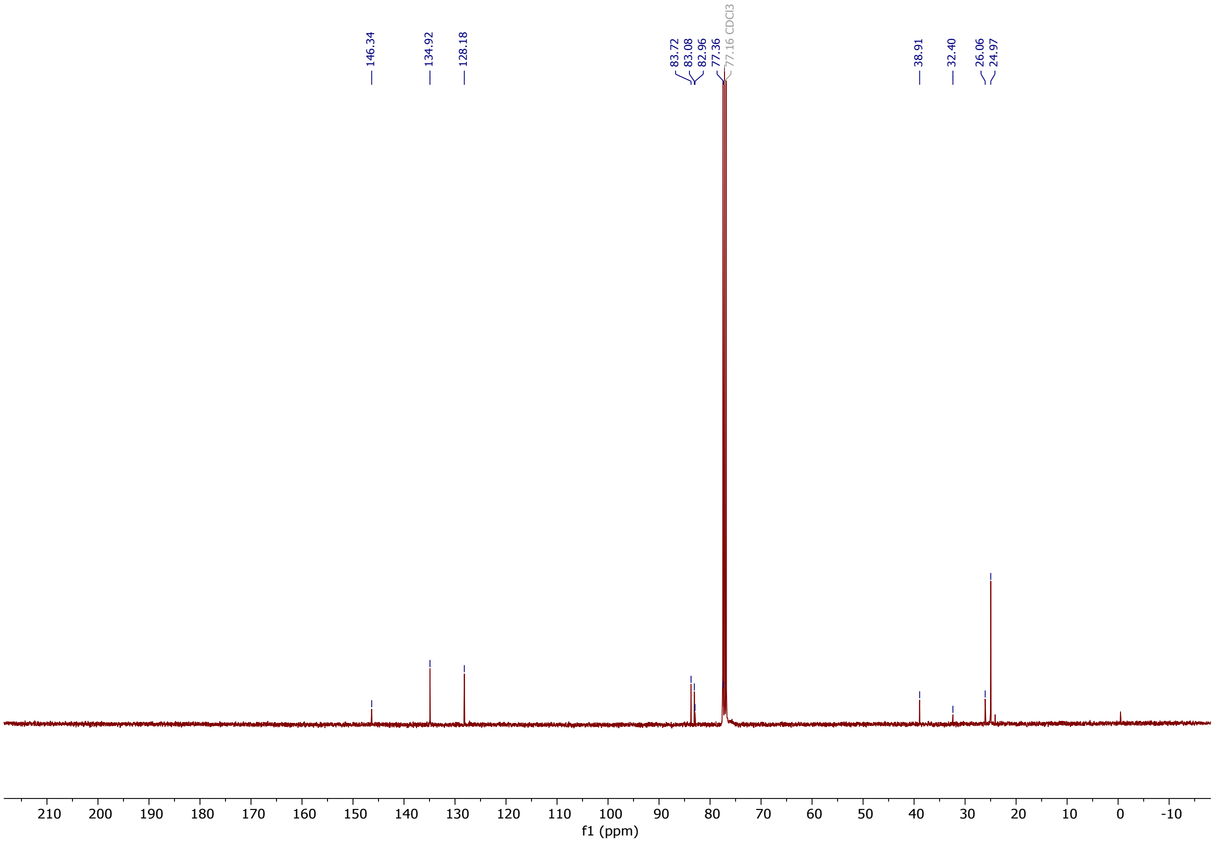

26, ^{1}H NMR (400 MHz, $CDCl_3$)

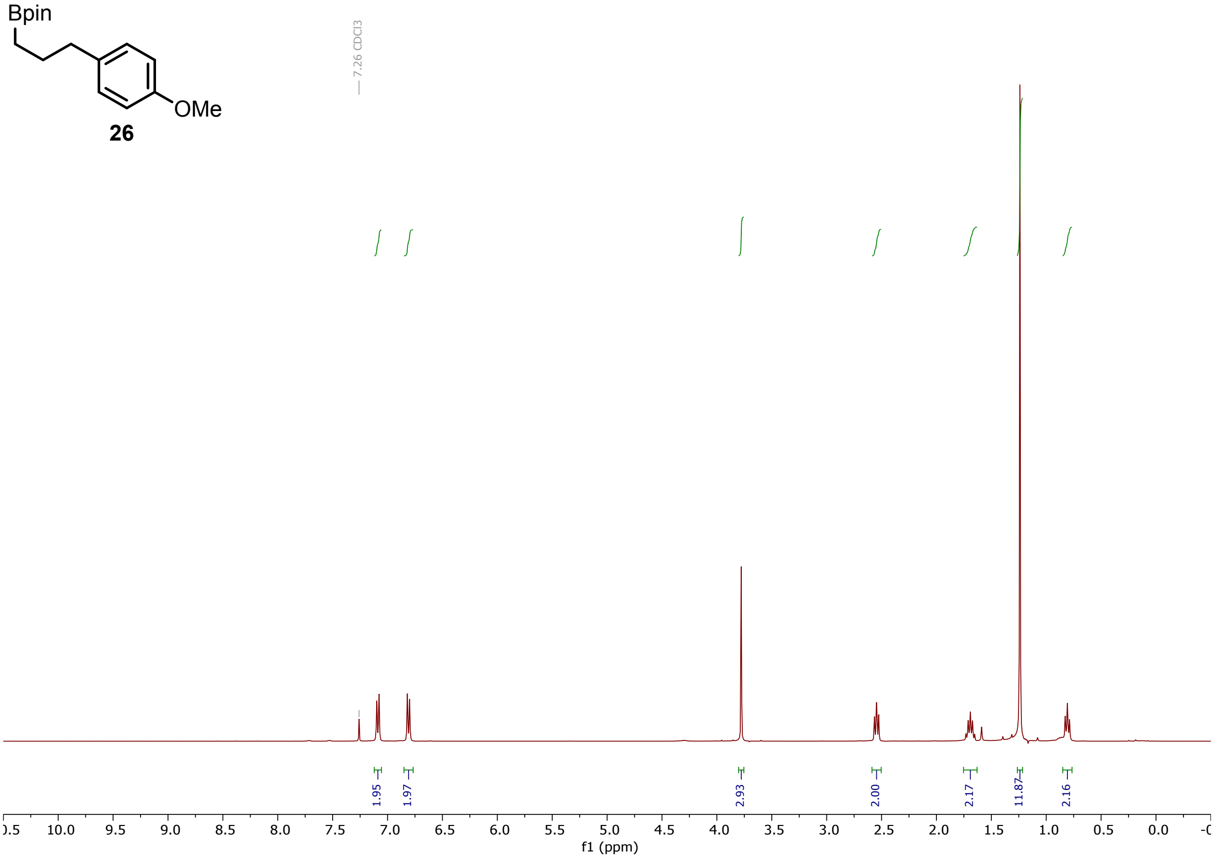

26, ^{13}C NMR (101 MHz, $CDCl_3$)

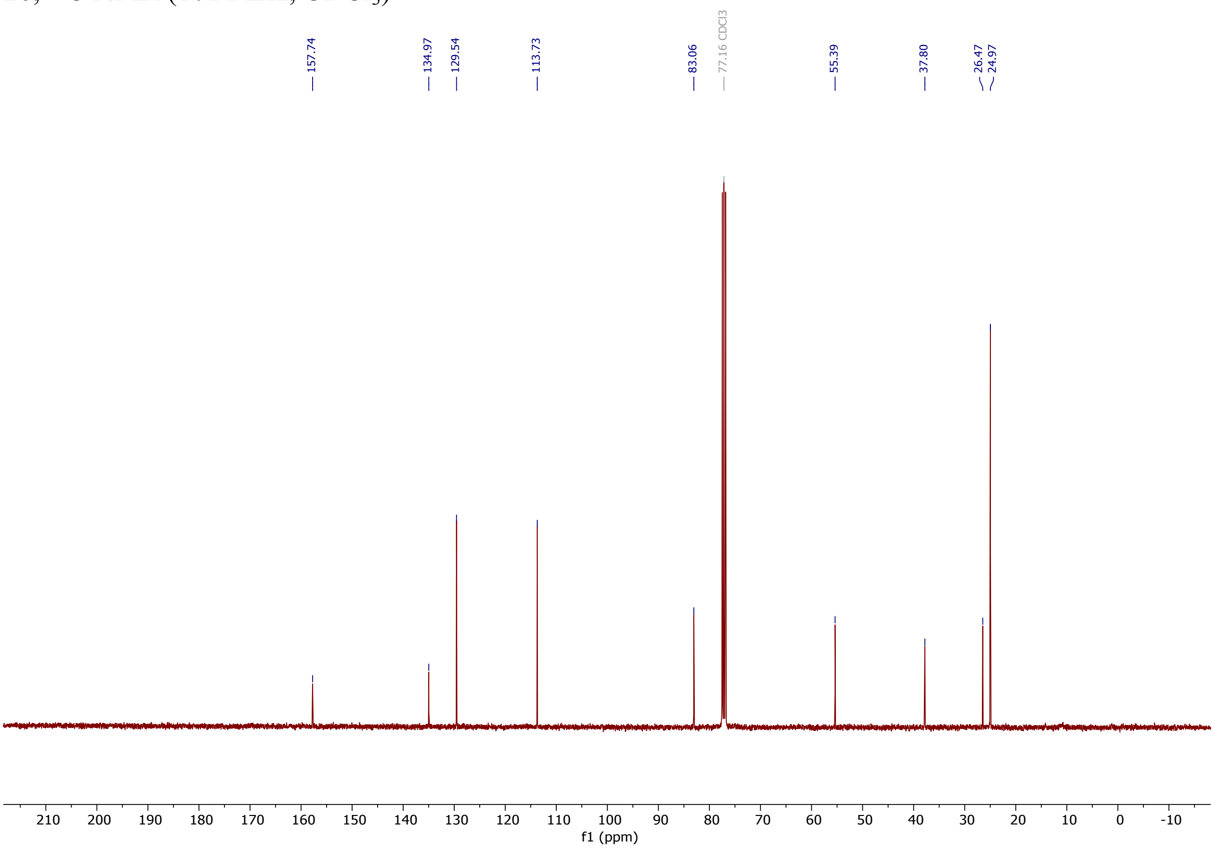

27, ¹H NMR (400 MHz, $CDCl_3$)

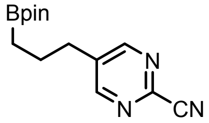
27

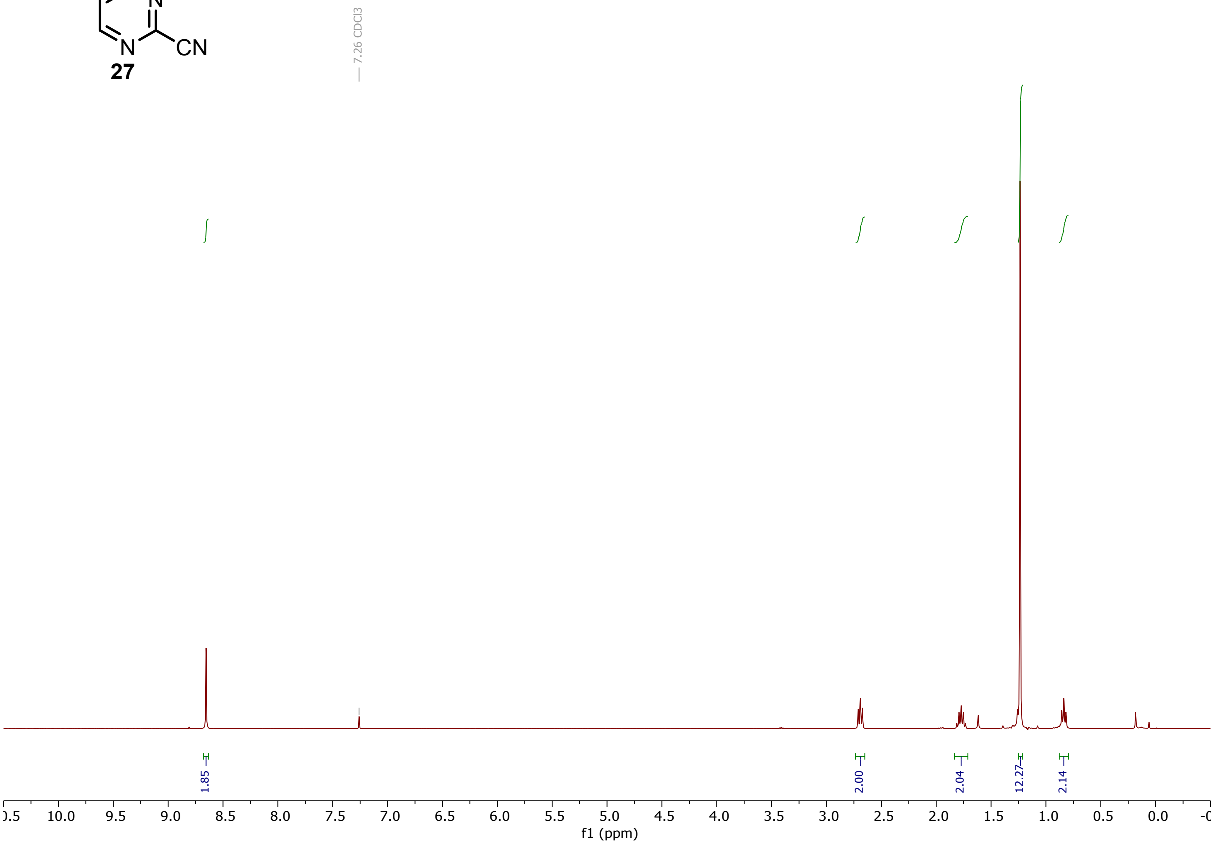

27, ¹³C NMR (101 MHz, $CDCl_3$)

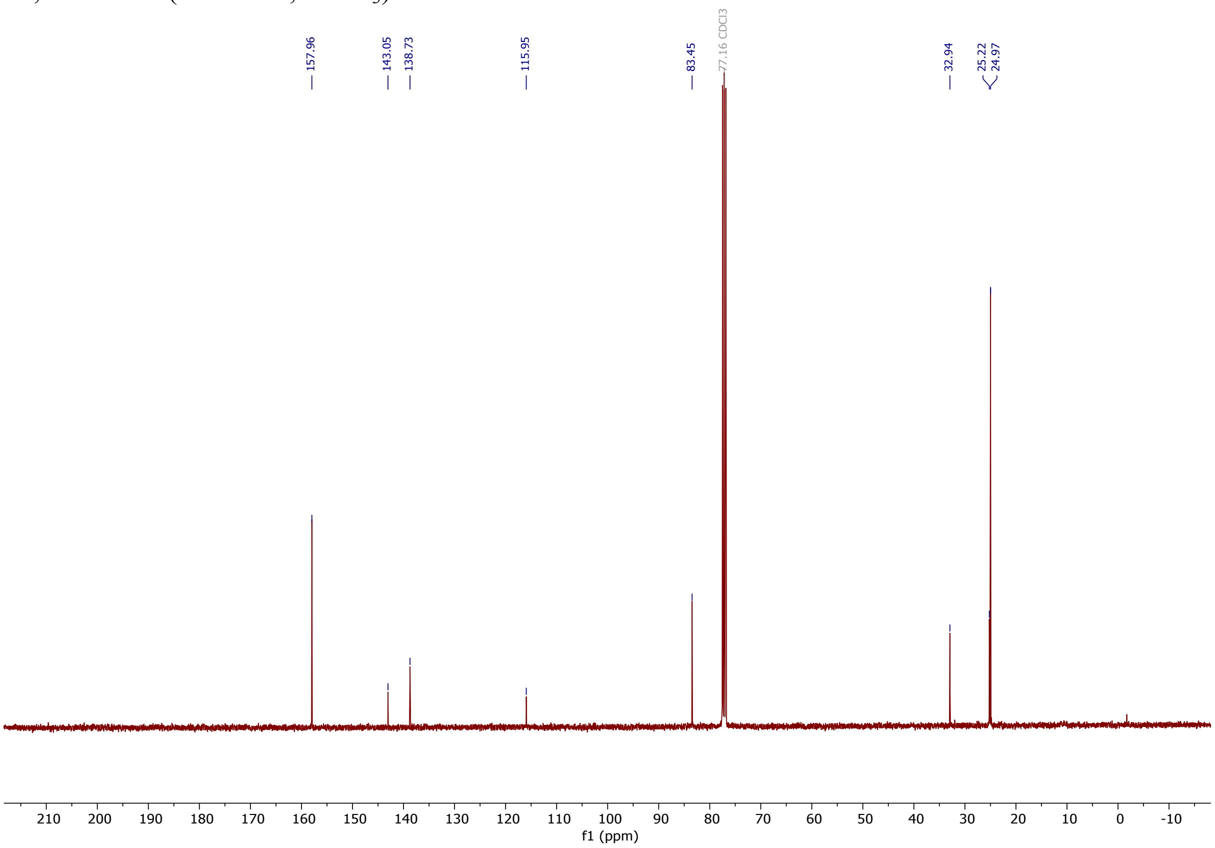

28, ^{1}H NMR (400 MHz, $CDCl_3$)

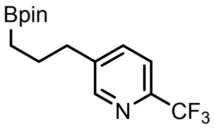

28

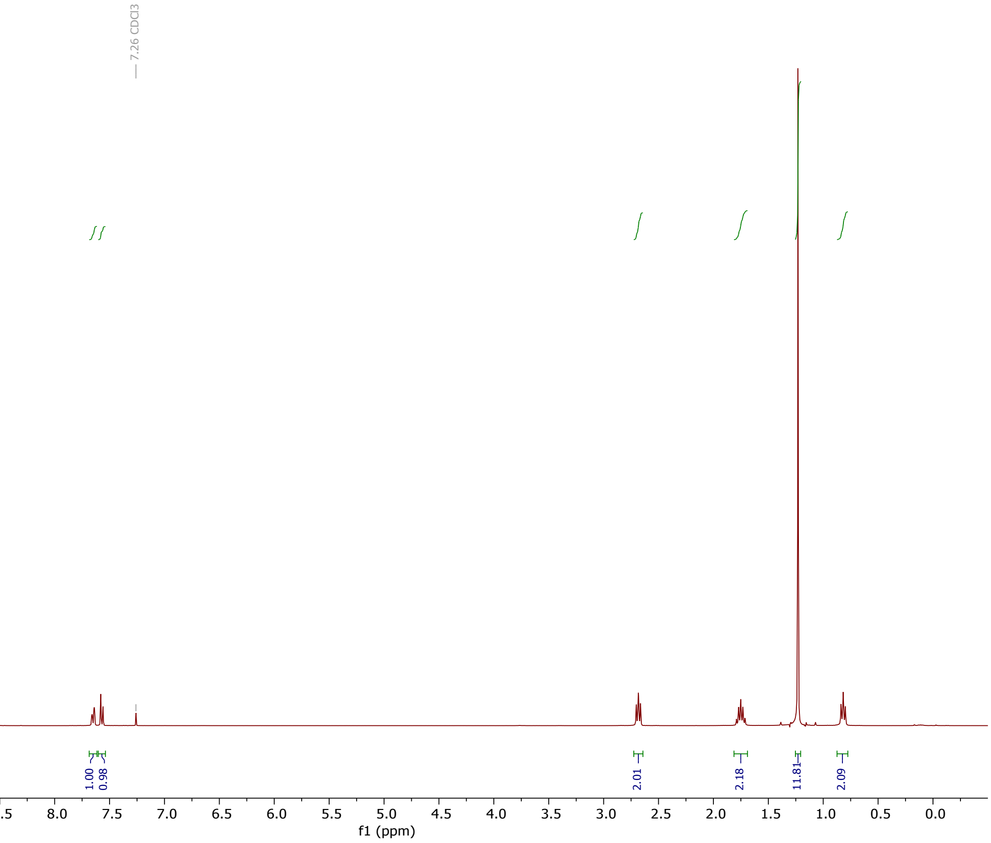

28, ^{13}C NMR (101 MHz, $CDCl_3$)

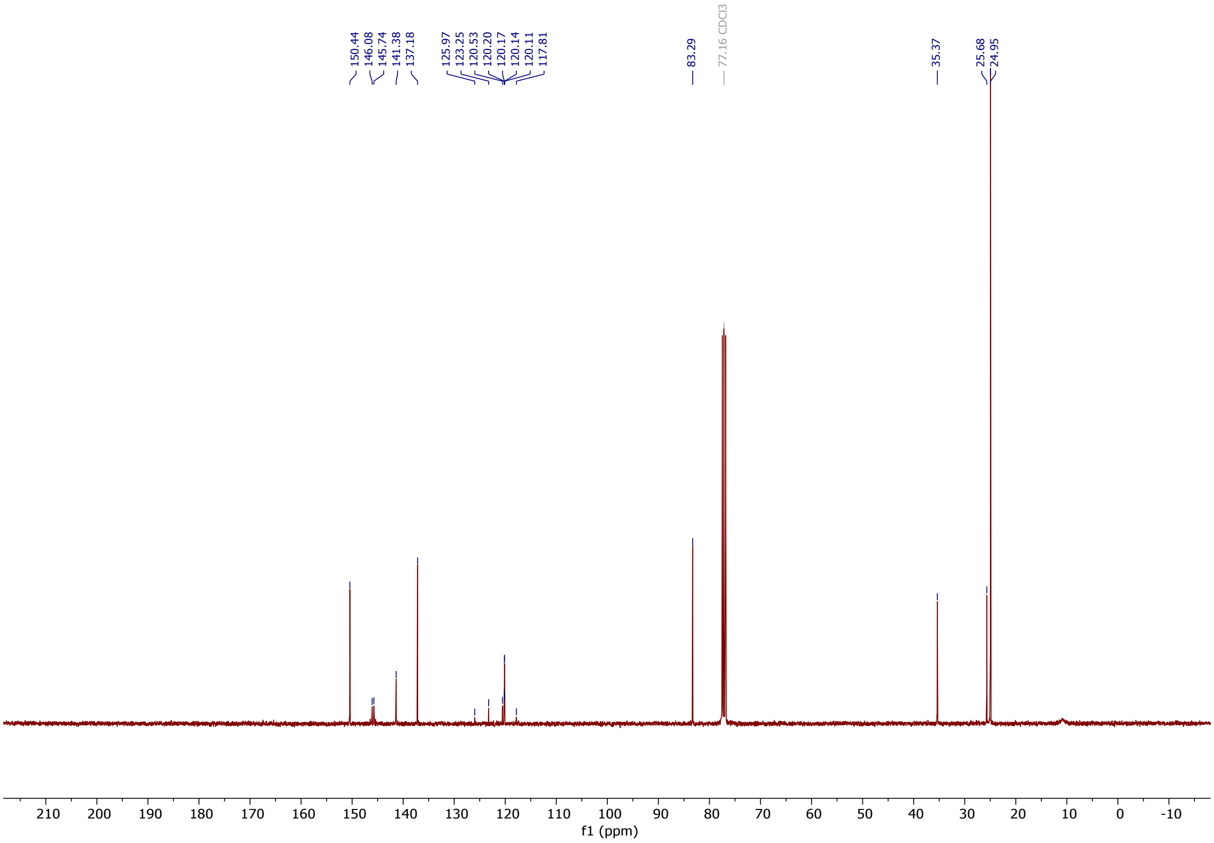

28, ^{19}F NMR (282 MHz, $CDCl_3$)

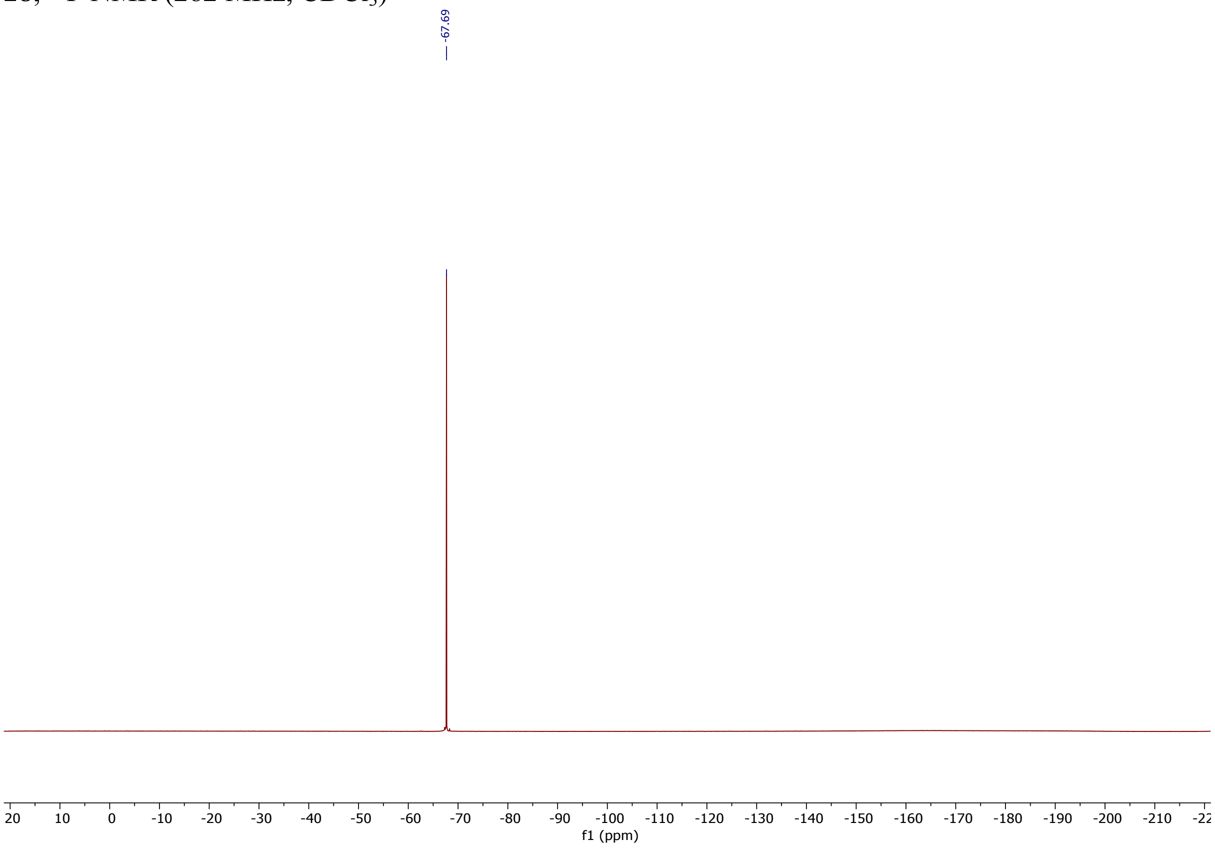

29, ^{1}H NMR (400 MHz, $CDCl_3$)

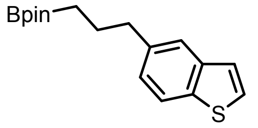

29

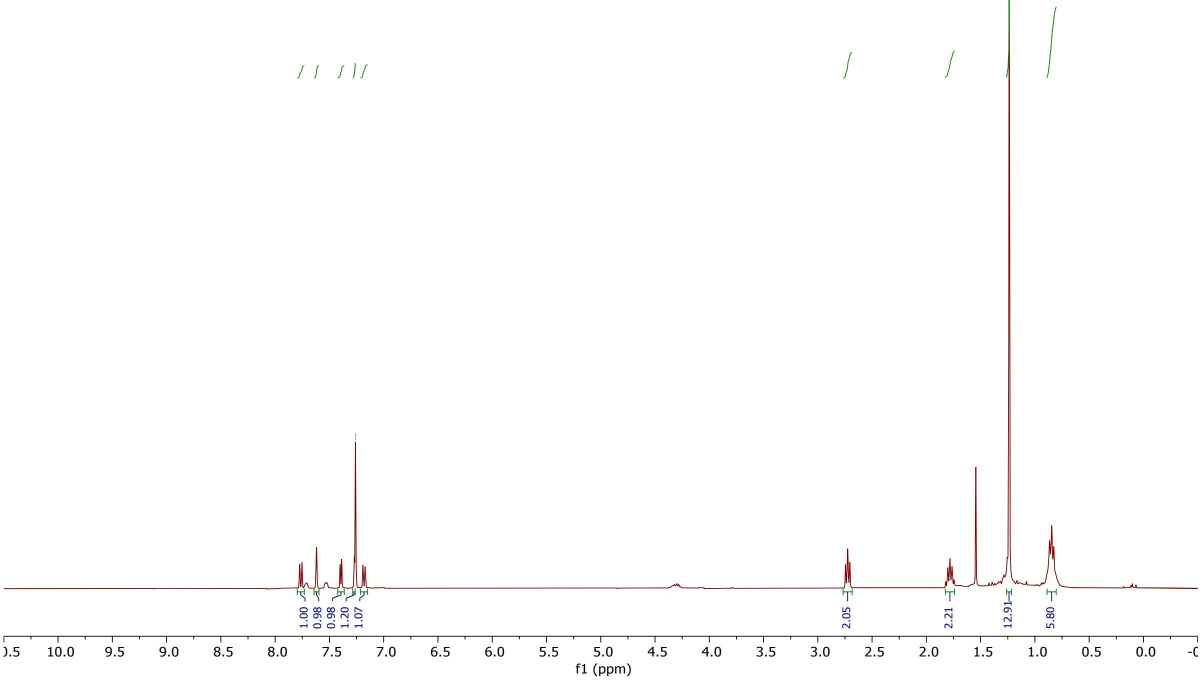

29, ^{13}C NMR (101 MHz, $CDCl_3$)

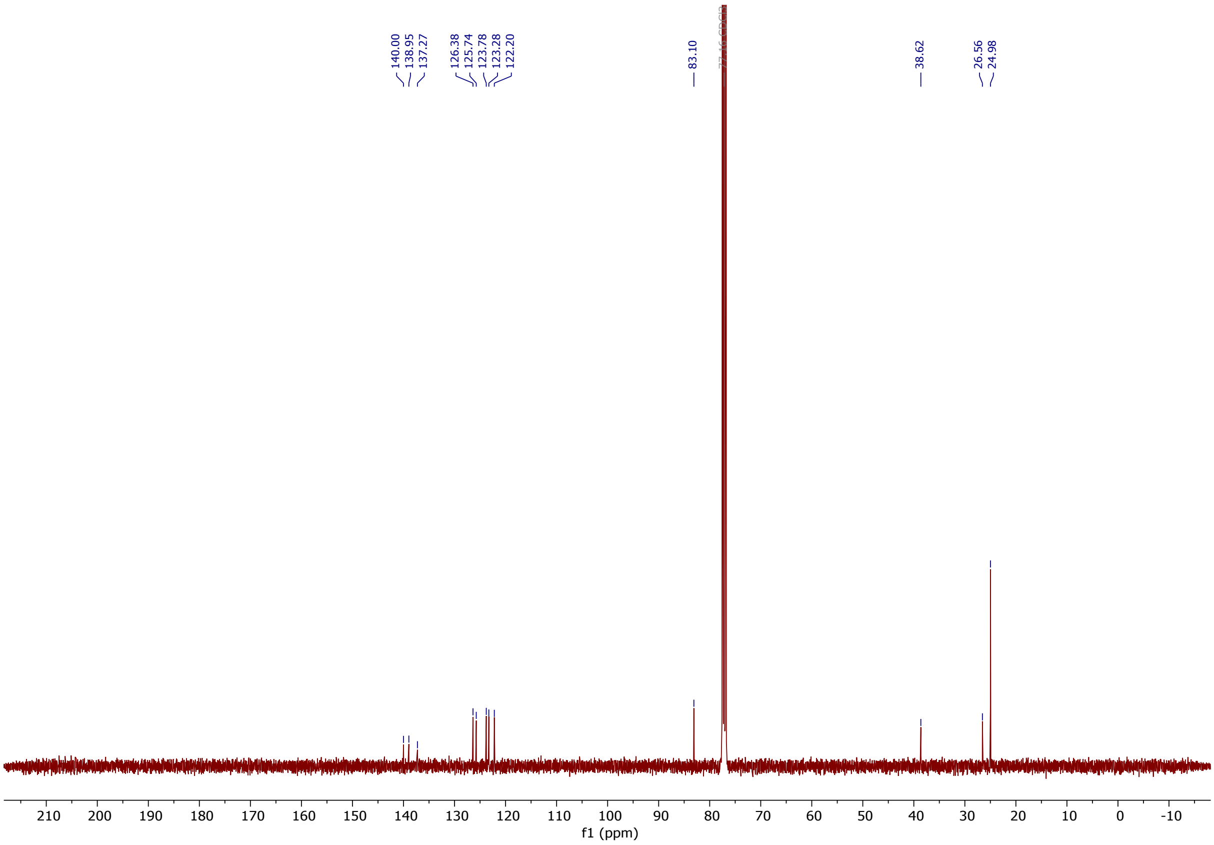

30, ^{1}H NMR (400 MHz, $CDCl_3$)

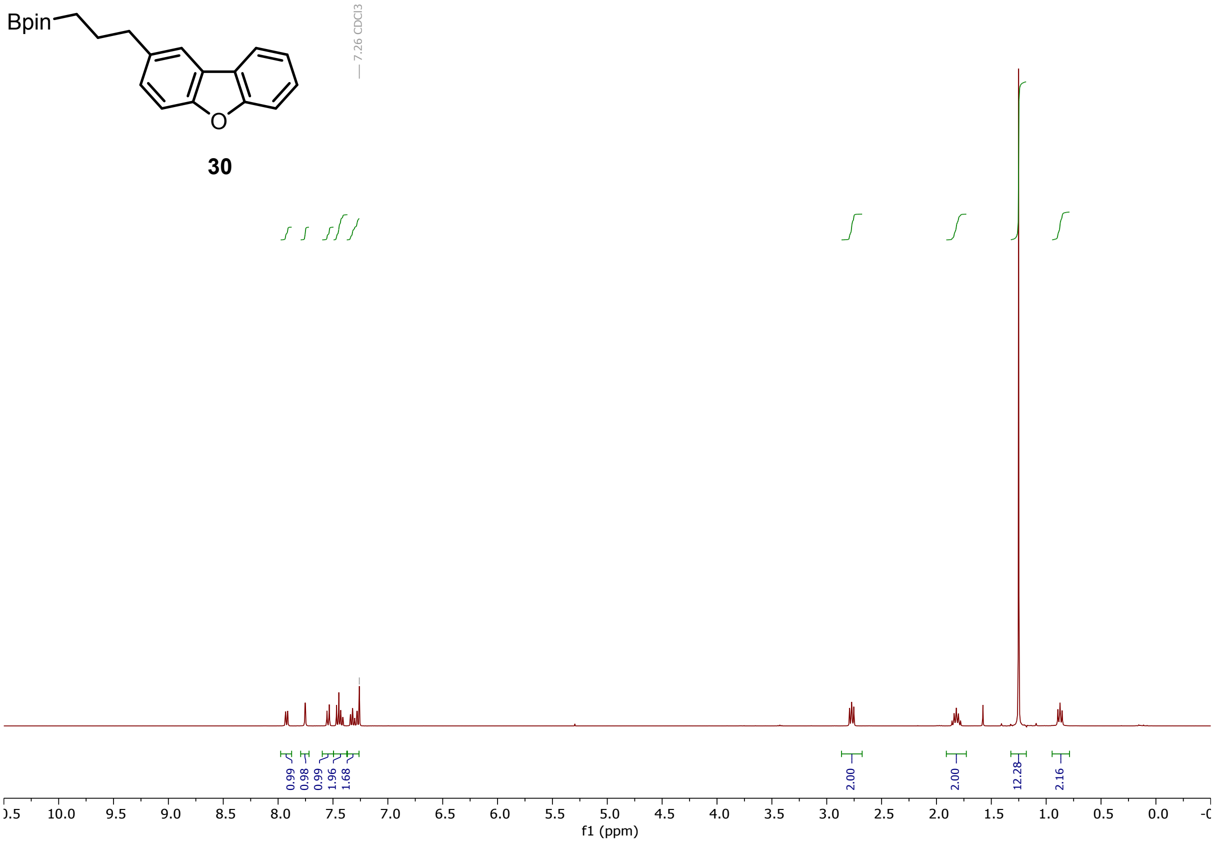

30, ^{13}C NMR (101 MHz, $CDCl_3$)

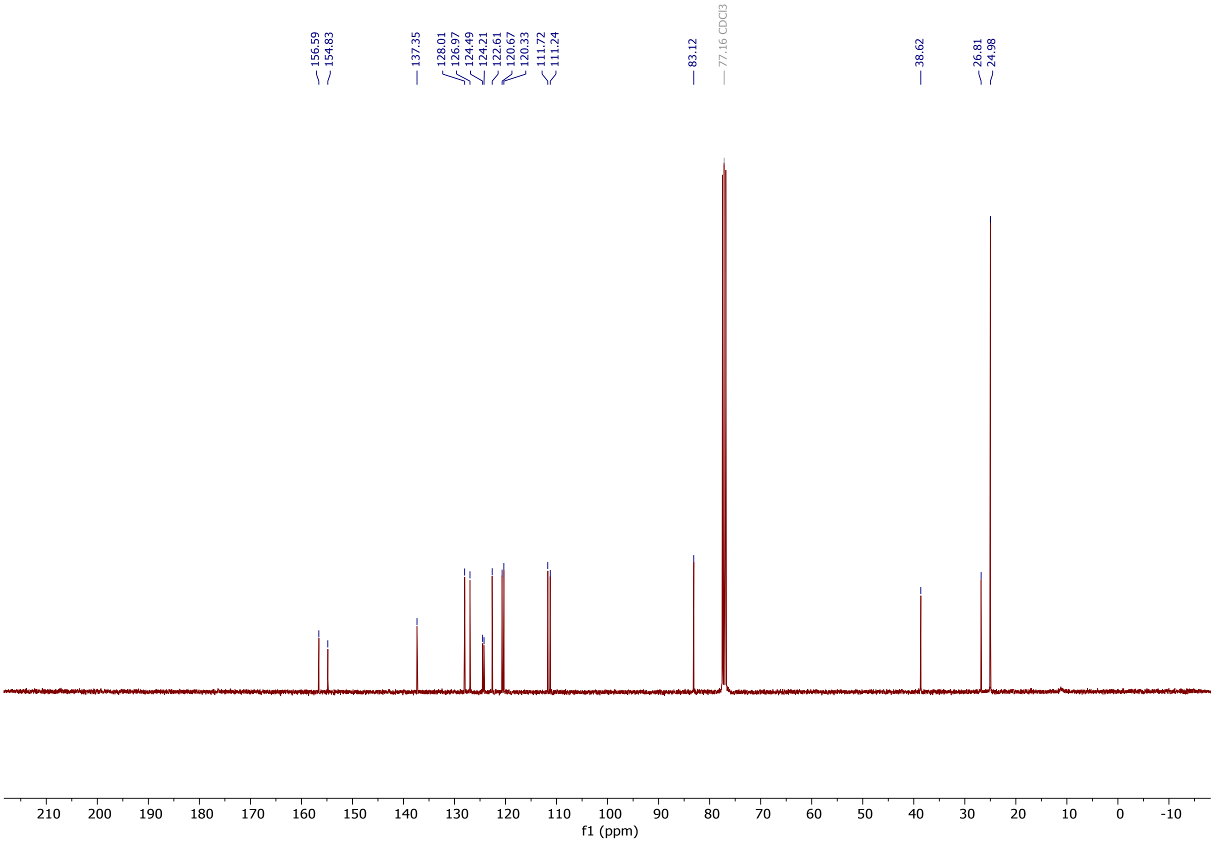

31, ^{1}H NMR (400 MHz, $CDCl_3$)

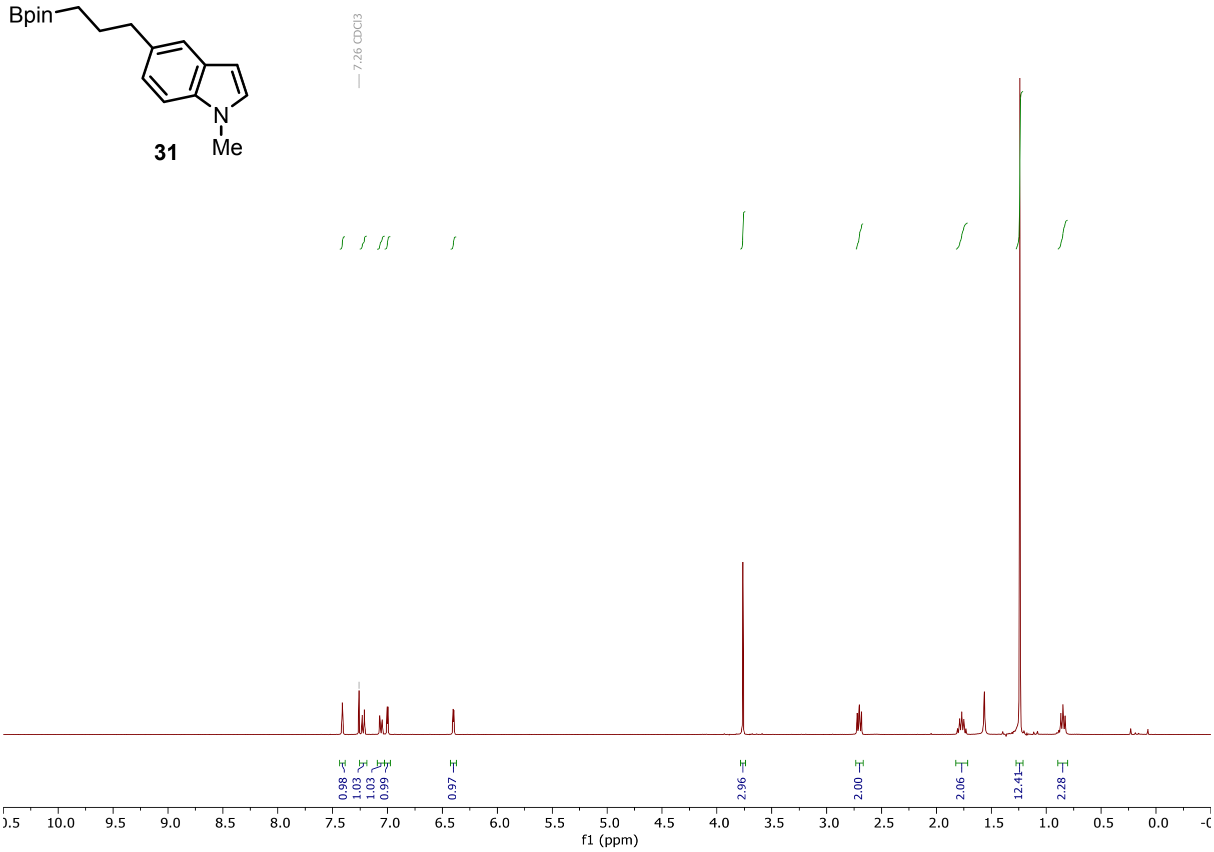

31, ^{13}C NMR (101 MHz, $CDCl_3$)

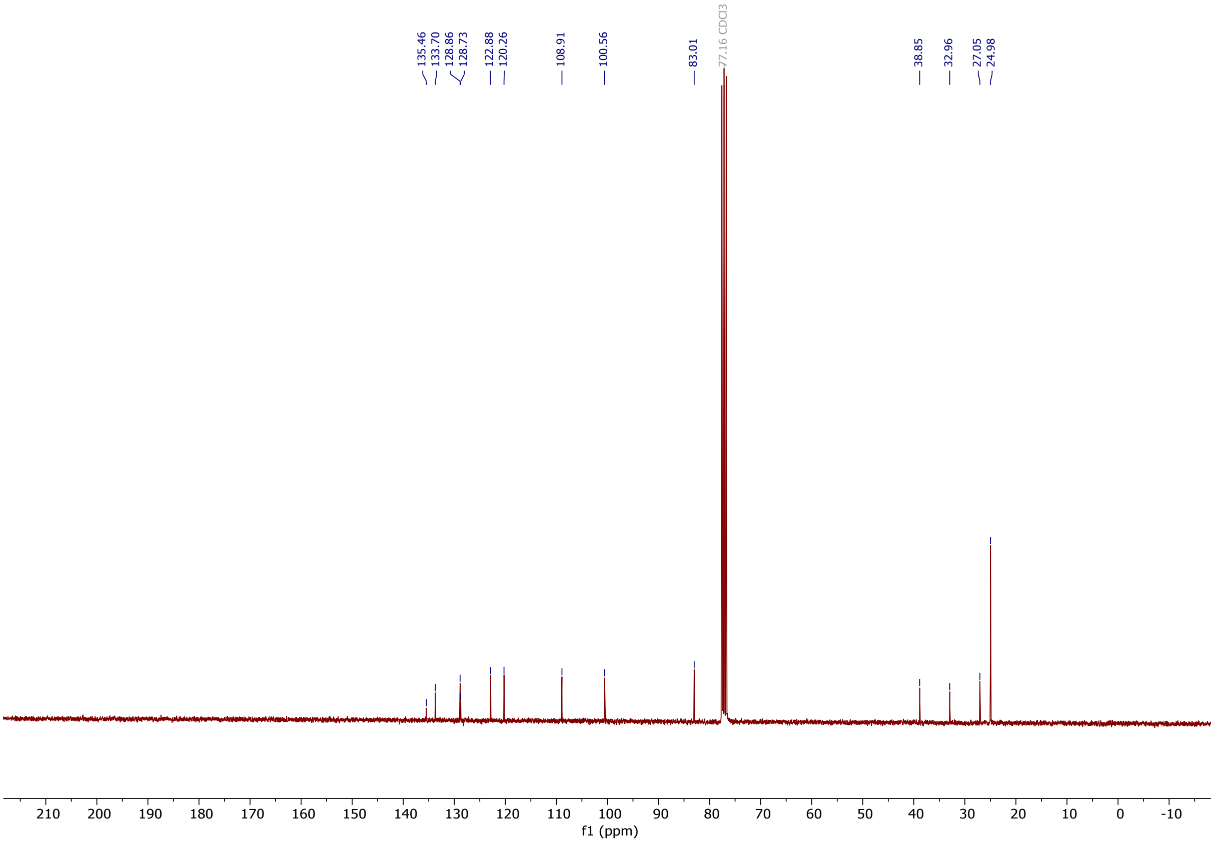

32, ^{1}H NMR (400 MHz, $CDCl_3$)

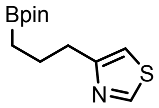

32

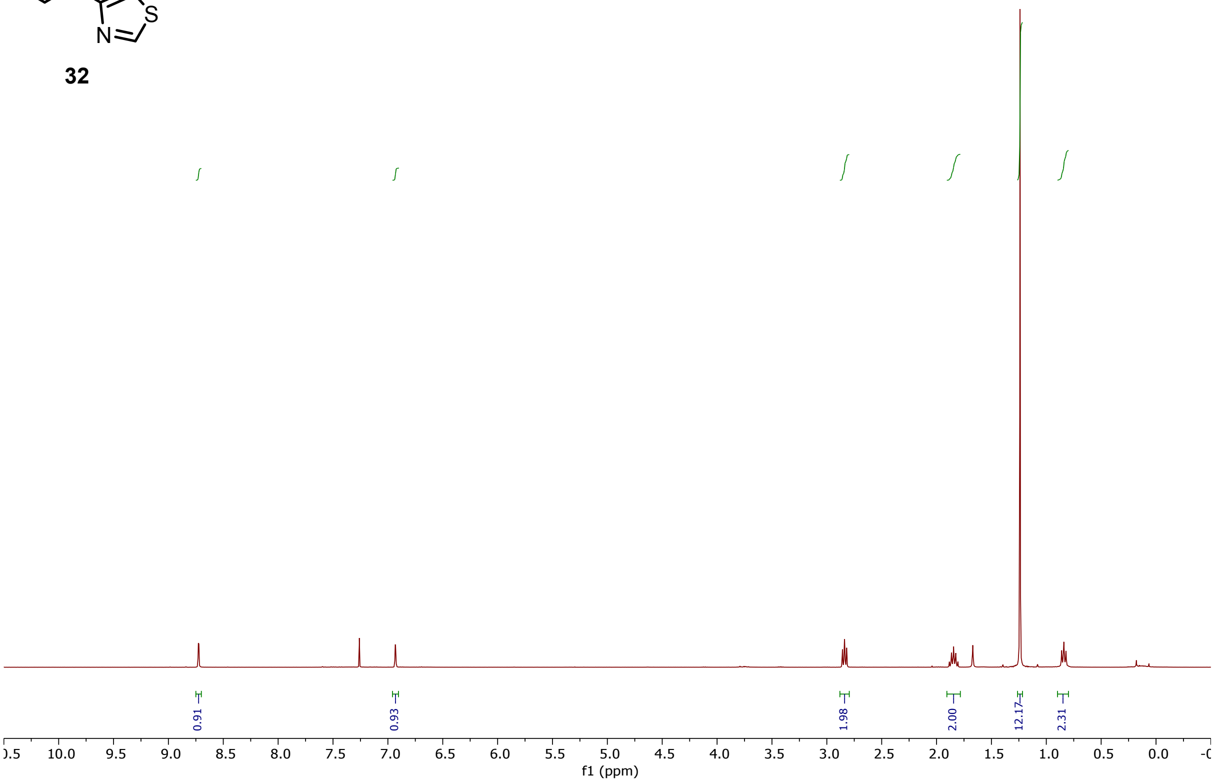

32, ^{13}C NMR (101 MHz, $CDCl_3$)

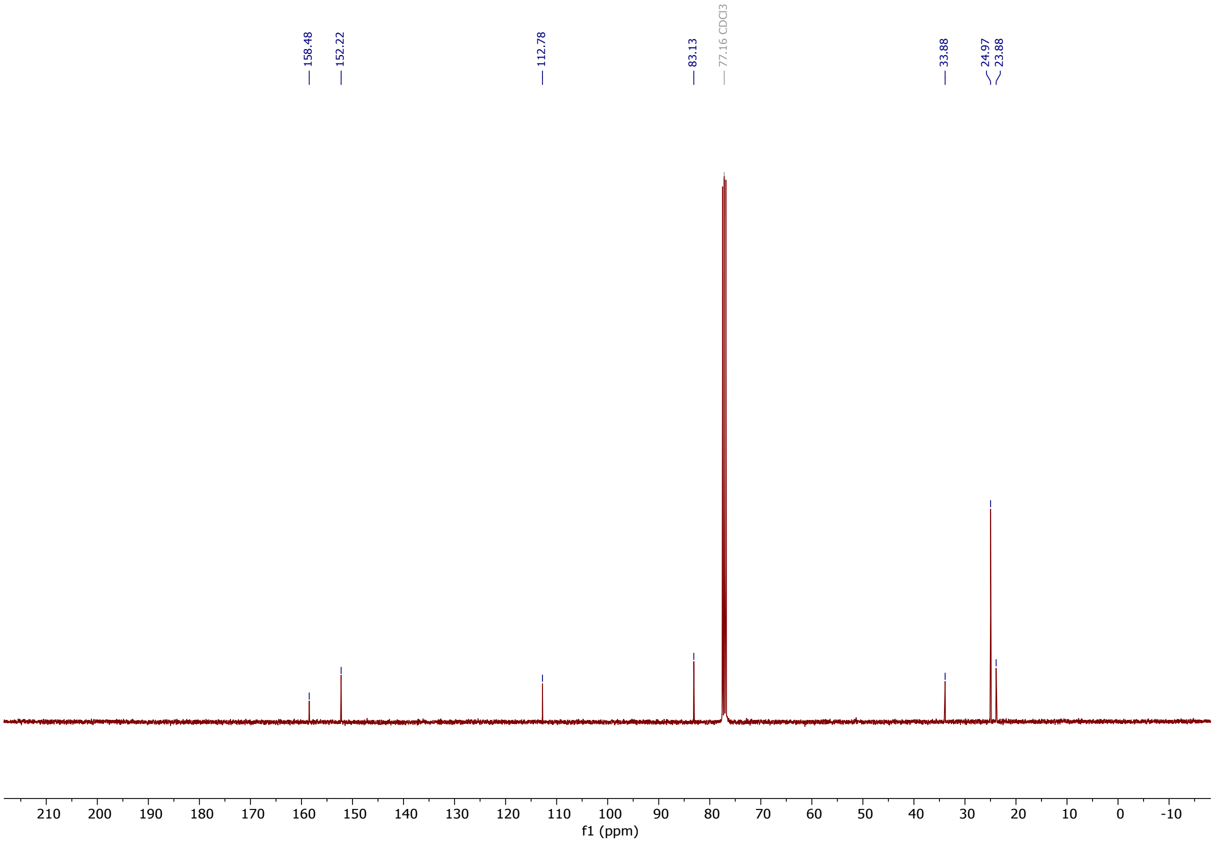

33, ^{1}H NMR (400 MHz, $CDCl_3$)

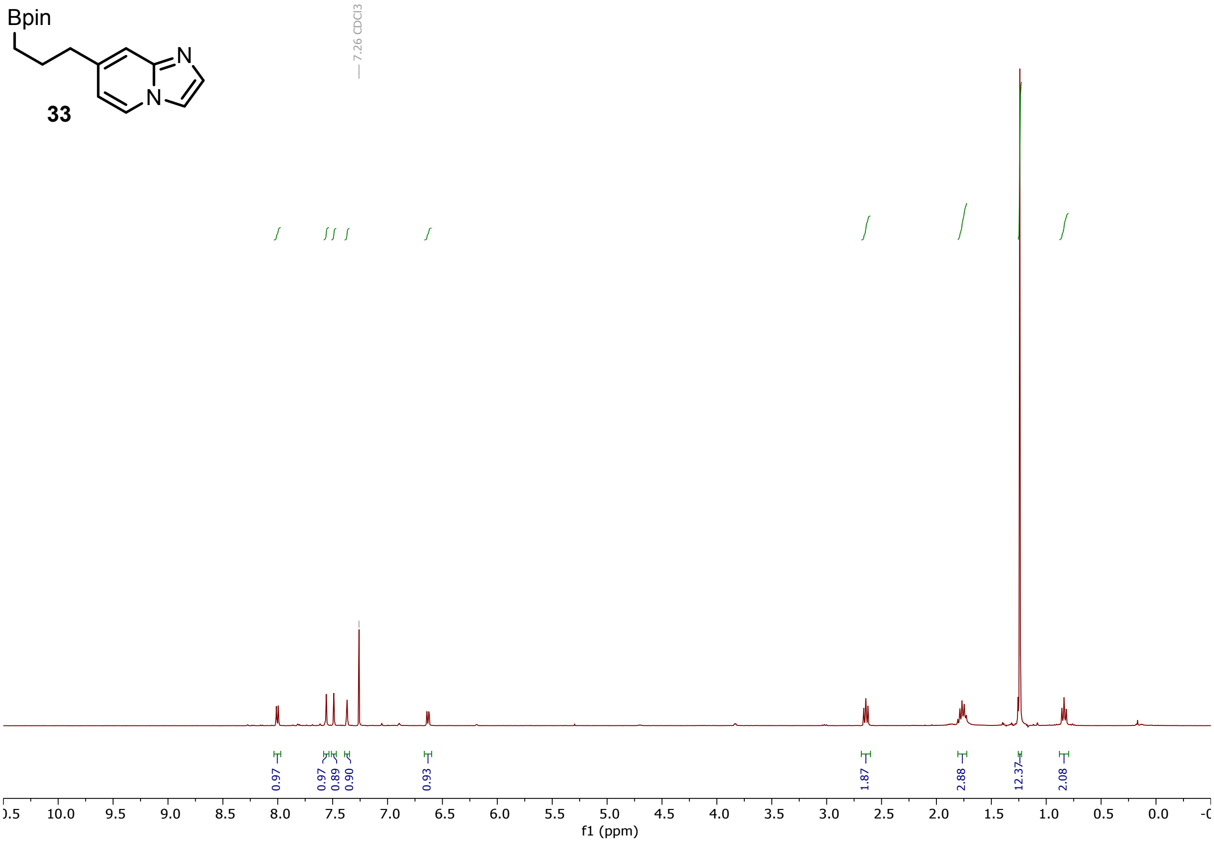

33, ^{13}C NMR (101 MHz, $CDCl_3$)

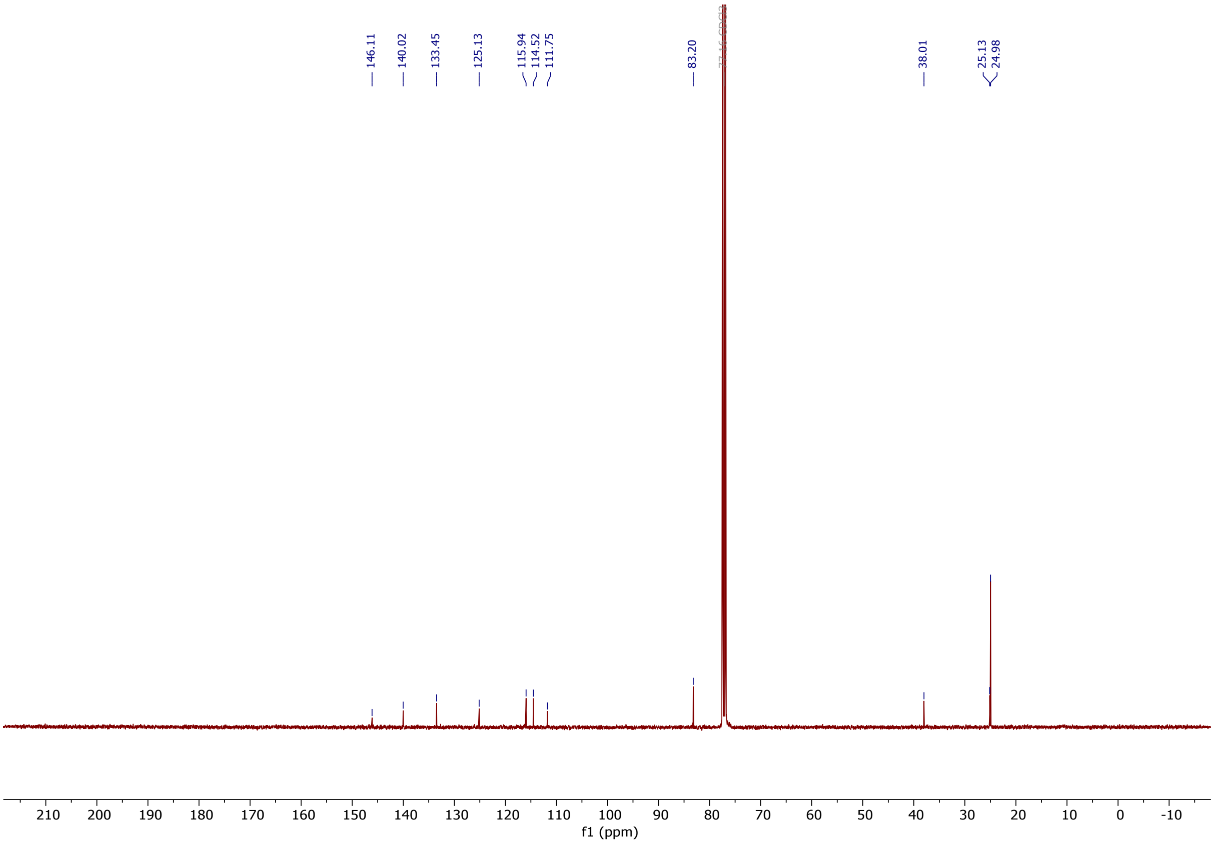

34, ¹H NMR (400 MHz, $CDCl_3$)

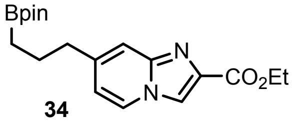

34

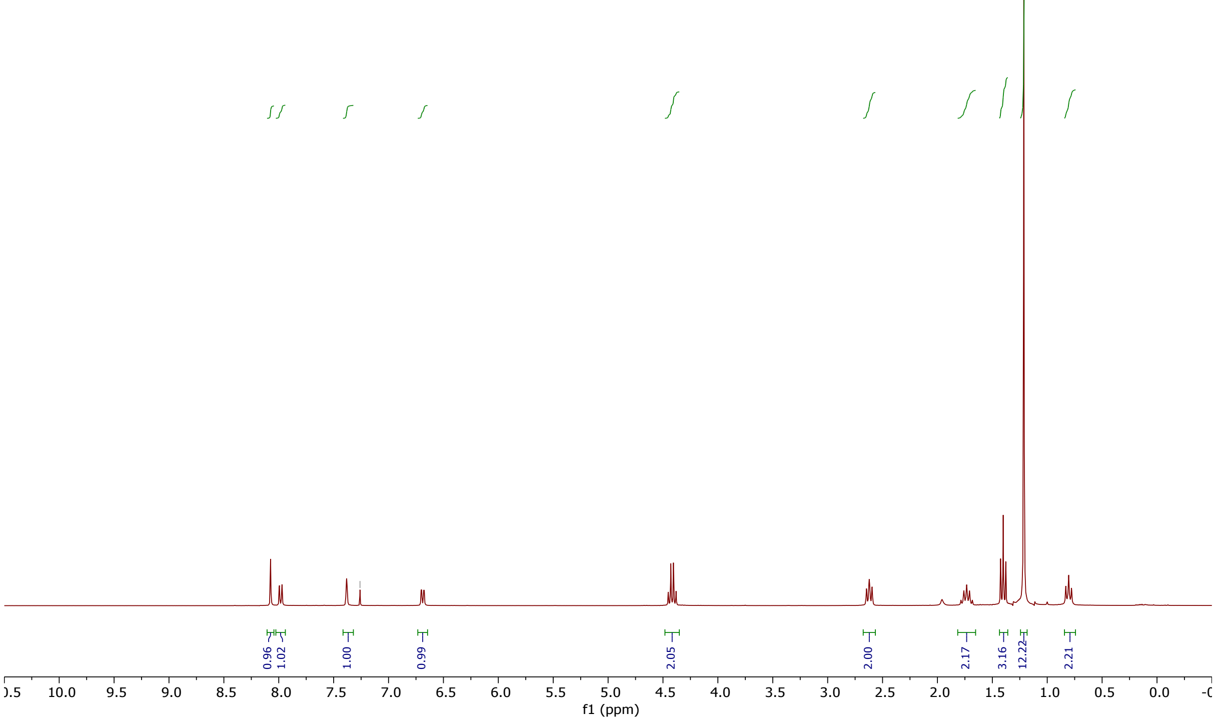

34, ¹³C NMR (101 MHz, $CDCl_3$)

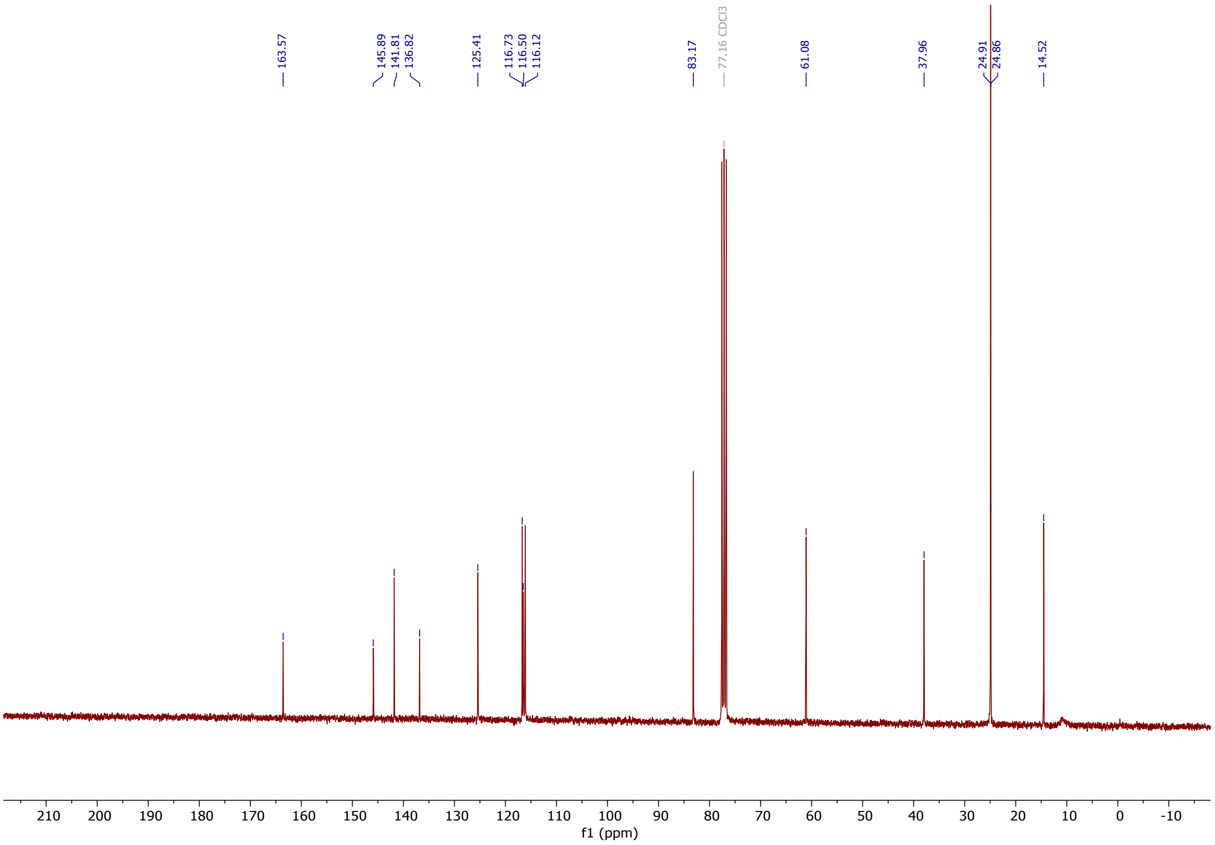

35, ^{1}H NMR (400 MHz, $CDCl_3$)

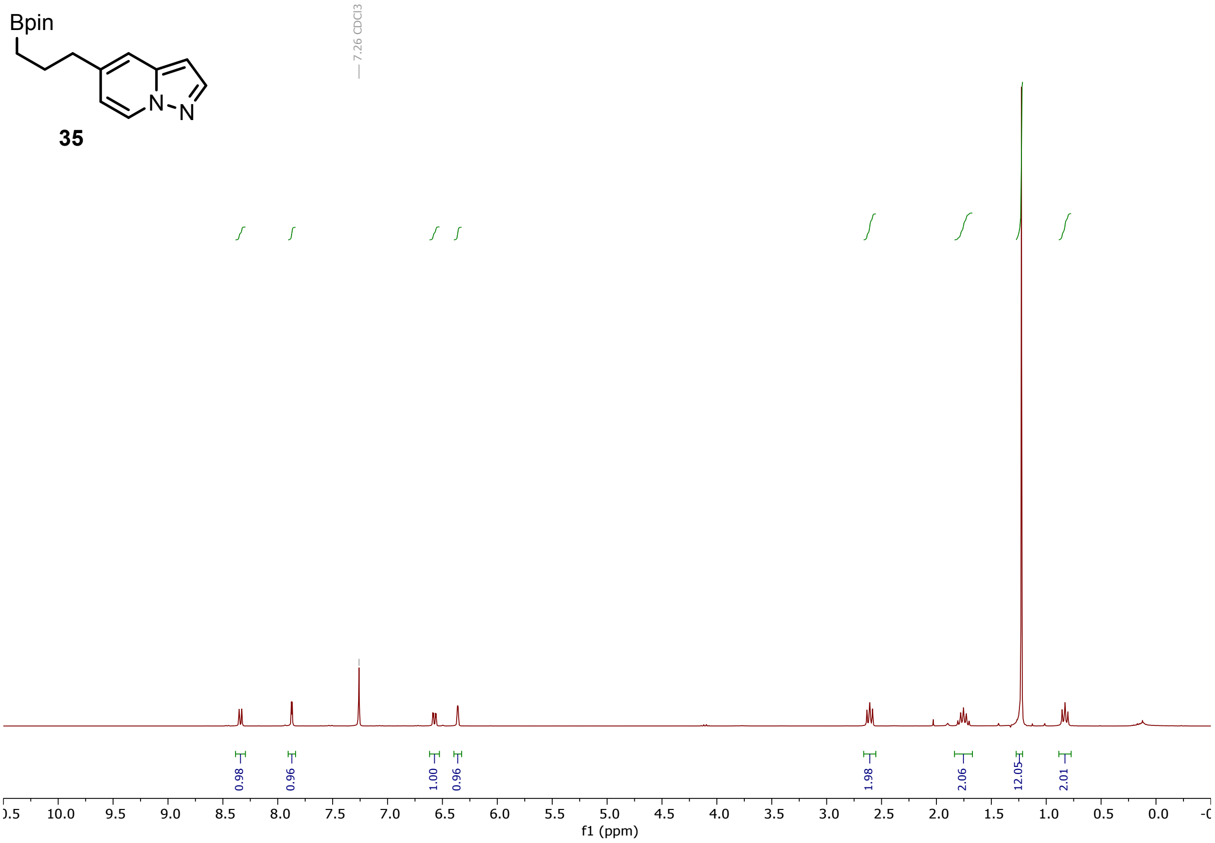

35, ^{13}C NMR (101 MHz, $CDCl_3$)

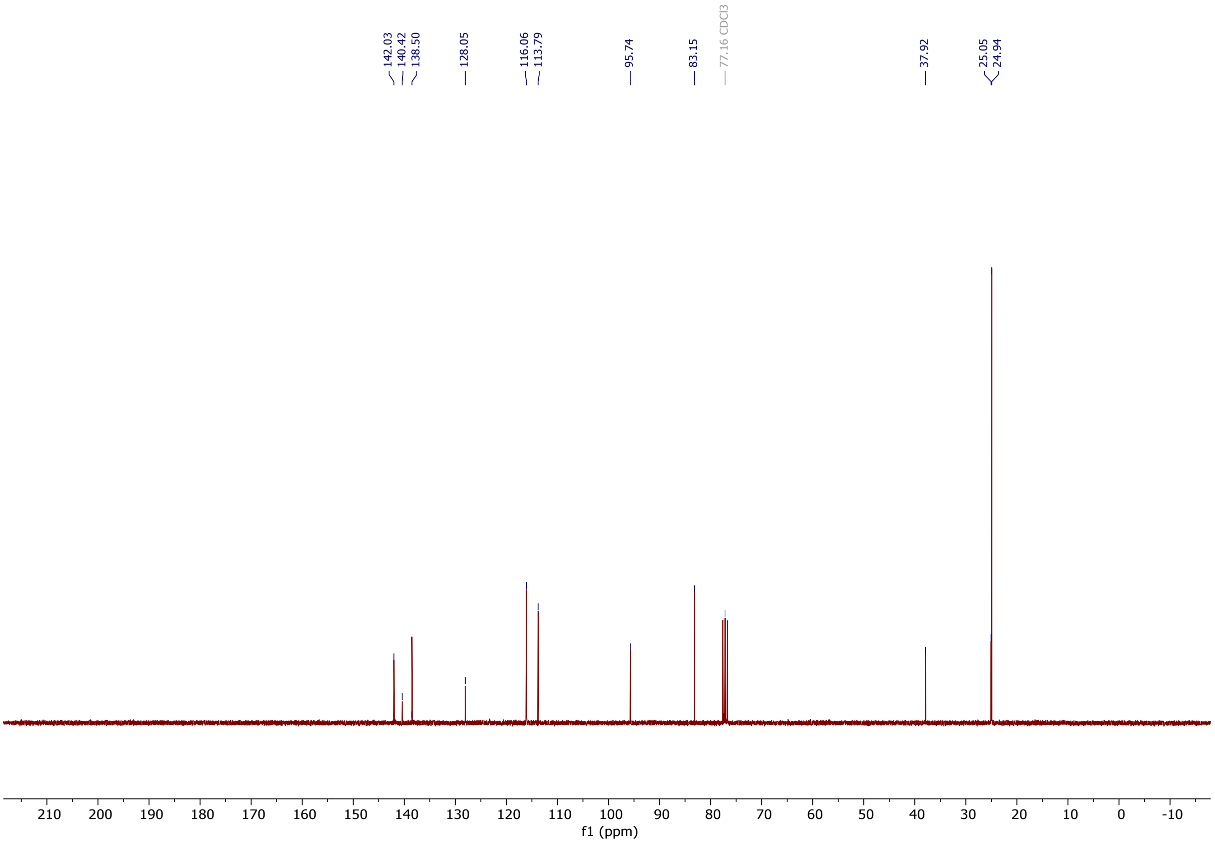

36, ^{1}H NMR (400 MHz, $CDCl_3$)

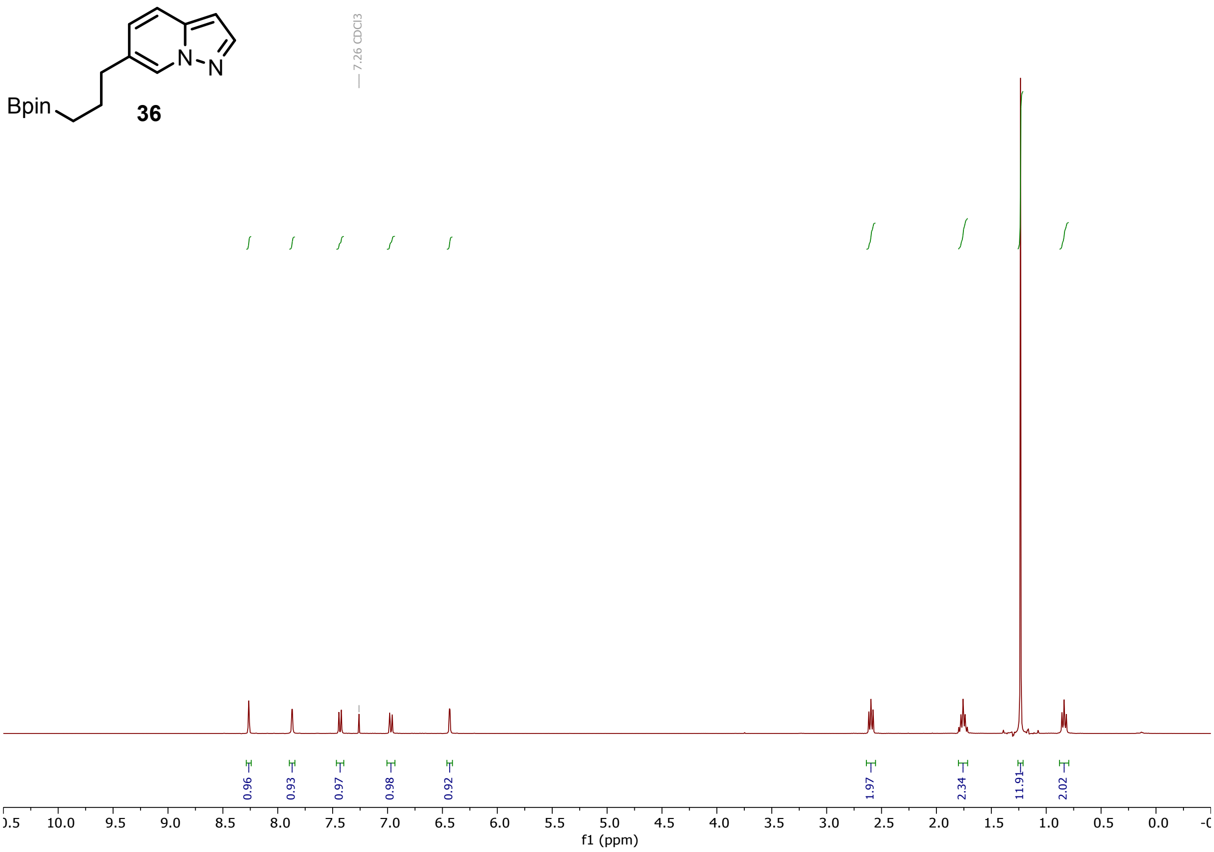

36, ^{13}C NMR (400 MHz, $CDCl_3$)

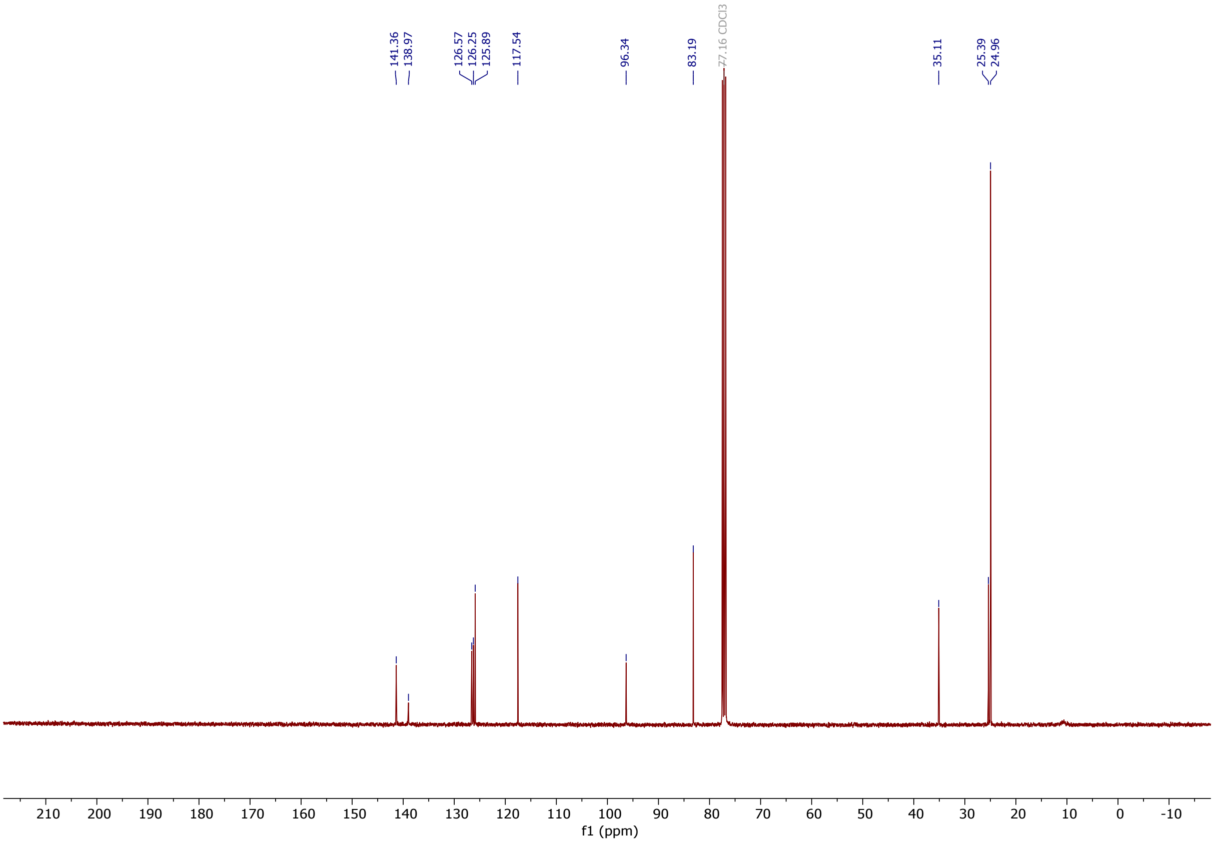

38, ^{1}H NMR (400 MHz, $CDCl_3$)

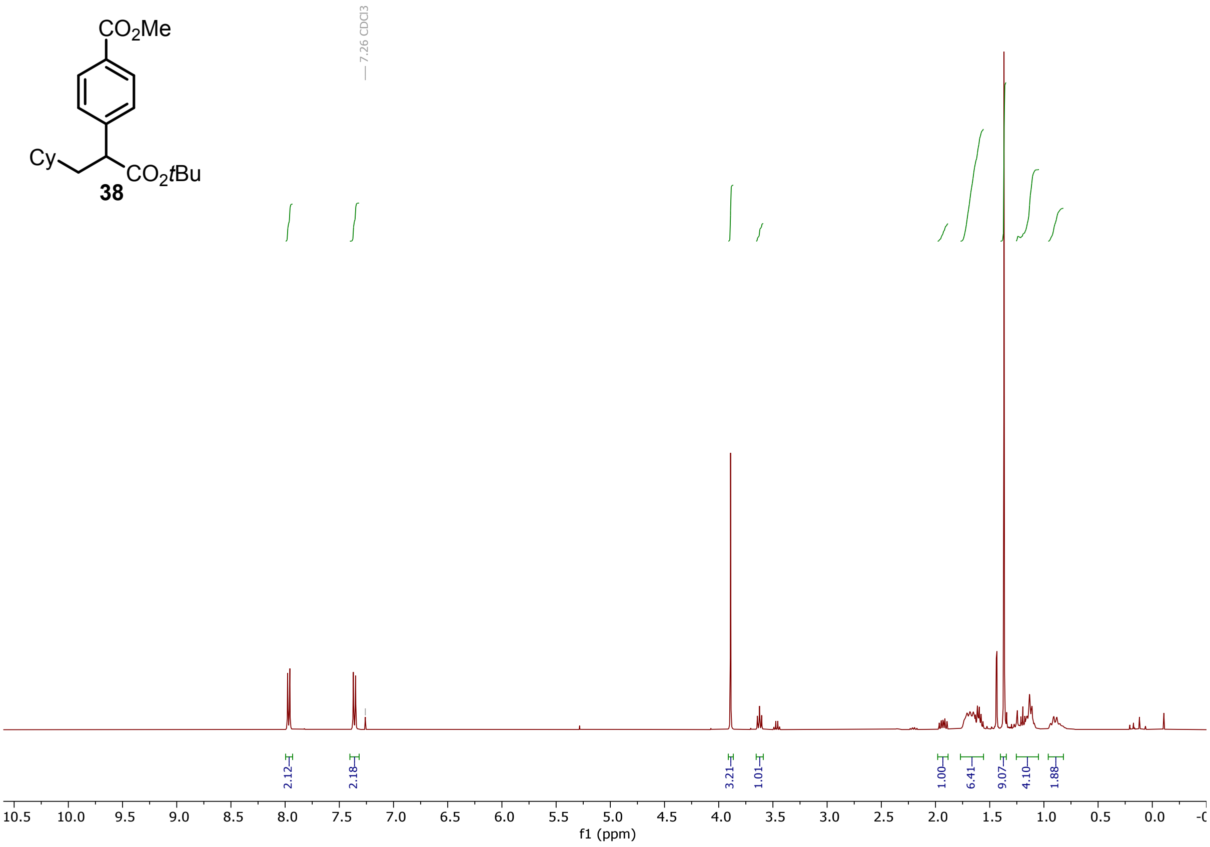

38, ^{13}C NMR (101 MHz, $CDCl_3$)

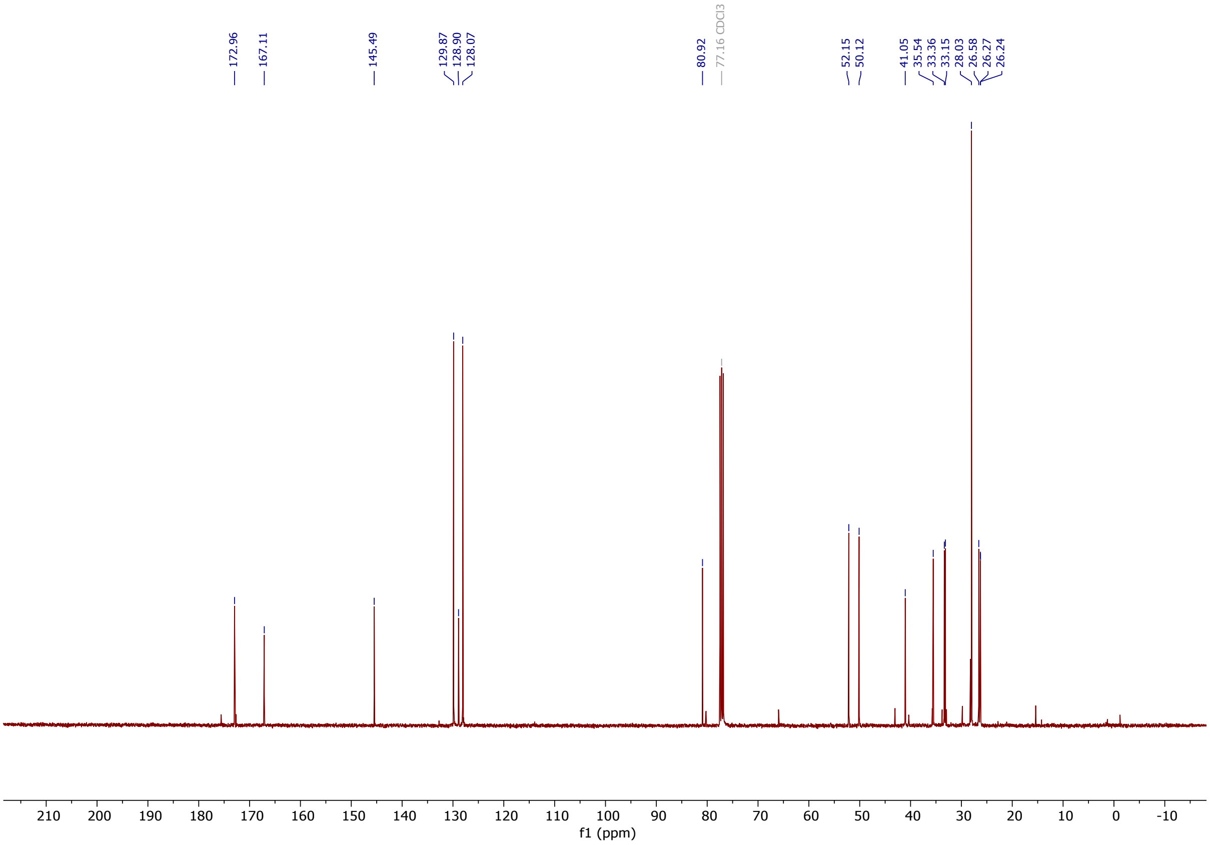

39, ^{1}H NMR (400 MHz, $CDCl_3$)

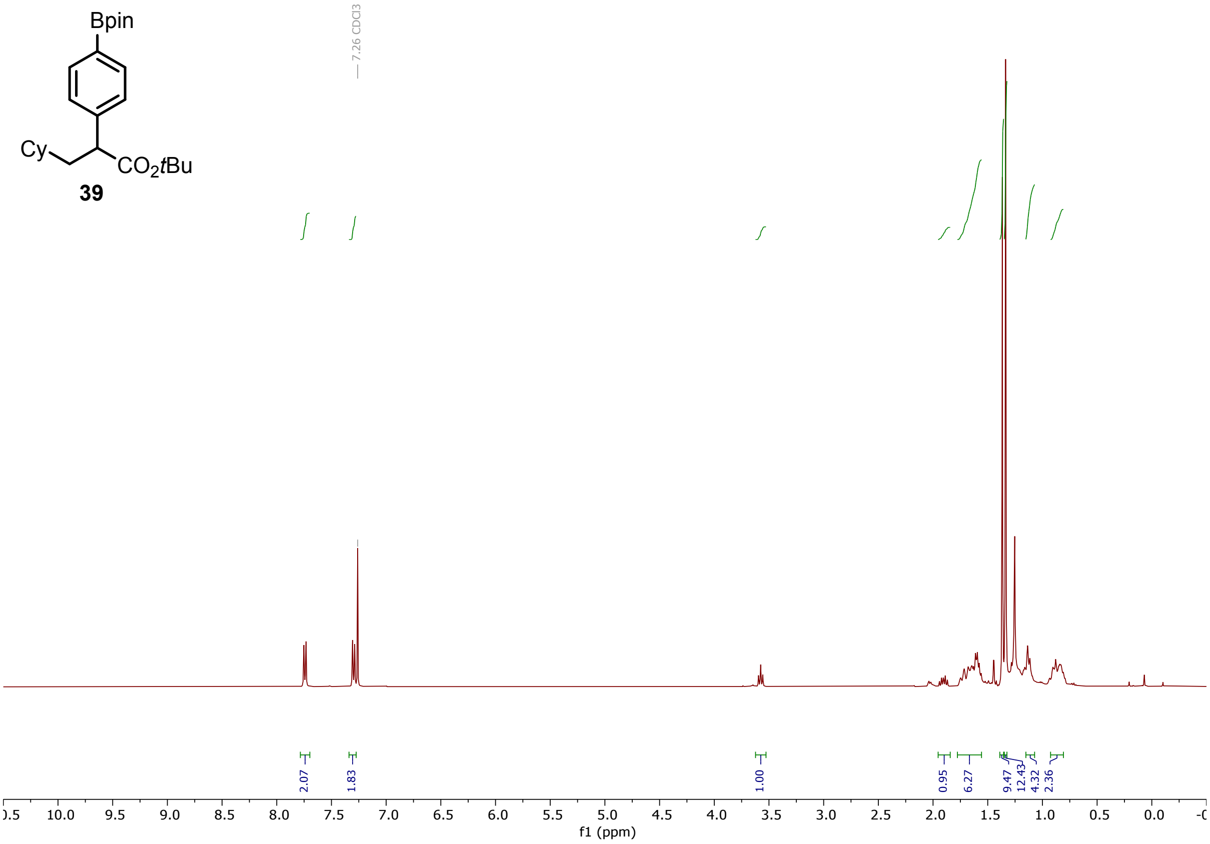

39, ^{13}C NMR (101 MHz, $CDCl_3$)

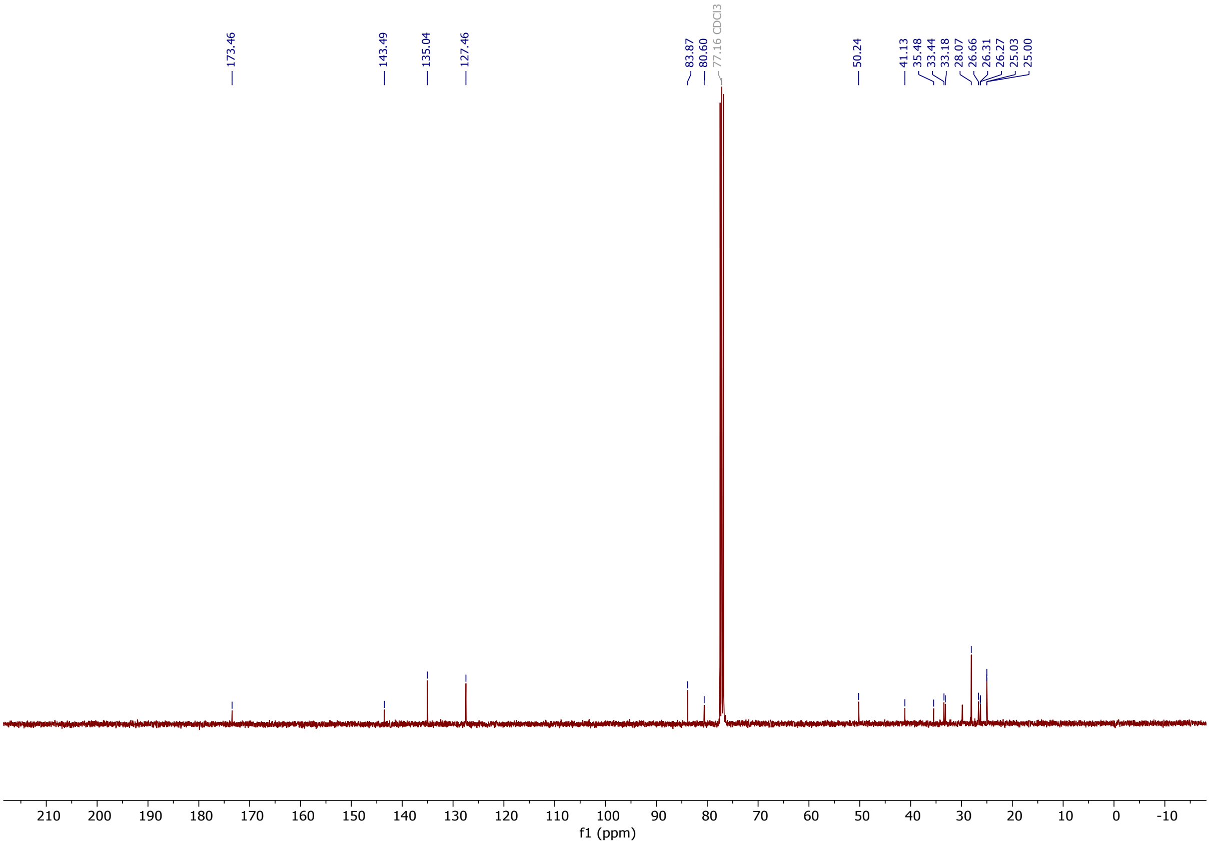

40, ^{1}H NMR (400 MHz, $CDCl_3$)

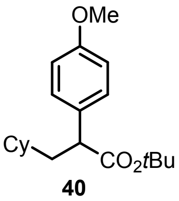

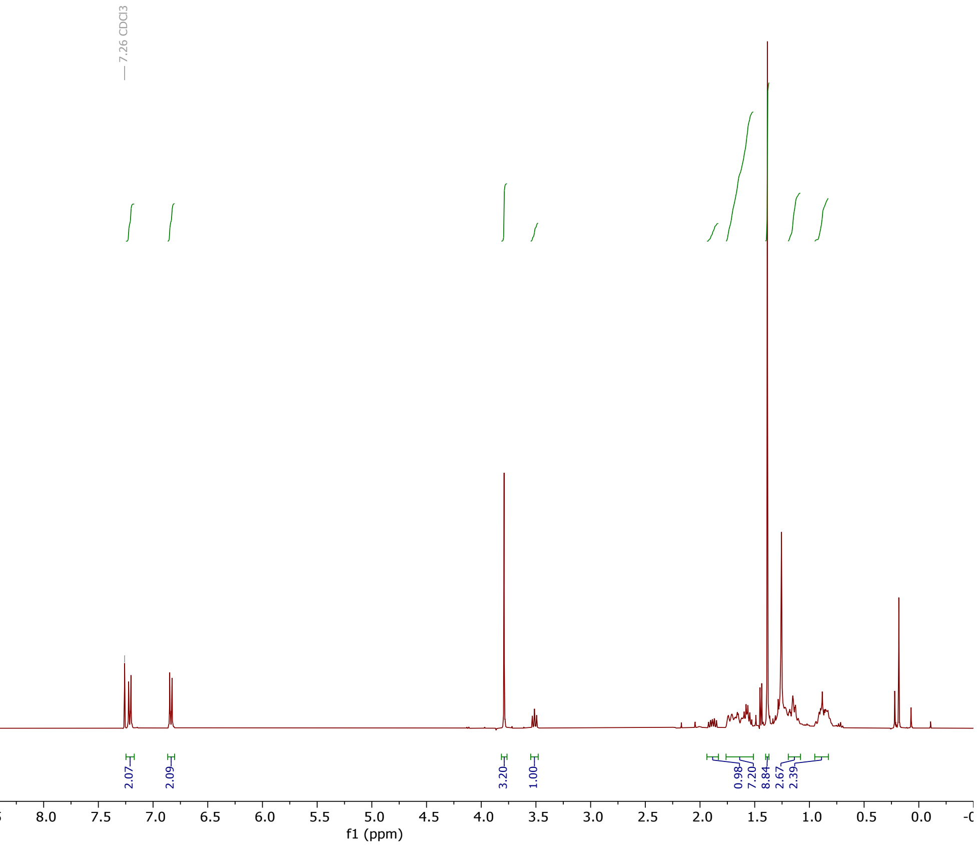

40, ^{13}C NMR (101 MHz, $CDCl_3$)

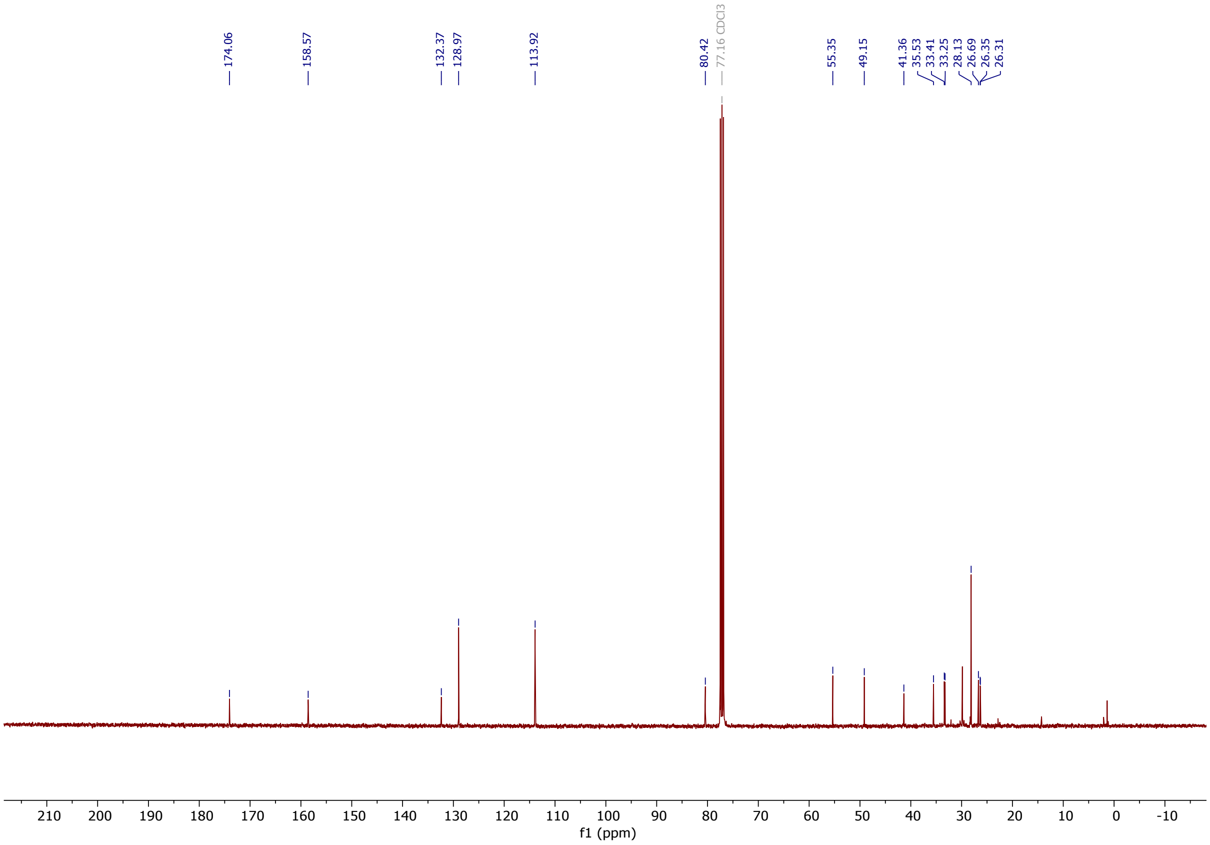

41, ^{1}H NMR (400 MHz, $CDCl_3$)

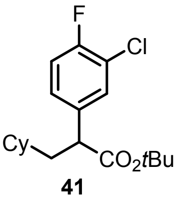

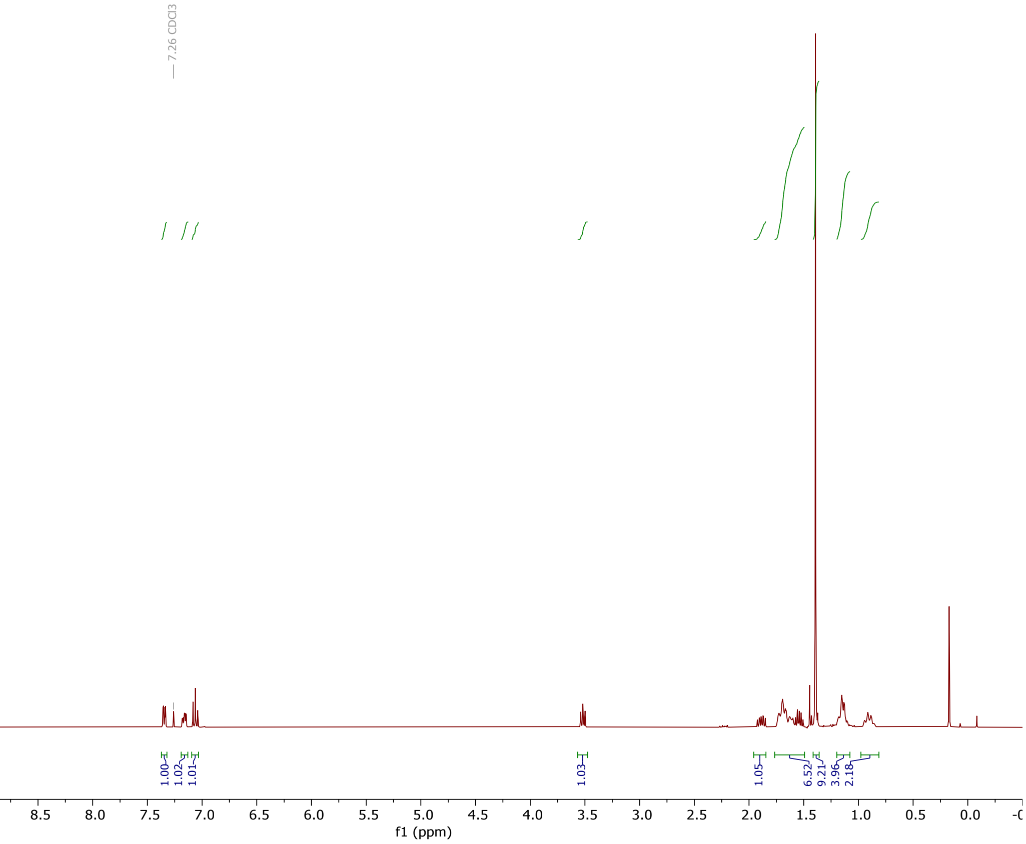

41, ^{13}C NMR (101 MHz, $CDCl_3$)

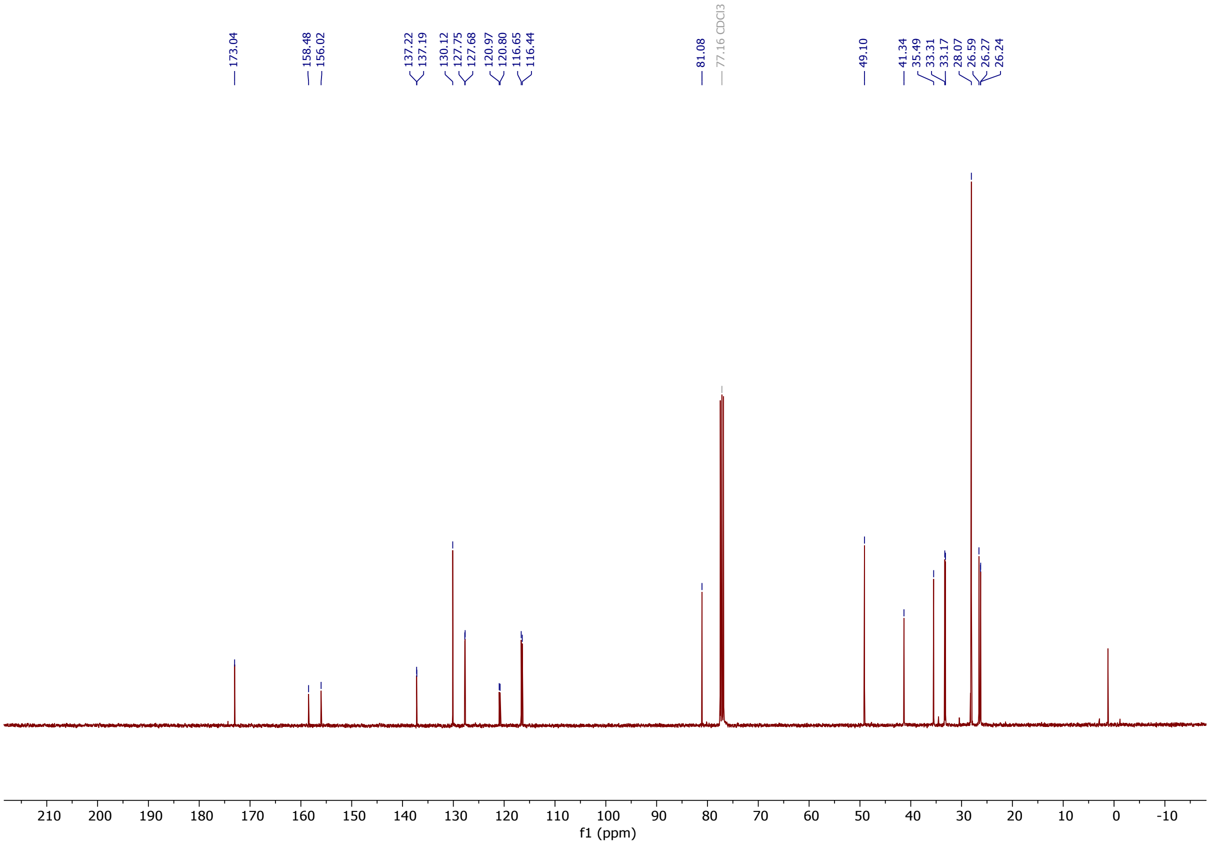

41, ^{19}F NMR (376 MHz, $CDCl_3$)

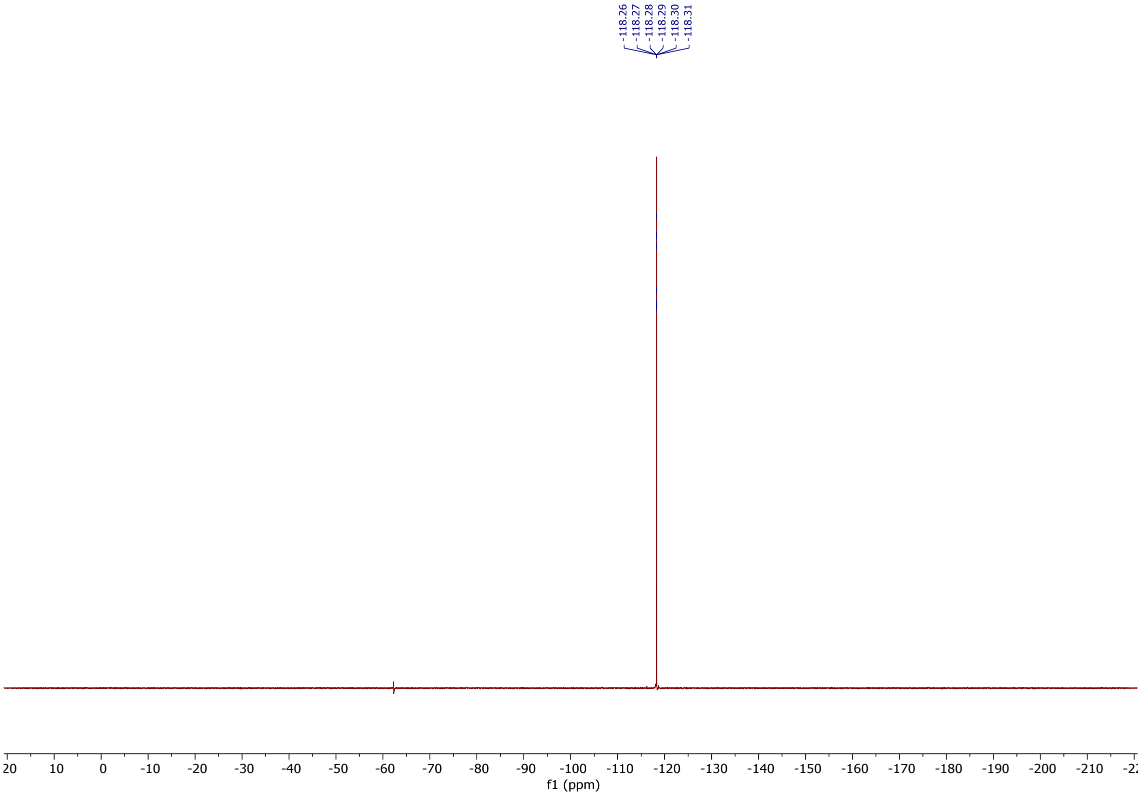

42, ^{1}H NMR (400 MHz, $CDCl_3$)

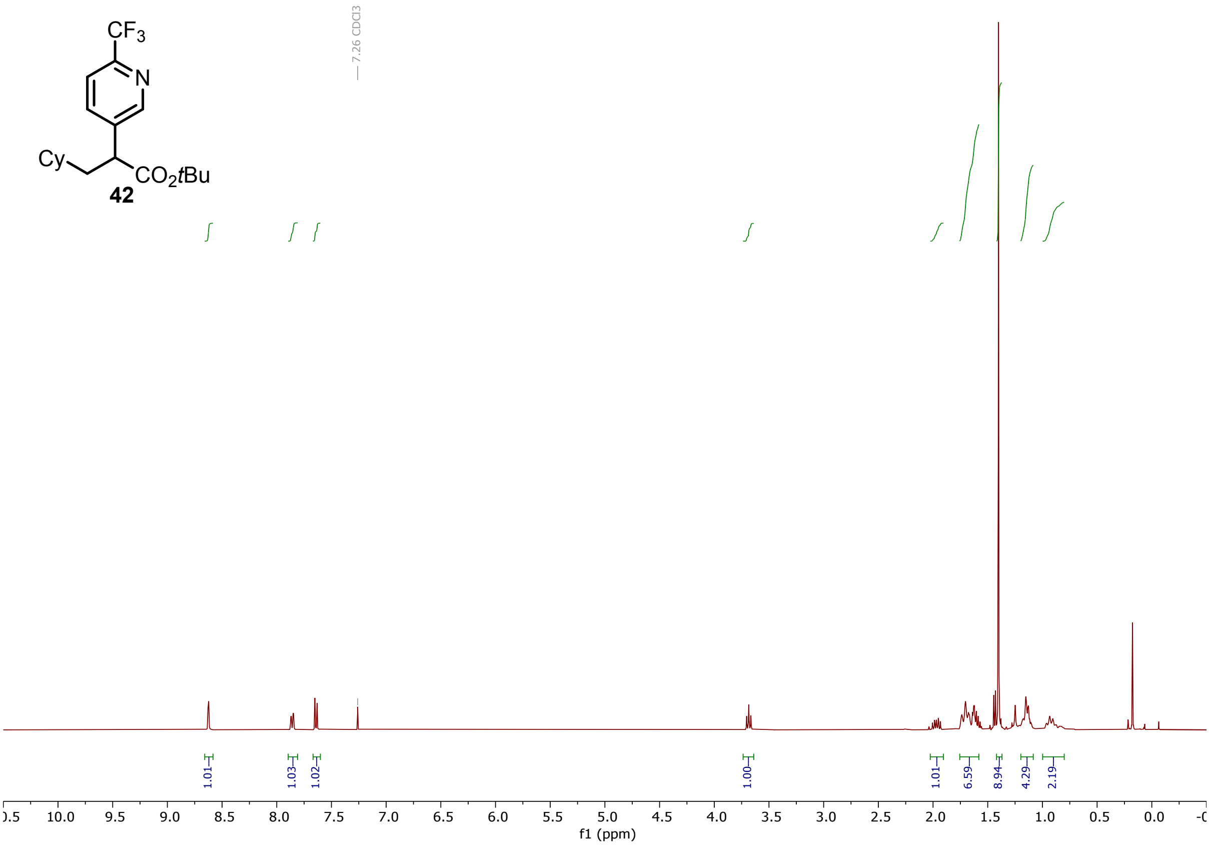

42, ^{13}C NMR (101 MHz, $CDCl_3$)

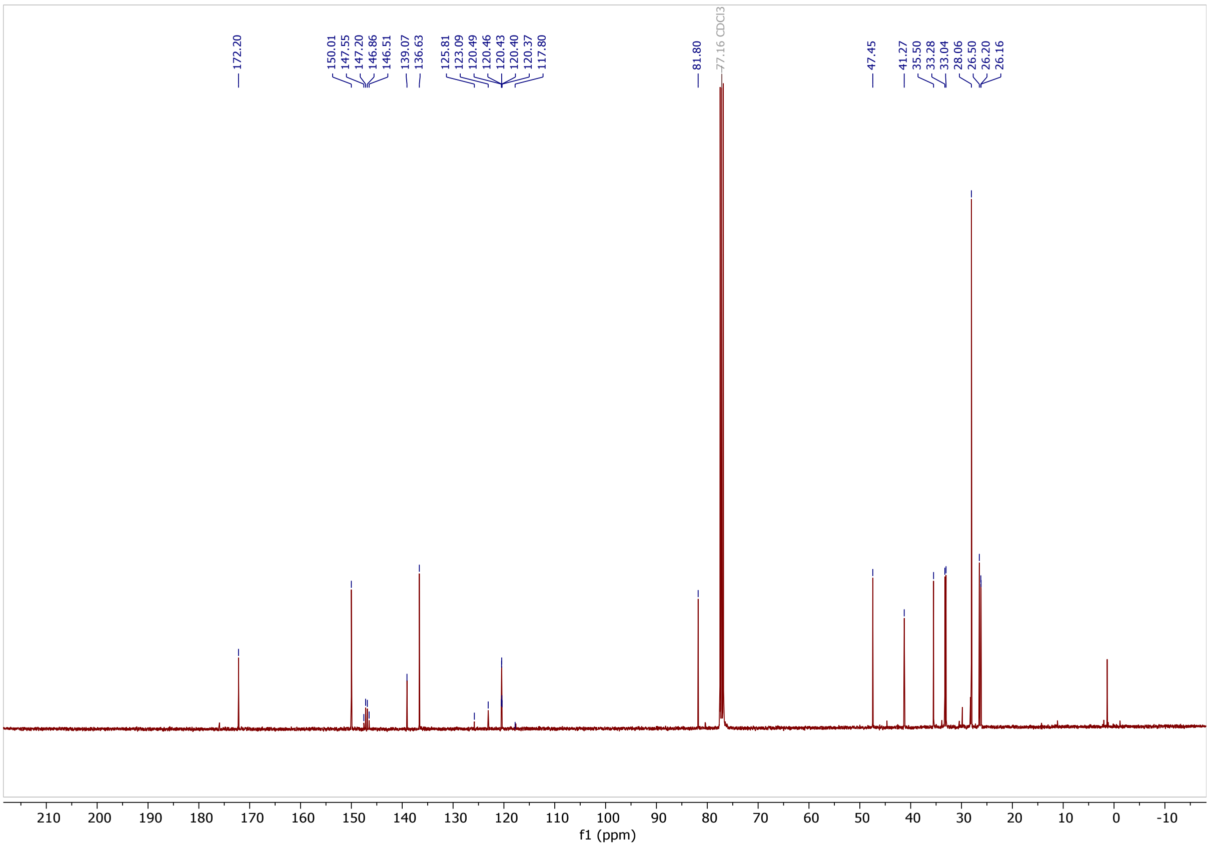

42, ^{19}F NMR (376 MHz, $CDCl_3$)

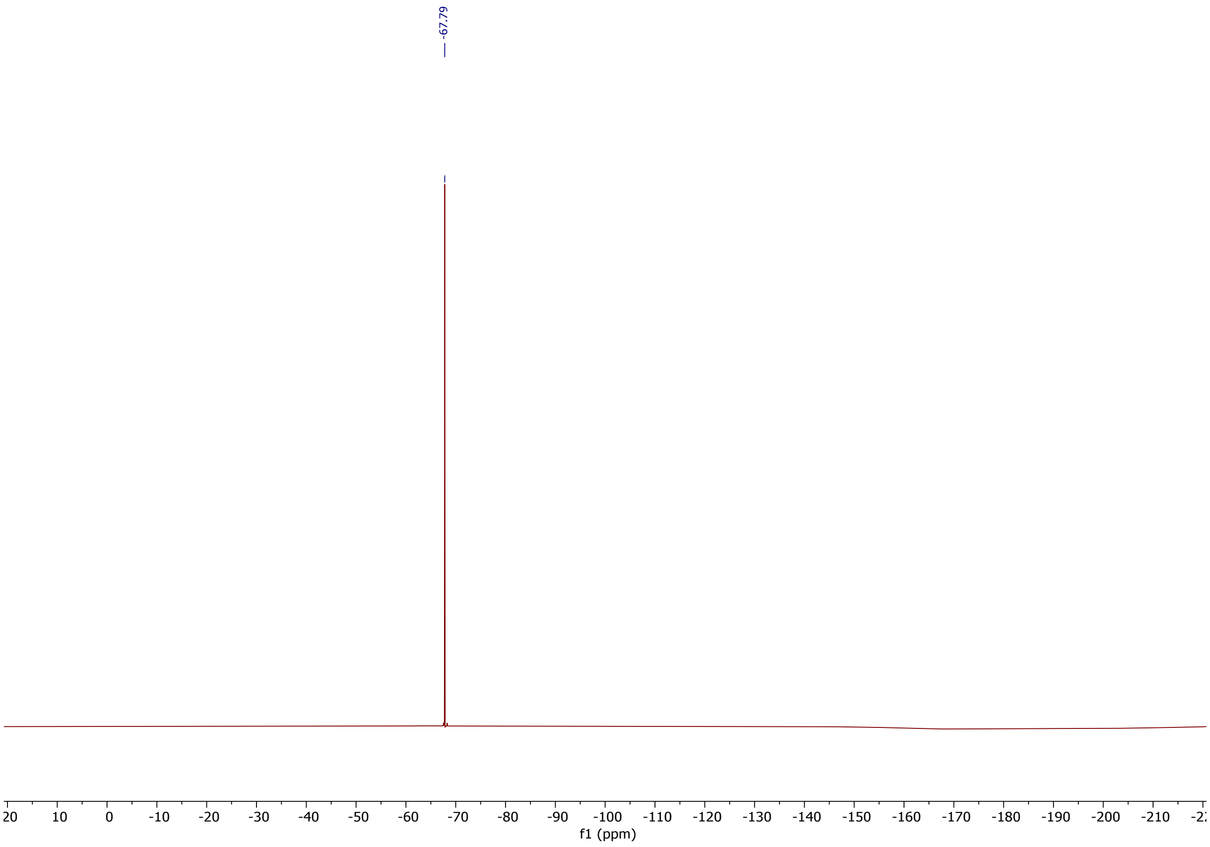

43, ^{1}H NMR (400 MHz, $CDCl_3$)

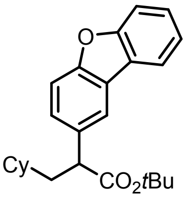

43

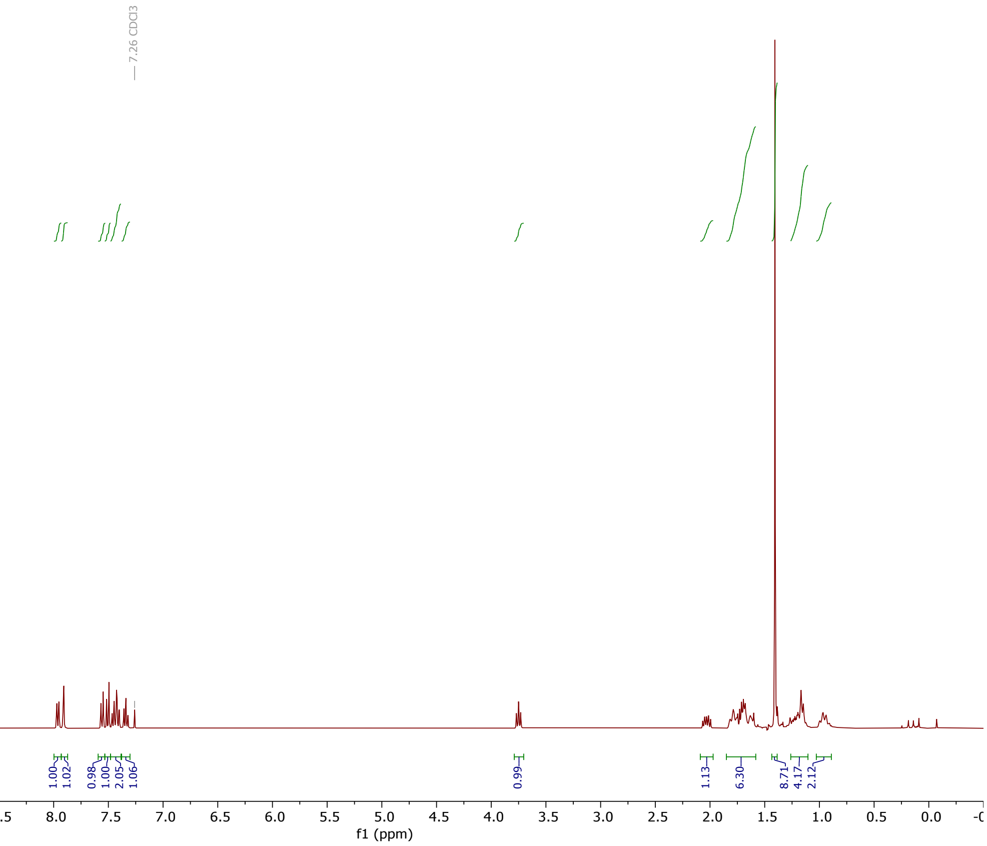

43, ^{13}C NMR (101 MHz, $CDCl_3$)

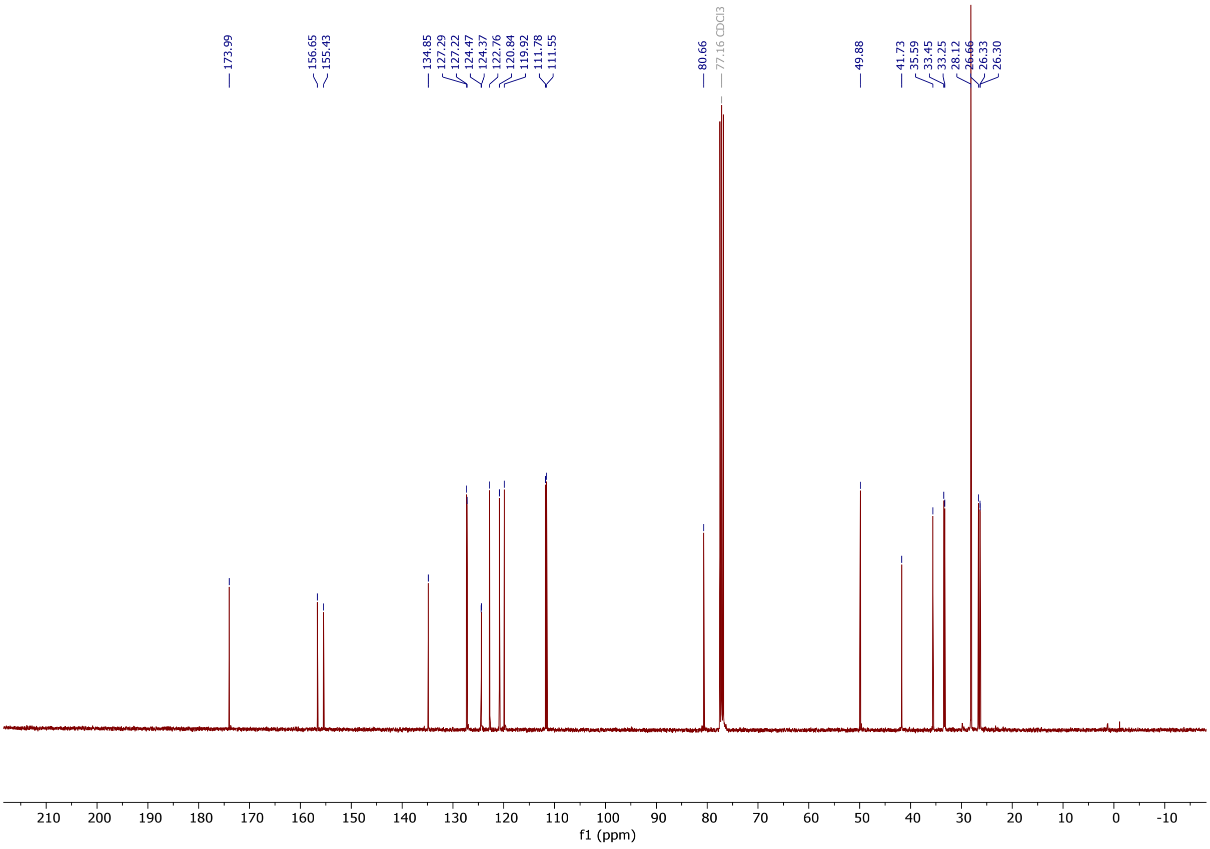

44, ^{1}H NMR (400 MHz, $CDCl_3$)

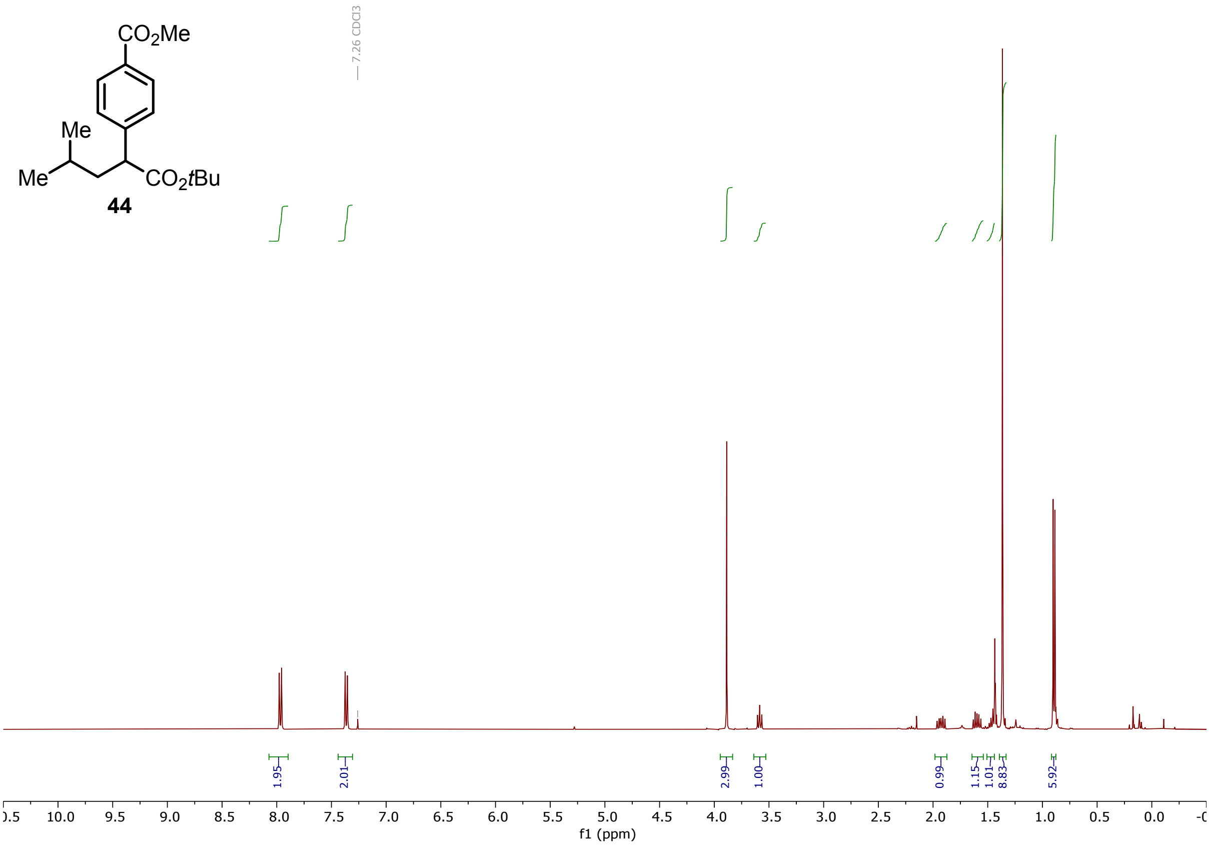

44, ^{13}C NMR (101 MHz, $CDCl_3$)

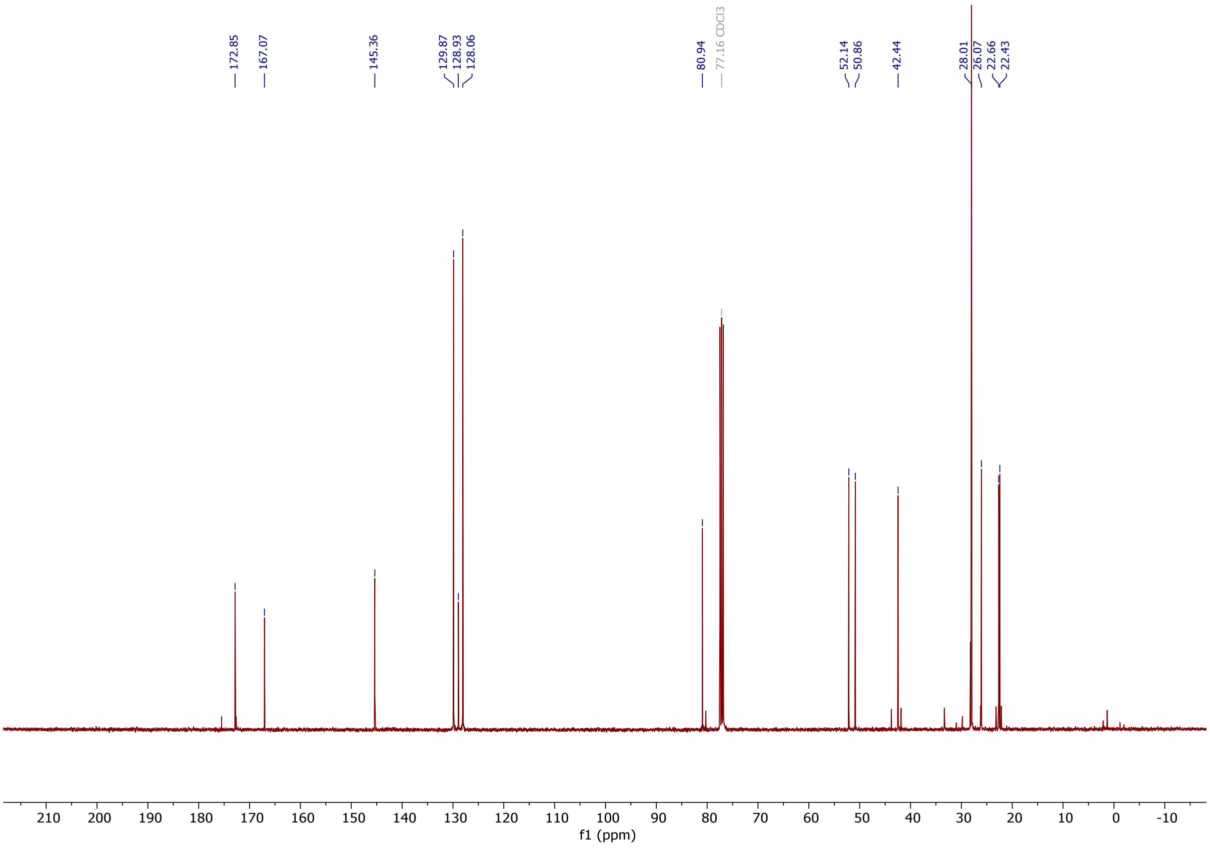

45, ^{1}H NMR (400 MHz, $CDCl_3$)

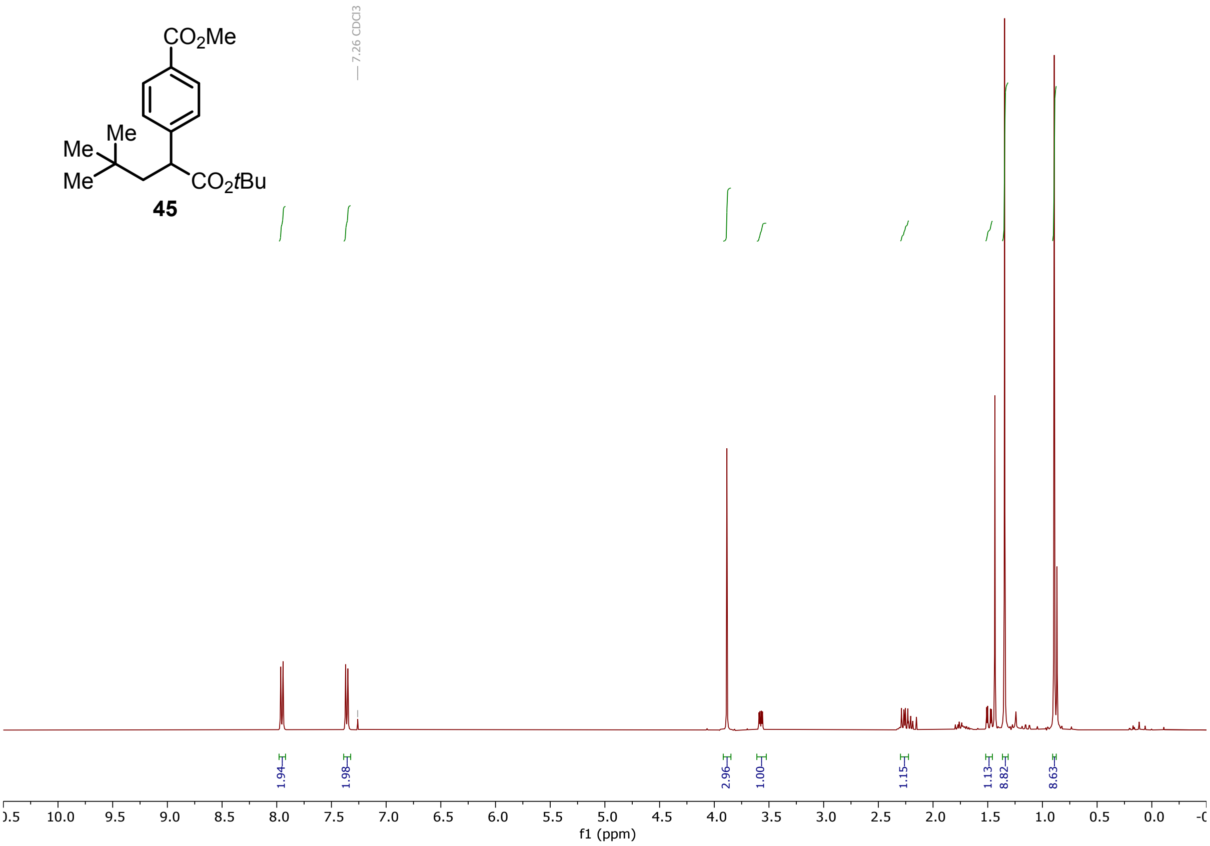

45, ^{13}C NMR (101 MHz, $CDCl_3$)

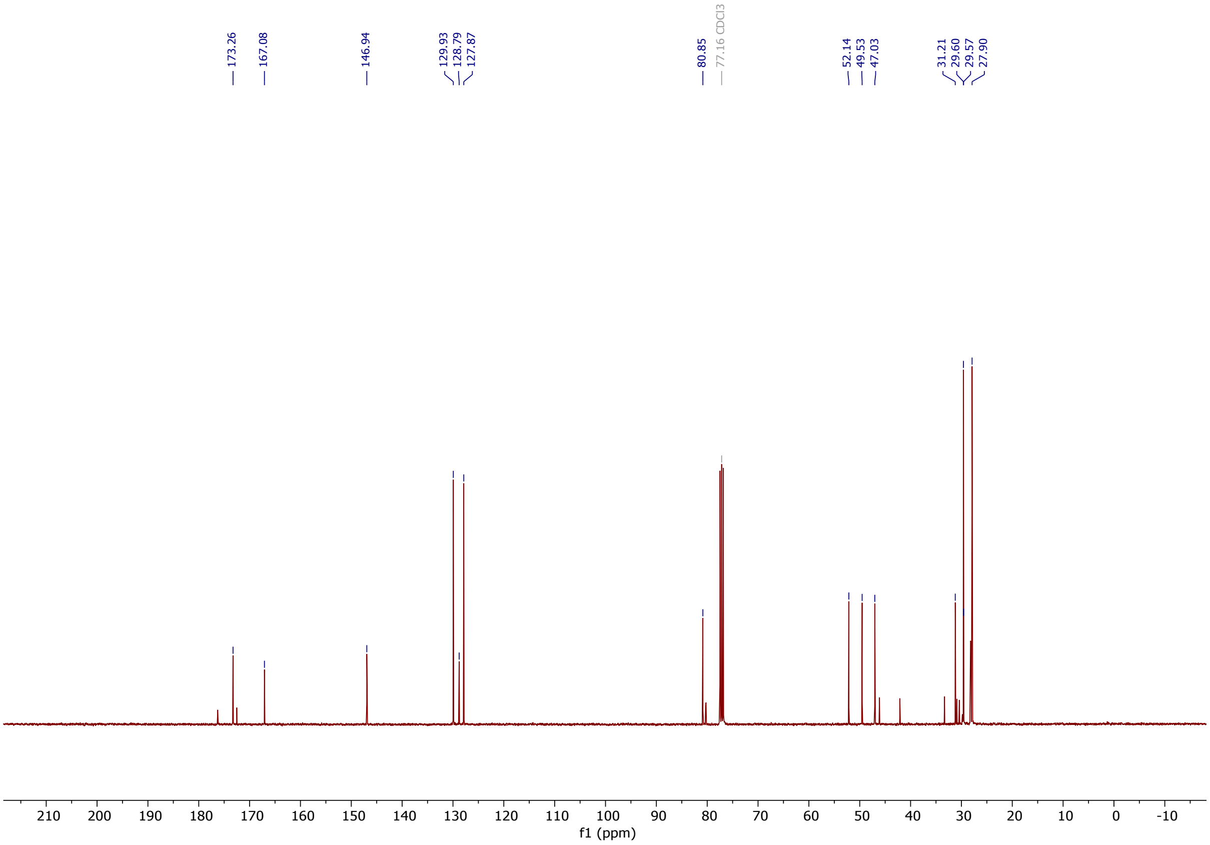

46, ^{1}H NMR (400 MHz, $CDCl_3$)

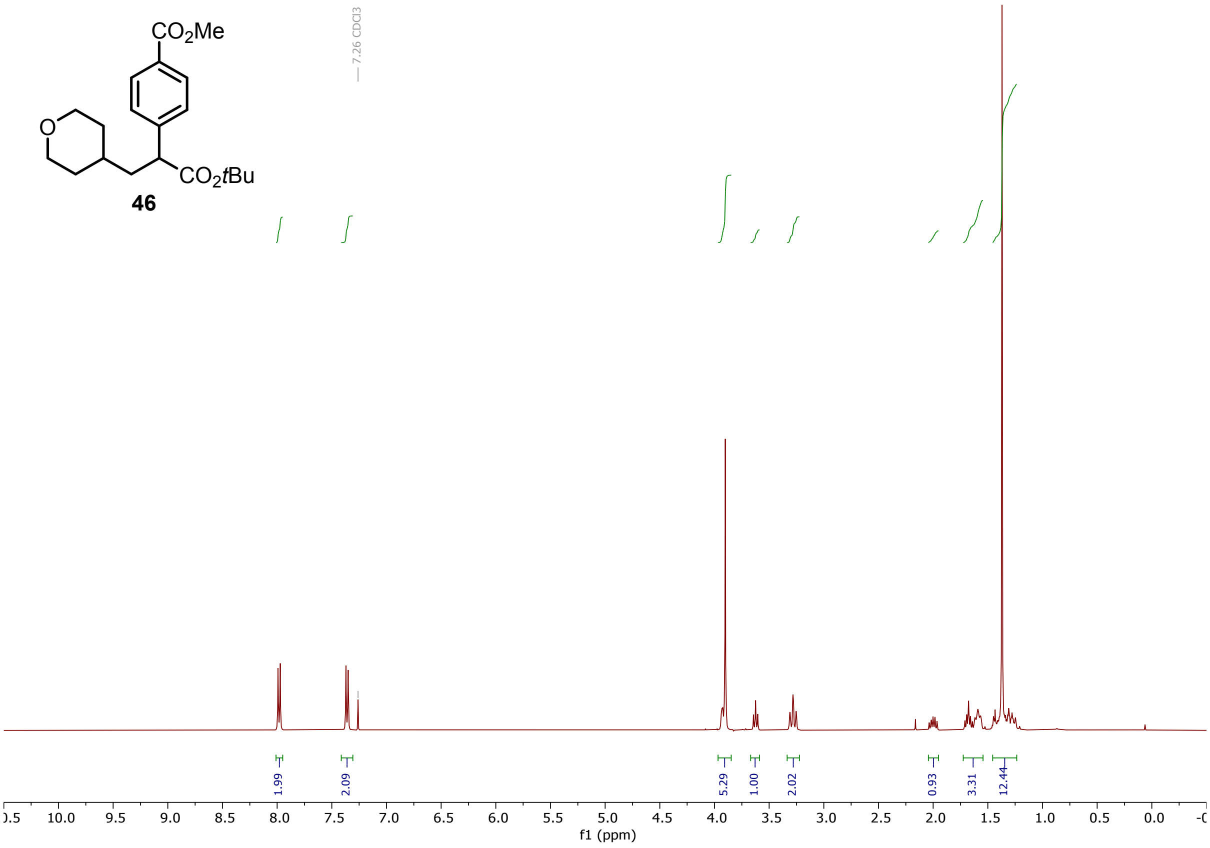

46, ^{13}C NMR (101 MHz, $CDCl_3$)

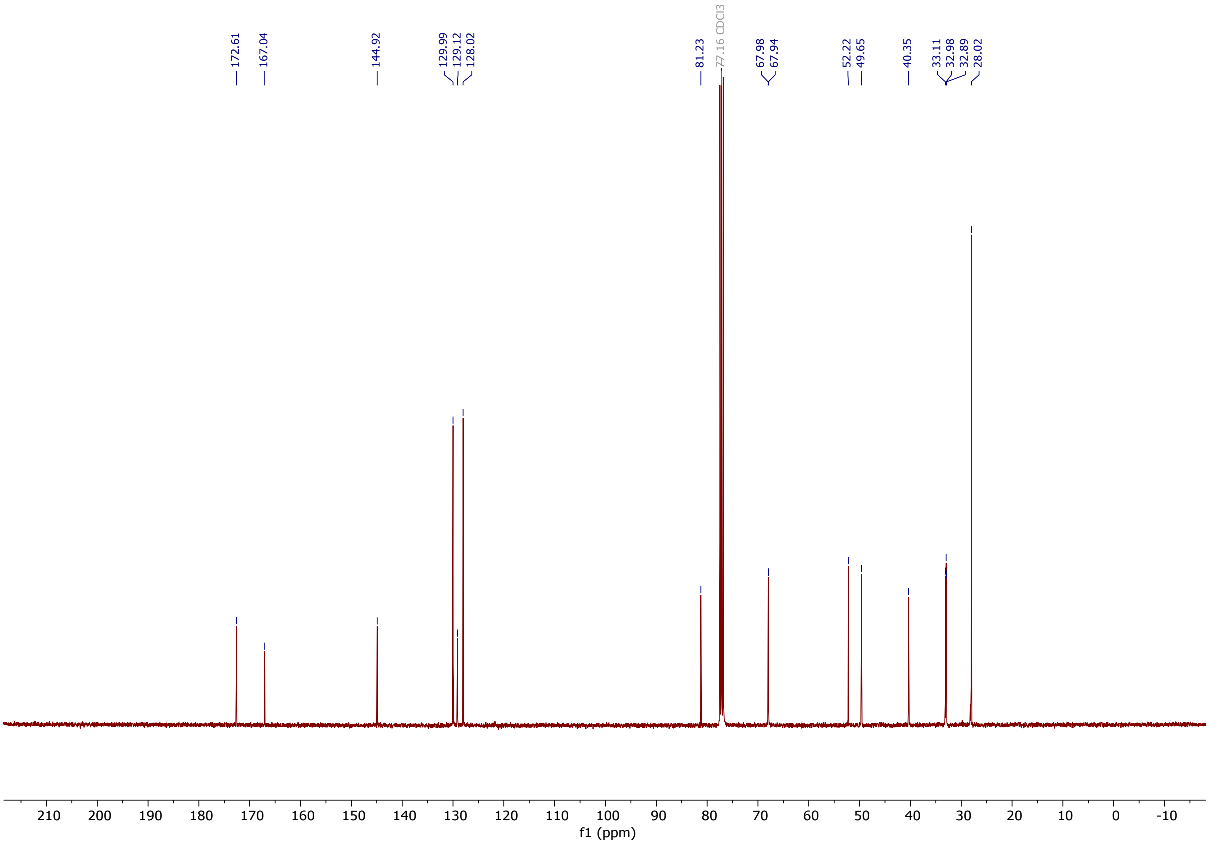

47, ^{1}H NMR (400 MHz, $CDCl_3$)

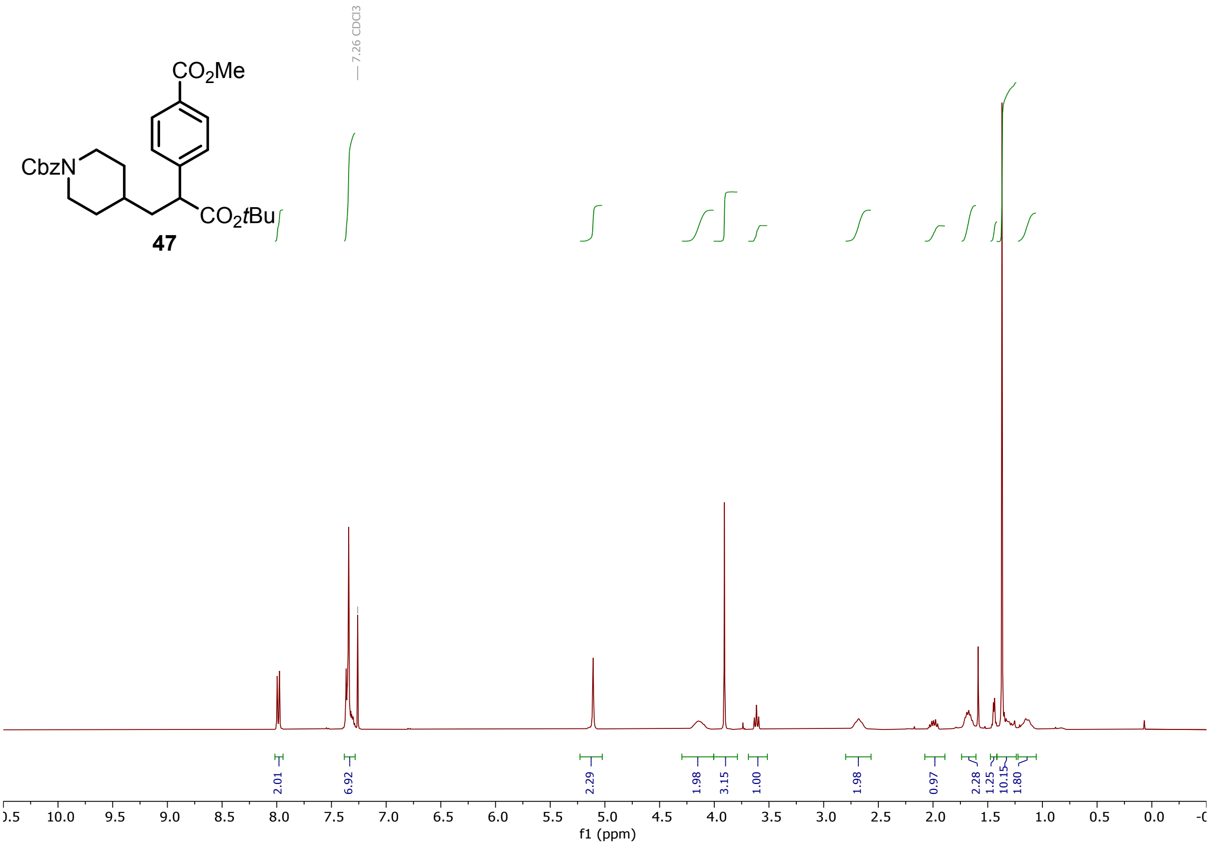

47, ^{13}C NMR (101 MHz, $CDCl_3$)

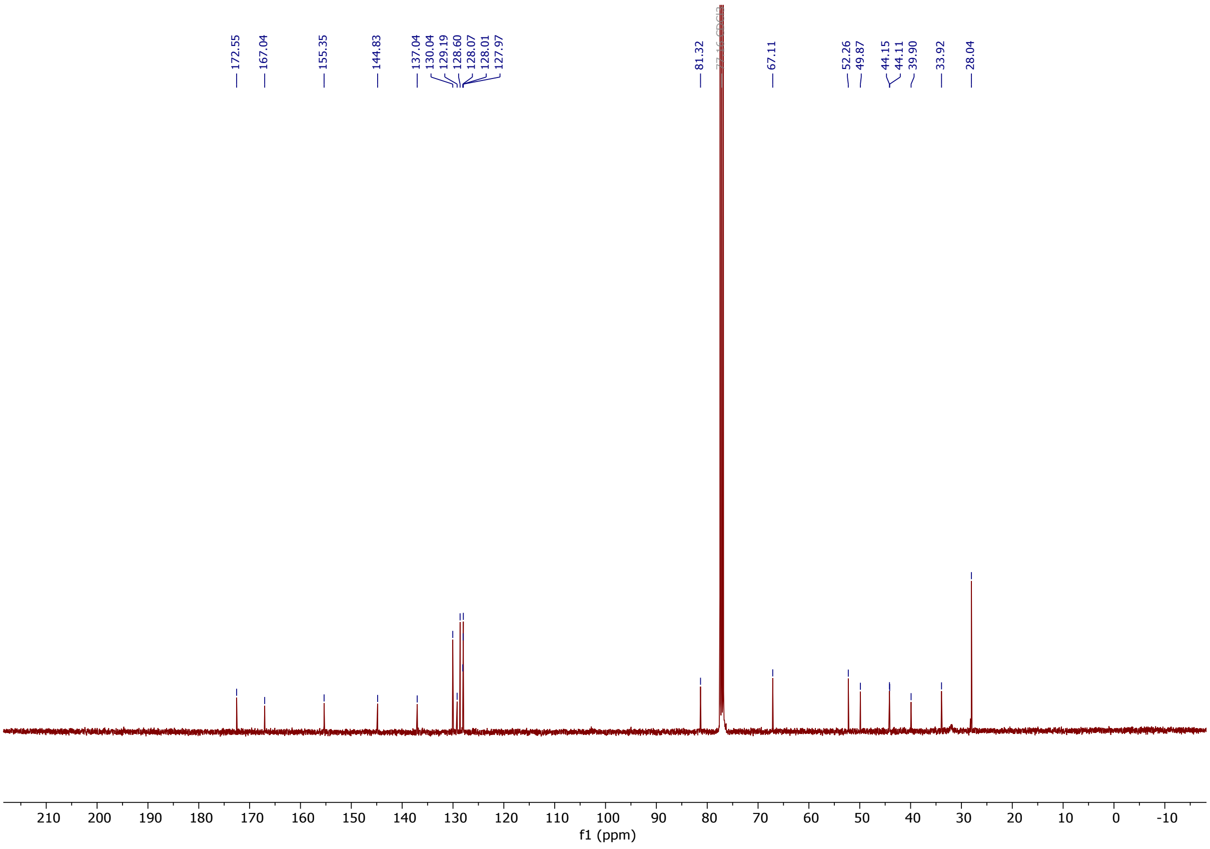

48, ^{1}H NMR (400 MHz, $CDCl_3$)

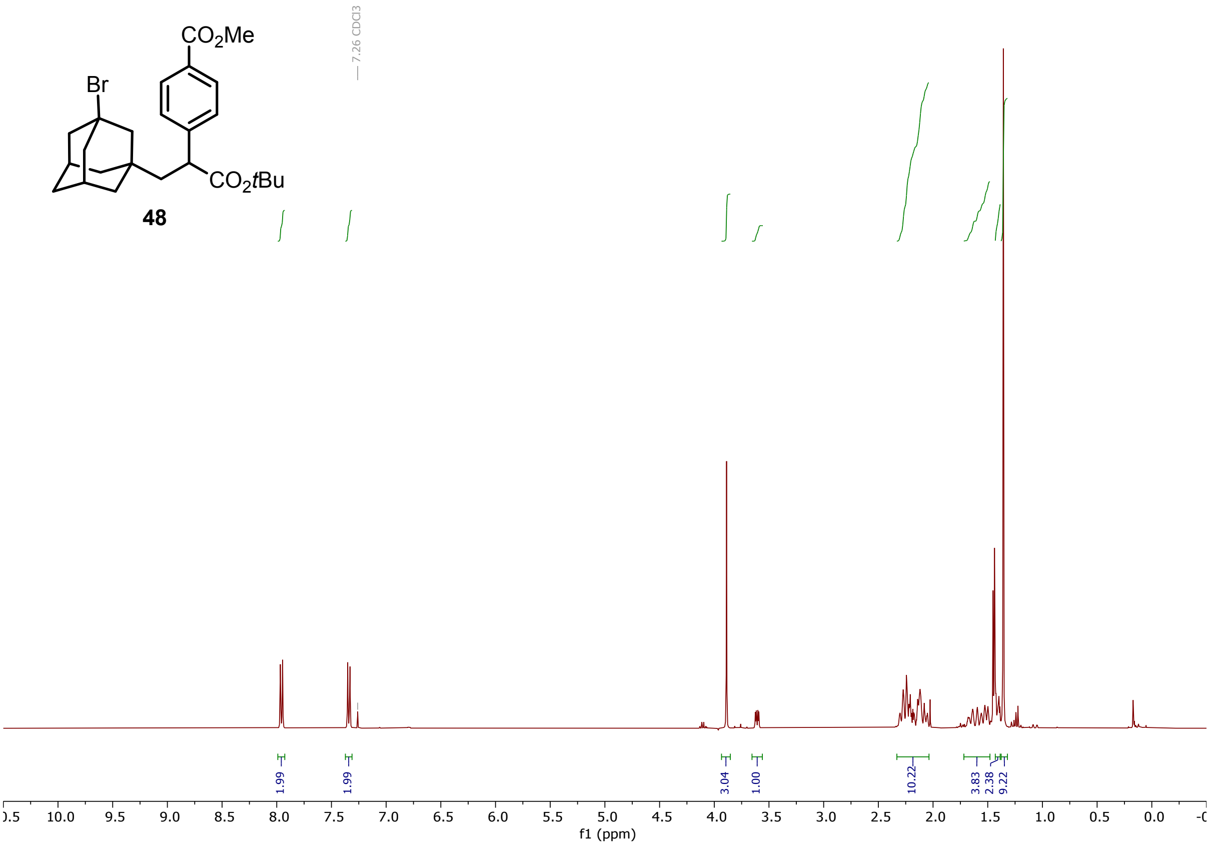

48, ^{13}C NMR (101 MHz, $CDCl_3$)

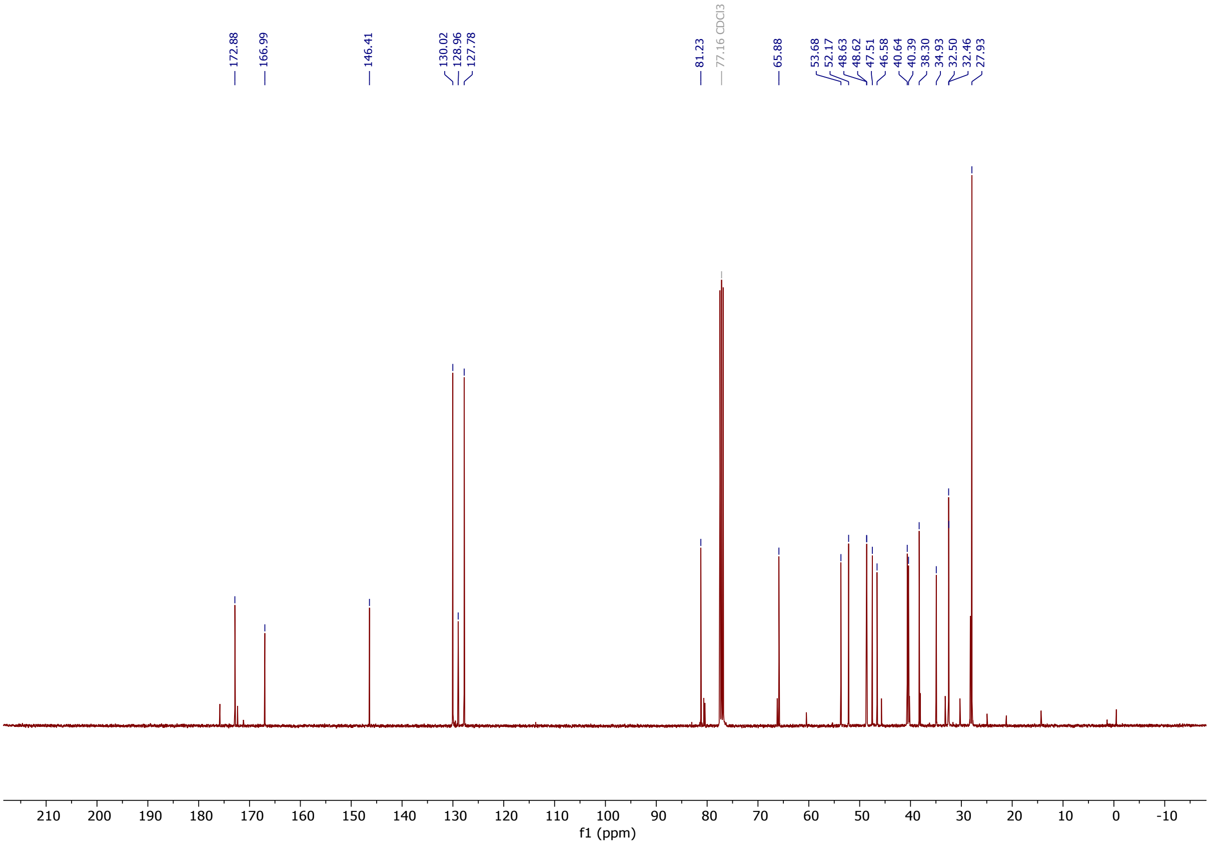

49, ^{1}H NMR (400 MHz, $CDCl_3$)

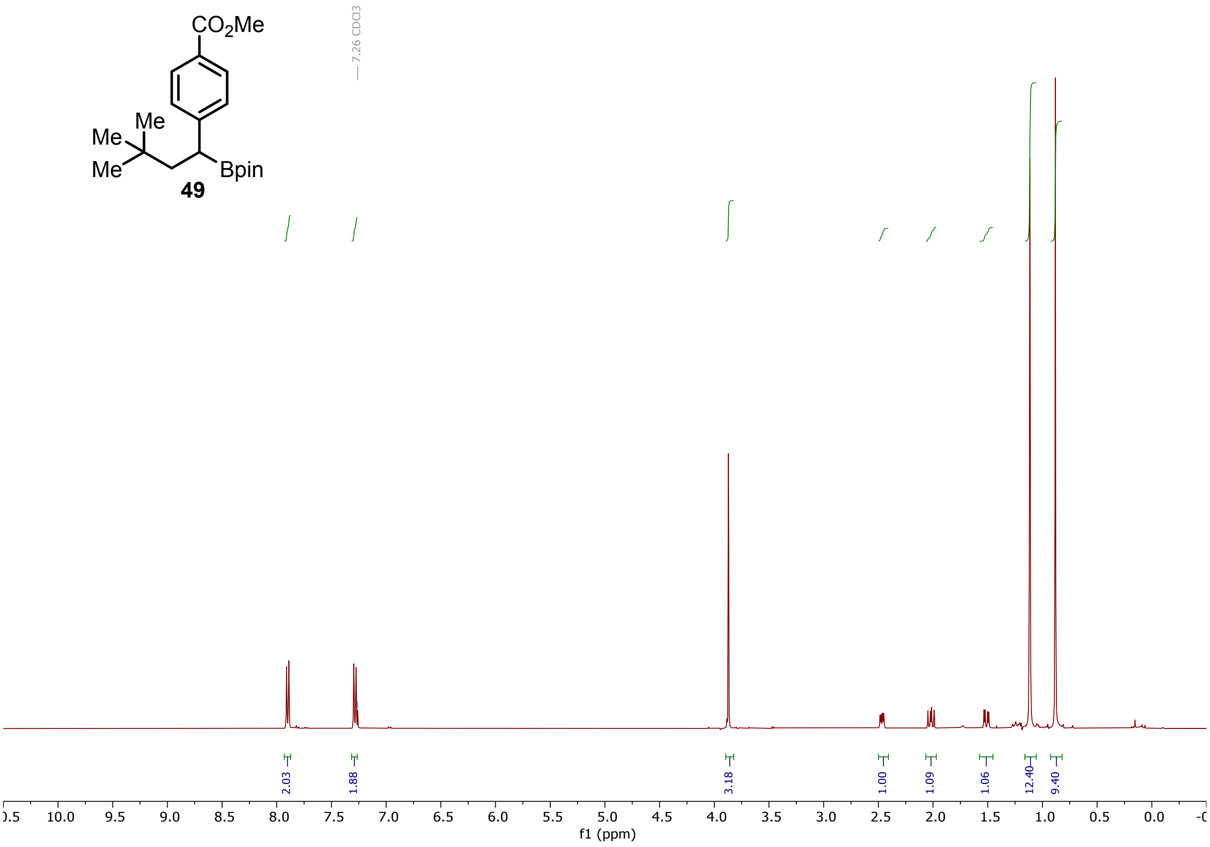

49, ^{13}C NMR (101 MHz, $CDCl_3$)

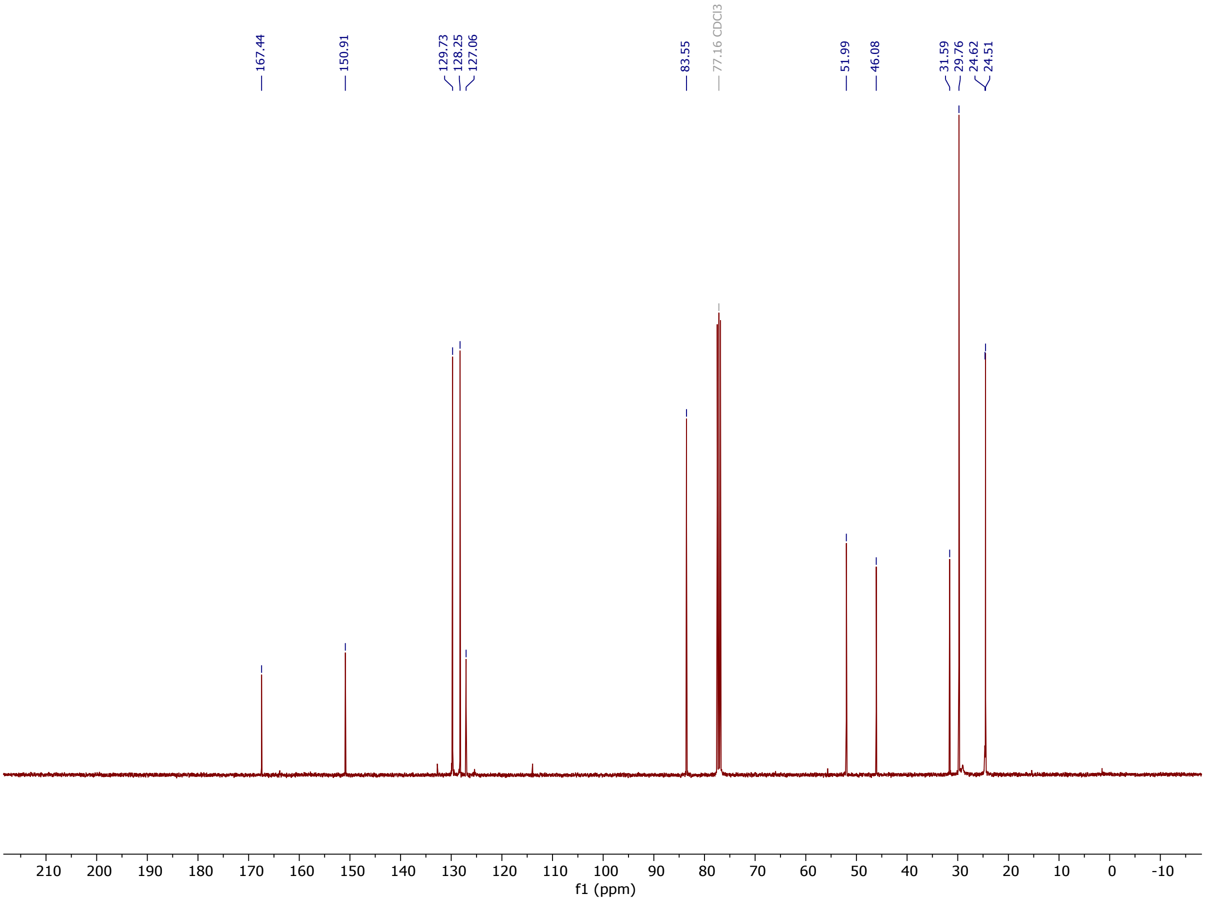

50, ^{1}H NMR (400 MHz, $CDCl_3$)

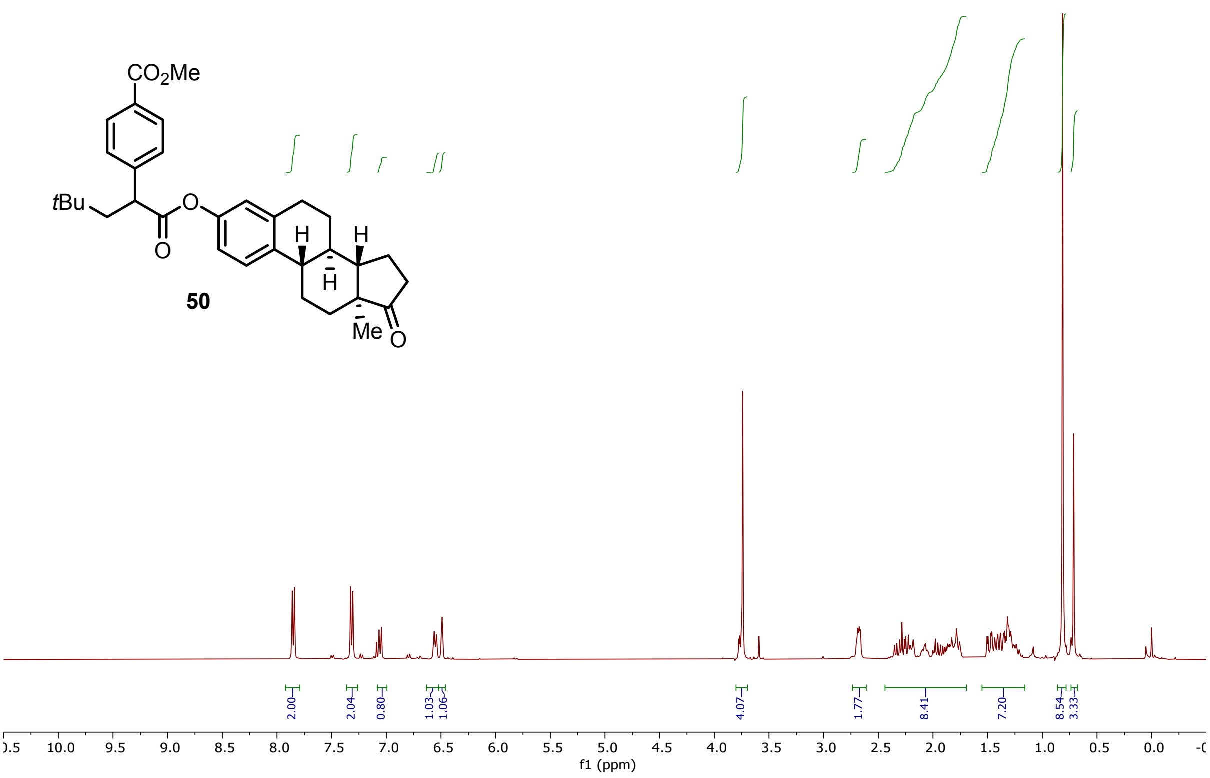

50, ^{13}C NMR (75 MHz, $CDCl_3$)

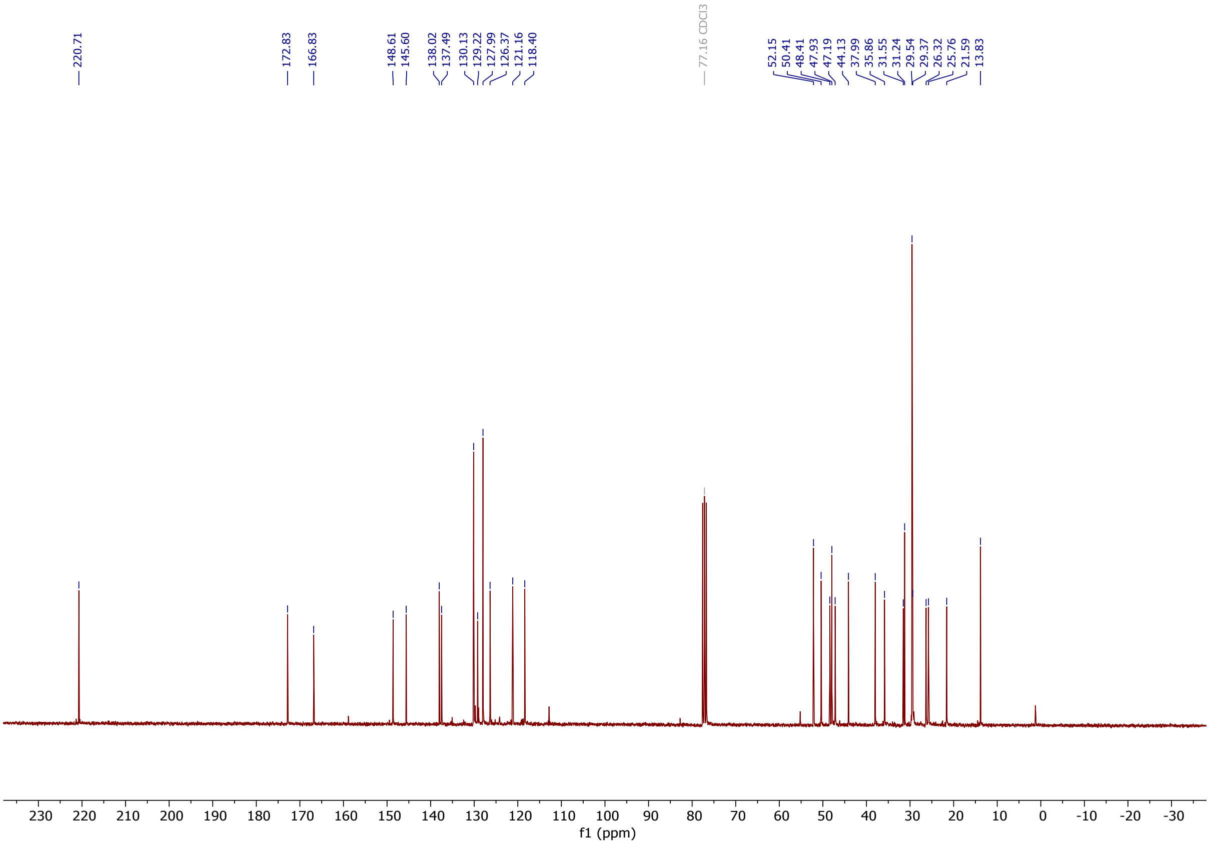

51, ^{1}H NMR (400 MHz, $CDCl_3$)

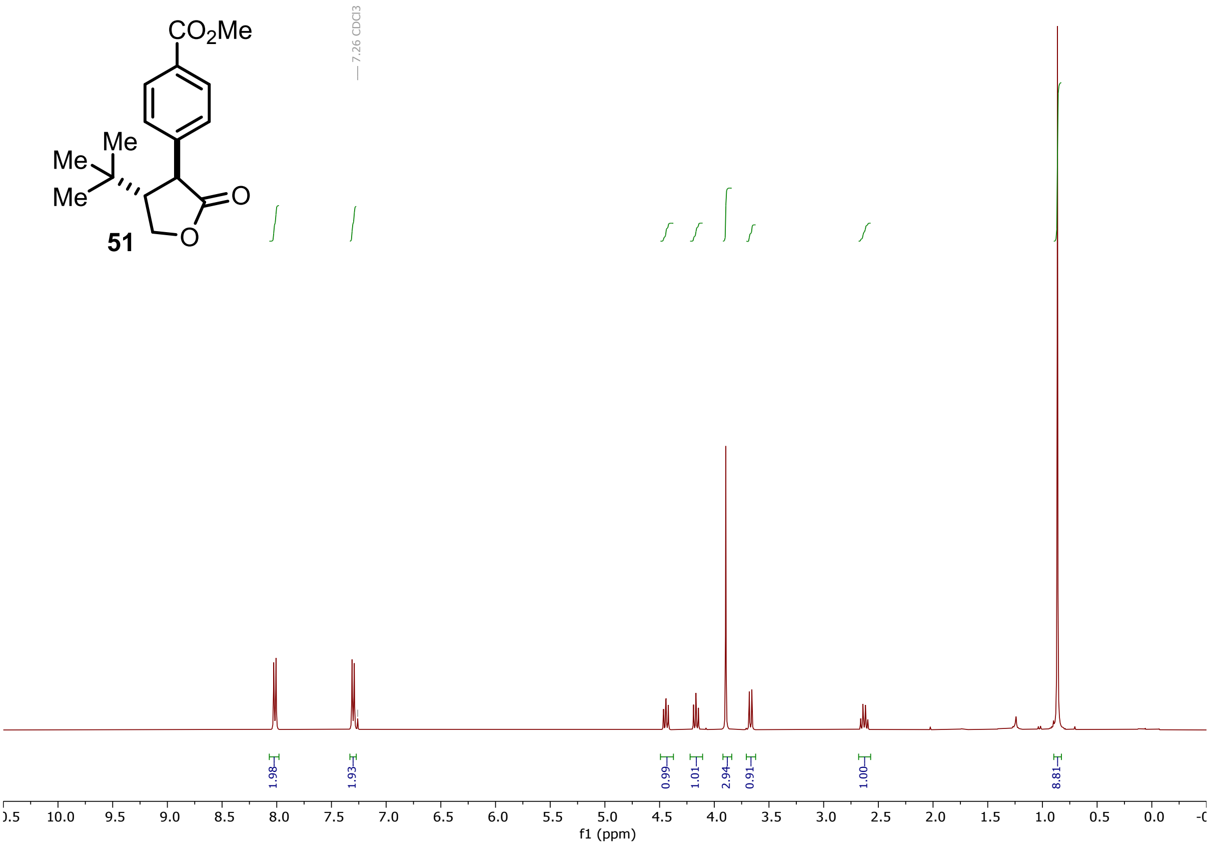

51, ^{13}C NMR (101 MHz, $CDCl_3$)

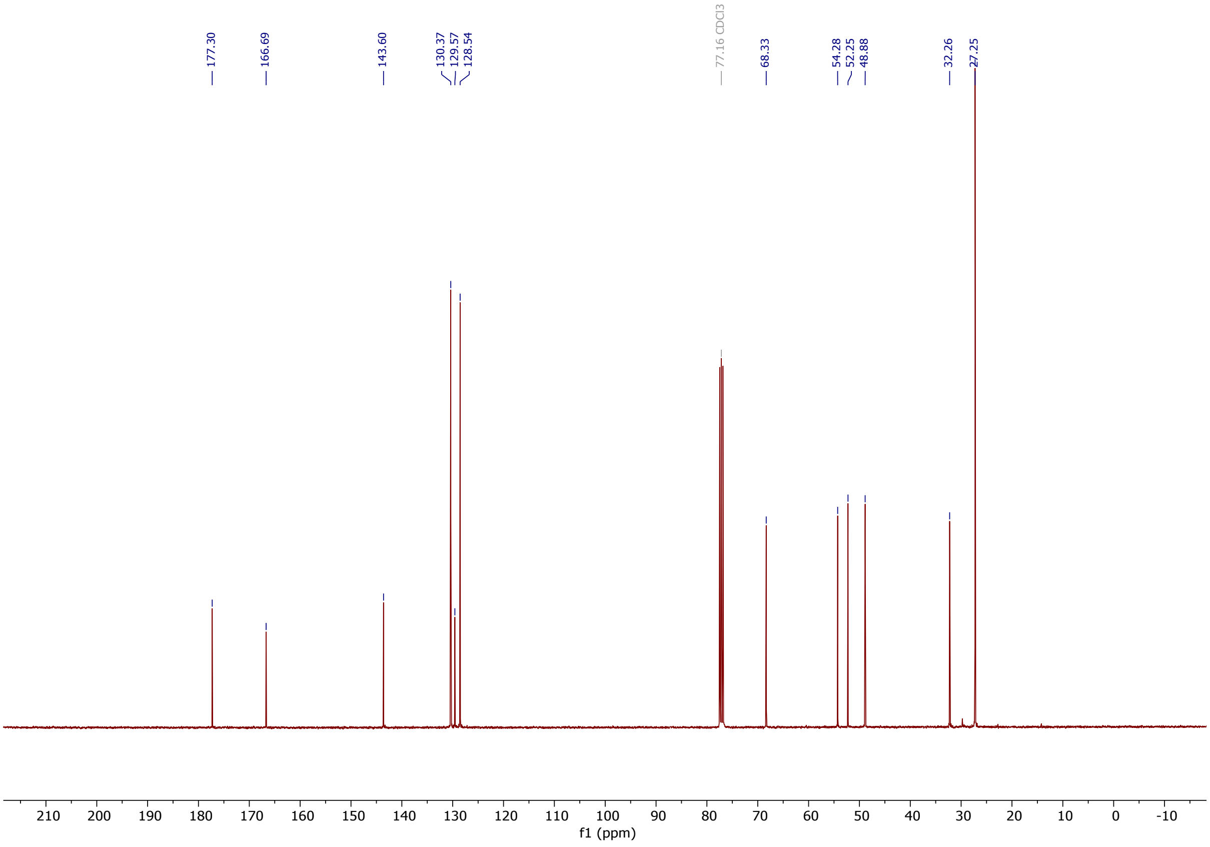

51, NOESY

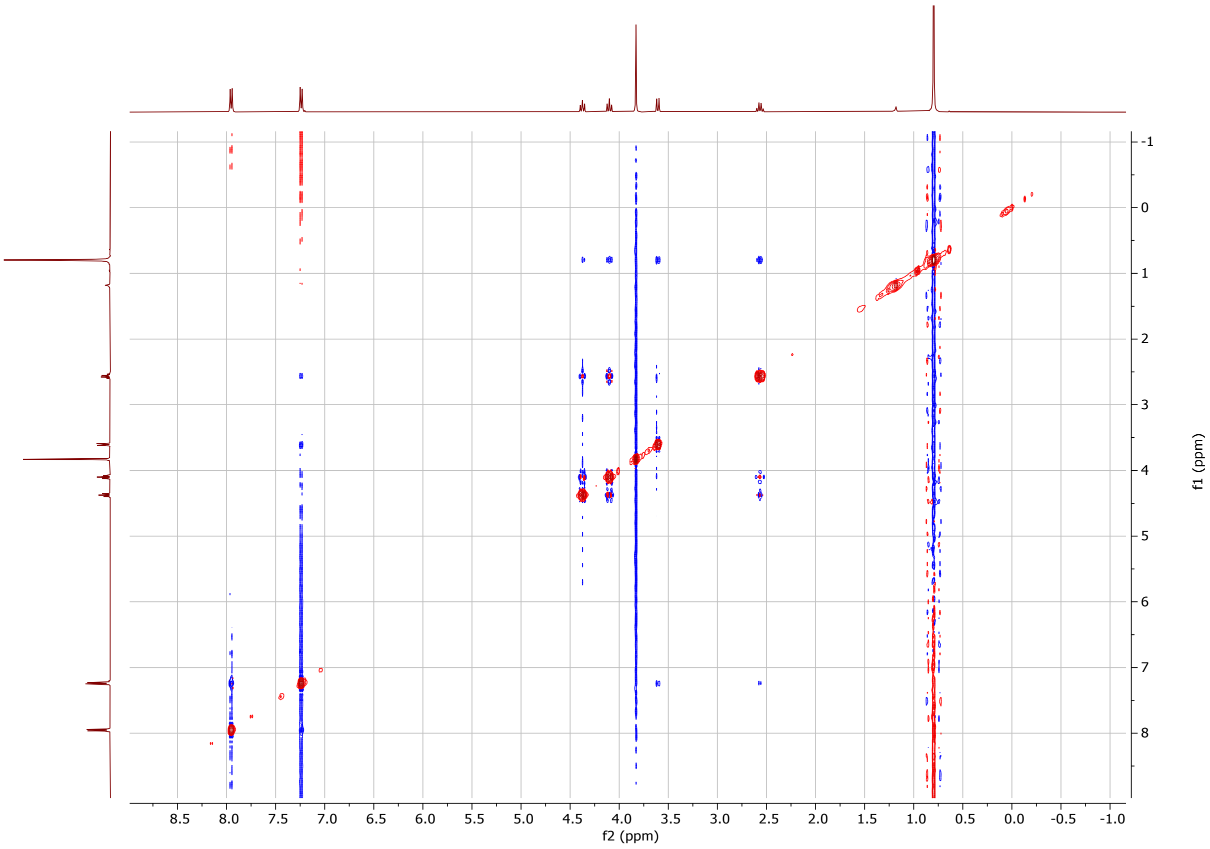

8.5
8.0
7.5
7.0
6.5
6.0
5.5
5.0
4.5
4.0
3.5
3.0
2.5
2.0
1.5
1.0
0.5
0.0
-0.5
-1.0
f2 (ppm)
-1
0
1
2
3
4
5
6
7
8
f1 (ppm)

52, ^{1}H NMR (400 MHz, $CDCl_3$)

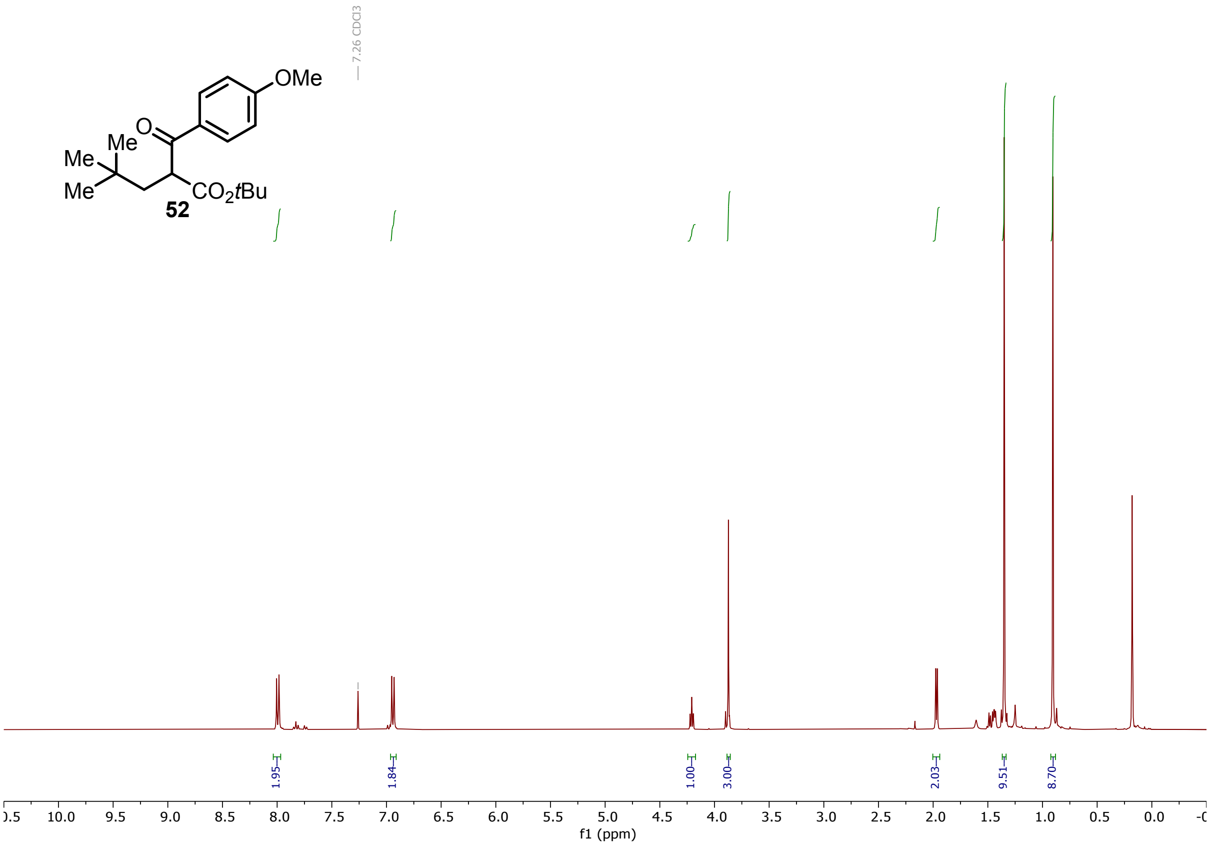

52, ^{13}C NMR (101 MHz, $CDCl_3$)

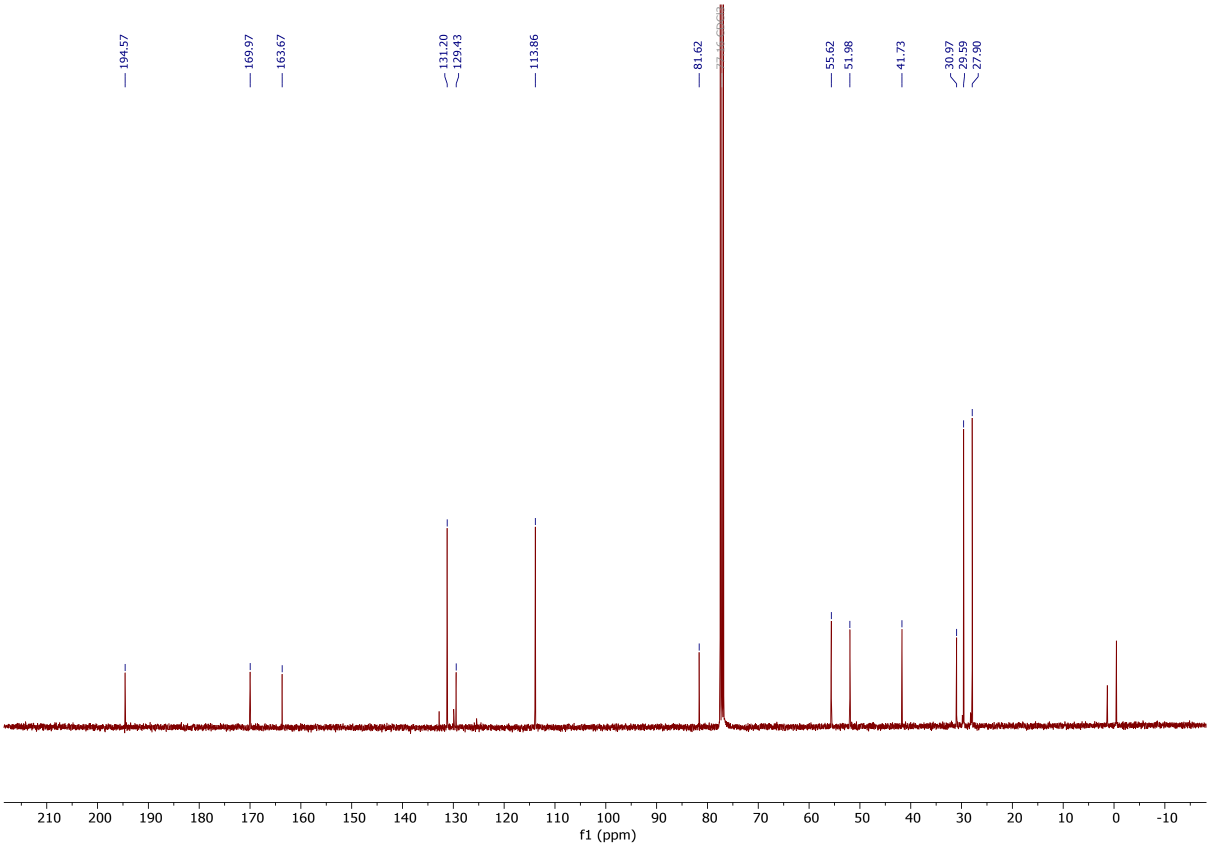

53, ^{1}H NMR (400 MHz, $CDCl_3$)

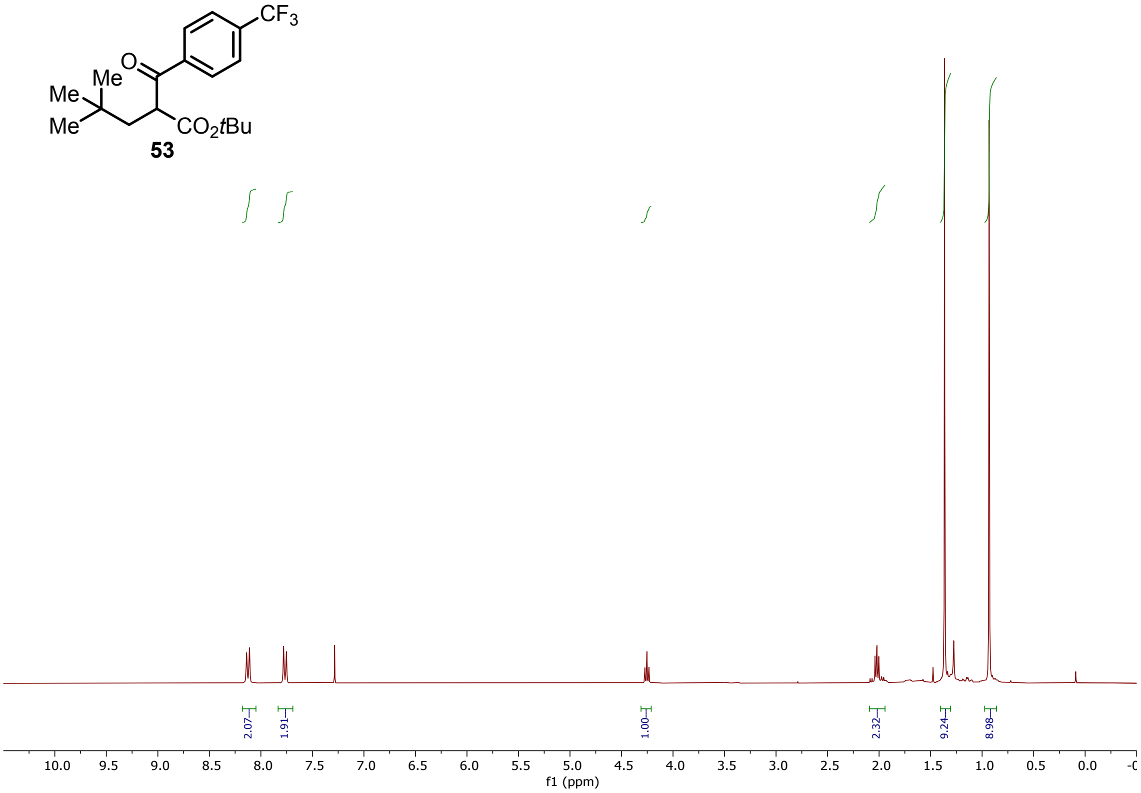

53, ^{13}C NMR (101 MHz, $CDCl_3$)

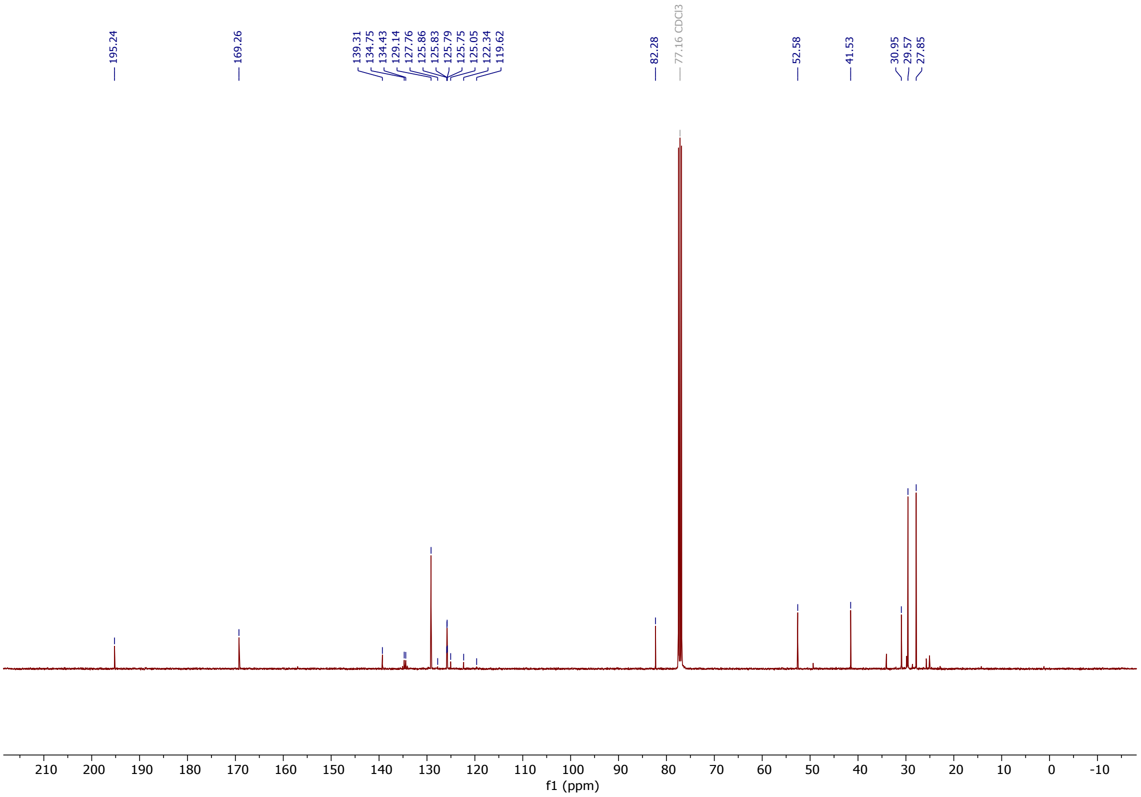

53, ^{19}F NMR (376 MHz, $CDCl_3$)

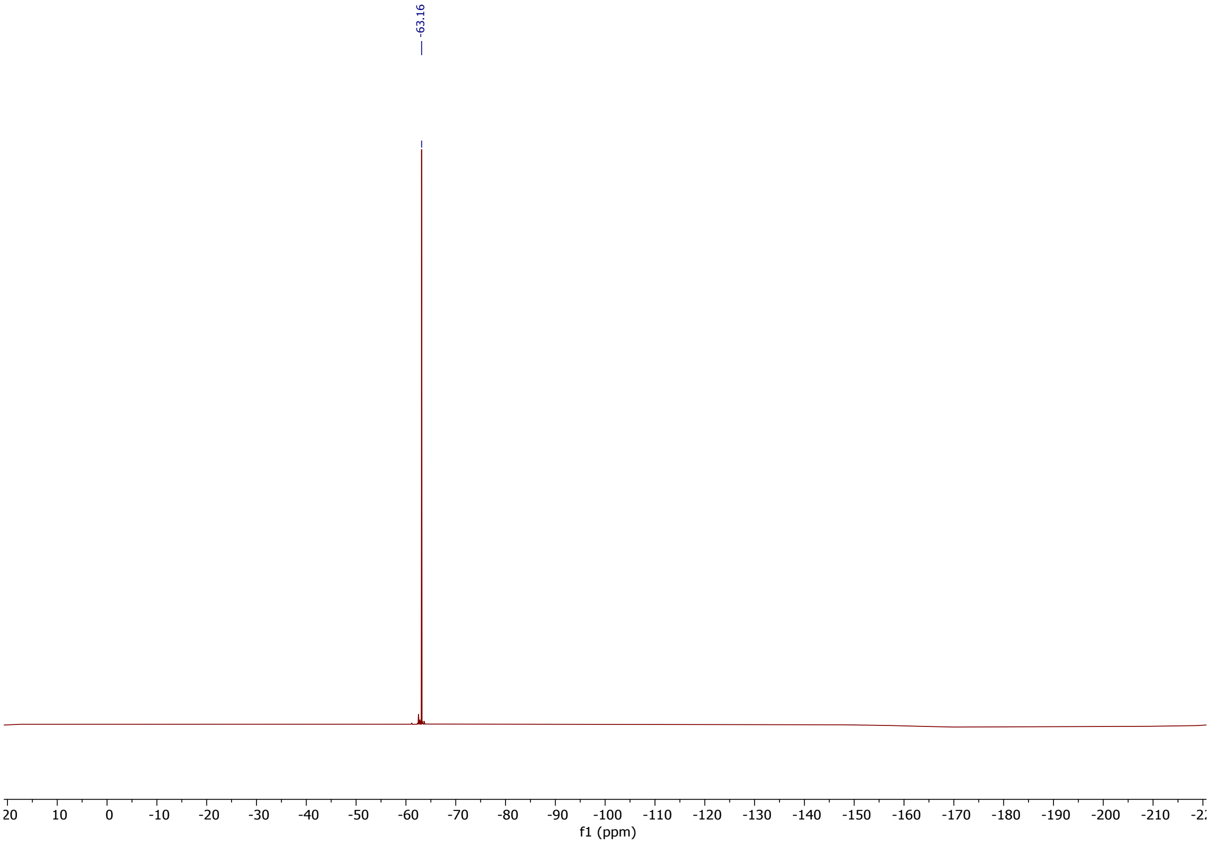

54, ^{1}H NMR (300 MHz, $CDCl_3$)

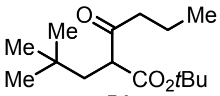

54

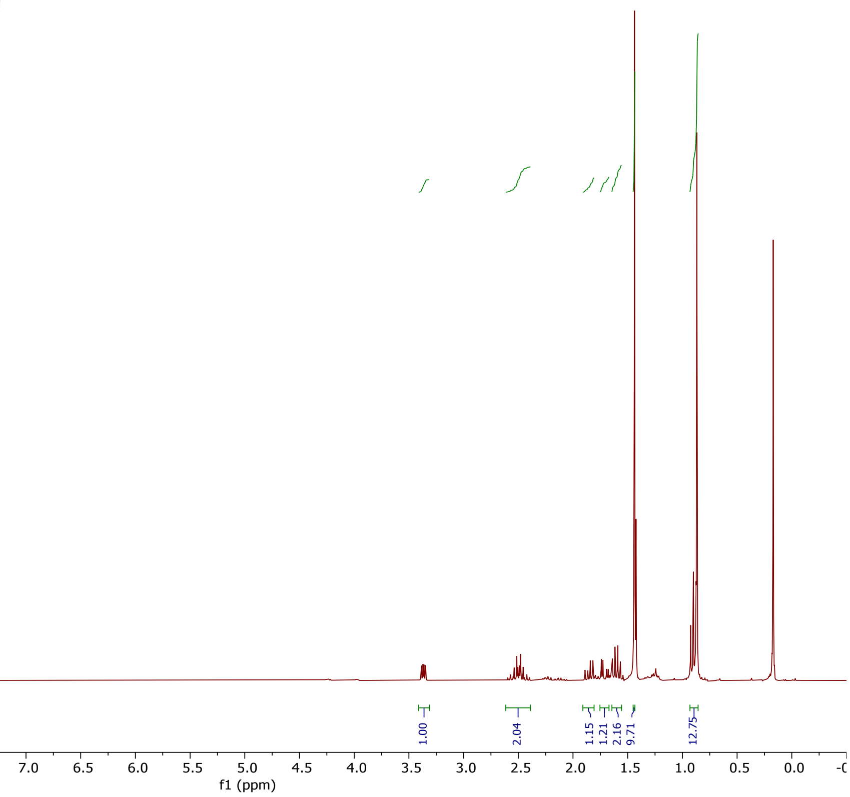

54, ^{13}C NMR (101 MHz, $CDCl_3$)

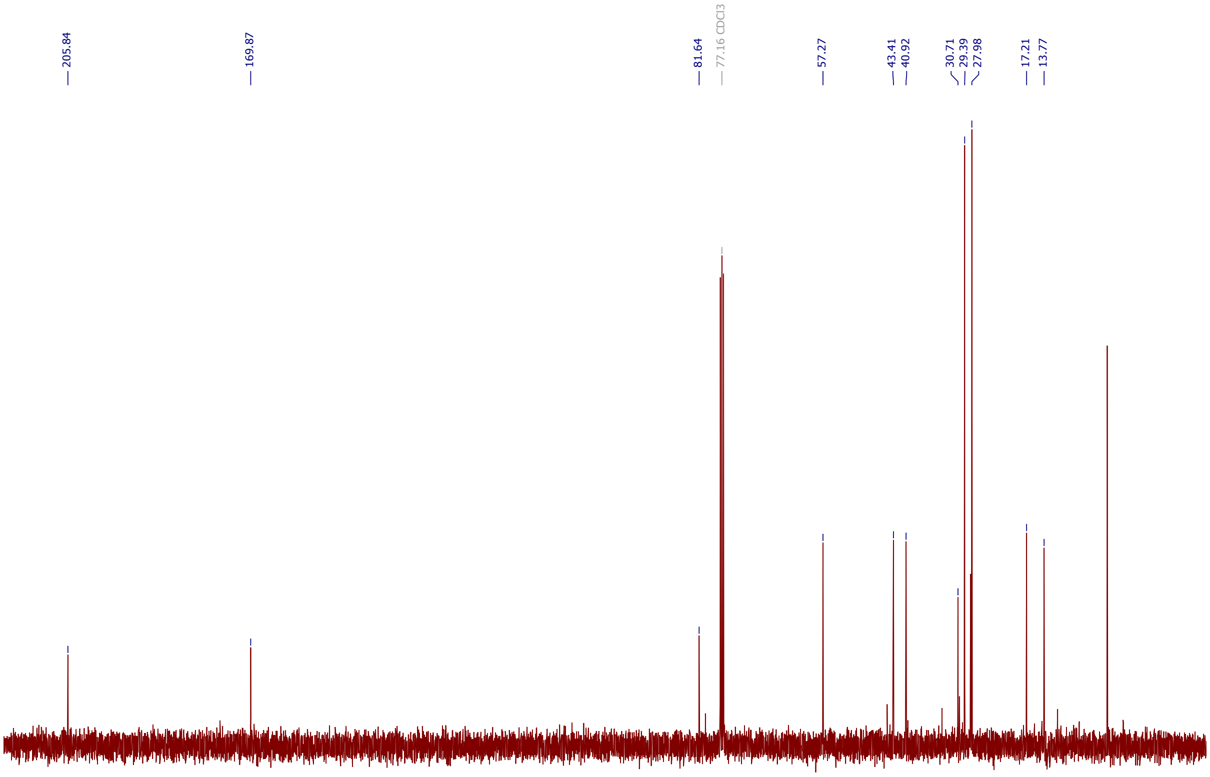

Supplement: Supplementary file 1 — cs2c03805_si_001.pdf [file cs2c03805_si_001.pdf]
